# Under control: how a dietary additive can restore the gut microbiome and proteomic profile, and improve disease resilience in a marine teleostean fish fed vegetable diets

**DOI:** 10.1186/s40168-017-0390-3

**Published:** 2017-12-28

**Authors:** María Carla Piazzon, Josep Alvar Calduch-Giner, Belén Fouz, Itziar Estensoro, Paula Simó-Mirabet, Mónica Puyalto, Vasileios Karalazos, Oswaldo Palenzuela, Ariadna Sitjà-Bobadilla, Jaume Pérez-Sánchez

**Affiliations:** 1 Fish Pathology Group, Instituto de Acuicultura Torre de la Sal (IATS-CSIC), Castellón, Spain; 2 Nutrigenomics and Fish Growth Endocrinology Group, Instituto de Acuicultura Torre de la Sal (IATS-CSIC), Castellón, Spain; 3 Department of Microbiology and Ecology, Faculty of Biology, University of Valencia, Valencia, Spain; 4 Norel S.A., Madrid, Spain; 5 BioMar R&D, Grangemouth, UK

**Keywords:** *Sparus aurata*, Intestinal health, Microbiome, Proteome, Sodium butyrate, Nutrition, *Enteromyxum leei*, Myxozoa, *Photobacterium*, Pathogen challenge

## Abstract

**Background:**

The constant increase of aquaculture production and wealthy seafood consumption has forced the industry to explore alternative and more sustainable raw aquafeed materials, and plant ingredients have been used to replace marine feedstuffs in many farmed fish. The objective of the present study was to assess whether plant-based diets can induce changes in the intestinal mucus proteome, gut autochthonous microbiota and disease susceptibility of fish, and whether these changes could be reversed by the addition of sodium butyrate to the diets. Three different trials were performed using the teleostean gilthead sea bream (*Sparus aurata*) as model. In a first preliminary short-term trial, fish were fed with the additive (0.8%) supplementing a basal diet with low vegetable inclusion (D1) and then challenged with a bacteria to detect possible effects on survival. In a second trial, fish were fed with diets with greater vegetable inclusion levels (D2, D3) and the long-term effect of sodium butyrate at a lower dose (0.4%) added to D3 (D4 diet) was tested on the intestinal proteome and microbiome. In a third trial, the long-term effectiveness of sodium butyrate (D4) to prevent disease outcome after an intestinal parasite (*Enteromyxum leei*) challenge was tested.

**Results:**

The results showed that opposed forces were driven by dietary plant ingredients and sodium butyrate supplementation in fish diet. On the one hand, vegetable diets induced high parasite infection levels that provoked drops in growth performance, decreased intestinal microbiota diversity, induced the dominance of the *Photobacterium* genus, as well as altered the gut mucosal proteome suggesting detrimental effects on intestinal function. On the other hand, butyrate addition slightly decreased cumulative mortality after bacterial challenge, avoided growth retardation in parasitized fish, increased intestinal microbiota diversity with a higher representation of butyrate-producing bacteria and reversed most vegetable diet-induced changes in the gut proteome.

**Conclusions:**

This integrative work gives insights on the pleiotropic effects of a dietary additive on the restoration of intestinal homeostasis and disease resilience, using a multifaceted approach.

**Electronic supplementary material:**

The online version of this article (10.1186/s40168-017-0390-3) contains supplementary material, which is available to authorized users.

## Background

Aquaculture is one of the fastest growing food production industries and plays a significant role in meeting global protein needs of humans. Indeed, it is estimated that about one billion people worldwide rely on fish as their primary source of animal protein, mainly in Africa and Asia [1], while the consumption of fish is continuously promoted for its multifaceted health benefits [2–4]. However, the use of marine resources (mainly fisheries) as the main protein and oil ingredients in aquafeed is no longer feasible due to the stagnation of the catches, and the increased demand for both human food and aquafeed. This scenario has forced the industry to explore alternative and more sustainable raw materials as aquafeed ingredients [5, 6] and plant ingredients have been used to replace marine feedstuffs at relatively high levels in many fish species [7–11]. However, low fish meal (FM) and low fish oil (FO) inclusion diets are often associated with poor growth and survival, enteritis or immune suppression and impaired quality [12–19]. Furthermore, the nutritional value of marine farmed fish can be compromised by a low content of ω-3 long-chain polyunsaturated fatty acids, when low FO inclusion levels are used [20–22]. Thus, a better understanding of the long-term physiological consequences of plant-based diets or other alternative feed ingredients is a major issue, and there is now an increasing interest for fish feed additives to prevent or repair adverse effects of extreme diet formulations. In addition, the need to find potential substitutes to antibiotics has led to the use of other functional feed additives including probiotics, prebiotics, synbiotics, immunostimulants, organic acids, nucleotides and medicinal herbs for boosting aquafeeds and safeguarding general health of aquatic animals. Some of these substances have been found to possess beneficial immunostimulant and stress relieving properties and their use increases the consumer confidence in farmed fish (reviewed in [23]).

The microbial community in the human gut exerts a major impact on host physiological, nutritional and immunological processes, which expands beyond the gastrointestinal tract to far distant organs [24]. Diet composition is among the main external factors that can affect the composition of the intestinal microbiota. Thus, high-protein diet, high-fat diet, prebiotics, probiotics and polyphenols can induce changes in some selected bacterial groups. In fact, there is evidence that 57% of the gut microbiota’s entire variation is due to dietary alterations [25, 26]. Alterations to the gut microbiota have been observed in numerous diseases, including human metabolic diseases such as obesity, type 2 diabetes, irritable bowel syndrome, Alzheimer’s disease [27] and allergic diseases [28]. However, few studies have validated causality and the underlying mechanisms remain to be elucidated [29].

A major metabolic role of the gut microbiota is the conversion of indigestible dietary carbohydrates (predominantly resistant starch and dietary fibre) into short-chain fatty acids (SCFAs), which mainly include acetate, propionate, and butyrate. These microbial metabolites are sensed by the host as a signal, and in the case of butyrate, the host responds by strengthening the epithelial barrier, reducing inflammation, and increasing the production of mucins and antimicrobial peptides [30]. Among the available strategies to stimulate butyrate production in the gut are diets with large amounts of dietary fibre, but this is generally not well tolerated and has a number of gastrointestinal side effects. A second option is the delivery of prebiotic substrates that are broken down by bacteria into smaller molecules, which are then used by butyrate producers. The third one is the direct administration of SCFAs, which has proved effective on the management of ulcerative colitis, Crohn’s disease, diarrhoea and obesity, brain function and behaviour in clinical trials [31].

The gut microbiota of fish is considerably understudied compared to that of humans and mammals and most studies have used culture dependant techniques, which are limited by the fact that many microbial species are not cultivable. The recent advent of next-generation sequencing (NGS) technologies has allowed for studying complex microbial ecosystems, and their use has led to a growing appreciation of the importance of the indigenous microbiota of fish [32, 33]. Such approaches have shown that the gut microbiota of aquatic animals is in general more fluidic than that of terrestrial vertebrates, is highly sensitive to dietary changes, and is modulated by life cycle variations, health status, farming conditions, and environmental and ecological factors (reviewed in [34, 35]).

In fish, the effects of butyrate on gut microbiota have been poorly explored [36, 37] and the underlying mechanisms of action of butyrate remain unclear and controversial. Dietary butyrate improved growth and feed utilisation in carp [37], yet no consistent effects have been reported in rainbow trout [38] or European sea bass [39, 40]. In gilthead sea bream (GSB), dietary butyrate resulted in a very slight improvement of growth rates in short trials [41], whereas no changes in growth performance were detected in longer trials [8]. However, dietary butyrate reversed in GSB many potentially detrimental effects of extreme vegetable diet formulations, including an intestinal inflammatory profile, imbalance of the genes involved in mucus production, changes in the epithelial junctions, and decreased intestinal transepithelial resistance [42]. Yet other gut features have to be explored with respect to butyrate supplementation.

The objective of the present study was to assess whether extreme vegetable diets induced changes in the intestinal mucus proteome, gut autochthonous microbiota and disease susceptibility, and whether these changes could be reversed by the addition of sodium butyrate to the diets. We first performed a preliminary short term trial (T1) in which small juveniles were fed for 10 weeks with the additive (0.8%) supplementing a basal diet with low vegetable inclusion (D1) and then challenged with a homologous pathogenic bacterium to detect effects on survival. This dose of additive produced a mild inflammatory reaction in the intestine and pronounced glycogen accumulation in liver [42]. Consequently, we performed a second trial (T2) where we fed fish with even more extreme low FM/FO diets (D2, D3) and tested the long-term effect of sodium butyrate (D4) at a lower dose (0.4%) on the intestinal proteome and microbiome. In a third trial (T3), the long-term effectiveness of sodium butyrate (D4) to prevent disease outcome after an intestinal parasite challenge was tested.

## Methods

### Trials performed and diet formulations investigated

**Fig. 1:**
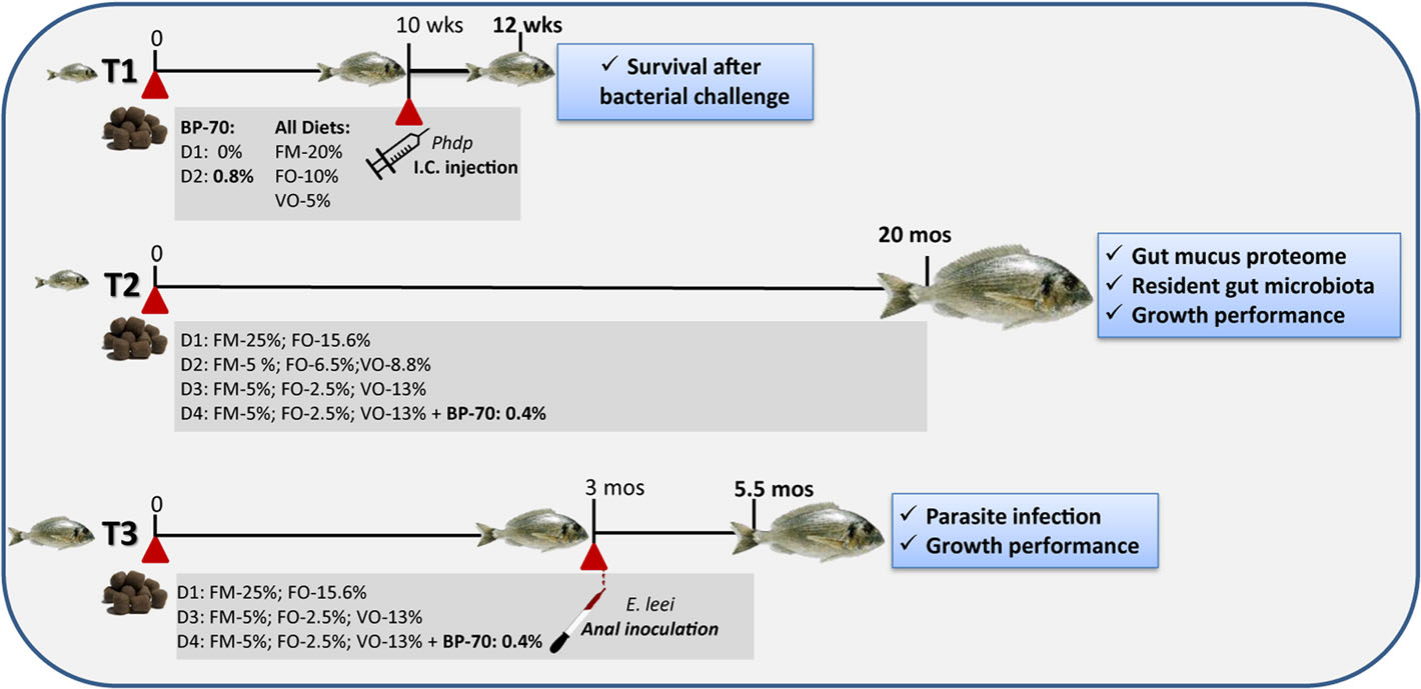
Diagrammatic summary of the different gilthead sea bream feeding trials (T) showing their timing, the main features of diet composition and the analyses performed at the end of each of them. T1 and T3 involved bacterial (*Photobacterium damselae* subsp. *piscicida*, *Phdp*) and parasite (*Enteromyxum leei*) challenges, respectively. BP-70 refers to the sodium butyrate used to supplement the diets. I.C. stands for intracoelomic

Three feeding trials were conducted and are summarised diagrammatically in Fig. 1. A preliminary short trial (T1) was conducted at the facilities of the Planta Piloto de Acuarios de Experimentación (PAE) del Servei Central de Suport a la Investigació Experimental (SCSIE) at the University of València (UV, Spain). Fish (*n* = 400) with an initial average size of 10 g were randomly distributed in two 600 L tanks, acclimated to the experimental conditions (water temperature = 21 ± 0.5 °C, water salinity = 30‰) and fed a basal diet (D1) for 3 weeks, and then fed ad libitum for 10 weeks with two different diets. D1 was a control diet corresponding to the 33VO (vegetable oil) diet in Benedito-Palos et al. [43], and D2 was D1 supplemented with 0.8% of a commercial sodium butyrate preparation (Gustor BP-70 ®Norel). This preparation is a partially protected sodium butyrate, with 70% sodium butyrate and 30% vegetable fat. This small amount of fat allows the active principle to be active along the entire gastrointestinal tract.

In a second long-term trial (T2) juvenile gilthead sea bream were acclimatised for 4 weeks to the indoor experimental facilities of the Institute of Aquaculture Torre de la Sal (IATS-CSIC, Spain) and fed with a standard diet (Efico YM 568 1.9 mm, BioMar). When fish reached a body weight of 15–16 g they were allocated among 12 2500 L-tanks, where each diet had triplicated tanks (150 fish/tank) and fed for 20 months four experimental diets formulated and produced by BioMar (Brande, Denmark), as described in [42]. FM was added at 25% in the control diet (D1) and at 5% in the other three diets (D2, D3, D4). Added oil was fish oil (FO) for D1 or a blend of VOs replacing 58% of the FO for D2 and 84% for D3 and D4. The BP-70 compound was added to D4 diet at 0.4%. All diets were isonitrogenous, isolipidic and isoenergetic and met all known nutritional requirements of GSB. For details of diet composition, see Benedito-Palos et al. [8]. The number of fish per tank was progressively reduced by periodical sampling, maintaining the rearing density below 15 kg/m^3^. Fish were fed to visual satiety once-twice per day, 3–6 days per week depending on season and fish size. Body weight was determined collectively every 3–6 weeks to calculate the specific growth rate (SGR) in each period. Oxygen content of outlet water remained higher than 75% saturation and day-length and water temperature followed natural changes at IATS latitude (40° 5′ N; 0° 10′ E). At month 20 (September 2014), animals were sampled for proteomic profiling of intestinal mucus (*n* = 6 fish per diet group, 3 per each of 2 replicated tanks; a total of 24 fish) and analysis of the biodiversity of intestinal microbiota (*n* = 8 fish per diet group, 4 per each of 2 replicated tanks; a total of 32 fish, pools were performed with fish originating from the same tank).

In a third feeding trial (T3), juvenile GSB (25–30 g) were fed with diets D1, D3 and D4 for 3 months in 500 L tanks under the same water and feeding conditions of T2 before the parasite challenge.

### Sampling procedures and ethics statement

At the end of the trials, one day-fastened fish were sacrificed by overexposure to the anaesthetics benzocaine (100 g/L, in T1) or 3-aminobenzoic acid ethyl ester (MS-222, 0.1 g/L, in T2 and T3) and tissue samples were obtained for microbiome, proteome and parasitological analyses.

Procedures in trial T1 were approved by the Ethics and Animal Welfare Committee of UV and carried out in registered installation facilities (code ES461900001203). Procedures of trials T2 and T3 were approved by the Ethics and Animal Welfare Committee of IATS. They were carried out in a registered installation facility (code ES120330001055) in accordance with the principles published in the European animal directive (2010/63/EU) and Spanish laws (Royal Decree RD53/2013) for the protection of animals used in scientific experiments. For lethal samplings, the suffering of animals was kept to a minimum.

### Intestinal microbiome

#### Sample collection

The intestinal mucus of sacrificed fish was sampled following the method described by Kim et al. [44]. Briefly, the intestine from the midgut region to the hindgut region was aseptically separated from the abdominal cavity and the contents were removed by mechanical force with forceps. These compartments have been reported to have the greatest microbial activity [34]. After rinsing the evacuated gut several times with sterile PBS, the mucus was scraped off with a sterile scalpel and collected in sterile 1.5 ml tubes. Four pools per dietary group were obtained (two pools per each replicated tank). Each pool consisted of the intestinal mucus from two different individuals, resulting in a total number of 16 samples for microbiome analysis. During the time lapse from mucus sampling and DNA extraction, less than 4 h, samples were held fresh in ice.

#### DNA extraction

DNA was extracted using the Master Pure Complete DNA and RNA Purification Kit (Epicentre Biotechnologies) following the manufacturer’s instructions and adding some modifications according to Carda-Diéguez et al. [45]. The DNA concentration and quality was determined by agarose gel electrophoresis (1% *w*/*v* agarose in Tris-acetate-EDTA (TAE) buffer) and Qubit dsDNA HS Assay Kit (Life Technologies). DNA was stored at −20 °C until used for PCR amplification.

#### PCR and pyrosequencing

The first 500 bp of the 16S rRNA gene, covering the V1 to V3 regions, were chosen for sequencing as they provide robust and informative taxonomies for studying microbial diversity [46]. 16S rRNA genes were amplified with the universal eubacterial primers 27F (5′-AGAGTTTGATCMTGGCTCAG-3′) and 533R (5′-TTACCGCGGCKGCTGGCACG-3′) with an annealing temperature of 52 °C and 20 cycles to minimise PCR biases [47]. A secondary amplification with equal conditions was performed using the purified PCR product as a template when the DNA concentration was insufficient. The 27F universal primer was modified to contain an 8 bp “tag sequence” specific to each sample, following McKenna et al. [48]. Barcodes were different in at least two nucleotides from each other to minimise mistakes in sample assignments. The Agilent High Technology Assay (Agilent 2100 Expert) was used to determine the amount and quality of DNA per sample.

PCR products were mixed in equal concentrations and purified using the PCR Clean-up DNA Purification Kit (Mo Bio). Samples were sequenced using the Genome Sequencer GS Junior Series (454 Life Science, Brandford, USA) at the Central Service Support Experimental Research (SCSIE) (University of Valencia, Spain).

#### Quality filtering and taxonomic assignment of sequence reads

Quality filtering of the raw sequences was performed using the Ribosomal Database Project (RDP) pipeline [46]. Sequences shorter than 200 bp, as well as those with an average quality score lower than 20 and sequences with more than one ambiguous base call were removed. Quality sequences were allocated to respective samples according to the barcode sequences at the beginning of each read. Chimeras were detected using UCHIME [49]. Taxonomic assignment of the sequences was made using the RDP classifier [46], implemented in the MG-RAST server database, with an 80% confidence threshold. Sequences were clustered into operational taxonomical units (OTUs) at 97% sequence identity.

#### Statistical analysis of the microbiome

Rarefaction curves were obtained by plotting the number of observed OTUs against the number of sequences. When the curves approximated saturation, the number of sequences was considered appropriate for further analyses. The Shannon diversity index was calculated as a measure of alpha-diversity. Beta-diversity among samples was calculated using by Fast Unifrac [50], and visualised using principal component analysis (PCA). Krona analysis was used to allow for a complete visual exploration of relative abundances within the complex hierarchies of microbial communities [51]. The shared and unique OTUs among the four dietary groups were also represented by a Venn diagram. All the above analyses were performed using the GPRO software [52] and R statistical software (v3.3.1).

The batch of sequences from this study has been deposited in the Sequence Read Archive of National Centre for Biotechnology Information under the project identification name “BIOPROJECT ID PRJNA381135” (from SAMN06670806 to SAMN06670821).

### Intestinal mucus proteomics

The proteome of the intestinal mucus of fish fed the four experimental diets in T2 was analysed by means of the quantitative iTRAQ technique. This analysis was carried out in two stages, (1) an initial analysis examining separately samples of anterior and posterior intestinal segments, using in total 8 pooled diet samples (each pool consisting of 6 individuals per each intestine segment and diet), and (2) a second analysis of only the anterior intestine, using individual samples from each diet (*n* = 6 for each diet, a total of 24 samples).

#### Mucus collection

Intestines of sacrificed fish were removed and unfolded on a sterile petri dish. The posterior end of the intestine, including the rectum plus three times the rectum length, was cut and locked at one end with hemostatic pliers. With a sterile syringe, 2 ml of mucus isolation buffer (PBS with 1% dithiothreitol, 1% sodium pyruvate, 0.6% HEPES and 0.03% amphotericin B) was introduced through the open end of the intestinal segment, after which the open end was immediately locked with hemostatic pliers. The same procedure was performed with an anterior intestine segment of equivalent length, placing the first pliers closely after the pyloric caeca. After 20 min incubation at 20 °C, isolation buffer was recovered by puncture with a syringe and individually disposed into ice-cold tubes. Each incubated intestinal tissue was opened lengthwise and the overlaying mucus layer softly recovered with a spatula avoiding epithelial scrapping. Mucus samples from anterior and posterior intestine were then centrifuged for 30 min at 13,500×*g* at 4 °C, and supernatants were transferred to cryotubes and kept at − 80 °C until further processing.

#### Protein digestion and iTRAQ labelling

Protein samples were precipitated with trichloroacetic acid, washed with cold acetone and air-dried. Pellets were dissolved with 40 μl of 8 M urea 0.5 M tetraethylammonium bicarbonate (TEAB) and quantified by Qubit (Invitrogen) according to manufacturer instructions. For each 100-μg sample, volume was adjusted up to 40 μl with 0.5 M TEAB. Samples were then reduced with 50 mM Tris-(2-carboxyethyl) phosphine at 37 °C for 180 min, alkylated with 100 mM methylmethanethiosulfonate for 10 min, and urea concentration was lowered to less than 2 M with 500 mM TEAB. Samples were then digested with 10 μg of sequencing grade trypsin in 0.5 mM TEAB overnight at 37 °C and dried in a speed vacuum. Trypsin digested samples were labelled for 3 h with 8-plex iTRAQ reagents with the signature ion signals 115 to 121 Da and an internal standard was included. Labelled samples were dissolved with 200 μl of 7 M urea, 2 M thiourea and 1.6% ampholites.

After iTRAQ labelling, peptides were separated by isolectrofocusing (IEF) on immobilised pH gradient (IPG) strips (13 cm, pH 3–11) with 5000 V to 25,000 Vh. After IEF, each IPG strip was cut into 11 equal pieces and peptides were extracted with 120 μl of acetonitrile (ACN) solutions at increasing concentrations (5%, 50%, 70%, 100%) plus 0.1% trifluoroacetic acid (TFA). After concentration by POROS R2 (Millipore), labelled peptides were speed vacuum-dried and adjusted to a concentration of 0.20 μg/μl in 2% ACN and 0.1% TFA.

#### Protein identification and quantification

iTRAQ tagged peptides from IEF fractions were analysed with the mass spectrometer nanoESI qQTOF (5600 TripleTOF, AB SCIEX). Samples were desalted out with 0.1% TFA at 3 μl/min for 5 min with a NanoLC Column (3 μ, C18CL, 15 cm × 75 μm), and then loaded onto an analytical LC column (3 μ, C18CL, 25 cm × 75 μm) equilibrated in 5% ACN and 0.1% formic acid (FA). Elution was achieved using a linear gradient of ACN (5% - 35%) in 0.1% FA for 90 min at a flow rate of 300 nl/min. Mass spectra were acquired in Information Dependent Acquisition mode by TOF MS scanning from 350 to 1250 *m*/*z* performed at spectral acquisition time of 0.25 s, followed by product ion scanning from 100 to 1500 *m*/*z* performed at 0.075 s on the 25 most intense charged ions. The MS data has been deposited to the ProteomeXchange Consortium via the PRIDE partner repository with the dataset identifier PXD006183.

Protein identification and relative quantification were performed with the ProteinPilot software (version 5.0) using the Paragon algorithm as the search engine. Each MS/MS spectrum was searched against the protein dataset expressed from CSIC Nutrigroup gilthead sea bream transcriptomic database (http://www.nutrigroup-iats.org/seabreamdb) [53]. Parameters considered for the search were trypsin as the digestion enzyme and MMTS as cysteine alkylation reagent. To minimise false positive results, the cut-off value of Unused Protein Score for protein identification was set at > 1.3, corresponding to a confident limit of 95%, and the score threshold was set at a 5% FDR. The resulting dataset was auto bias corrected to remove any variations due to unequal mixing during the combining of different labelled samples. Peptides for quantification were automatically selected by the ProteinPilot Pro Group algorithm. Functional analysis of identified proteins was performed by means of IPA software (www.ingenuity.com). For each protein in the analysis, the Uniprot accession equivalent for one of the three higher vertebrates model species in IPA (human, rat or mouse) was searched as previously reported for the transcriptome-encoding proteins of gilthead sea bream [53].

#### Data analysis and statistics

Data on protein expression was analysed using SPSS 21.0. Comparison of anterior and posterior intestine samples was performed by one-way analysis of variance (ANOVA) analysis followed by Benjamini–Hochberg multiple testing correction. Differences among experimental diets in anterior intestine individual samples were assessed by ANOVA followed by a Student–Newman–Keuls (SNK) post hoc test. A *P* value < 0.05 was considered statistically significant. Principal component analysis (PCA) and K-means cluster analysis of proteins with differential abundance were performed using Genesis software (v1.7.7). Heatmaps were constructed using R statistical software (v3.3.1).

### Pathogen challenges

#### Bacterial challenge

At the end of the feeding period of trial T1, 60 fish per dietary treatment (20 fish/tank, 3 replicates), which means a total of 6 tanks, were lightly anaesthetised with clove oil (30 ppm, Guinama, Spain), intracoelomically injected with 0.1 ml of bacterial suspensions in phosphate-buffered saline (PBS, pH 7.4) with 8.0 × 10^7^ colony forming units (CFU) ml^−1^ [54, 55]. A low lethal dose producing mortalities around 20% (LD_20_) was chosen for the bacterial challenge with *Photobacterium damselae* subsp. *piscicida* (*Phdp*) [56]. Bacterial suspensions consisted of *Phdp* strain SK 216/12, isolated from diseased European sea bass (*Dicentrarchus labrax*), cultured in tryptic soy agar (TSA, Pronadisa, Madrid, Spain) supplemented with NaCl at a final concentration of 1% (TSA-1) and sheep blood at a final concentration of 5% (TSAB-1), at 22 °C for 48 h.

A group of 10 fish per dietary treatment was injected with 0.1 ml PBS, as a control of the experimental handling. Fish were fed the same diets along the post-challenge period. After the challenge, the average water temperature was 21 ± 1 °C and fish mortality was monitored daily until no more mortalities were recorded for a minimum of two consecutive days. Post-mortem examination was performed by standard microbiological methods (pathogen culturing and isolation steps as described above) to confirm the presence of *Phdp*. Identification of the pathogen was carried out by means of an agglutination test with the corresponding antiserum. Cumulative mortality (CM) was calculated per dietary treatment using the following formula: CM = “number of dead fish” × “initial total number of fish”^−1^ × 100. Mortality percentages were subjected to an analysis of variance, using the SPSS 19.0 software (SPSS Inc.) to determine the differences among diets. A *P* value < 0.05 was considered statistically significant.

#### Parasite challenge

Recipient fish (*n* = 30) were tested by qPCR and histology to be free of *E. leei* infection before starting the experimental feeding. At the end of the feeding period of T3, fish were distributed in 12 200 L tanks (20 fish/tank, average weight 120 g). Four replicate tanks were allocated to each diet group. After 1 week of acclimatisation, two tanks of each diet were challenged with the intestinal myxozoan parasite *Enteromyxum leei* and the other two were not exposed to the parasite (control). Each diet group continued to be fed with the same experimental diets after parasite challenge. Parasite challenge was performed by anal intubation, as previously described [57]. Briefly, each recipient fish received 0.35 ml of an inoculum containing viable parasites from a single homogeneous batch prepared from donor infected fish. The inoculum was maintained cold and with frequent mixing to ensure similar viable parasite doses to each fish. Control fish were intubated with the same volume of PBS. A non-lethal (NL) PCR was conducted 5 weeks post intubation (p.i.) to verify the status of the infection in all recipient D1 fish and to decide the final sampling point. NL samples were obtained by probing the rectum with a swab [58] and PCR diagnosis was carried out as indicated in Sitjà-Bobadilla & Palenzuela [59]. Fish were weighted and sized before the challenge, at an intermediate time point (5 weeks p.i.) when checked for the infection status, and at the last sampling (10 weeks p.i.). Fulton’s condition factor (CF = (100 × body weight)/length^3^) and SGR (100 × ln (final body weight − initial body weight) / days)) were also calculated. Only two casualties were registered after the challenge in recipient groups: one in D3 and one in D4. All fish were sacrificed and intestinal samples taken 10 weeks p.i. for parasite diagnosis by histology and qPCR.

#### Parasite quantification by qPCR

After necropsy, entire intestines of 12 experimental fish per tank were removed and individually weighed. They were then placed in stomacher bags (Stomacher® 400, Seward), and the volume of each sample was adjusted by eye with variable volumes of PBS (4–8 ml), depending on the tissue weight/volume. Samples were homogenised, mesh-filtered and collected. Aliquots (500 μl) were taken from each sample for DNA extraction. They were centrifuged, and the pellets suspended in 200 μl of DNA lysis buffer. DNA extraction from these samples was performed using commercial silica-based spin columns kits. Parasite rDNA gene copies in the samples were quantified by qPCR [59]. Numbers were interpolated from the cycle thresholds (Ct) of the samples using a standard curve with known numbers of the target gene (6–7 orders of magnitude), run in the same plates on each assay. Only data from reactions with standard curves within an efficiency range (E = 0.85–1.1), and *R* ^2^ > 0.99 were accepted. Two dilutions of each DNA sample were run. Samples with Ct < 38 were considered positive whereas samples with 38 < Ct < 40 were flagged and repeated. In several cases, new dilutions and new DNA extractions from additional aliquots of the original homogenates had to be processed to reach a consensus on the status of weakly positive or inconsistent samples. Statistical differences between groups on prevalence, intensity and abundance data were analysed by Fisher’s exact test, one-way ANOVA or Kruskal–Wallis tests followed by Tukey’s or Dunn’s multiple comparison tests (significance was considered when *P* < 0.05).

#### Parasite evaluation by histology

After necropsy, anterior, middle and posterior intestinal segments of 5 fish from each tank (different from those used for qPCR) were fixed in 10% buffered formalin, dehydrated in a graded ethanol series, embedded in paraffin, 4-μm sectioned and stained with Giemsa. Parasitological evaluation was done by the observation of all the microscopic fields of the three intestinal segments by the same observer in a blind mode. The intensity of the infection by *E. leei* was semi-quantitatively evaluated in a scale ranging from 1 (very low) to 6 (very high), and the type of parasitic stages registered. The parasite was not detected in any control fish. The prevalence of infection and the mean intensity of infection for each of intestinal segment were calculated. The possible dependence between the prevalence of infection and the diet group for each intestinal segment was analysed by chi-square with Yates’-correction (significance was considered when *P* < 0.05).

## Results

### Diet induced changes on the intestinal microbiome

Microbial communities present in the intestinal mucus (autochthonous or resident microbiota) of fish fed different experimental diets in T2 were analysed. A total of 143,283 quality reads were obtained, ranging from 2985 to 11,906 reads per sample, and with an average of 8955. Reads were clustered and assigned to OTUs, and were predominantly classified to the phylum Proteobacteria. Since rarefaction curves approximated saturation (horizontal asymptote), a good coverage of the bacterial community was achieved and the number of sequences for analysis was considered appropriate (Additional file 1: Figure S1). Rarefaction analysis and the Shannon index was used to examine alpha-diversity and showed that microbiota from fish fed control diet (D1) was more diverse than fish fed experimental D2 and D3 diets, but less than those fed D4 diet. Thus, the diet supplemented with butyrate showed the highest diversity (Table 1).

**Table 1:** Relative abundance of the dominant genera and diversity of intestinal microbiota of gilthead sea bream fed control diet (D1) and test diets (D2, D3 and D4)

Fish group	Dominant genera (%)		Shannon index^a^
	*Photobacterium*	*Vibrio*	
D1	71	19	1.53
D2	69	19	1.36
D3	82	2	1.05
D4	44	7	2.27

^a^Shanon’s diversity index integrates both the number of genus and their relative abundance

**Fig. 2:**
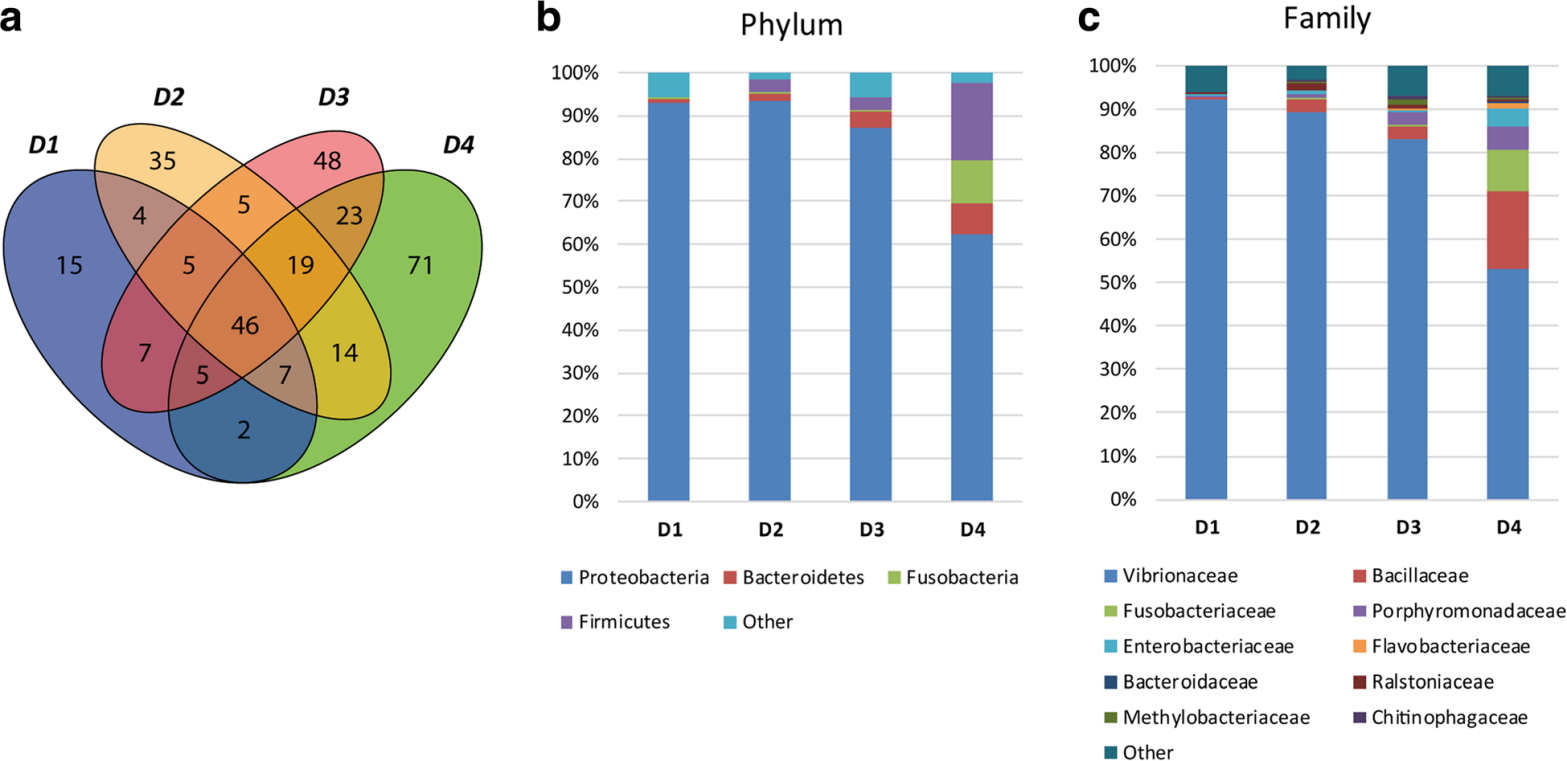
Intestinal mucus microbiome composition upon dietary challenges in T2. **a** Venn diagram depicting unique and shared OTUs among the different diets. **b** Stacked bar chart representing the relative abundance of bacterial phyla in the different dietary groups. **c** Stacked bar chart showing the relative abundance of the most abundant bacterial families in the different dietary groups

Forty-six OTUs (species/genus level) were shared among all samples, mainly consisting of members of the phylum Proteobacteria (60%), Bacteroidetes (17.4%) and Firmicutes (8.7%) (Fig. 2a and Additional file 2: Table S1). The phylum Proteobacteria dominated the intestinal microbiota in D1 (93%), D2 (93%) and D3 (87%) groups. However, this proportion decreased to 62% in fish fed D4 diet, while increasing the presence of Firmicutes, Fusobacteria and Bacteroidetes phyla (18, 10 and 7%, respectively) (Fig. 2b and Additional file 2: Table S1). *Vibrionaceae* was the dominant family in all diet groups, constituting 93.4, 89.5 and 83.4% in D1, D2 and D3, respectively, and was significantly lower in abundance in D4 (53.1%). Instead, *Bacillaceae*, *Fusobacteriaceae* and *Porphyromonadaceae* families were considerably more abundant in D4 (Fig. 2c).

Differences were also observed at genus and species taxonomic levels (Additional file 2: Table S1). The dominant genera in control samples were *Photobacterium* (71%) and *Vibrio* (19%) (Table 1). After extreme FM/FO replacement with plan ingredients (D3 diet), the percentage of *Vibrio* drastically diminished (2%) and the dominance of *Photobacterium* increased, reaching strikingly high values (81%) in the microbiota of fish fed D3 diet (Table 1). Butyrate supplementation in D4 fish induced a partial reversion to the control diet phenotype, with a decrease in *Photobacterium* and an increase in *Vibrio* genera (Table 1). Moreover, the presence of members of the genera *Bacillus*, *Fusobacterium* and *Tannerella* significantly increased in the intestinal mucus of this fish group. The proportion of *Photobacterium damselae* (*Phd*, formerly *Vibrio damsela*) significantly increased from 59% in the control fish to 69% and 81% in D2 and D3 groups, respectively, going back down to 44% in D4 fish. The proportion and diversity of *Vibrio* species was drastically reduced in D3 fish, and only partially recovered in D4 butyrate-supplemented fish compared to D1 and D2. The relative abundances of bacterial genera and species within each diet group are shown using Krona analyses (Additional file 3: Figure S2, Additional file 4: Figure S3, Additional file 5: Figure S4, Additional file 6: Figure S5).

**Fig. 3:**
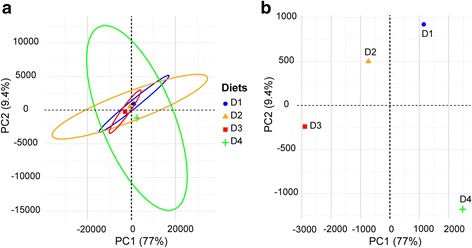
Principal component analysis (PCA) of the OTU composition from the intestinal mucus microbiome of fish under different dietary interventions in T2. For clarity, only the centroid of each dietary group is represented. **a** PCA showing the community centroid positions as well as the dispersion within each diet group (*n* = 4 per group, where each sample is a pool of two individual fish) using ellipses. **b** The same PCA is represented on a different scale to illustrate the differential position of each dietary group relative to each other

Principal component analysis (PCA) was used to visualise the relatedness of the samples depending on microbial community composition (Fig. 3a). The first two components, PC1 and PC2, explained 77 and 9.4% of the total variance, respectively. Variability of replicates within diet groups was shown using ellipses and clearly displayed a decrease in variability of microbial communities in response to the D3 diet. The largest variability was found in response to D4 diet. A closer look at the position of the multivariate centroids (Fig. 3b) revealed that the higher the FO substitution with VO (D2 < D3), the further the separation from D1. Interestingly, addition of butyrate in D4 induced a partial recovery of the control diet phenotype evidenced by the reversion in PC1 (77%).

### Diet induced changes in the proteomic profile of intestinal mucus

**Fig. 4:**
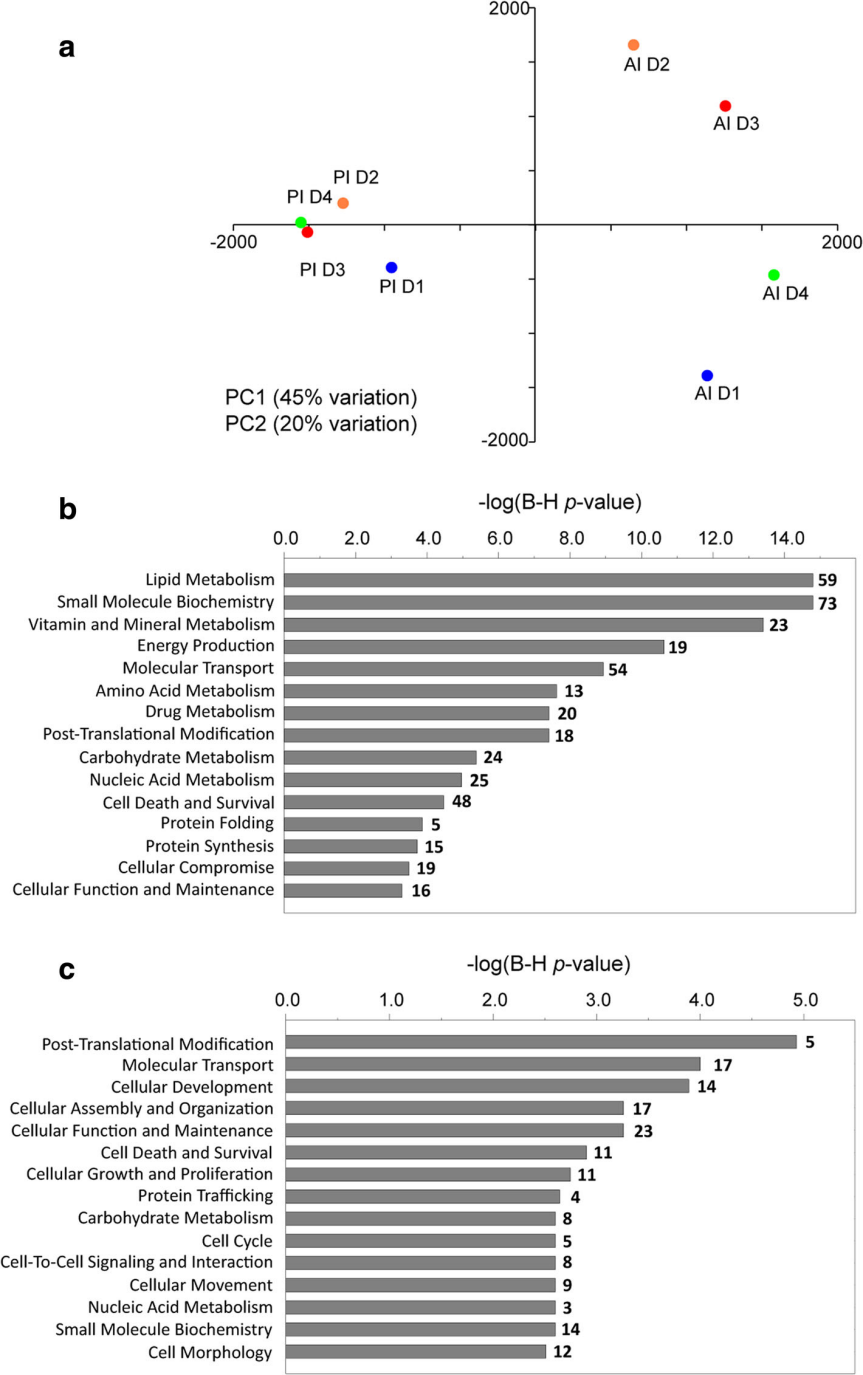
Spatial differences in gilthead sea bream gut mucus proteome in T2. **a** Principal component analysis of proteomic profiles of pooled samples from anterior (AI D1, D1 diet; AI D2, D2 diet; AI D3, D3 diet; AI4, D4 diet) and posterior (PI D1, D1 diet; PI D2, D2 diet; PI D3, D3 diet; PI D4, D4 diet) intestinal segments. Component 1 is represented along *X*-axis. Component 2 is represented along *Y*-axis. **b** Top significant molecular and cellular functions of proteins over-represented in anterior intestine. **c** Top significant molecular and cellular functions of proteins over-represented in posterior intestine. Significance in (**b**) and (**c**) is represented as a *P* value calculated using Benjamini-Hochberg multiple testing correction test. Numbers above bars represent the number of over-represented proteins for each function

An initial iTRAQ analysis was performed with eight pooled samples resulting from the combination of individual samples of each intestinal segment (anterior and posterior) and dietary treatment (D1–D4). In this first analysis, more than 2000 proteins were unequivocally identified in samples of intestinal mucus (Additional file 7: Table S2). The first two components of the PCA explained 65% of the total variance (45 and 20% for PC1 and PC2, respectively), and this analysis clearly separated the two intestinal segments along the first principal component (Fig. 4a). Comparison of anterior and posterior intestine samples revealed that 196 proteins were significantly more abundant in one intestine portion, with 139 proteins greater in abundance in the anterior intestine and 57 in the posterior intestine. Among them, 180 proteins (91.8%, 126 from anterior intestine and 54 from posterior intestine) were eligible for functional pathway analysis using the Ingenuity IPA software. In the anterior intestine, 101 over-abundant proteins participated in 26 relevant molecular functions. Among them, the most representative (Fig. 4b) were related to lipid metabolism (absorption of cholesterol and triacylglycerol), catabolism of amino acids and energy production (oxidation of fatty acids). In posterior intestine, 40 over-abundant proteins participated in 25 relevant molecular functions (Fig. 4c), with special relevance on post-translational modification (mainly protein deubiquitination) and several functions (cellular development, cellular assembly and organisation, cellular function and maintenance, cell death and survival, cellular growth and proliferation) related to cell proliferation. The molecular function of lipid metabolism was not extensively represented in the posterior intestine, with only seven proteins with higher abundance than in the anterior intestine, although it is interesting to note that two of them (fatty acid binding protein 6 and phospholipase A2 group 1B) had the highest abundance ratio between posterior and anterior intestines (33.33 and 5.26, respectively) (Additional file 7: Table S2).

The diet-mediated effects on intestine pooled samples were evidenced along the second principal component of the PCA (Fig. 4a), and were restricted to the anterior intestine. Thus, a second iTRAQ analysis was performed only on individual samples of the anterior intestine to better establish the effect of diets on intestinal mucus proteome. This second analysis detected 1045 different proteins with a confidence score higher than 95% (Additional file 8: Table S3). There were 125 proteins that were differentially abundant (*P* < 0.05) for at least one dietary group (Additional file 9: Table S4). Among these differentially detected proteins, K-means clustering supported four major protein patterns, according to the increase or decrease of abundance with D2 and/or D3 diets in comparison to D1, and the reversion or not to values closer to D1 in the D4 diet.

**Fig. 5:**
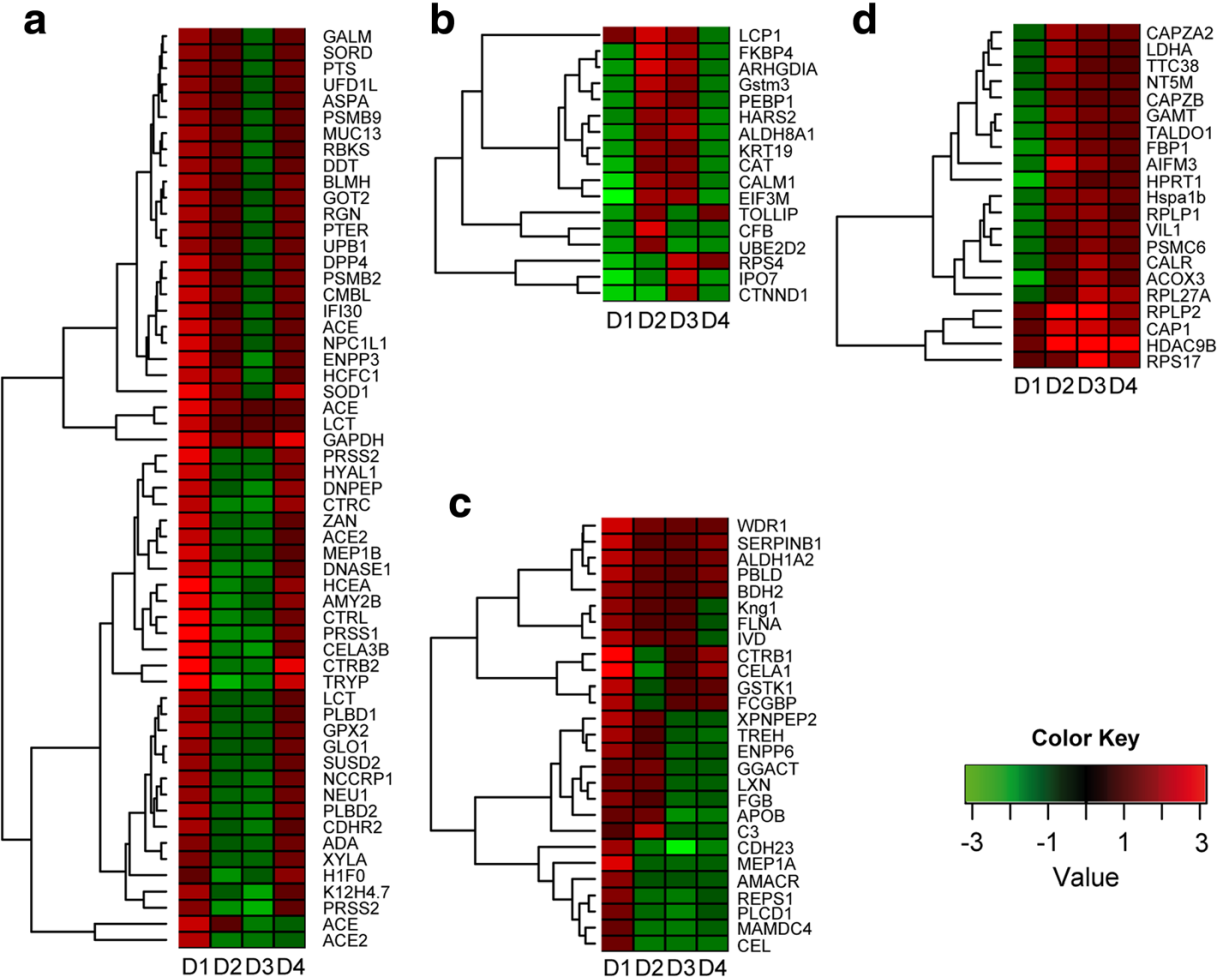
K-means clustering heatmaps of nutritionally regulated proteins from anterior intestine mucus in T2. **a** Cluster 1, with proteins downregulated by D2 and/or D3 diets and returning to control values in D4. **b** Cluster 2, with proteins upregulated by D2 and/or D3 diets and returning to control values in D4. **c** Cluster 3, with proteins downregulated by D2 and/or D3 diets and no restoration to control values in D4. **d** Cluster 4, with proteins upregulated by D2 and/or D3 diets and no restoration to control values in D4

Cluster 1 consisted of 57 proteins that were mainly downregulated by D2 and D3 diets, and most of them were restored to control values by D4 diet (Fig. 5a). This cluster was composed of many proteins involved in protein degradation with molecular functions related to digestion, immune response and cell growth and differentiation, such as angiotensin-converting enzyme (ACE), angiotensin-converting enzyme 2 (ACE2), bleomycin hydrolase, chymotrypsin B, chymotrypsin-like protease CTRL-1, dipeptidyl peptidase 4, gamma-interferon-inducible lysosomal thiol reductase, host cell factor 1, meprin A subunit beta, superoxide dismutase [Cu-Zn] and ubiquitin fusion degradation protein 1 homologue. Also remarkable was the presence of proteins involved in cholesterol metabolism (chymotrypsin-like elastase family member 3B, Niemann-Pick C1-like protein 1), and antioxidant defence (glutathione peroxidase 2). The epithelial mucin 13 (MUC13) was present in this cluster with downregulation only in D3 and return to D1 levels with butyrate supplementation (D4).

Proteins with a clear increase in D2 and/or D3 with a return to control values in D4 fish were represented in cluster 2, and constituted 17 proteins (Fig. 5b). These proteins were related to cell morphology and epithelial architecture (importin-7, phosphatidylethanolamine-binding protein 1, rho GDP-dissociation inhibitor 1, keratin 19, catenin delta 1 and plastin 2) and antioxidant defence (catalase and glutathione S transferase Mu 3).

Cluster 3 included 27 proteins that decreased in D2 and/or D3 without a clear reversion in D4 (Fig. 5c). This cluster consisted mostly of proteins related to lipid metabolism and digestion (such as alpha-methylacyl-CoA racemase, bile salt-activated lipase and apolipoprotein B-100-like), as well as cell junction processes, like apical endosomal glycoprotein and cadherin 23.

Cluster 4 comprised 21 upregulated proteins in D2 and/or D3 without a clear reversion by butyrate supplementation in D4 fish (Fig. 5d). Tissue repair was the most represented physiological process in this group with a high representation of molecular chaperones (calreticulin, heat shock cognate 70 kDa protein) and proteins involved in protein synthesis (60S acidic ribosomal protein P1, 60S acidic ribosomal protein P2). Proteins related to intracellular architecture were also represented in this cluster (villin 1, adenylyl cyclase associated protein 1, F-actin-capping protein subunits alpha 2 and beta).

**Fig. 6:**
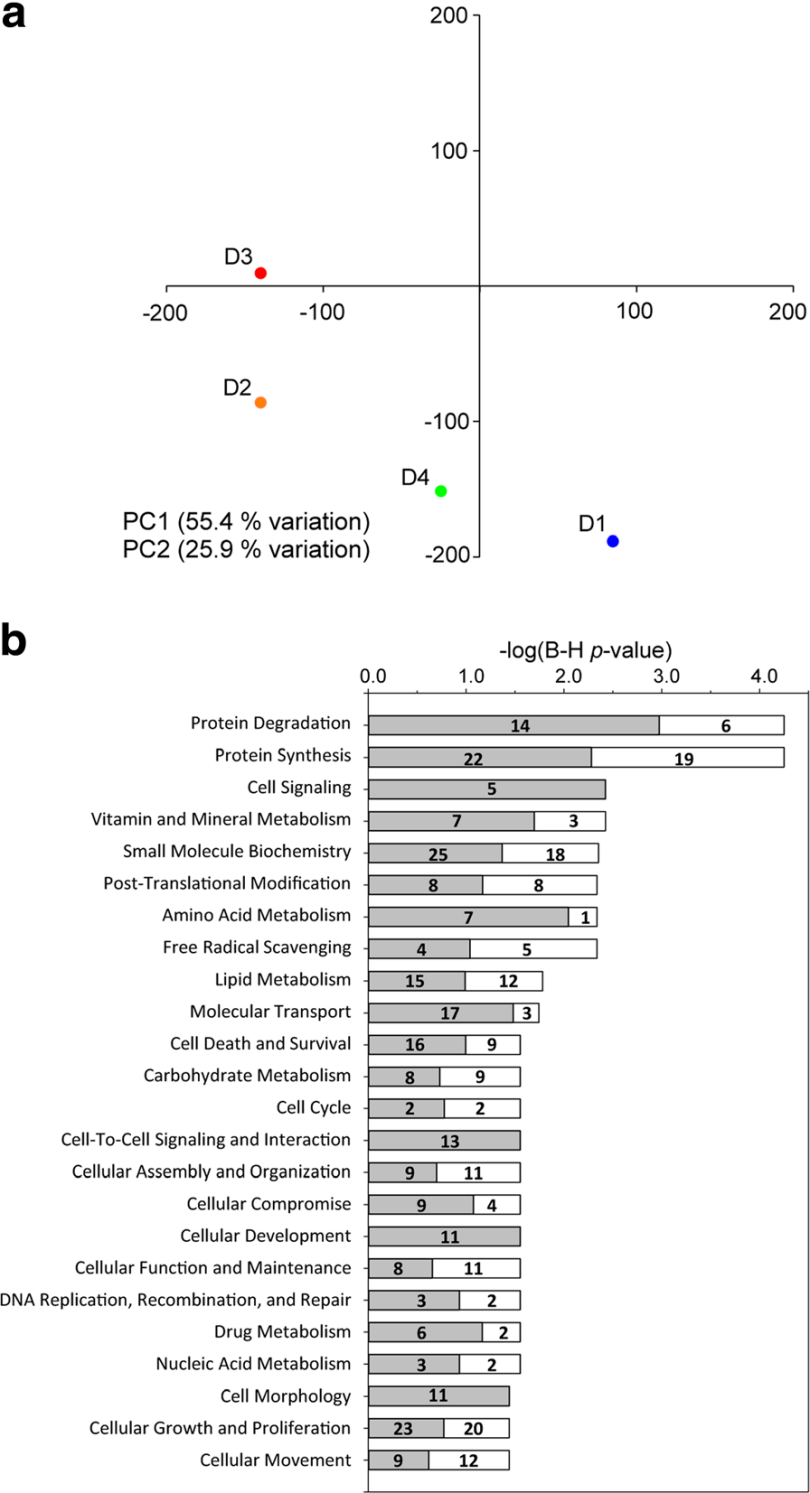
Butyrate effect on anterior intestine mucus proteome in T2. **a** Principal component analysis of nutritionally regulated proteins in anterior intestine mucus samples. Component 1 is represented along *X*-axis. Component 2 is represented along *Y*-axis. **b** Significant molecular and cellular functions (IPA) of nutritionally regulated proteins. Significance is represented as a *P* value calculated using Benjamini-Hochberg multiple testing correction test. Numbers in grey bars represent the number of proteins with a reversion to control values (clusters 1 and 2 in Fig. 5) in D4 group. Numbers in white bars represent the number of proteins with no reversion to control values (clusters 3 and 4) in D4 group

For proteins of clusters 1–4, PCA analysis showed that the two first components explained 81% of the total variance (Fig. 6a). PC1 separated the groups according to the inclusion level of FM (D1: 23%; D2, D3, D4: 3% FM), whereas the distribution of groups along PC2 was related to the different inclusion level of FO/VO (D1: 15.6% FO; D2: 6.5% FO, 8.8% VO; D3-D4: 2.5% FO, 13% VO). Accordingly, fish fed D2 and D3 diets moved along PC1 and PC2 when compared to D1 and only along the PC2 when compared to each other. Butyrate supplementation (D4) helped to restore the intestinal mucus proteome of fish fed the control diet evidenced by a reversion in both PC axes. When considering all the proteins as a whole, regardless of their reversion patterns, some molecular and cellular functions were highlighted as statistically significant (determined by Ingenuity pathway analysis (IPA), and corrected by Benjamini–Hochberg multiple testing correction) (Fig. 6b). Among the proteins with a reversion pattern to control values in the D4 diet group (clusters 1 and 2), processes related to protein degradation, cell signalling, vitamin and mineral metabolism, amino acid metabolism, molecular transport, and cellular development and morphology were highly represented.

### Diet induced effects on disease resilience

#### Diet effect on survival upon bacterial challenge

**Fig. 7:**
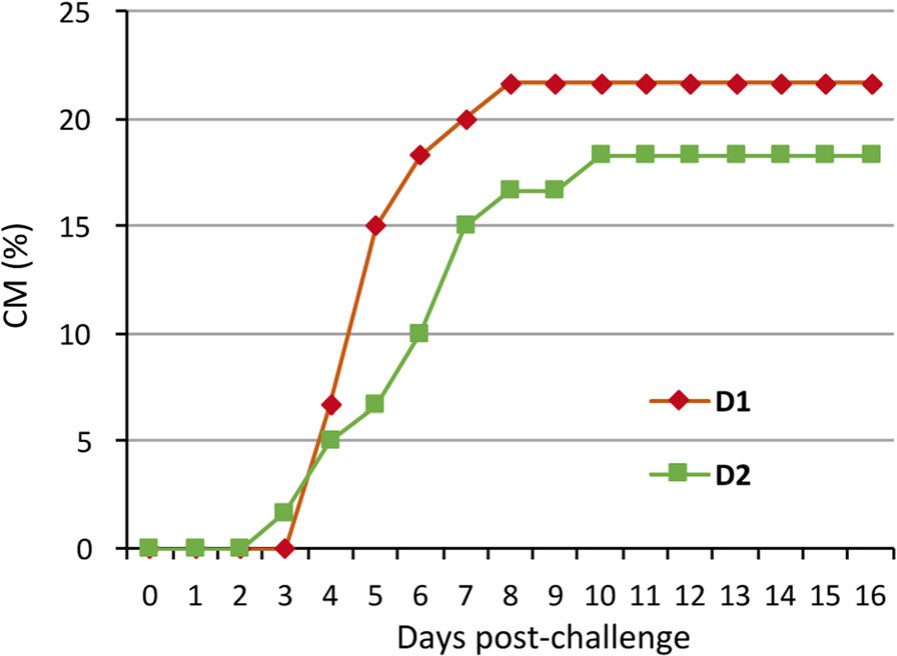
Effect of butyrate supplementation on survival against bacterial infection in T1. Cumulative mortality (CM) of gilthead sea bream fed with a vegetable substitution diet (D1) and the same diet supplemented with butyrate challenged with *Photobacterium damsela* subsp. *piscicida*

In trial T1, the average mortality rate of fish fed the butyrate supplemented diet (D2) after bacterial inoculation was lower (18.33%) than that of non-supplemented one (D1) (21.67%). However, this improvement was not statistically significant. Mortality started 3–4 days after bacterial inoculation and prolonged until day 10 post-injection. No mortality was observed in fish injected with PBS, regardless of the diet. The inoculated bacterium was always isolated from internal organs of moribund fish in pure culture (Fig. 7).

#### Diet effect on parasite infection and disease outcome

**Fig. 8:**
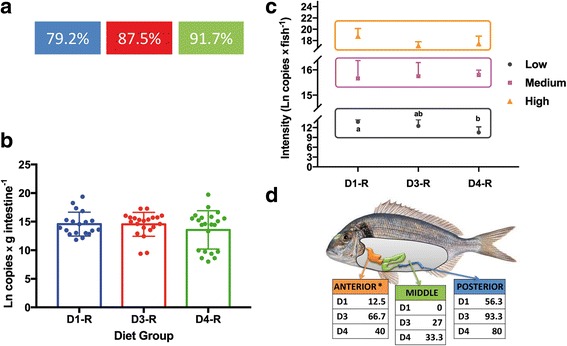
*Enteromyxum leei* infection levels of gilthead sea bream fed three different diets in T3. **a** Prevalence of infection (%) for each dietary challenged group (R) by qPCR diagnosis. **b** Intensity of infection measured by qPCR, the graph shows the mean and standard deviation of the logarithmic transformation of the number of DNA copies of the parasite per gram of fish intestine and individual scores for each dietary group. **c** Intensity of infection segmented in three categories with equal number of individuals. In the low tercile, different letters among diets indicate statistically significant differences in the mean (ANOVA *P* < 0.001) and the median (Kruskal-Wallis *P* = 0.014). **d** Prevalence of infection (%) per intestinal segment by histological diagnosis. No statistically significant differences were detected in any of the results except at the anterior intestine (*), in which a dependency was found between the diet and the number of infected fish, being D1 and D3 significantly different (Yates’ chi-square *P* = 0.0064)

In trial T3, there were no significant differences in the prevalence of infection by *E. leei*, though the values registered with the experimental vegetable diets (D3) were slightly higher (Fig. 8a). No significant differences between groups D1 (control) and D3/D4 (experimental diets) were found in the mean, mode or distribution of values related to the intensity of infection, i.e. DNA copies of parasite per gram of intestine (Fig. 8b) or per fish (data not shown). However, a wider range and a noticeable cluster of values on the lower end of the intensity distribution in D4 were observed (Fig. 8b). Thus, when the intensity of infection data was segmented into three groups with equal number of individuals, statistically significant differences were detected in the mean and the median between D4 and D1 groups within the lower infection level category (Fig. 8c). The histopathological study of the different intestinal segments showed a statistically significant influence of diet on the prevalence of infection at the anterior intestine (Fig. 8d). Few D1 fish were infected at the anterior intestinal segment, whereas D3 registered the highest prevalence and D4 an intermediate value. Only the difference between D1 and D3 was significant. None of the D1 fish were infected at the middle intestine, which is the last segment being infected during the chronology/progress of the infection (Fig. 8d). The number of fish with more than one infected intestinal segment was significantly higher in D3 (66.7%) than in D1 (12.5%), but did not differ statistically from D4 (40%).

**Fig. 9:**
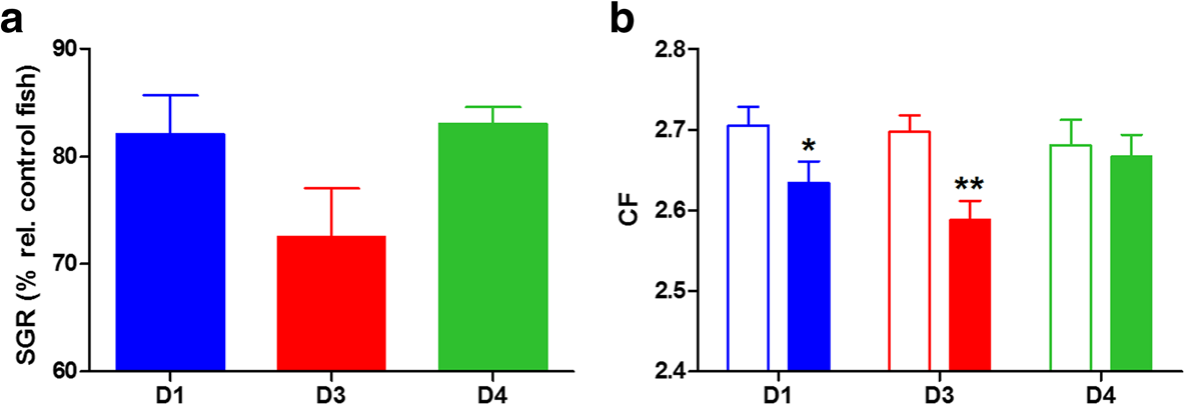
Effect of butyrate on growth parameters upon *Enteromyxum leei* infection in T3. **a** Specific growth rate (SGR) of challenged (R) fish represented as mean percentage (+ SEM) relative to each control group for the different dietary groups. **b** Condition factor (CF) represented as mean + SEM of each control (open bars) and recipient (solid bars) group for each dietary group. Asterisks represent significant differences between each pair of control and recipient groups (*P < 0.05, **P < 0.01)

Since clinical signs of enteromyxosis include anorexia, weight loss and cachexia, growth performance parameters were analysed throughout the trial. No statistical differences in weight were detected among replicates or diet groups before the challenge, nor at the intermediate sampling (5 weeks p.i.), but differences did appear at the end of the trial (10 weeks p.i.). Specific growth rate (SGR) decreased in all recipient groups with respect to their control group, and although no significant differences were detected, the greatest decrease was observed in D3, and there was little difference between D4 and D1 (Fig. 9a). The effect of the infection on growth performance was clearly dependent on the nutritional background, as the condition factor (CF) of fish exposed to the parasite decreased significantly due to the infection in both D1 and D3 recipient fish, whereas almost no impact was found in D4 recipient fish (Fig. 9b).

## Discussion

### Intestinal microbiota composition and effect of butyrate supplementation

The fish gastrointestinal (GI) microbiota is a relatively new field of research, and has only begun to be adequately defined with NGS tools in the past few years (reviewed in [32, 33, 60, 61]). The analysis of GI microbiota (mainly the transient one) in GSB, in wild and farmed individuals, has been addressed in few recent works using 16S 454 or Illumina sequencing platforms [62–64], PCR-denaturing gradient gel electrophoresis (PCR-DGGE) [65–67] or amplified ribosomal DNA restriction analysis and 16S rRNA gene sequencing of cultivable colonies [68]. However, scarce information on the diversity and role of prokaryotic populations adhering to intestine mucus in fish (autochthonous) is available, and specifically with respect to host health status. In this study, we focused on the characterisation of the autochthonous microbiota in the intestine of cultured GSB after a dietary intervention using pyrosequencing of the 16S rRNA gene.

The results revealed that the GSB gut microbiome is dominated by the phylum Proteobacteria, regardless of diet (constituting more than 60% relative abundance in all diet groups examined here), which is in accordance with what has been described in many teleosts [16, 45, 61, 69–72] and marine carnivorous fish [73]. The dominance of Proteobacteria in the resident gut microbiota of GSB was also reported by Kormas et al. [62]. Bacteroidetes and Firmicutes appeared in lesser percentages in our study (from 0.5 to 27.9%), as reported in other fish gut microbiota studies [61, 70]. By contrast, in other GSB studies, the phylum Firmicutes widely dominated the distal gut transient bacterial community (71%), regardless of the dietary treatment [64], or was well represented in the mixed gut microbiota (23.7%) [63]. In this latter work, a co-dominance of Actinobacteria (36.5%) and Proteobacteria (31.7%) was also observed. These differences show, once more, that not all datasets are comparable, due to the nature of the samples (intestinal content vs mucus) and the protocols used to obtain/store them [33, 45]. The transient microbiota in the intestinal content (allochthonous) of fish is known to differ from the autochthonous community [44, 45, 71] and this has been observed in studies on human gut microbiota [74]. Importantly, the transient microbiota may not accurately represent the complexity of the bacterial consortium living within the fish intestine, and appear more dependent on the interaction with the diet. Other possible explanations for the detected differences could be related to the experimental setup. While the previous works were performed using closed recirculation systems with lower water salinity (from 28 to 33‰), shorter feeding times (from 100 to 154 days) and constant water temperature (from 22 to 23 °C), our trial (T2) was much longer (20 months), with an open flow system and with natural temperature variations that mimicked fish farm rearing conditions.

Although fish GI microbiota composition is highly influenced by many factors [34, 71, 75, 76] that make very difficult to define a global microbiota profile at the host species level, some authors have tried to define a “core” gut microbiota, as already explored for humans [77]. Deep sequencing of microbiota from seven intestinal samples of zebrafish from three different locations suggested a “core” microbiome made of 21 OTUs [78]. This is not unexpected, since host factors also select which microbial species can survive. Rawls et al. [79] showed that transplantation of mouse intestinal microbes (dominated by Firmicutes and Bacteroidetes) into zebrafish, resulted in the outgrowth of the small number of Proteobacteria present in the mouse intestine becoming dominant in 2 weeks in the fish gut. The opposite occurred in mice colonised with zebrafish intestinal bacteria. The “core” gut microbiota of GSB under the study conditions was composed of 46 OTUs, but further NGS analyses of a variety wild and farmed GSB are needed to try to define a global core microbiota of this important commercial species.

In the current study, the composition of the autochthonous gut microbiota in terms of phylum, family and genus relative abundance as well as species richness was clearly affected by the dietary intervention. The extreme FM/FO replacement (D3 diet) induced a decrease in the intestinal bacterial diversity and a 22.5- and 4.3-fold increase of Firmicutes and Bacteriodetes phyla, respectively. This trend was further increased by butyrate addition (D4 diet) (139- and 8.5-fold in Firmicutes and Bacteroidetes, respectively) which was also responsible for a 23.2-fold increase of Fusobacteria with respect to control diet (D1). Thus, fish fed diet D4 with butyrate supplementation had the most diverse microbiota. In mice treated with sodium butyrate, Bacteroidetes decreased, whereas Firmicutes increased [80]. Other dietary interventions in fish, such as 6% hydrolysed wheat gluten inclusion in a low-fishmeal diet in Asian sea bass (*Lates calcarifer*) [81], or the use of vegetable proteins from pea and soy instead of FM in rainbow trout [82], also increased the abundance of Firmicutes in the gut microbiota. Prebiotic carbohydrate administration in Siberian sturgeon led to significant and beneficial shifts in gut-associated bacterial communities towards butyrate-producing/enhancing bacteria, including lactic acid bacteria and *Clostridium*, and therefore increased the concentration of SCFAs in the intestine [83]. In humans, Western-style diets, which are low in fibre, decrease beneficial Bacteroidetes and increase mucosa-associated Proteobacteria compared with a high fibre diet [84]. Thus, it seems we are facing a common trend across both aquatic and terrestrial gut systems, in which vegetarian diets or SCFAs increase the relative abundances of Bacteroidetes and Firmicutes, which comprise butyrate-producing bacterial groups such as Clostridial cluster XIV and IV and other with probiotic potential as *Bacillus cereus*. Therefore, GSB, a carnivorous fish, seems to adapt part of its autochthonous bacteria to the diet by increasing the abundance of OTUs typical of vegetarian fish [73].

Fusobacteria significantly increased in fish fed with the D4 diet. This group has been recognised to produce butyric acid as a major product of fermentation [85], and some species have been associated with diseases in mammals [86]. Fusobacteria are the most abundant phylum of the autochthonous normal gut microbiota in common carp [37], and are also well represented in the gut microbiota of some lab reared zebrafish [78] and tropical siluriform fish [87]. Other authors have reported the presence of *Fusobacterium* spp. in the gut microbiota of three commercial warm-water fish species [88]. *F. mortiferum*, which represented the 3% of gut microbiota of fish fed D4 diet, was the second most commonly identified genus in the fish species *Lepomis macrochirus* in this latter study [88]. These bacteria probably provide beneficial functions for the host by producing butyrate from complex polysaccharides.

Within Proteobacteria, VO-based diets favoured the dominance of *Photobacterium* species (mainly *P. damselae*) over *Vibrio* indicating that fatty acids also play a role in such changes. Butyrate supplementation reversed the decrease of *Vibrio* only to some extent, following a restoration trend also observed in the intestinal mucus proteome. Similarly, diets with partial substitution of FM with seaweed ingredients induced a significant reduction of *Vibrio* species in the intestines of GSB juveniles [66]. Total replacement of FM by vegetable ingredients in GSB diets produced an increase of the genus *Photobacterium* in the mixed gut microbiota [63]. *Photobacterium damselae* strains are high producers of exoenzymes, helpful for plant diet assimilation [89, 90] which explains their prevalence in VO diets. By contrast, the most prevalent phylum in resident gut microbiota of GSB from wild populations, or fed conventional or organic diets, were β-Proteobacteria [62] (this study) and not γ-Proteobacteria. This dominance of γ-Proteobacteria has been related to vegetable diets [82]. The dietary regime is likely the best explanation of these differences, but methodological differences need also consideration, e.g. DNA extraction was performed from frozen gut tissue at − 80 °C and this could have affected the bacterial community structure [33, 45]. Moreover, specific bacterial consortia can be associated with the distinct selective pressures imposed within the gut habitat of each host and with the diet, but also with the time of exposure to the dietary challenge, as observed in GSB fed probiotics [65].

In humans, reduced faecal microbial diversity is characteristic of diseases such as obesity, type 2 diabetes and irritable bowel disease [91]. Similarly, the bacterial diversity in the intestine of diseased fish was markedly lower than in healthy fish [92]. In agreement with our current results, decreased diversity in gut microbiome has also been observed in other fish species with different dietary interventions, such as the combination of probiotic and prebiotic in sole (*Solea senegalensis*) [93], the replacement of FO by VO in sablefish (*Anoplopoma fimbria*) [16], or soya inclusion in salmon [94]. In GSB, the number of OTUs decreased from wild to conventionally reared fish, involving a response of the gut prokaryotic community to the supplied food as well as possible alterations in food assimilation [62]. However, the diversity indexes of mixed gut microbiota were not significantly affected by FM and FO replacement in European sea bass (*Dicentrarchus labrax*) [17] or FM replacement in GSB [63, 64]. In agreement with the current study, the addition of butyrate also increased the level of biodiversity in transient microbiota of European sea bass [95] and mouse [80]. On the contrary, sodium butyrate did not affect significantly the gut microbial communities of common carp [37]. The partial restoration of the bacterial gut composition to D1 profile and the increase in diversity in GSB fed butyrate supplemented diet (D4), could be considered as reversing signs of the excessive growth of normal components of GSB gut microbiota found in D3, such as *Photobacterium damsela*, which can act as secondary pathogens of marine animals [96].

Another effect of the vegetable diets was a decrease in variability, which was also reversed by butyrate addition. In fact, in humans, the constellations of microbes that make up an individual’s microbiome are unique, with only up to 30% conservation of strains shared among unrelated individuals [97]. In European sea bass, the high variability of the bacterial profiles among groups reared under the same conditions was attributed to differences in genotypes [71]. In GSB, other authors found the variability among fish within the same group in the mixed gut microbiota was higher in fish fed an extreme vegetable diet than in those fed a standard one [63].

### Diet effect on intestinal mucus proteome

The iTRAQ methodology is a gel-free approach that allows the simultaneous comparison of multiple proteomes, each labelled with specific isobaric mass tags, and the ratio of the intensities between the different reporter ions can be used to measure increases or decreases in the amount of the corresponding peptides [98]. This technique yields highly reliable quantitative results for the same protein in different samples, though it cannot quantify in absolute levels or discriminate post-translational modifications, and is highly dependent on homologous protein databases to match and quantify the obtained MS spectra. In the present trial, this yielded a high number of intestinal mucus proteins (2217 in pooled samples) that was reduced to 1045 on individual samples, since proteins not present in all the individual analysed samples were not taken into account in the analysis. Recently, we have also identified more than 2000 proteins in the skin mucus of GSB using 1-DE/MS approaches [99], when the digested protein fragments were matched against our protein database with a high coverage of GSB protein-codifying sequences (more than 15,000 unique sequences in Swissprot). These numbers are in the same order of magnitude or even higher than those reported for other mucosal tissues and body fluids in humans and other animal models [100–102], and in skin mucus in GSB [103, 104]. The recent use of the iTRAQ technique to characterise the proteins in bile and intestinal mucus of Nile tilapia [105] was also able to discriminate more than 2700 peptide fragments, but only 319 (corresponding to 179 different proteins) were properly identified. Whether this relatively low number reflects the limitations of the use of a non-homologous protein database in iTRAQ approaches remains unclear.

Our wide-proteome analysis shows the changes in the composition of anterior-posterior mucus and clearly evidences the functional specialisation along the intestinal tract of teleost fish. These results are in line with the data from both zebrafish [106] and Mediterranean farmed fish [42, 107] that show a high spatial specialisation of intestine at the transcriptional and functional level. Indeed, when comparing iTRAQ profiling of intestinal mucus proteome of GSB with the microarray gene expression profiling of European sea bass intestine [107], a high coincidence for the top molecular and cellular functions was found in both anterior and posterior intestinal segments (11 out of 15 for anterior and 10 out 15 for posterior).

We focused on the anterior intestine when examining dietary effects upon the intestinal mucus proteome of GSB because of the results of the first iTRAQ analysis (anterior and posterior pooled samples from each dietary condition). A relatively low number of proteins responded to the diets in the anterior intestine mucus (121 proteins, 11.5% of the identified proteins). Since all diets used in this study supported fast growth and feed conversion efficiency from early life stages to completion of sexual maturation in four-year old fish [108], the reduced impact of vegetable diet formulations on intestinal mucus composition can be viewed as an indirect evidence of preserved intestinal health. This also applies to previous wide-transcriptomic studies of intestine in several farmed fish, including GSB [109] and Atlantic salmon [110]. However, when a targeted transcriptomic approach was applied with selected markers of cell differentiation and proliferation, intestinal architecture and permeability, enterocyte mass and epithelial damage, immune-surveillance and pattern recognition receptors, the number of differentially expressed genes in the anterior intestine of GSB highly reflected the level of FM and FO replacement [42]. Moreover, in this previous study, dietary butyrate supplementation was able to reverse most of these transcriptionally mediated effects, and even the changes in the intestinal permeability assessed by electrophysiology. In the same way, in the present study, PCA analysis of anterior intestine mucus proteome clearly showed that the addition of butyrate in D4 could revert nutritionally regulated proteins to levels much closer to control diet (D1). This restoration effect is in agreement with the results on the intestinal microbiome.

The reversion process in the group fed the D4 diet was observed in more than 60% of the nutritionally regulated proteins (clusters 1 and 2, 74 proteins), with a high representation of proteins involved in digestion, transport, cell signalling and cellular morphology. In addition to this, the presence of mucin MUC13 in cluster 1 is remarkable, as mucins represent the most abundant components of the intestinal mucus, and are responsible for the mucus structure and the protection of intestinal epithelial surface and membrane proteins from luminal digestive enzymes and pathogens [111–113]. Interestingly, two other mucins, mucin 2 (MUC2) and mucin 2-like (MUC2-L), were also present among the identified proteins in anterior intestine (Additional file 8: Table S3) and their response to diets was very close (although not enough to be statistically significant) to that of MUC13, including the reversion in D4 group.

Previous characterisation of the main mucins expressed in different GSB tissues [114] indicated the presence of MUC13, MUC2, MUC2-L and mucin 18 (MUC18) in the anterior intestine, but gene expression of MUC18 was 30- to 50-fold lower than that of the other mucins. Thus, the lack of detection of MUC18 at the protein level here is not unexpected. It is also of importance that one of the core proteins associated with mucins, the igGFc-binding protein (FCGBP), that is usually bound together with MUC2 in mucus layers [115], showed a similar pattern of change with the diets, although K-means clustering placed it in cluster 3.

Diet effects on the abundance of mucins and accompanying proteins can have an impact on intestinal function and integrity. MUC13 plays an important modulatory role in epithelial response to damage and infection [116, 117]. Deficiency of MUC2 leads to colonic inflammation [118], and MUC2 expression has been proven to be stimulated by short chain fatty acids like butyrate in intestinal epithelial and myofibroblast cell lines of humans origin [119]. In this regard, downregulation of MUC13 and MUC2 in GSB fed D2 and D3 diets could result in potential inflammation and pathological problems that can be alleviated by butyrate supplementation.

A number of proteins involved in protein catabolism (chymotrypsins, trypsins, bleomycin hydrolase, lactase-phlorizin hydrolase, proteasome subunits) were less abundant in mucus samples of fish fed D2 and D3 diets and recovered the control values in the D4 group (cluster 1). Proteases are heavily present in the gastrointestinal tract, where they exert digestive functions [120]. ACE and ACE2 proteases were also represented in cluster 1. ACE is involved in the modulation of intestinal epithelial cells apoptosis and proliferation [121], while ACE2, involved in intestinal inflammation and innate immunity, is a key regulator of dietary amino acid homeostasis, an important task for regenerative responses and repair mechanisms [122–124].

Other proteins that initially were not filtered as nutritionally regulated could be of functional relevance. This is the case of several molecular chaperones and heat shock proteins that were detected in the intestinal mucus (78 kDa glucose-regulated protein, 90 kDa heat shock protein beta, heat shock 70 kDa protein 4, heat shock protein 105 kDa) that could be considered as representative proteins of cluster 4 (up-regulation in D2 and D3 diets) in addition to heat shock cognate 70 kDa protein. In vivo and in vitro studies have concluded that several nutritional components can affect heat shock proteins expression in the gut (as reviewed in Liu et al. [125]), although it must be noted that this increase is also a usual symptom of gut inflammation diseases [126].

Altogether, the use of extreme vegetable diets induced changes in intestinal mucus proteome that could lead to an increased susceptibility to pathogens and a partial loss of intestinal functions. This was especially evident with the downregulation of mucins that affect the composition of the mucus layer and protect the epithelium, and with the downregulation of proteins related to digestion. Importantly, most of these changes were partially reversed with butyrate addition in D4 diet, suggesting butyrate/SCFA supplementation as a means to improve the use of vegetable diets.

### Ability of butyrate supplementation to alleviate disease signs

SCFAs, primarily butyrate, not only play a role in energy homeostasis [127], but also possess antioxidative, anticarcinogenic and anti-inflammatory properties that play an essential role in maintaining gastrointestinal and immune homeostasis in humans and different animal models [128–131]. However, whether SCFAs impact antimicrobial host defences remains largely unknown. Previous studies with human pathogens [132, 133] and animal (mammals and birds) models [80, 134–137] have shown the capability of different dietary butyrate formulations to reduce some bacterial and parasitic infections or their impact on the host. However, the impact of butyrate on fish health is poorly documented and studies mainly focus on improvement of immune factors/genes [37, 138–140]. Studies on the effect of SCFAs against fish pathogens are very scarce [141, 142].

In the current study, we chose two different types of pathogens to decipher whether butyrate could affect host susceptibility. The Gram-negative bacteria *Photobacterium damselae* subsp. *piscicida* was selected because it is the causative agent of photobacteriosis, an important disease affecting, among others, GSB juveniles [56]. Our results show that butyrate supplementation helps to enhance the survival rate against this pathogen. Previous studies on rainbow trout fed β-hydroxy-β-methylbutyrate for 8 weeks [141] or poly-β-hydroxybutyrate for 6 weeks [142] also showed improved survival against *Aeromonas salmonicida* and *Yersinia ruckeri* infections. The decrease in mortality found in our study was lower than those reported for rainbow trout, which can be due to interspecies differences, different dietary formulations or pathogen aetiology. However, even a modest improvement in survival (16%) can result in a significant economic impact at the animal production industry scale. The reduced mortality could be mediated by the direct bactericidal effect of sodium butyrate, by the indirect effect of lower intestinal pH which favours the growth of beneficial bacteria, or by improving host immune functions, as described in broilers [143].

*Enteromyxum leei*, an enteric myxozoan parasite (microscopic metazoan relative of Cnidaria), was selected because it progressively invades the paracellular space of the intestinal epithelium, producing changes at local and systemic levels [144–146]. It causes important economic losses in Mediterranean sparid farms [147]. Earlier studies had shown that vegetable oil substitution can worsen the disease outcome of GSB, when challenged with *E. leei* by effluent transmission [148]. To elucidate this further and to see if this worsening effect could be alleviated, a diet with high substitution of FM and FO without (D3) or with supplementation of sodium butyrate (D4) was tested against a control (D1) using a highly effective, direct infective route, the anal intubation. The results confirmed that indeed D3 and D4 groups reached a higher prevalence of infection than D1, though differences were not statistically significant. Interestingly, however, D4 recipient fish showed indicators of a somehow reduced infection degree. These included a less extensive dispersion of parasites along the intestinal tract (significant lower prevalence of infection at the anterior intestine than D3 recipient fish), and the presence of a cluster of low-intensity infected fish in this group. Furthermore, D4 recipient fish did not show the typical disease signs of *E. leei*-infected fish, in which anorexia and intestinal damage lead to impaired growth, cachexia and finally death [59]. Instead, recipient D4 group did not show decreased condition factor as seen in recipient D1 and D3, or decreased SGR detected in recipient D3.

Therefore, the main outcome of butyrate supplementation in D4 recipient fish was that health, in terms of growth, was not altered, and no obvious disease signs were observed. Thus, the parasitosis occurred as a “subclinical infection”. While a lower infection prevalence at the anterior intestine of D4 compared to D3 fish could partly explain these results (this segment has a major role of nutrient absorption which could be impaired by the parasites), this does not seem to be the case in this experiment because the prevalence of infection at the anterior intestine in D1 fish was the lowest and this group did indeed show a significant decrease of CF. A more likely explanation relates to a more efficient repair and improvement of the intestinal integrity and function, which was demonstrated in previous complementary studies using butyrate-supplemented diets [42]. Indeed, the D4 diet restored the expression of genes related to epithelial permeability and structure (tight-junction and adherence-junction proteins) and mucus production (which were altered in fish fed D3 diet), and also restored the transepithelial electric resistance damaged by D3 [42]. Similarly, in vitro supplementation of porcine small intestinal epithelial cells with sodium butyrate significantly enhanced mRNA expression of intestinal mucosal tight junction proteins, which suggests that the promotion of wound healing by butyrate is related to the maintenance of the function of the intestinal barrier [149]. In addition, butyrate-treated mice ameliorated histological colitis [80]. Overall, the mechanisms behind this alleviation are complex and multifaceted and remain to be defined.

Another intervening factor could be the restoration of the intestinal pro-anti-inflammatory balance, as shown by the transcriptomic profile of immune related genes of fish fed D1, D3 and D4 diets, and the inflammatory histological signs of the intestine [42] and muscle [8]. Extreme plant diets are known to produce inflammatory effects in fish, particularly soybean meal in Atlantic salmon [19, 150] and other species [40]. However, in the present trial no such effects were shown for diet D2 [8, 42] indicating that the use of high quality, concentrated plant protein sources like soy protein concentrate, corn gluten and wheat gluten can successfully replace FM in GSB diets even at extreme levels. This can be linked to the lower levels of anti-nutrients and non-starch polysaccharides in these raw materials compared to their respective meals [82]. On the other hand, diets D3 and D4 also had a much lower FO content compared to D1 and D2 and consequently they contained lower dietary levels of essential fatty acids EPA and DHA. The latter are known to affect fish health status and immune responses [151] and could explain, at least partially, the effects shown in this trial. Butyrate (0.2%), had some mitigating effect on the intestinal inflammation signs induced by soybean meal in European sea bass, though no significant changes were observed in the expression of several cytokines [40]. The up-regulation of inflammatory markers in soybean meal-induced enteropathy [150] was also reversed by adding bacterial cell wall fractions [152] or a lyophilized live lactic acid bacterium [153] to the diet. However, little is known on the effects of butyrate on low FO diets and the potential interactions with essential fatty acids as shown in this study. In the current study, such changes were mainly observed at the anterior intestine of GSB, which was more responsive to the dietary treatments. This antero-posterior decreasing gradient could be explained, at least in part, by the fact that the delivery of the highest dose of butyrate, even being partially protected, is supposed to occur preferentially at the anterior intestine segment [42].

In the present study, the concomitant changes detected in the microbiome and the mucus proteome of D4 intestines could also help to explain the better disease outcome of these fish. In particular, research in the last decade in humans and other animal models has convincingly demonstrated that the microbiota is crucial in order to prime and orchestrate innate and adaptive immune responses and influence barrier function as well as multiple developmental and metabolic parameters of the host. Reciprocally, host reactions and immune responses instruct the composition of the microbiota [154]. Changes in the balance between commensal and pathogenic bacteria could result in a different impact of bacteria at the epithelial level (the target site of *E. leei*) or even affect the development of the parasite. This has already been demonstrated for an insect-parasite model: a *Trypanosoma cruzi* clone changes the microbiota population in the digestive tract by modulating the host immune responses and this contributes to parasite development in the gut of *Rhodnius prolixus* [155]. Likewise, human intestinal apicomplexan parasites have a remodelling effect on the gut microbiota profile [156]. Although rapid advances in our understanding of host-intestinal bacteria interactions have been achieved in fish [157, 158], the inter-relationship of intestinal microparasites and gut microbiota remains largely unexplored and future efforts should be made to address this gap. Further studies in this fish-parasite model will elucidate how much the parasite infection alters the gut microbiota and the mucosal proteome.

## Conclusions

**Fig. 10:**
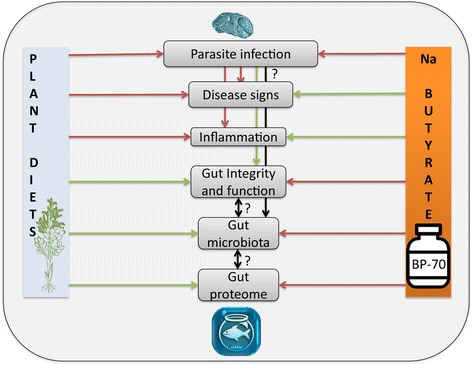
Integrative diagram showing the main results obtained in the current and previous works [42] when feeding gilthead sea bream with extreme vegetable diets or supplementing them with sodium butyrate (BP-70 ®Norel). Red arrows indicate increasing effect and green ones decreasing effect. Black arrows indicate possible relationship to be further explored in future studies

Figure 10 summarises and integrates the current results with those previously obtained by us using a fish-parasite model. Opposed forces are driven by dietary plant ingredients and sodium butyrate supplementation in GSB diet. On the one hand, vegetable diets induced high parasite infection levels that provoked drops in growth performance, inflammation and loss of gut integrity and function, reduction in intestinal microbiota diversity and dominance of *Photobacterium* genus, as well as changes of gut mucosal proteome with potential detrimental effects on intestinal function. On the other hand, butyrate addition did not prevent the infection, but avoided growth retardation and decreased inflammation in challenged fish, as well as restored gut integrity and function, increased intestinal microbiota diversity with a higher representation of butyrate-producing bacteria and reversed most changes in the gut proteome.

This multifaceted integrative study provides insights on the pleiotropic effects of a dietary additive on the maintenance of intestinal health and disease resilience. The notion that different dietary components can modulate the microbiota has started to be used therapeutically in humans [159] and is becoming a major avenue of research in aquaculture. However, information and understanding regarding fish gut microbiota still lags behind that of human and other mammals, including evidence for cause-effect relationships between gut microbiota and host physiology. In any case, this study is an important step towards establishing GSB as a powerful model for the development of sustainable and healthy fish feeds.

## Additional files

Additional file 1: Figure S1. Rarefaction curves obtained from the sequencing data of the four different pooled samples per dietary group. (TIFF 437 kb)

Additional file 2: Table S1. Percentage of the different bacterial OTUs identified in intestinal mucus of fish fed different diets. The results can be sorted by Phylum, Family, Genus and Species (OTUs). Numbers represent the mean percentage of four replicate pools. (XLSX 63 kb)

Additional file 3: Figure S2. Krona visualisation of the relative abundance of intestinal bacterial OTUs identified in fish fed D1. (HTML 238 kb)

Additional file 4: Figure S3. Krona analysis of the relative abundance of intestinal bacterial OTUs identified in fish fed D2. (HTML 243 kb)

Additional file 5: Figure S4. Krona analysis of the relative abundance of intestinal bacterial OTUs identified in fish fed D3. (HTML 246 kb)

Additional file 6: Figure S5. Krona analysis of the relative abundance of intestinal bacterial OTUs identified in fish fed D4. (HTML 250 kb)

Additional file 7: Table S2. List of proteins detected in anterior and posterior intestine pooled samples. Data on protein expression are mean ± SEM of 4 pools fed the experimental diets. The number of the contig in the Sea Bream Database (http://nutrigroup-iats.org/seabreamdb) is indicated. (PDF 1435 kb)

Additional file 8: Table S3. List of proteins detected in anterior intestine samples. Data on protein expression are mean ± SEM of 6 fish fed the experimental diets. The number of the contig in the Sea Bream Database (http://nutrigroup-iats.org/seabreamdb) is indicated. (PDF 562 kb)

Additional file 9: Table S4. List of differentially expressed proteins (*P* < 0.05, one-way ANOVA). Data on protein expression are mean ± SEM of 6 fish fed the experimental diets. Different superscript letters in each row indicate significant differences among experimental groups. The number of the contig in the Sea Bream Database (http://nutrigroup-iats.org/seabreamdb) is indicated. (DOCX 63 kb)

## Abbreviations

CF: Condition factor
FM: Fish meal
FO: Fish oil
GI: Gastrointestinal
GSB: Gilthead sea bream
NGS: Next generation sequencing
NL: Non-lethal
OTU: Operational taxonomic units
p.i.: Post-infection
SCFA: Short-chain fatty acids
SGR: Specific growth rate
VO: Vegetable oil
