# Supplementary figures and images for: Under control: how a dietary additive can restore the gut microbiome and proteomic profile, and improve disease resilience in a marine teleostean fish fed vegetable diets
Source: Microbiome. 2017 Dec 28;5:164. doi: 10.1186/s40168-017-0390-3 (PMC5745981; doi:10.1186/s40168-017-0390-3)

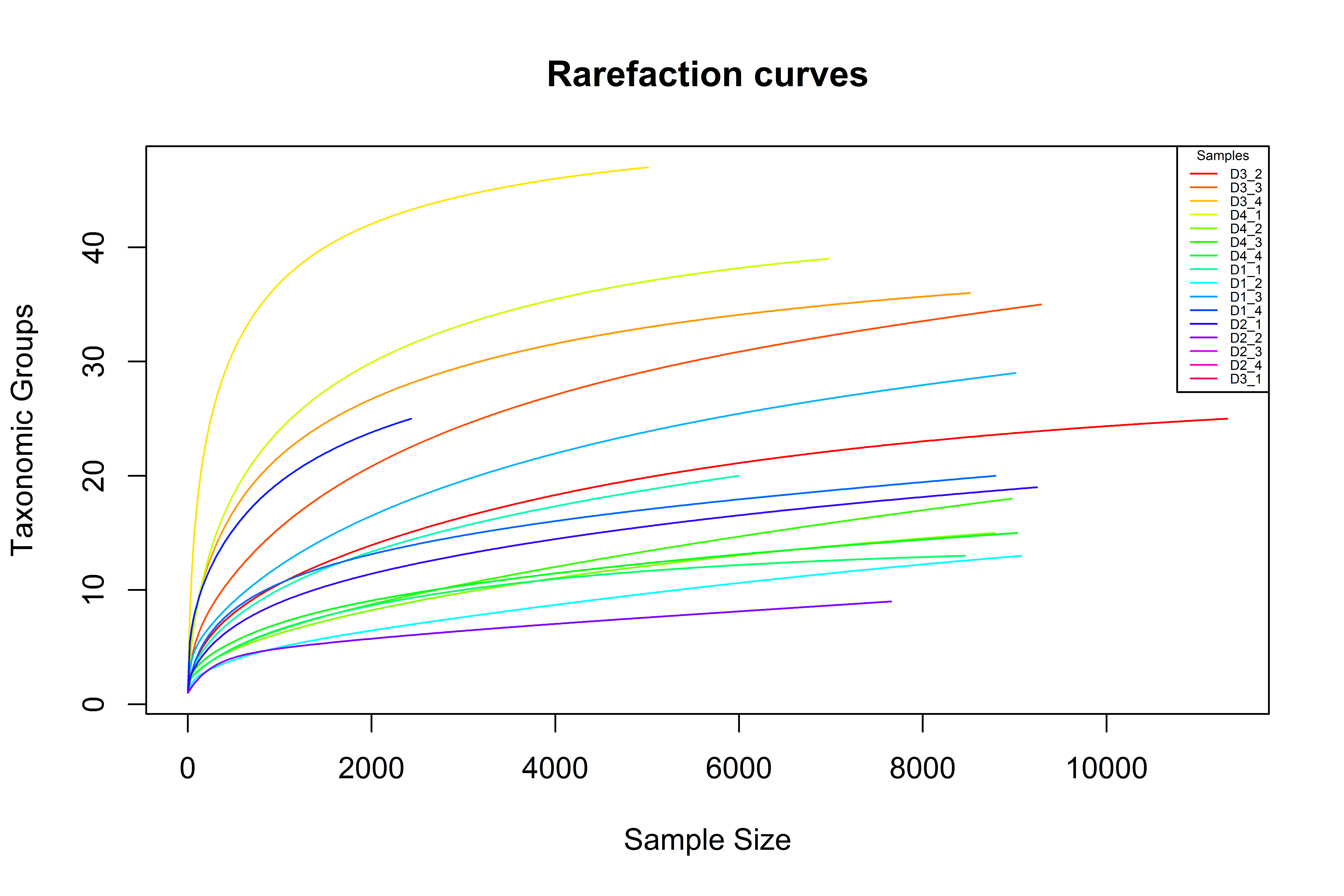

Supplement: Supplementary file 1 — Rarefaction curves obtained from the sequencing data of the four different pooled samples per dietary group. (TIFF 437 kb) [file 40168_2017_390_MOESM1_ESM.tif]
