# Supplementary material for: Under control: how a dietary additive can restore the gut microbiome and proteomic profile, and improve disease resilience in a marine teleostean fish fed vegetable diets
Source: Microbiome. 2017 Dec 28;5:164. doi: 10.1186/s40168-017-0390-3 (PMC5745981; doi:10.1186/s40168-017-0390-3)
Supplement: Supplementary file 3 — Krona visualisation of the relative abundance of intestinal bacterial OTUs identified in fish fed D1. (HTML 238 kb) [file 40168_2017_390_MOESM3_ESM.html]

Javascript must be enabled to view this page.

magnitude

 10875

 1

 1

 119

 119

 1

 1

 9

 8

 1

 1

 1

 4

 4

 1

 1

 3

 1

 1

 1

 1

 1

 2

 1

 1

 1

 1

 1

 1

 1

 1

 2

 1

 1

 23

 10

 1

 6

 6

 27

 11

 3

 10

 3

 2

 2

 1

 1

 3

 3

 1

 1

 1

 1

 39

 13

 1

 24

 1

 2

 2

 2

 1

 1

 1

 1

 1

 1

 1

 1

 7741

 6406

 23

 1310

 2

 1

 1

 26

 26

 2

 2

 14

 14

 2

 1

 1

 2

 1

 1

 5

 5

 3

 1

 1

 1

 1

 1

 1

 1

 1

 1

 3

 1

 1

 1

 20

 20

 6

 1

 5

 1

 1

 1

 1

 1

 1

 2154

 2

 1

 1

 930

 12

 21

 1

 1

 805

 122

 258

 14

 1

 5

 8

 624

 1

 13

 2

 606

 1

 1

 1

 1
