# Supplementary material for: Under control: how a dietary additive can restore the gut microbiome and proteomic profile, and improve disease resilience in a marine teleostean fish fed vegetable diets
Source: Microbiome. 2017 Dec 28;5:164. doi: 10.1186/s40168-017-0390-3 (PMC5745981; doi:10.1186/s40168-017-0390-3)
Supplement: Supplementary file 4 — Krona analysis of the relative abundance of intestinal bacterial OTUs identified in fish fed D2. (HTML 243 kb) [file 40168_2017_390_MOESM4_ESM.html]

Javascript must be enabled to view this page.

magnitude

 12023

 1

 1

 1

 1

 15

 1

 5

 7

 2

 1

 1

 2

 1

 1

 211

 209

 1

 1

 7

 7

 15

 4

 11

 1

 1

 3

 1

 1

 1

 7

 1

 6

 1

 1

 3

 1

 1

 1

 1

 1

 1

 1

 6

 1

 1

 1

 2

 1

 3

 3

 1

 1

 6

 1

 1

 1

 1

 1

 1

 1

 1

 46

 20

 13

 13

 2

 2

 135

 135

 1

 1

 2

 2

 1

 1

 1

 1

 10

 10

 10

 10

 1

 1

 6

 6

 3

 1

 1

 1

 1

 1

 70

 1

 4

 1

 32

 32

 1

 1

 5

 1

 1

 1

 1

 1

 2

 1

 1

 2

 1

 1

 1

 1

 2

 1

 1

 2

 1

 1

 1

 1

 8382

 8317

 5

 6

 51

 3

 1

 1

 1

 1

 3

 3

 11

 11

 10

 10

 37

 2

 4

 31

 52

 1

 51

 181

 181

 1

 1

 14

 1

 1

 1

 10

 1

 2

 1

 1

 1

 1

 1

 1

 109

 109

 62

 7

 55

 1

 1

 1

 1

 2316

 1

 1

 21

 286

 355

 1

 1

 1625

 15

 1

 1

 8

 22

 1

 21

 1

 1

 220

 1

 38

 2

 5

 163

 1

 1

 1

 8

 2

 2
