# Supplementary material for: Under control: how a dietary additive can restore the gut microbiome and proteomic profile, and improve disease resilience in a marine teleostean fish fed vegetable diets
Source: Microbiome. 2017 Dec 28;5:164. doi: 10.1186/s40168-017-0390-3 (PMC5745981; doi:10.1186/s40168-017-0390-3)
Supplement: Supplementary file 5 — Krona analysis of the relative abundance of intestinal bacterial OTUs identified in fish fed D3. (HTML 246 kb) [file 40168_2017_390_MOESM5_ESM.html]

Javascript must be enabled to view this page.

magnitude

 12882

 1

 1

 1

 1

 7

 2

 4

 1

 2

 1

 1

 1

 1

 315

 304

 1

 1

 1

 1

 7

 4

 4

 2

 2

 1

 1

 1

 1

 1

 1

 2

 1

 1

 1

 1

 16

 1

 15

 2

 1

 1

 3

 1

 1

 1

 1

 1

 1

 1

 1

 1

 2

 2

 1

 1

 1

 1

 8

 8

 6

 1

 1

 4

 1

 1

 16

 1

 2

 11

 1

 1

 31

 2

 25

 2

 2

 1

 1

 58

 39

 16

 1

 1

 1

 1

 1

 4

 1

 1

 1

 1

 2

 2

 2

 2

 1

 1

 4

 3

 1

 165

 22

 3

 1

 1

 86

 52

 1

 1

 2

 1

 1

 1

 1

 5

 3

 2

 1

 1

 1

 1

 2

 1

 1

 3

 1

 2

 2

 1

 1

 10435

 10416

 1

 11

 1

 6

 1

 1

 1

 1

 1

 1

 11

 11

 35

 4

 31

 42

 41

 1

 1

 1

 96

 96

 1

 1

 1

 1

 15

 15

 1

 1

 1

 1

 11

 5

 1

 1

 1

 2

 1

 6

 1

 4

 1

 3

 1

 1

 1

 3

 3

 1

 1

 7

 1

 1

 2

 1

 1

 1

 353

 353

 94

 16

 78

 1

 1

 1

 1

 1

 1

 241

 1

 3

 78

 56

 1

 1

 2

 75

 23

 1

 39

 24

 15

 1

 1

 791

 8

 30

 1

 1

 2

 1

 710

 2

 3

 1

 2

 30

 2

 1

 1
