# Supplementary material for: Under control: how a dietary additive can restore the gut microbiome and proteomic profile, and improve disease resilience in a marine teleostean fish fed vegetable diets
Source: Microbiome. 2017 Dec 28;5:164. doi: 10.1186/s40168-017-0390-3 (PMC5745981; doi:10.1186/s40168-017-0390-3)
Supplement: Supplementary file 6 — Krona analysis of the relative abundance of intestinal bacterial OTUs identified in fish fed D4. (HTML 250 kb) [file 40168_2017_390_MOESM6_ESM.html]

Javascript must be enabled to view this page.

magnitude

 11769

 2

 2

 11

 2

 4

 5

 110

 110

 1

 1

 1

 1

 3

 2

 1

 1954

 1934

 1

 1

 1

 1

 1

 1

 1

 13

 48

 48

 1

 1

 10

 1

 9

 1

 1

 1

 1

 54

 23

 31

 8

 1

 6

 1

 5

 3

 1

 1

 3

 1

 1

 1

 1

 1

 1

 1

 6

 6

 1

 1

 1

 1

 1

 1

 2

 2

 11

 1

 1

 2

 4

 3

 1

 1

 1

 1

 1

 1

 1

 1

 1

 1

 35

 26

 6

 2

 1

 1

 1

 1145

 356

 756

 28

 1

 4

 139

 1

 4

 133

 1

 1

 1

 16

 16

 7

 4

 1

 1

 1

 1

 1

 12

 7

 5

 1

 1

 12

 12

 1

 1

 1

 1

 42

 1

 1

 38

 2

 1

 1

 1

 1

 84

 84

 3

 1

 1

 1

 11

 9

 1

 1

 1

 1

 1

 1

 2

 2

 1

 1

 2

 1

 1

 3

 3

 1

 1

 1

 1

 5

 2

 2

 1

 5289

 5202

 22

 54

 11

 1

 1

 3

 3

 1

 1

 1

 1

 7

 7

 9

 9

 71

 1

 70

 1

 1

 48

 1

 1

 18

 1

 17

 10

 1

 1

 45

 2

 43

 1

 1

 1

 1

 1

 1

 34

 34

 2

 2

 14

 1

 5

 1

 1

 1

 1

 1

 1

 1

 1

 16

 16

 3

 1

 1

 1

 1

 1

 4

 1

 1

 1

 1

 6

 6

 1

 1

 1

 1

 612

 612

 1

 1

 27

 18

 9

 1

 1

 1

 1

 806

 153

 13

 1

 21

 1

 1

 420

 40

 3

 153

 1

 1

 1

 1

 315

 315

 1

 1

 666

 12

 1

 31

 1

 1

 1

 1

 280

 1

 333

 2

 2

 2

 1

 1
