# Supplementary material for: Under control: how a dietary additive can restore the gut microbiome and proteomic profile, and improve disease resilience in a marine teleostean fish fed vegetable diets
Source: Microbiome. 2017 Dec 28;5:164. doi: 10.1186/s40168-017-0390-3 (PMC5745981; doi:10.1186/s40168-017-0390-3)
Supplement: Supplementary file 7 — List of proteins detected in anterior and posterior intestine pooled samples. Data on protein expression are mean ± SEM of 4 pools fed the experimental diets. The number of the contig in the Sea Bream Database (http://nutrigroup-iats.org/seabreamdb) is indicated. (PDF 1435 kb) [file 40168_2017_390_MOESM7_ESM.pdf]

Table S2. List of proteins detected in anterior and posterior intestine pooled samples. Data on protein expression are mean ± SEM of 4 pools fed the experimental diets. The number of the contig in the Sea Bream Database (http://nutrigroup-iats.org/seabreamdb) is indicated.

Contig	Protein Description	Symbol	Anterior	Posterior	Ratio Ant/Pos
C2_6629	1,4-alpha-glucan-branching enzyme	GBE1	0.88±0.1	0.91±0.03	0.98
C2_4764	116 kDa U5 small nuclear ribonucleoprotein component	EFTUD2	0.74±0.09	0.71±0.05	1.03
C2_299	14-3-3 protein beta/alpha-1	YWHAB	1.45±0.23	2.18±0.09	0.67
C2_268	14-3-3 protein epsilon	YWHAE	1.28±0.2	2.01±0.13	0.63
C2_2474	14-3-3 protein gamma-1	YWHAG	1.8±0.41	2.72±0.09	0.66
C2_1017	14-3-3 protein zeta	YWHAZ	1.33±0.14	4.41±0.38	0.30
C2_34474	14-3-3-like protein 2	YWHAQ	1.3±0.11	1.85±0.13	0.70
C2_4902	17-beta-hydroxysteroid dehydrogenase 14	HSD17B14	0.93±0.05	2.33±0.09	0.40
C2_3100	1-acylglycerol-3-phosphate O-acyltransferase ABHD5	ABHD5	0.85±0.07	0.78±0.13	1.10
C2_15440	1-phosphatidylinositol phosphodiesterase	PLCD1	0.65±0.12	0.4±0.06	1.65
C2_12986	1-phosphatidylinositol-4,5-bisphosphate phosphodiesterase delta-1	PLCD1	0.76±0.08	1.15±0.16	0.66
C2_4412	1-phosphatidylinositol-4,5-bisphosphate phosphodiesterase gamma-2	PLCG2	1.13±0.08	2.08±0.27	0.54
C2_3170	2,4-dienoyl-CoA reductase, mitochondrial	DECR1	1.16±0.1	0.83±0.03	1.39
C2_1520	26S protease regulatory subunit 10B	PSMC6	1.37±0.21	1.43±0.04	0.96
C2_4264	26S protease regulatory subunit 4	PSMC1	1.2±0.2	1.78±0.08	0.68
C2_1666	26S protease regulatory subunit 6A	PSMC3	1.44±0.24	1.61±0.08	0.90
C2_482	26S protease regulatory subunit 6B	PSMC4	1.72±0.4	2.15±0.08	0.80
C2_3002	26S protease regulatory subunit 7	PSMC2	1.18±0.07	1.17±0.04	1.01
C2_514	26S protease regulatory subunit 8	PSMC5	0.91±0.04	0.67±0.03	1.37
C2_2728	26S proteasome non-ATPase regulatory subunit 1	PSMD1	1.1±0.06	1.33±0.04	0.82
C2_993	26S proteasome non-ATPase regulatory subunit 10	PSMD10	0.97±0.03	0.95±0.08	1.01
C2_2261	26S proteasome non-ATPase regulatory subunit 11	PSMD11	1.8±0.47	0.65±0.04	2.76
C2_1392	26S proteasome non-ATPase regulatory subunit 12	PSMD12	1.15±0.09	1.22±0.04	0.95
C2_790	26S proteasome non-ATPase regulatory subunit 13	PSMD13	0.95±0.07	1.39±0.06	0.69
C2_807	26S proteasome non-ATPase regulatory subunit 14	PSMD14	1.08±0.11	1.3±0.05	0.84
C2_4556	26S proteasome non-ATPase regulatory subunit 2	PSMD2	1.37±0.17	1.76±0.03	0.78
C2_1006	26S proteasome non-ATPase regulatory subunit 3	PSMD3	1.03±0.08	1.51±0.11	0.68
C2_5351	26S proteasome non-ATPase regulatory subunit 5	PSMD5	1.1±0.05	1.39±0.18	0.79
C2_8032	26S proteasome non-ATPase regulatory subunit 6	PSMD6	1±0.05	1.5±0.13	0.67
C2_364	26S proteasome non-ATPase regulatory subunit 7	PSMD7	1.51±0.24	2.1±0.12	0.72
C2_1843	26S proteasome non-ATPase regulatory subunit 8	PSMD8	0.98±0.05	1.45±0.16	0.68
C2_6795	2-acylglycerol O-acyltransferase 2-A	MOGAT2	1.67±0.29	1.2±0.1	1.39
C2_8379	2-amino-3-carboxymuconate-6-semialdehyde decarboxylase	ACMSD	1.32±0.14	1.03±0.03	1.28
C3_c25398	2'-deoxynucleoside 5'-phosphate N-hydrolase 1-like	DNPH1	1.55±0.37	1.66±0.17	0.94
C2_12608	2-hydroxyacyl-CoA lyase 1	HACL1	1.05±0.1	0.68±0.03	1.53
C2_8246	2-hydroxyacyl-CoA lyase 1	HACL1	1.04±0.07	0.46±0.07	2.28
C2_19129	2-oxoglutarate dehydrogenase, mitochondrial	OGDH	0.8±0.07	0.69±0.07	1.16
C2_61153	2-oxoglutarate dehydrogenase, mitochondrial	OGDH	0.97±0.08	0.51±0.05	1.89
C2_9582	2-oxoglutarate dehydrogenase, mitochondrial	OGDH	0.96±0.08	0.49±0.05	1.96
C2_34981	3'(2'),5'-bisphosphate nucleotidase 1	BPNT1	1.29±0.12	1.39±0.07	0.93
C2_7568	3-hydroxyacyl-CoA dehydrogenase type-2	HSD17B10	0.96±0.04	0.95±0.06	1.02
C2_1004	3-hydroxyanthranilate 3,4-dioxygenase	HAAO	1.5±0.22	1.72±0.03	0.88
C2_850	3-hydroxybutyrate dehydrogenase type 2	BDH2	0.78±0.09	0.55±0.03	1.42
C2_1580	3-hydroxyisobutyrate dehydrogenase, mitochondrial	HIBADH	0.87±0.06	0.47±0.04	1.87
C2_4404	3-hydroxyisobutyryl-CoA hydrolase, mitochondrial	HIBCH	1.12±0.15	0.43±0.04	2.61
C2_7520	3-ketoacyl-CoA thiolase B, peroxisomal	Acaa1b	1.24±0.12	1.12±0.17	1.11
C2_1174	3-ketoacyl-CoA thiolase, mitochondrial	ACAA2	1.24±0.18	0.74±0.05	1.68
C2_742	3-oxo-5-beta-steroid 4-dehydrogenase	AKR1D1	1.15±0.05	1.54±0.08	0.75
C2_8112	3-oxoacyl-[acyl-carrier-protein] reductase FabG	FABG	0.78±0.09	0.75±0.05	1.05
C2_2505	3-phosphoinositide-dependent protein kinase 1	PDPK1	0.9±0.05	1.69±0.19	0.53
C2_8805	40S ribosomal protein S10	RPS10	2.41±0.61	1.64±0.11	1.46
C2_367	40S ribosomal protein S11	RPS11	2.81±0.69	2.18±0.08	1.29
C2_583	40S ribosomal protein S12	RPS12	2.34±0.61	1.44±0.16	1.63
C2_14171	40S ribosomal protein S13	RPS13	2.28±0.62	1.86±0.17	1.23
C2_20708	40S ribosomal protein S14	RPS14	2.91±0.81	2.2±0.18	1.32
C2_26	40S ribosomal protein S15	RPS15	2.93±0.76	2.47±0.22	1.19
C2_414	40S ribosomal protein S15a	RPS15A	3.39±0.92	2.22±0.16	1.53
C2_7337	40S ribosomal protein S16	RPS16	3.14±0.94	1.78±0.12	1.77
C2_971	40S ribosomal protein S17	RPS17	3.66±1.14	2.19±0.23	1.67
C2_9151	40S ribosomal protein S18	RPS18	3.55±0.99	2.05±0.11	1.73
C2_955	40S ribosomal protein S19	RPS19	4.88±1.49	3.04±0.19	1.60
C2_684	40S ribosomal protein S2	RPS2	2.24±0.51	1.71±0.15	1.31

C2_232	40S ribosomal protein S20	RPS20	1.82±0.4	2.58±0.33	0.70
C2_11917	40S ribosomal protein S21	RPS21	1.76±0.37	1.34±0.16	1.31
C2_29474	40S ribosomal protein S23	RPS23	2.66±0.81	1.84±0.19	1.44
C2_1271	40S ribosomal protein S24	RPS24	1.88±0.38	1.27±0.1	1.48
C2_310	40S ribosomal protein S25	RPS25	3.56±0.97	2.43±0.12	1.46
C2_698	40S ribosomal protein S26	RPS26	2.63±0.67	1.85±0.15	1.42
C2_175	40S ribosomal protein S27	Rps27	1.57±0.38	1.18±0.11	1.33
C2_3275	40S ribosomal protein S28	RPS28	1.86±0.4	1.29±0.07	1.44
C2_23479	40S ribosomal protein S29	RPS29	1.9±0.47	1.22±0.13	1.56
C2_17873	40S ribosomal protein S3	RPS3	2.21±0.47	1.7±0.09	1.30
C2_2139	40S ribosomal protein S3a	Rps3a1	2.66±0.73	1.8±0.16	1.48
C2_593	40S ribosomal protein S4	GM15013	2.54±0.7	1.84±0.13	1.38
C2_433	40S ribosomal protein S5	RPS5	2.5±0.55	2.07±0.18	1.21
C2_164	40S ribosomal protein S6	RPS6	1.23±0.18	1.41±0.41	0.87
C2_133	40S ribosomal protein S7	RPS7	1.65±0.33	1.68±0.29	0.98
C2_4273	40S ribosomal protein S8	RPS8	1.72±0.35	1.5±0.22	1.15
C2_252	40S ribosomal protein S9	RPS9	2.6±0.65	2.18±0.13	1.19
C2_16	40S ribosomal protein SA	RPSA	1.82±0.35	1.38±0.14	1.32
C2_4721	4-aminobutyrate aminotransferase, mitochondrial	ABAT	1.14±0.06	0.76±0.02	1.50
C2_2062	4F2 cell-surface antigen heavy chain	SLC3A2	1.51±0.29	0.83±0.08	1.82
C2_5124	5'(3')-deoxyribonucleotidase, mitochondrial	NT5M	1.61±0.26	1.34±0.06	1.21
C2_53761	5'-3' exoribonuclease 1	XRN1	1.14±0.08	0.95±0.11	1.19
C2_16488	5'-3' exoribonuclease 2	XRN2	0.97±0.01	0.96±0.05	1.01
C2_66522	5'-3' exoribonuclease 2	XRN2	0.83±0.06	0.67±0.04	1.24
C2_10452	55 kDa erythrocyte membrane protein	MPP1	0.55±0.15	0.55±0.15	1.01
C2_4899	5'-AMP-activated protein kinase catalytic subunit alpha-1	PRKAA1	0.7±0.12	0.46±0.06	1.52
C2_9207	5-beta-cholestane-3-alpha,7-alpha-diol 12-alpha-hydroxylase	CYP8B1	1.3±0.18	0.91±0.23	1.42
C2_58937	5-oxoprolinase	OPLAH	0.99±0.05	0.68±0.05	1.46
C2_5222	60 kDa heat shock protein, mitochondrial	HSPD1	1.11±0.12	0.85±0.1	1.30
C2_47	60S acidic ribosomal protein P0	RPLP0	1.85±0.38	1.91±0.09	0.97
C2_46323	60S acidic ribosomal protein P1	Rplp1	1.36±0.15	1.07±0.04	1.27
C2_6923	60S acidic ribosomal protein P2	RPLP2	1.29±0.11	1.07±0.05	1.20
C2_167	60S ribosomal protein L10	RPL10	3.72±1.01	2.27±0.09	1.64
C2_67	60S ribosomal protein L10a	RPL10A	2.41±0.57	1.42±0.08	1.70
C2_236	60S ribosomal protein L11	RPL11	2.87±0.88	1.14±0.06	2.52
C2_587	60S ribosomal protein L12	RPL12	1.6±0.27	2.05±0.13	0.78
C2_453	60S ribosomal protein L13	RPL13	2.9±0.68	1.94±0.12	1.50
C2_441	60S ribosomal protein L13a	RPL13A	2.32±0.55	1.04±0.07	2.23
C2_142	60S ribosomal protein L14	RPL14	3.12±0.93	1.34±0.07	2.32
C2_279	60S ribosomal protein L15	RPL15	2.48±0.63	1.21±0.1	2.06
C2_1244	60S ribosomal protein L17	RPL17	2.97±0.88	1.3±0.04	2.29
C2_842	60S ribosomal protein L18	RPL18	1.8±0.36	0.88±0.03	2.05
C2_12358	60S ribosomal protein L18a	RPL18A	2.02±0.52	1.02±0.07	1.98
C2_700	60S ribosomal protein L19	RPL19	4±1.32	1.71±0.07	2.34
C2_94336	60S ribosomal protein L21	RPL21	2.81±0.7	1.5±0.06	1.87
C2_39656	60S ribosomal protein L22	RPL22	1.63±0.3	1.78±0.13	0.91
C2_373	60S ribosomal protein L23	RPL23	2.71±0.74	1.18±0.06	2.29
C2_1007	60S ribosomal protein L23a	Rpl23a	2.73±0.7	1.38±0.04	1.97
C2_119969	60S ribosomal protein L24	RPL24	5.15±1.75	1.54±0.21	3.35
C2_392	60S ribosomal protein L26	RPL26	2.56±0.69	1.45±0.11	1.77
C2_121623	60S ribosomal protein L27	RPL27	4.5±1.27	2.95±0.12	1.53
C2_343	60S ribosomal protein L27a	RPL27A	2.72±0.64	1.77±0.06	1.54
C2_1383	60S ribosomal protein L28	RPL28	4.41±1.21	4.75±0.29	0.93
C2_12	60S ribosomal protein L3	RPL3	2.34±0.67	0.94±0.06	2.49
C2_17126	60S ribosomal protein L30	RPL30	3.52±1	2.27±0.11	1.55
C2_111961	60S ribosomal protein L31	RPL31	2.1±0.44	0.95±0.08	2.22
C2_361	60S ribosomal protein L32	Rpl32	2.47±0.6	1.36±0.09	1.81
C2_72598	60S ribosomal protein L34	Rpl34	8.36±2.83	5.21±0.53	1.60
C2_89835	60S ribosomal protein L35	RPL35	2.57±0.58	2.63±0.12	0.98
C2_3033	60S ribosomal protein L35a	RPL35A	2.66±0.7	1.24±0.04	2.14
C2_2019	60S ribosomal protein L36	Rpl36	2.1±0.39	1.69±0.12	1.24
C2_1788	60S ribosomal protein L36a	Rpl36a	2.66±0.64	1.1±0.11	2.42
C2_9484	60S ribosomal protein L38	RPL38	3.2±0.92	1.53±0.1	2.10
C2_25	60S ribosomal protein L4	RPL4	2.59±0.72	1.25±0.07	2.07
C2_143	60S ribosomal protein L5	RPL5	2.25±0.48	1.91±0.13	1.18
C2_827	60S ribosomal protein L6	RPL6	2.87±0.83	1.31±0.07	2.19
C2_434	60S ribosomal protein L7	RPL7	2.98±0.85	1.37±0.12	2.18

C2_98	60S ribosomal protein L7a	RPL7A	2.38±0.53	1.39±0.11	1.71
C2_174	60S ribosomal protein L8	RPL8	2.66±0.72	1.1±0.09	2.42
C2_156	60S ribosomal protein L9	RPL9	2.96±0.91	1.14±0.04	2.59
C2_14127	6-phosphofructo-2-kinase/fructose-2,6-biphosphatase 1	PFKFB1	1.52±0.18	1.64±0.09	0.93
C2_11947	6-phosphofructo-2-kinase/fructose-2,6-biphosphatase 4	PFKFB4	1.43±0.18	2.36±0.12	0.61
C2_3317	6-phosphofructokinase type C	PFKP	1.18±0.07	1.6±0.17	0.74
C2_4969	6-phosphofructokinase, liver type	PFKP	1.19±0.11	1.22±0.16	0.97
C2_531	6-phosphogluconate dehydrogenase, decarboxylating	PGD	1.35±0.17	1.62±0.02	0.84
C2_6550	6-phosphogluconolactonase	PGLS	0.91±0.08	0.78±0.07	1.16
C2_14692	6-pyruvoyl tetrahydrobiopterin synthase	PTS	0.78±0.11	0.77±0.09	1.01
C2_25027	78 kDa glucose-regulated protein	HSPA5	1.9±0.39	1.14±0.02	1.67
C2_14888	78 kDa glucose-regulated protein	HSPA5	1.41±0.16	0.64±0.05	2.23
C2_17792	7-dehydrocholesterol reductase	DHCR7	1.34±0.13	0.77±0.09	1.74
C2_4132	90kDa heat shock protein alpha 1	HSP90AA1	0.65±0.15	1.17±0.09	0.55
C2_42	90kDa heat shock protein beta	HSP90AB1	1.52±0.23	3.07±0.48	0.49
C2_1584	Abhydrolase domain-containing protein 13	ABHD13	1.21±0.14	0.93±0.18	1.29
C2_40874	Abhydrolase domain-containing protein 14B	ABHD14B	1.4±0.19	2.05±0.2	0.68
C2_7286	Abhydrolase domain-containing protein 14B	ABHD14B	1.06±0.07	1.07±0.04	0.99
C2_98839	Acetolactate synthase-like protein	ILVBL	1.66±0.25	1.09±0.06	1.52
C2_13751	Acetolactate synthase-like protein	ILVBL	1.51±0.2	0.69±0.04	2.20
C2_53898	Acetyl-CoA acetyltransferase, cytosolic	ACAT2	1.73±0.3	0.6±0.03	2.89
C2_181	Acetyl-CoA acetyltransferase, mitochondrial	ACAT1	1.03±0.13	1.02±0.13	1.01
C2_5360	Acetyl-coenzyme A synthetase 2-like, mitochondrial	ACSS1	0.92±0.04	1.99±0.09	0.46
C2_14060	Acetyl-coenzyme A synthetase, cytoplasmic	ACSS2	0.97±0.12	2.01±0.35	0.48
C2_10641	Acetyl-coenzyme A synthetase, cytoplasmic	ACSS2	0.9±0.05	1.67±0.23	0.54
C2_2879	Acid ceramidase	ASAH1	0.91±0.12	0.61±0.05	1.49
C2_3009	Acid sphingomyelinase-like phosphodiesterase 3b	SMPDL3B	0.69±0.11	0.29±0.03	2.42
C2_8126	Acid trehalase-like protein 1	ATHL1	0.62±0.14	0.76±0.05	0.82
C2_86088	Acidic leucine-rich nuclear phosphoprotein 32 family member B	Anp32b	1.22±0.22	1.26±0.11	0.97
C2_18917	Acidic leucine-rich nuclear phosphoprotein 32 family member E	Anp32e	1.09±0.04	1.19±0.1	0.92
C3_lrc63576	Acidic mammalian chitinase	CHIA	0.49±0.18	0.25±0.04	1.95
C2_18809	Aconitate hydratase, mitochondrial	ACO2	2.6±0.72	1.61±0.18	1.61
C2_33453	Aconitate hydratase, mitochondrial	ACO2	2.27±0.52	1.33±0.15	1.70
C2_15154	Aconitate hydratase, mitochondrial	ACO2	0.35±0.22	0.13±0.02	2.72
C2_210	Actin, cytoplasmic 1	ACTB	1.71±0.29	3.76±0.52	0.46
C2_102126	Actin, cytoplasmic 2	ACTG1	1.68±0.3	2.87±0.36	0.58
C2_89711	Actin-binding LIM protein 2	ABLIM2	0.95±0.08	6.2±1.77	0.15
C2_376	Actin-like protein 6A	ACTL6A	0.7±0.12	0.58±0.07	1.21
C2_8398	Actin-related protein 10	ACTR10	1.67±0.26	2.57±0.11	0.65
C2_1852	Actin-related protein 2	ACTR2	0.56±0.15	0.5±0.03	1.11
C3_c11102	Actin-related protein 2/3 complex subunit 1A	ARPC1A	1.04±0.04	1.63±0.26	0.64
C2_4212	Actin-related protein 2/3 complex subunit 1B	ARPC1B	1.73±0.3	2.1±0.15	0.82
C2_22529	Actin-related protein 2/3 complex subunit 2	ARPC2	1.59±0.24	2.11±0.12	0.76
C2_4166	Actin-related protein 2/3 complex subunit 3	ARPC3	1.43±0.26	2.3±0.26	0.62
C2_427	Actin-related protein 2/3 complex subunit 4	ARPC4	1.46±0.21	1.98±0.19	0.73
C2_503	Actin-related protein 2/3 complex subunit 5	ARPC5	2.3±0.65	3.81±0.31	0.61
C2_3453	Actin-related protein 2-A	ACTR2	1.05±0.22	0.44±0.03	2.36
C2_42139	Actin-related protein 2-B	ACTR2	0.69±0.11	0.71±0.04	0.97
C2_1771	Actin-related protein 3	ACTR3	1.47±0.29	2.47±0.2	0.59
C2_2259	Activated RNA polymerase II transcriptional coactivator p15	SUB1	0.75±0.12	0.45±0.06	1.69
C2_102	Activating signal cointegrator 1 complex subunit 1	ASCC1	0.67±0.12	0.43±0.05	1.57
C2_2407	Activator of 90 kDa heat shock protein ATPase homolog 1	AHSA1	1.12±0.07	1.36±0.02	0.82
C2_20973	Active breakpoint cluster region-related protein	ABR	1.08±0.03	1.11±0.07	0.97
C2_11239	Acyl carrier protein, mitochondrial	NDUFAB1	1.24±0.09	0.87±0.05	1.44
C2_2532	Acylamino-acid-releasing enzyme	APEH	0.79±0.1	0.62±0.04	1.27
C2_47266	Acyl-CoA dehydrogenase family member 11	ACAD11	0.97±0.19	0.78±0.08	1.25
C2_3692	Acyl-CoA dehydrogenase family member 9, mitochondrial	ACAD9	0.85±0.12	0.4±0.03	2.14
C2_8427	Acyl-CoA synthetase family member 2, mitochondrial	ACSF2	0.98±0.04	0.51±0.03	1.92
C2_649	Acyl-CoA-binding protein	DBI	1.25±0.12	2.35±0.59	0.53
C2_1332	Acyl-CoA-binding protein homolog	DBI	2.51±0.8	2.83±0.22	0.89
C2_6592	Acyl-coenzyme A thioesterase 11	ACOT11	0.99±0.08	1.49±0.04	0.66
C2_39905	Acyl-coenzyme A thioesterase 13	ACOT13	0.88±0.11	0.8±0.1	1.10
C2_9418	Acyl-coenzyme A thioesterase 3	ACOT1	1.15±0.13	3.34±0.1	0.34
C2_1648	Acyl-protein thioesterase 1	LYPLA1	1.02±0.05	1.03±0.02	0.99
C2_4253	Acyl-protein thioesterase 2	LYPLA2	1.03±0.04	2.62±0.56	0.40
C3_c23213	Adapter molecule crk	CRK	0.66±0.11	0.83±0.07	0.79
C2_584	Adaptin ear-binding coat-associated protein 2	NECAP2	1.08±0.05	1.59±0.24	0.68

C2_11692	Adenine phosphoribosyltransferase	APRT	1.2±0.07	1.36±0.15	0.88
C2_7078	Adenosine deaminase	ADA	0.77±0.11	0.44±0.02	1.73
C2_5126	Adenosine kinase	ADK	0.96±0.02	0.66±0.03	1.46
C2_39087	Adenosine kinase 1	ADK	0.82±0.1	0.63±0.05	1.31
C2_2187	Adenosylhomocysteinase A	AHCY	0.8±0.13	0.19±0.02	4.11
C2_6468	Adenylate kinase 2, mitochondrial	AK2	0.79±0.07	0.99±0.28	0.79
C2_6546	Adenylosuccinate lyase	ADSL	1.01±0.09	0.97±0.05	1.04
C2_41283	Adenylyl cyclase-associated protein 1	CAP1	1.14±0.05	0.94±0.07	1.21
C2_75777	Adenylyl cyclase-associated protein 1	CAP1	0.91±0.07	0.67±0.06	1.37
C2_927	ADP/ATP translocase 2	SLC25A5	1.18±0.22	0.69±0.06	1.69
C2_7332	ADP-ribose pyrophosphatase, mitochondrial	NUDT9	1.11±0.13	1.54±0.07	0.72
C2_1001	ADP-ribosylation factor 1	ARF1	1.44±0.19	2.68±0.37	0.54
C2_487	ADP-ribosylation factor 1-like 2	ARF1	1.61±0.25	2.57±0.16	0.62
C2_2038	ADP-ribosylation factor 4	ARF4	1.38±0.22	2.49±0.38	0.55
C2_1092	ADP-ribosylation factor 6	ARF6	1.35±0.14	1.95±0.2	0.69
C2_12596	ADP-ribosylation factor GTPase-activating protein 2	ARFGAP2	0.81±0.09	0.44±0.04	1.86
C2_1966	ADP-ribosylation factor-like protein 1	ARL1	1.31±0.13	1.45±0.16	0.90
C2_20513	ADP-ribosylation factor-like protein 8A	ARL8A	0.67±0.11	0.6±0.04	1.12
C2_2357	ADP-ribosylation factor-like protein 8B	ARL8B	0.86±0.06	0.91±0.07	0.94
C2_3920	ADP-sugar pyrophosphatase	NUDT5	0.85±0.07	0.57±0.04	1.49
C2_1101	Adrenodoxin, mitochondrial	FDX1	1.01±0.09	0.85±0.03	1.18
C2_36260	Afadin	MLLT4	1.08±0.09	1.35±0.08	0.80
C2_970	Aflatoxin B1 aldehyde reductase member 2	AKR7A2	1.19±0.06	1.33±0.02	0.89
C2_4151	Agmatinase, mitochondrial	AGMAT	0.69±0.14	0.2±0.02	3.47
C2_4703	Alanine--glyoxylate aminotransferase 2-like 1	AGXT2	0.9±0.1	1.46±0.15	0.61
C2_9243	Alanine--glyoxylate aminotransferase 2-like 2	AGXT2	0.69±0.1	1.17±0.06	0.60
C2_28761	Alanyl-tRNA editing protein Aarsd1	AARSD1	1.01±0.17	1.53±0.32	0.66
C2_8373	Alanyl-tRNA synthetase, cytoplasmic	AARS	0.93±0.03	1.1±0.06	0.84
C2_4628	Alanyl-tRNA synthetase, cytoplasmic	AARS	0.81±0.07	0.71±0.07	1.15
C2_3044	Alcohol dehydrogenase [NADP+] A	AKR1A1	0.79±0.07	2.02±0.15	0.39
C2_996	Alcohol dehydrogenase [NADP+] B	AKR1A1	0.91±0.03	1.1±0.1	0.83
C2_189	Alcohol dehydrogenase 1	ADH1C	0.73±0.14	0.4±0.04	1.81
C2_288	Alcohol dehydrogenase class-3	ADH5	0.76±0.12	0.45±0.05	1.69
C2_43704	Alcohol dehydrogenase class-3 chain H	ADH5	0.79±0.16	0.11±0.01	7.24
C2_33526	Alcohol dehydrogenase class-3 chain L	ADH5	0.77±0.13	0.19±0.02	4.02
C2_2085	Aldehyde dehydrogenase family 1 member L1	ALDH1L1	1.36±0.13	1.7±0.11	0.80
C2_4444	Aldehyde dehydrogenase family 16 member A1	ALDH16A1	1.25±0.11	2.44±0.12	0.51
C2_77670	Aldehyde dehydrogenase family 8 member A1	ALDH8A1	0.72±0.11	0.66±0.05	1.10
C2_53791	Aldehyde dehydrogenase family 8 member A1	ALDH8A1	0.94±0.1	0.85±0.03	1.10
C2_3814	Aldehyde dehydrogenase family 9 member A1	ALDH8A1	1.59±0.25	2±0.42	0.79
C2_8710	Aldehyde dehydrogenase family 9 member A1-B	ALDH9A1B	1.53±0.28	1.44±0.22	1.06
C2_399	Aldehyde dehydrogenase, mitochondrial	ALDH2	1.61±0.23	0.87±0.05	1.85
C2_97934	Aldehyde oxidase	AOX1	0.92±0.1	0.67±0.06	1.36
C2_11977	Aldehyde oxidase	AOX1	0.85±0.11	0.51±0.06	1.66
C2_83262	Aldehyde oxidase	AOX1	0.84±0.11	0.49±0.06	1.73
C2_3792	Aldose 1-epimerase	GALM	0.8±0.15	0.65±0.11	1.22
C2_1189	Aldose reductase	AKR1B1	1.02±0.02	1.96±0.13	0.52
C2_11780	Allantoinase, mitochondrial	ALN	0.8±0.11	0.69±0.03	1.15
C2_27912	Allograft inflammatory factor 1-like	AIF1L	0.95±0.09	1.63±0.28	0.58
C2_97568	Alpha-(1,6)-fucosyltransferase	FUT8	0.7±0.12	0.36±0.04	1.94
C2_14294	Alpha-1-antitrypsin homolog	SERPINA1	0.93±0.04	0.81±0.14	1.15
C2_6331	Alpha-1-macroglobulin	Pzp	0.96±0.06	1.07±0.17	0.90
C2_9982	Alpha-2-antiplasmin	SERPINF2	0.89±0.05	0.75±0.1	1.19
C2_22548	Alpha-2-HS-glycoprotein	AHSG	1±0.08	1.66±0.59	0.60
s_flp0005a11_f_1	Alpha-2-macroglobulin	Pzp	1.14±0.11	1.33±0.21	0.86
C2_23524	Alpha-2-macroglobulin-like protein 1	A2ML1	0.94±0.03	1.87±0.82	0.50
C2_1801	Alpha-actinin-3	Actn3	1.85±0.37	1.98±0.19	0.93
C2_26557	Alpha-actinin-4	ACTN4	1.72±0.29	1.7±0.13	1.01
C2_25967	Alpha-adducin	ADD1	1.28±0.1	2.06±0.35	0.62
C2_1946	Alpha-aminoadipic semialdehyde dehydrogenase	ALDH7A1	0.88±0.13	0.44±0.05	1.97
C2_16242	Alpha-aspartyl dipeptidase	AAD-A	1.87±0.3	1.7±0.06	1.10
C2_440	Alpha-enolase	ENO1	1.12±0.06	2.25±0.14	0.50
C2_23595	Alpha-glucosidase 2	GAA	1±0.07	1.19±0.06	0.84
C2_17154	Alpha-mannosidase 2C1	MAN2C1	0.79±0.1	0.54±0.04	1.47
C2_52709	Alpha-methylacyl-CoA racemase	AMACR	0.72±0.1	0.29±0.02	2.46
C2_1063	Alpha-N-acetylgalactosaminidase	NAGA	0.84±0.09	1.03±0.1	0.81
C2_7302	Alpha-N-acetylglucosaminidase	NAGLU	0.79±0.1	0.6±0.07	1.33

C2_62996	Alpha-N-acetylglucosaminidase	NAGLU	0.67±0.14	0.32±0.04	2.11
C2_5411	Alpha-parvin	PARVA	52.87±17.6	76.65±8.3	0.69
C2_1920	Alpha-soluble NSF attachment protein	NAPA	0.95±0.07	1.48±0.15	0.65
C2_2739	Amiloride-sensitive amine oxidase [copper-containing]	AOC1	0.82±0.08	0.63±0.08	1.30
C2_7596	Amine oxidase [flavin-containing]	MAOB	1.46±0.24	0.39±0.01	3.75
C2_23714	Amine oxidase [flavin-containing] A	MAOA	0.93±0.12	0.44±0.03	2.11
C2_52395	Amine sulfotransferase	Gm4794	0.92±0.04	1.41±0.19	0.65
C2_616	Aminoacyl tRNA synthase complex-interacting multifunctional protein 1	AIMP1	1.51±0.28	1.99±0.12	0.76
C2_12687	Aminoacyl tRNA synthase complex-interacting multifunctional protein 2	AIMP2	1.02±0.02	1.11±0.02	0.92
C2_1372	Aminoacylase-1	ACY1	0.77±0.12	0.65±0.06	1.18
C2_73930	Aminoglycoside phosphotransferase domain-containing protein 1	HYKK	1.22±0.14	3.68±0.17	0.33
C3_c17712	Aminomethyltransferase, mitochondrial	AMT	1.48±0.16	0.61±0.02	2.41
C2_7522	Aminopeptidase N	ANPEP	0.71±0.11	0.84±0.11	0.84
C2_16955	AMP deaminase 2	AMPD2	1.37±0.17	2.19±0.14	0.62
C2_43976	Amyloid beta A4 precursor protein-binding family B member 1-interacting protein	APBB1IP	0.86±0.08	1.03±0.05	0.84
C2_94051	Angiotensin-converting enzyme	ACE	0.63±0.13	0.35±0.04	1.79
C2_49201	Angiotensin-converting enzyme	ACE	0.6±0.14	0.27±0.03	2.18
C2_31056	Angiotensin-converting enzyme	ACE	0.62±0.13	0.28±0.04	2.21
C2_2687	Angiotensin-converting enzyme	ACE	0.67±0.12	0.27±0.03	2.50
C2_1538	Angiotensin-converting enzyme 2	ACE2	0.58±0.15	0.26±0.03	2.22
C2_89317	Angiotensin-converting enzyme 2	ACE2	0.48±0.17	0.19±0.03	2.48
C2_17965	Angiotensinogen	AGT	0.88±0.08	1.3±0.29	0.67
C2_1919	Anionic trypsin-2	PRSS2	0.52±0.17	0.29±0.08	1.79
C2_98249	Ankyrin repeat and FYVE domain-containing protein 1	ANKFY1	1.03±0.07	1.95±0.42	0.53
C2_5024	Ankyrin repeat and FYVE domain-containing protein 1	ANKFY1	1.24±0.13	1.65±0.04	0.75
C2_6506	Ankyrin repeat and SAM domain-containing protein 4B	ANKS4B	1.31±0.19	2.26±0.12	0.58
C3_c47959	Ankyrin-3	ANK3	0.83±0.07	1.15±0.21	0.72
C2_1550	Annexin A1	ANXA1	0.79±0.09	0.59±0.06	1.33
C2_2096	Annexin A11	ANXA11	1.04±0.07	0.65±0.05	1.61
C2_3972	Annexin A13	ANXA13	1.31±0.18	0.48±0.03	2.74
C2_2251	Annexin A2	ANXA2	0.92±0.08	0.73±0.17	1.27
C2_5883	Annexin A2-A	ANXA2	3.32±1.06	1.47±0.07	2.27
C2_930	Annexin A3	ANXA3	1.39±0.2	1.24±0.09	1.12
C2_88	Annexin A4	ANXA4	1.09±0.17	0.36±0.04	3.05
C2_772	Annexin A5	ANXA5	1.22±0.12	0.68±0.08	1.79
C2_4250	Annexin A6	ANXA6	1.23±0.14	0.65±0.05	1.89
C2_4218	Anterior gradient protein 2 homolog	AGR2	1.78±0.35	1.21±0.13	1.47
C3_c16373	Antithrombin-III	SERPINC1	1.04±0.04	1.03±0.14	1.01
C2_11830	AP-1 complex subunit beta-1	AP1B1	1.21±0.09	2.21±0.17	0.55
C2_28545	AP-1 complex subunit beta-1	AP1B1	0.95±0.03	1.19±0.05	0.80
C2_37278	AP-1 complex subunit beta-1	AP1B1	1.27±0.09	1.2±0.02	1.06
C2_8787	AP-1 complex subunit gamma-1	AP1G1	1.23±0.12	1.54±0.02	0.80
C2_18258	AP-1 complex subunit gamma-like 2	AP1G2	0.95±0.13	1.01±0.11	0.94
C2_2248	AP-1 complex subunit mu-2	AP1M2	1.1±0.07	1.39±0.19	0.79
C2_9477	AP-1 complex subunit sigma-1A	AP1S1	0.82±0.08	1.16±0.06	0.71
C2_82848	AP-2 complex subunit alpha-1	AP2A1	1±0.05	1.15±0.07	0.87
C2_13693	AP-2 complex subunit alpha-1	AP2A1	1.27±0.09	1.37±0.09	0.93
C2_29524	AP-2 complex subunit alpha-2	AP2A2	0.82±0.07	3.02±0.77	0.27
C2_12233	AP-2 complex subunit alpha-2	AP2A2	0.8±0.08	1.18±0.21	0.68
C2_5784	AP-2 complex subunit beta	Ap2b1	0.98±0.11	0.73±0.06	1.34
C2_1474	AP-2 complex subunit mu-1-A	AP2M1	1.08±0.07	1.47±0.02	0.74
C2_678	AP-2 complex subunit sigma	AP2S1	1.12±0.09	0.99±0.06	1.14
C2_4970	AP-3 complex subunit delta-1	AP3D1	1±0.03	1.51±0.17	0.66
C2_5231	AP-3 complex subunit delta-1	AP3D1	0.75±0.12	0.64±0.05	1.17
C2_79275	AP-3 complex subunit delta-1	AP3D1	1.15±0.15	0.7±0.1	1.63
C2_11663	AP-3 complex subunit mu-1	AP3M1	0.97±0.05	0.99±0.05	0.98
C2_31643	AP-3 complex subunit mu-2	AP3M2	1.13±0.11	1.94±0.11	0.58
C2_6208	Apical endosomal glycoprotein	MAMDC4	0.77±0.11	0.46±0.02	1.68
C3_c37563	APOBEC1 complementation factor	A1CF	0.88±0.05	1.18±0.21	0.75
C2_120884	Apolipoprotein A-I	APOA1	3.29±1.44	9.39±2.53	0.35
C2_4177	Apolipoprotein A-I-binding protein	APOA1	1.01±0.05	0.98±0.06	1.03
C2_23243	Apolipoprotein A-IV	APOA4	1.61±0.63	1.85±1.01	0.87
C3_lrc60236	apolipoprotein A-IV-like isoform X1 [Maylandia zebra]	APOA4	1.57±0.43	3.27±1.33	0.48
s_rl0001e11_f_1	Apolipoprotein B-100	APOB	0.77±0.1	0.7±0.1	1.10

C3_c28587	Apolipoprotein B-100	APOB	0.95±0.06	0.79±0.07	1.21
AM973748	Apolipoprotein B-100	APOB	1.68±0.27	1.13±0.2	1.48
C2_15164	Apolipoprotein B-100	APOB	1.28±0.14	0.84±0.1	1.53
C2_5614	Apolipoprotein B-100	APOB	1.31±0.17	0.54±0.07	2.42
C2_28992	Apolipoprotein B-100	APOB	0.88±0.17	0.23±0.02	3.85
C3_c2363	apolipoprotein B-100-like [Oreochromis niloticus]	APOB	0.8±0.1	0.77±0.17	1.03
C2_5591	Apolipoprotein Eb	APOEB	1.12±0.18	1.93±0.64	0.58
C2_28734	Apolipoprotein M	APOM	1.4±0.21	1.91±0.35	0.73
C2_9385	Apoptosis inhibitor 5	API5	0.97±0.02	2.2±0.72	0.44
C2_322	Apoptosis-associated speck-like protein containing a CARD	PYCARD	1.55±0.49	4.09±0.51	0.38
C2_1626	Apoptosis-associated speck-like protein containing a CARD	PYCARD	0.8±0.08	1.55±0.25	0.51
C2_6260	Apoptosis-inducing factor 1, mitochondrial	AIFM1	1.36±0.13	0.75±0.04	1.81
C2_6887	Apoptosis-inducing factor 3	AIFM3	0.94±0.06	1.32±0.09	0.71
C2_19566	Apoptotic protease-activating factor 1	APAF1	1.83±0.32	1.55±0.08	1.18
C2_489	Arachidonate 12-lipoxygenase, 12R-type	ALOX12B	0.8±0.09	0.86±0.13	0.93
C2_1821	Arfaptin-1	ARFIP1	0.74±0.1	1.07±0.11	0.69
C2_14892	Arf-GAP with SH3 domain, ANK repeat and PH domain-containing protein 2	ASAP2	1.1±0.16	2.53±0.35	0.43
C2_19914	Arginyl-tRNA synthetase, cytoplasmic	RARS	1.18±0.1	1.93±0.24	0.62
C2_69580	Arginyl-tRNA synthetase, cytoplasmic	RARS	1.05±0.07	1.55±0.15	0.68
C2_29167	Aromatic-L-amino-acid decarboxylase	DDC	0.87±0.05	1.24±0.07	0.70
C2_43142	Arrestin domain-containing protein 1	ARRDC1	0.73±0.11	0.6±0.07	1.21
C2_857	Arsenite methyltransferase	AS3MT	2.15±0.57	1.71±0.31	1.26
C2_3422	Arylamine N-acetyltransferase, pineal gland isozyme NAT-10	NAT1	1.26±0.09	2.29±0.42	0.55
C2_43004	Arylsulfatase B	ARSB	0.88±0.11	1.01±0.15	0.87
C2_3390	Asparaginyl-tRNA synthetase, cytoplasmic	NARS	1.24±0.1	1.4±0.09	0.88
C2_14096	Aspartate aminotransferase, cytoplasmic	GOT1	0.73±0.1	0.51±0.05	1.44
C2_645	Aspartate aminotransferase, mitochondrial	GOT2	0.88±0.15	0.37±0.04	2.37
C2_4570	Aspartoacylase	ASPA	0.75±0.09	0.58±0.05	1.29
C2_1059	Aspartoacylase-2B	ACY3	0.85±0.08	0.67±0.04	1.27
C2_15853	Aspartyl aminopeptidase	DNPEP	0.7±0.16	0.28±0.03	2.54
C2_2153	Aspartyl-tRNA synthetase, cytoplasmic	ARSA	1.16±0.1	1.91±0.79	0.61
C3_c41038	Astrocytic phosphoprotein PEA-15	PEA15	0.73±0.11	0.63±0.06	1.15
C2_23566	Atlastin-3	ATL3	0.75±0.08	0.45±0.03	1.68
C2_736	ATP synthase lipid-binding protein, mitochondrial	ARSA	1.52±0.31	0.47±0.1	3.23
C2_1751	ATP synthase subunit alpha, mitochondrial	ATP5A1	1.32±0.25	0.82±0.03	1.62
C2_176	ATP synthase subunit b, mitochondrial	ATP5A1	1.18±0.14	0.71±0.07	1.65
C2_1973	ATP synthase subunit beta, mitochondrial	ATP5B	1.45±0.3	1±0.08	1.45
C2_939	ATP synthase subunit gamma, mitochondrial	ATP5C1	2.17±0.64	1.1±0.07	1.97
C2_2391	ATPase asna1	ASNA1	1.41±0.22	1.65±0.14	0.85
C2_7005	ATP-binding cassette sub-family D member 3	ABCD3	2.43±0.6	0.57±0.05	4.23
C2_2323	ATP-binding cassette sub-family E member 1	ABCE1	1.13±0.05	1.29±0.03	0.88
C2_1676	ATP-binding cassette sub-family F member 1	ABCF1	1.49±0.24	1.09±0.15	1.37
C2_8958	ATP-citrate synthase	ACLY	1.52±0.21	1.66±0.14	0.92
C3_c19506	ATP-citrate synthase	ACLY	1.12±0.07	0.64±0.05	1.74
C2_9938	ATP-dependent RNA helicase DDX18	DDX18	1±0.04	1.18±0.09	0.85
C2_1283	ATP-dependent RNA helicase DDX39A	DDX39A	1.27±0.17	2.3±0.39	0.55
C2_20811	ATP-dependent RNA helicase DDX3Y	DDX3Y	1.36±0.2	2±0.28	0.68
C2_94749	ATP-dependent RNA helicase DDX42	DDX42	0.79±0.14	0.62±0.09	1.29
C2_5878	Autophagy-related protein 16-1	ATG16L1	0.93±0.03	0.82±0.04	1.14
C2_214	Bactericidal permeability-increasing protein	BPI	0.89±0.09	0.93±0.19	0.96
C2_5274	Baculoviral IAP repeat-containing protein 4	XIAP	0.92±0.1	0.5±0.06	1.83
C2_6477	Band 4.1-like protein 3	EPB41L3	1.14±0.08	1.45±0.08	0.79
C2_18157	Band 4.1-like protein 5	EPB41L5	1.06±0.1	1.27±0.12	0.83
C2_111896	Band 7 protein AAEL010189	AAEL010189	1.13±0.27	0.45±0.03	2.50
C2_1409	Barrier-to-autointegration factor	BANF1	1.06±0.09	0.58±0.1	1.82
C2_10232	Barrier-to-autointegration factor-like protein	BANF2	1.03±0.07	0.6±0.07	1.71
C2_871	Basic leucine zipper and W2 domain-containing protein 1-A	BZW1	1.11±0.05	1.12±0.06	0.99
C2_8263	Beta,beta-carotene 15,15'-monooxygenase	BCO1	1.44±0.15	0.94±0.03	1.53
C2_7258	Beta,beta-carotene 9',10'-oxygenase	BCO2	1.14±0.07	1.42±0.23	0.81
C2_28	Beta-2-microglobulin	B2M	1.24±0.13	0.85±0.04	1.46
C2_19834	Beta-actin-like protein 2	ACTBL2	1.07±0.03	2.95±0.57	0.36
C2_69899	Beta-centractin	ACTR1B	1.03±0.07	1.49±0.17	0.69
C2_370	Beta-enolase	ENO3	0.89±0.04	0.49±0.03	1.80
C2_2136	Beta-galactosidase	GLB1	1.04±0.07	1.51±0.14	0.69
C2_26906	Beta-galactosidase-1-like protein 2	GLB1L2	1.02±0.08	0.72±0.01	1.41
C2_14188	Beta-galactosidase-1-like protein 2	GLB1L2	0.99±0.03	0.64±0.04	1.55

C2_68077	Beta-galactosidase-1-like protein 3	GLB1L3	0.85±0.06	0.66±0.07	1.30
C2_8457	Beta-glucuronidase	GUSB	0.75±0.14	0.27±0.03	2.81
C2_3871	Beta-hexosaminidase subunit beta	HEXB	0.78±0.14	0.57±0.12	1.36
C2_43304	Betaine--homocysteine S-methyltransferase 1	BHMT	0.72±0.1	0.85±0.12	0.85
C2_110871	Betaine--homocysteine S-methyltransferase 1	BHMT	1.22±0.31	0.26±0.02	4.64
C2_79951	Betaine--homocysteine S-methyltransferase 2	BHMT	0.84±0.08	0.23±0.02	3.64
C2_5459	Beta-ureidopropionase	UPB1	0.78±0.12	0.44±0.04	1.76
C2_3176	Bifunctional 3'-phosphoadenosine 5'-phosphosulfate synthase 2	PAPSS2	0.94±0.03	2.52±0.72	0.37
C2_48204	Bifunctional aminoacyl-tRNA synthetase	EPRS	1.29±0.14	1.98±0.06	0.65
C2_4884	Bifunctional aminoacyl-tRNA synthetase	EPRS	1.24±0.14	1.71±0.12	0.73
C2_6394	Bifunctional polynucleotide phosphatase/kinase	PNKP	0.91±0.09	1.05±0.22	0.87
C2_4731	Bifunctional protein NCOAT	MGEA5	1.14±0.08	2.44±0.47	0.47
C2_4254	Bifunctional purine biosynthesis protein PURH	ATIC	0.93±0.03	1.17±0.06	0.79
C2_20039	Bifunctional UDP-N-acetylglucosamine 2-epimerase/N-acetylmannosamine kinase	GNE	0.53±0.16	0.21±0.02	2.48
C2_73027	Bile salt-activated lipase	CEL	0.65±0.12	0.36±0.01	1.82
C2_1589	Biliverdin reductase A	BLVRA	1.03±0.03	1.03±0.06	1.01
C2_1056	Biogenesis of lysosome-related organelles complex 1 subunit 1	BLOC1S1	0.93±0.11	1.09±0.11	0.86
C2_9034	Biogenesis of lysosome-related organelles complex 1 subunit 2	BLOC1S2	0.97±0.05	1.5±0.1	0.65
C2_29998	Biotinidase	BTD	0.93±0.04	0.81±0.11	1.14
C2_17007	Bis(5'-nucleosyl)-tetraphosphatase [asymmetrical]	NUDT2	1.4±0.14	1.73±0.04	0.81
C2_885	Bleomycin hydrolase	BLMH	0.76±0.1	0.46±0.05	1.67
C2_4365	BolA-like protein 1	BOLA1	0.79±0.12	0.78±0.13	1.01
C2_15564	Brain-specific angiogenesis inhibitor 1-associated protein 2-like protein 2	BAIAP2L2	0.71±0.1	1.11±0.15	0.64
C2_1593	BRCA1-A complex subunit BRE	BRE	1.06±0.05	1.16±0.08	0.91
C2_692	BRCA2 and CDKN1A-interacting protein	BCCIP	1±0.05	1.06±0.07	0.94
C2_24767	Breast cancer anti-estrogen resistance protein 1	BCAR1	0.89±0.05	0.94±0.08	0.95
C2_79857	Breast cancer anti-estrogen resistance protein 3	BCAR3	1.01±0.04	0.95±0.06	1.06
C3_c10749	Brefeldin A-inhibited guanine nucleotide-exchange protein 1	ARFGEF1	1.96±0.41	1.6±0.19	1.23
C2_11359	Bridging integrator 2	BIN2	2.54±0.98	13.08±3.54	0.19
C2_14149	BRISC and BRCA1-A complex member 1	BABAM1	1.04±0.03	1.31±0.05	0.79
C2_65091	Butyrophilin subfamily 2 member A3	BTN3A3	1.16±0.16	0.72±0.05	1.62
C2_27587	Butyrophilin-like protein 2	BTNL2	0.93±0.13	0.25±0.03	3.68
C2_111124	Butyrophilin-like protein 8	BTNL8	1.32±0.22	0.52±0.05	2.55
C2_22710	CAAX prenyl protease 1 homolog	ZMPSTE24	0.98±0.08	0.58±0.03	1.71
C2_5533	CAAX prenyl protease 1 homolog	ZMPSTE24	0.99±0.14	0.27±0.01	3.66
C2_48101	Cadherin EGF LAG seven-pass G-type receptor 1	CELSR1	0.66±0.11	0.65±0.07	1.03
C2_2045	Cadherin-1	CDH1	0.91±0.1	0.6±0.06	1.51
C2_3038	Cadherin-17	CDH17	0.9±0.14	0.33±0.02	2.73
C2_20741	Cadherin-23	CDH23	0.49±0.17	0.39±0.03	1.28
C2_116735	Cadherin-89D	CAD89D	0.65±0.12	0.61±0.05	1.08
C2_70214	Cadherin-related family member 2	CDHR2	0.59±0.14	0.53±0.06	1.11
C2_5783	Cadherin-related family member 2	CDHR2	0.81±0.09	0.64±0.07	1.26
C2_25329	Cadherin-related family member 2	CDHR2	0.58±0.14	0.42±0.04	1.38
C2_23986	Calcineurin subunit B type 1	PPP3R1	1.46±0.26	1.38±0.01	1.06
C2_33603	Calcium/calmodulin-dependent protein kinase type 1	CAMK1	0.99±0.07	2.35±0.32	0.42
C2_54503	Calcium/calmodulin-dependent protein kinase type II delta chain	CAMK2D	0.9±0.04	0.68±0.07	1.32
C2_22563	Calcium/calmodulin-dependent protein kinase type II subunit gamma	CAMK2G	1.42±0.3	2.18±0.3	0.65
C2_14650	Calcium-binding protein 39	CAB39	1.1±0.1	1.6±0.12	0.69
C2_3736	Calcium-binding protein 39-like	CAB39L	1.5±0.24	1.84±0.23	0.81
C2_1929	Calcium-binding protein p22	CHP1	0.98±0.13	0.33±0.03	3.02
C2_1095	Calcyclin-binding protein	CACYBP	0.92±0.05	0.61±0.08	1.49
C2_103184	Calmodulin	CALM1	1.62±0.33	1.71±0.06	0.95
C2_98055	Calmodulin-like protein 4	CALML4	1.13±0.22	1.27±0.19	0.89
C2_19770	Calnexin	CANX	1.53±0.23	0.5±0.05	3.08
C2_5497	Calpain small subunit 1	CAPNS1	0.99±0.07	1.26±0.07	0.79
C2_7535	Calpain small subunit 1	CAPNS1	1.02±0.1	1.21±0.03	0.84
C2_15607	Calpain-1 catalytic subunit	CAPN1	0.68±0.13	0.64±0.1	1.06
C2_4520	Calpain-2 catalytic subunit	CAPN2	1.18±0.06	1.15±0.01	1.02
C3_c11665	Calpain-5	CAPN5	1.4±0.45	1.46±0.21	0.96
C2_61779	Calpain-9	CAPN9	1.17±0.09	2.03±0.19	0.58
C2_12340	Calpastatin	CAST	1.21±0.21	2.18±0.37	0.56
C2_1023	Calreticulin	CALR	2.78±0.7	1.38±0.05	2.01
C2_15813	cAMP-dependent protein kinase catalytic subunit alpha	PRKACA	0.83±0.07	0.95±0.04	0.87
C2_6010	cAMP-dependent protein kinase type II-alpha regulatory subunit	PRKAR2A	0.93±0.04	1.36±0.15	0.68

C2_45801	Caprin-2	CAPRIN2	1.34±0.16	1.34±0.32	1.00
C2_2482	Carbohydrate kinase domain-containing protein	CARKD	0.97±0.07	0.61±0.03	1.59
C2_1572	Carbonic anhydrase	CA13	0.86±0.12	0.78±0.08	1.10
C2_1891	Carbonic anhydrase 1	CA1	1.45±0.19	1.61±0.17	0.90
C2_15533	Carbonic anhydrase 5B, mitochondrial	CA5B	0.86±0.07	1.18±0.15	0.73
C2_14726	Carbonyl reductase [NADPH] 1	CBR1	1.28±0.19	1.79±0.26	0.71
C2_32418	Carboxylesterase 5A	CES5A	1.05±0.08	0.87±0.07	1.21
C2_1812	Carboxymethylenebutenolidase homolog	CMBL	0.68±0.13	0.35±0.04	1.95
C2_936	Carboxypeptidase A1	CPA1	0.74±0.2	0.32±0.08	2.31
C2_5650	Carboxypeptidase A2	CPA2	0.73±0.14	0.41±0.07	1.77
C2_2018	Carboxypeptidase B	CPB1	0.8±0.18	0.36±0.07	2.20
C2_79368	Carboxypeptidase N subunit 2	CPN2	1.08±0.13	0.9±0.12	1.20
C2_4458	Carboxypeptidase O	CPO	0.61±0.13	0.28±0.04	2.16
C2_30675	Carcinoembryonic antigen-related cell adhesion molecule 1	CEACAM1	0.82±0.07	0.83±0.12	0.98
C2_29179	Carcinoembryonic antigen-related cell adhesion molecule 5	Ceacam5	0.79±0.13	0.48±0.08	1.66
C2_10090	Carcinoembryonic antigen-related cell adhesion molecule 6	CEACAM6	0.76±0.09	0.6±0.08	1.26
C2_6038	Carnitine O-acetyltransferase	CRAT	1.31±0.11	0.93±0.05	1.40
C2_4920	Carnitine O-palmitoyltransferase 1, liver isoform	CPT1A	1.52±0.29	0.62±0.1	2.45
C2_2166	Carnitine O-palmitoyltransferase 2, mitochondrial	CPT2	1.67±0.36	0.82±0.1	2.04
C2_7099	Casein kinase I isoform alpha	CSNK1A1	0.79±0.08	1.05±0.15	0.75
C2_540	Casein kinase II subunit alpha	CSNK2A1	1.05±0.05	0.79±0.07	1.33
C2_8203	Casein kinase II subunit beta	CSNK2B	0.87±0.08	0.54±0.05	1.61
C2_16670	Caspase-1	CASP1	1.13±0.12	1.86±0.36	0.61
C2_85583	Caspase-1-A	CASP1	0.87±0.1	0.81±0.17	1.07
C2_3535	Caspase-3	CASP3	0.93±0.03	0.79±0.04	1.18
C2_5709	Caspase-6	CASP6	1.07±0.05	1.54±0.12	0.69
C2_4205	Caspase-8	CASP8	0.91±0.07	0.96±0.11	0.95
C2_997	Catalase	CAT	1.91±0.36	0.85±0.05	2.25
C2_872	Catechol O-methyltransferase domain-containing protein 1	COMTD1	0.75±0.14	0.44±0.03	1.72
C2_13472	Catenin alpha-1	CTNNA1	1.35±0.18	2.82±0.23	0.48
C2_78432	Catenin alpha-1	CTNNA1	0.79±0.11	0.4±0.04	2.01
C2_25912	Catenin beta-1	CTNNB1	1.11±0.05	1.78±0.17	0.63
C2_23957	Catenin delta-1	CTNND1	1.06±0.04	1.38±0.26	0.77
C2_11960	Cathepsin D	CTSD	0.68±0.15	0.31±0.03	2.21
C2_21	Cathepsin L	CTSL	0.72±0.09	4.16±1.07	0.17
C2_108360	Cathepsin S	CTSS	0.77±0.11	0.44±0.05	1.74
C2_1045	Cathepsin Z	CTSZ	0.62±0.16	0.56±0.13	1.11
C2_6981	Cation-indipendent mannose-6-phosphate receptor	IGF2R	1.18±0.22	0.51±0.06	2.32
C2_67046	CCR4-NOT transcription complex subunit 1	CNOT1	0.92±0.08	1.03±0.16	0.89
C2_7739	CCR4-NOT transcription complex subunit 1	CNOT1	0.86±0.09	0.96±0.02	0.90
C2_21127	CCR4-NOT transcription complex subunit 1	CNOT1	1.26±0.18	1.19±0.06	1.06
C2_14565	CCR4-NOT transcription complex subunit 10	CNOT10	0.65±0.12	0.22±0.04	3.03
C2_10479	CCR4-NOT transcription complex subunit 6	CNOT6	1.25±0.2	1.54±0.07	0.81
C2_9606	CD2-associated protein	CD2AP	0.91±0.15	1.44±0.35	0.63
C2_211	CDGSH iron-sulfur domain-containing protein 1	CISD1	0.9±0.06	0.55±0.08	1.63
C2_6264	CDK5 regulatory subunit-associated protein 3	CDK5RAP3	0.94±0.02	0.83±0.04	1.14
C2_2412	Cell differentiation protein RCD1 homolog	RQCD1	1.34±0.22	1.78±0.06	0.75
C2_99597	Cell division control protein 42	CDK1	1.22±0.08	1.6±0.12	0.76
C2_1548	Cell division control protein 42 homolog	Cdc42	0.98±0.06	1.1±0.11	0.89
C2_12428	Cell growth regulator with EF hand domain protein 1	CGREF1	1.27±0.11	0.93±0.03	1.36
C2_2533	Centromere protein V	CENPV	1.77±0.28	1.5±0.08	1.18
C2_39986	Ceruloplasmin	CP	0.77±0.08	0.76±0.08	1.02
C2_19937	C-factor	CSGA	1.11±0.06	0.45±0.02	2.48
C2_12260	cGMP-dependent protein kinase 2	PRKG1	1.06±0.07	1.57±0.34	0.68
C2_3278	Chitin synthase 1	CHS1	0.83±0.1	0.98±0.11	0.84
C2_45714	Chloride intracellular channel protein 2	CLIC2	1.29±0.1	4.45±0.33	0.29
C2_1437	Chloride intracellular channel protein 4	CLIC4	1.27±0.2	2.76±0.07	0.46
C2_7684	Chloride intracellular channel protein 5	CLIC5	1.5±0.2	3.62±0.25	0.41
C2_3135	Cholesterol 24-hydroxylase	CYP46A1	1.18±0.09	0.69±0.05	1.72
C2_5331	Choline kinase alpha	CHKA	0.81±0.12	0.21±0.01	3.86
C2_6485	Choline transporter-like protein 4	SLC44A4	1.56±0.22	3.4±0.2	0.46
C2_7664	Cholinesterase	BCHE	0.73±0.1	0.55±0.02	1.34
C2_19026	Chromodomain-helicase-DNA-binding protein 1-like	CHD1L	0.85±0.07	0.66±0.02	1.29
C2_12123	Chymotrypsin A	CTRB1	0.62±0.15	0.36±0.06	1.74
C2_4647	Chymotrypsin B	CTRB2	0.57±0.18	0.23±0.07	2.44
C2_8139	Chymotrypsin-C	CTRC	0.56±0.17	0.27±0.05	2.10
C2_16147	Chymotrypsin-like elastase family member 1	CELA1	0.69±0.11	0.48±0.03	1.43

C2_9094	Chymotrypsin-like elastase family member 2A	CELA2A	0.5±0.18	0.29±0.05	1.76
C2_6507	Chymotrypsin-like elastase family member 3B	CELA3B	0.56±0.17	0.33±0.07	1.73
C2_4909	Chymotrypsin-like protease CTRL-1	CTRL	0.49±0.18	0.2±0.04	2.44
C2_19864	Cingulin	CGN	6.54±2.75	14.82±2.09	0.44
C2_2740	Citrate synthase, mitochondrial	CS	0.93±0.04	0.3±0.03	3.08
C2_121654	Class I histocompatibility antigen, F10 alpha chain	HLA-A	1.03±0.07	0.63±0.05	1.63
C2_18775	Clathrin heavy chain 1	CLTC	1.4±0.17	5.47±0.45	0.26
C2_13187	Clathrin heavy chain 1	CLTC	1.03±0.03	2.84±0.26	0.36
C2_49445	Clathrin heavy chain 1	CLTC	1.17±0.07	2.12±0.13	0.55
C2_15910	Clathrin heavy chain 1	CLTC	1.56±0.22	2.25±0.16	0.70
C2_19700	Clathrin interactor 1	CLINT1	1.06±0.04	1.67±0.08	0.64
C2_1221	Clathrin light chain A	CLTA	1.29±0.33	1.27±0.27	1.01
C2_14161	Cleavage and polyadenylation specificity factor subunit 1	CPSF1	0.7±0.11	0.43±0.08	1.64
C2_1094	Cleavage and polyadenylation specificity factor subunit 5	NUDT21	1.38±0.17	1.4±0.03	0.99
C3_c49530	cleavage stimulation factor subunit 2-like [Oreochromis niloticus]	CSTF2	1.87±0.48	1.44±0.41	1.30
C2_25025	Cleft lip and palate transmembrane protein 1-like protein	CLPTM1L	3.81±1.1	2.07±0.33	1.84
C2_76527	Coactosin-like protein	COTL1	1.03±0.11	2.25±0.51	0.46
C2_28225	Coagulation factor IX	F9	0.89±0.05	0.91±0.2	0.98
C2_17519	Coagulation factor XI	F11	1.33±0.12	1.14±0.09	1.16
C2_66184	Coatomer subunit alpha	COPA	0.94±0.06	1.37±0.06	0.69
C2_47987	Coatomer subunit alpha	COPA	1.61±0.34	1.67±0.14	0.97
C2_5592	Coatomer subunit alpha	COPA	1.59±0.31	1.59±0.05	1.00
C2_27574	Coatomer subunit beta	COPB1	1.41±0.21	2.01±0.26	0.70
C2_4111	Coatomer subunit beta	COPB1	1.46±0.19	1.59±0.14	0.92
C2_6399	Coatomer subunit beta'	COPB2	1.37±0.14	1.33±0.03	1.03
C2_5687	Coatomer subunit delta	ARCN1	1.08±0.04	1.54±0.11	0.70
C2_191	Coatomer subunit epsilon	COPE	1.26±0.19	1.27±0.05	0.99
C2_3555	Coatomer subunit gamma-2	COPG2	1.02±0.04	0.99±0.08	1.03
C2_157	Coatomer subunit zeta-1	COPZ1	0.82±0.1	0.98±0.08	0.84
C2_120873	Cocaine esterase	CES2	0.79±0.07	0.66±0.1	1.19
C2_1192	Cofilin-2	CFL2	1.32±0.19	4.32±1.07	0.31
C2_3194	Cohesin subunit SA-2	STAG2	1.1±0.18	1.13±0.1	0.98
C2_7339	Coiled-coil domain-containing protein 25	CCDC25	1.01±0	0.89±0.07	1.13
C2_2941	Coiled-coil domain-containing protein 56	FYCO1	0.79±0.16	0.32±0.06	2.46
C2_64770	Coiled-coil domain-containing protein 93	CCDC93	1.04±0.03	1.63±0.21	0.64
C2_36566	Cold-inducible RNA-binding protein A	CIRBP	0.8±0.08	1.29±0.33	0.62
C2_6346	Collagen alpha-1(VIII) chain	COL8A1	0.69±0.11	0.56±0.06	1.22
C2_47133	Collagen alpha-1(XII) chain	COL12A1	0.76±0.1	1.22±0.56	0.63
C2_54796	Collagen alpha-2(VIII) chain	COL8A2	0.99±0.05	1.05±0.14	0.94
C2_9466	Collagen alpha-3(VI) chain	COL6A3	0.99±0.09	1.12±0.08	0.88
C2_25181	Collagen alpha-3(VI) chain	COL6A3	0.95±0.06	1.05±0.07	0.91
C2_18814	Collagen alpha-3(VI) chain	COL6A3	0.76±0.09	0.62±0.03	1.22
C3_c35929	Collagen alpha-3(VI) chain	COL6A3	0.72±0.1	0.48±0.04	1.48
C2_6735	Collagen type IV alpha-3-binding protein	COL4A3BP	1.12±0.09	1.07±0.07	1.04
C2_15503	Collectin-12	COLEC12	0.96±0.09	0.9±0.19	1.06
C2_3111	Collectrin	TMEM27	0.9±0.04	1.23±0.06	0.73
C2_481	COMM domain-containing protein 2	COMMD2	1.04±0.02	1.85±0.32	0.56
C2_1106	COMM domain-containing protein 4	COMMD4	0.88±0.04	1.4±0.18	0.63
C2_6850	COMM domain-containing protein 5	COMMD5	1.1±0.06	1.46±0.09	0.75
C2_1725	COMM domain-containing protein 7	COMMD7	1.07±0.04	1.34±0.08	0.80
C2_9863	COMM domain-containing protein 9	COMMD9	0.92±0.05	1.27±0.09	0.73
C2_2765	Complement C1q tumor necrosis factor-related protein 3	C1QTNF3	0.74±0.09	1.27±0.1	0.58
C2_107825	Complement C1q-like protein 2	C1QL2	0.74±0.12	0.56±0.07	1.31
C2_9268	Complement C1q-like protein 4	C1QL4	0.57±0.15	0.94±0.22	0.60
C2_34468	Complement C1r-A subcomponent	C1R	0.89±0.06	0.73±0.05	1.22
s_flp0005h11_f_1	Complement C2	C2	1.31±0.38	3.14±0.49	0.42
FP333165	Complement C2	C2	1.09±0.06	1.21±0.06	0.89
C2_1398	Complement C3	C3	1.14±0.22	1.46±0.3	0.79
C2_23904	Complement C4-B	C4A/C4B	0.81±0.07	0.99±0.11	0.82
C3_c8707	Complement C5	C5	0.9±0.04	0.69±0.09	1.30
C2_2762	Complement component 1 Q subcomponent-binding protein, mitochondrial	C1QBP	1.27±0.23	0.83±0.09	1.53
C2_2093	Complement component C6	C6	1.24±0.23	2.31±0.78	0.54
C2_39232	Complement component C7	C7	0.97±0.01	0.78±0.12	1.25
C2_26343	Complement component C9	C9	0.65±0.13	1.05±0.36	0.61
C2_119654	Complement decay-accelerating factor, GPI-anchored	CD55	1.6±0.28	1.6±0.26	1.00
C2_9760	Complement factor B	CFB	1.45±0.27	1.98±0.43	0.73

C2_121665	Complement factor D	CFD	1.14±0.06	1±0.1	1.14
C2_2374	Complement factor H	CFH	1.06±0.1	1.2±0.12	0.88
C2_8397	Complement factor I	CFI	0.86±0.08	0.86±0.02	1.00
C2_1232	COP9 signalosome complex subunit 1	GPS1	0.99±0.06	1.27±0.03	0.78
C2_574	COP9 signalosome complex subunit 2	Cops2	1.17±0.11	1.66±0.11	0.71
C2_1948	COP9 signalosome complex subunit 3	COPS3	1.15±0.06	1.36±0.11	0.84
C2_100934	COP9 signalosome complex subunit 4	COPS4	1.03±0.08	1.16±0.1	0.88
C2_1203	COP9 signalosome complex subunit 5	COPS5	1.16±0.08	1.55±0.01	0.75
C2_3742	COP9 signalosome complex subunit 6	COPS6	1.03±0.03	1.43±0.11	0.72
C2_2034	COP9 signalosome complex subunit 8	COPS8	1.13±0.08	1.34±0.27	0.84
C2_2060	Copine-1	CPNE1	1.07±0.08	1.03±0.05	1.04
C2_6548	Copine-1	CPNE1	1.01±0.09	0.67±0.05	1.51
C2_2684	Copine-3	CPNE3	0.98±0.08	0.67±0.06	1.45
C2_9214	Copper chaperone for superoxide dismutase	CCS	1.46±0.25	2.45±0.09	0.60
C2_3222	Copper homeostasis protein cutC homolog	CUTC	0.82±0.06	0.85±0.06	0.97
C2_113919	Copper transport protein ATOX1	ATOX1	1.27±0.21	4.76±0.65	0.27
C2_2051	Coproporphyrinogen-III oxidase, mitochondrial	CPOX	1.01±0.12	0.66±0.08	1.52
C2_451	Coronin-1A	CORO1A	1.07±0.06	1.05±0.08	1.01
C2_5573	Coronin-7	CORO7	1.06±0.1	1.76±0.16	0.60
C2_2727	Costars family protein C6orf115 homolog	C6orf115	1.14±0.12	2.19±0.14	0.52
C2_12148	Coxsackievirus and adenovirus receptor homolog	CXADR	0.84±0.06	1.02±0.1	0.82
C2_690	Creatine kinase B-type	CKB	1.08±0.03	0.9±0.07	1.20
C2_27	Creatine kinase M-type	CKM	1.15±0.09	1.38±0.15	0.83
C2_2465	Creatine kinase S-type, mitochondrial	CKMT2	0.99±0.1	0.41±0.09	2.43
C2_24223	Creatine kinase U-type, mitochondrial	CKMT1A	0.88±0.13	0.66±0.06	1.33
C2_29254	Crk-like protein	CRKL	0.6±0.14	0.44±0.06	1.36
C2_1078	Crystallin J1C	CJ1C	1.01±0.07	1.06±0.17	0.96
C2_13761	Cubilin	CUBN	0.7±0.11	1.69±0.43	0.42
C2_24989	Cubilin	CUBN	0.97±0.11	1.43±0.34	0.68
C2_7016	CUGBP Elav-like family member 2	CELF2	0.85±0.06	0.87±0.07	0.98
C2_4406	Cullin 3	CUL3	1.16±0.06	0.45±0.02	2.61
C2_13502	Cullin 5	CUL5	1.08±0.11	2.11±0.43	0.51
C2_16199	Cullin-1	CUL1	1.06±0.05	1.42±0.16	0.75
C2_23133	Cyclin-G-associated kinase	GAK	0.63±0.16	0.51±0.03	1.25
C2_1820	Cystathionine beta-synthase	CBS	1.35±0.18	0.84±0.07	1.60
C2_2620	Cystathionine gamma-lyase	CTH	1.36±0.15	0.63±0.05	2.17
C2_6753	Cystatin	CST1	1.06±0.08	1.64±0.3	0.65
C2_617	Cystatin-B	CSTB	0.93±0.03	1.94±0.17	0.48
C2_483	Cysteine dioxygenase type 1	CDO1	1.05±0.06	0.86±0.06	1.22
C2_2752	Cysteine protease ATG4B	ATG4B	0.85±0.06	0.91±0.02	0.93
C2_9620	Cysteine sulfinic acid decarboxylase	CSAD	1.08±0.07	0.81±0.04	1.33
C2_42628	Cysteine-rich protein 1	CRIP1	2.54±0.72	4.13±0.37	0.62
C2_4479	Cysteinyl-tRNA synthetase, cytoplasmic	CARS	1.71±0.25	1.65±0.02	1.04
C2_34558	Cytidine and dCMP deaminase domain-containing protein 1	CDADC1	1±0.27	0.66±0.05	1.52
C2_12715	Cytochrome b5	CYB5A	0.78±0.11	0.21±0.02	3.78
C2_71561	Cytochrome b5 type B	CYB5B	1.05±0.09	0.61±0.04	1.72
C2_2189	Cytochrome c	LOC690675	0.98±0.12	0.84±0.11	1.17
C2_80	Cytochrome c oxidase subunit 2	MT-CO2	1.07±0.19	0.22±0.03	4.88
C2_462	Cytochrome c oxidase subunit 4 isoform 2, mitochondria	COX4I2	1.17±0.2	0.48±0.07	2.44
C2_785	Cytochrome c1, heme protein, mitochondrial	CYC1	2.69±0.96	0.77±0.04	3.51
C2_46314	Cytochrome c-b	CYCB	0.83±0.12	0.31±0.04	2.71
C2_7108	Cytochrome P450 20A1	CYP20A1	1.67±0.32	1.71±0.28	0.98
C2_36102	Cytochrome P450 2C23	Cyp2c44	1.03±0.07	1.12±0.31	0.92
C2_13986	Cytochrome P450 2D26	Cyp2d26	0.89±0.1	0.35±0.03	2.53
C2_4087	Cytochrome P450 2J2	CYP2J2	1.1±0.08	0.93±0.06	1.18
C2_4431	Cytochrome P450 2K1	CYP2K1	1.24±0.13	0.83±0.09	1.49
C2_90622	Cytochrome P450 2K3	CYP2K3	0.89±0.13	0.59±0.05	1.52
C2_57426	Cytochrome P450 2K3	CYP2K3	1.34±0.19	0.52±0.04	2.59
C2_2493	Cytochrome P450 3A40	CYP3A40	1.45±0.18	0.92±0.02	1.59
C2_10704	Cytoglobin-1	CYGB1	1.01±0.02	2.14±0.11	0.47
s_flp0006b09_f_1	Cytoplasmic aconitate hydratase	ACO1	1.28±0.1	2.18±0.5	0.59
FP333392	Cytoplasmic aconitate hydratase	ACO1	1.31±0.17	1.81±0.47	0.73
C2_68872	Cytoplasmic aconitate hydratase	ACO1	1.21±0.1	1.43±0.05	0.85
C2_2851	Cytoplasmic aconitate hydratase	ACO1	1.32±0.11	1.34±0.05	0.98
C2_49271	Cytoplasmic dynein 1 heavy chain 1	DYNC1H1	0.83±0.06	1.85±0.5	0.45
C2_39955	Cytoplasmic dynein 1 heavy chain 1	DYNC1H1	1.11±0.12	1.74±0.48	0.64
C2_50878	Cytoplasmic dynein 1 heavy chain 1	DYNC1H1	1.44±0.24	1.96±0.23	0.73

C2_5034	Cytoplasmic dynein 1 heavy chain 1	DYNC1H1	1.05±0.07	1.42±0.23	0.74
C2_16351	Cytoplasmic dynein 1 heavy chain 1	DYNC1H1	1.3±0.18	1.58±0.36	0.83
C2_82382	Cytoplasmic dynein 1 heavy chain 1	DYNC1H1	1.02±0.1	1.23±0.19	0.83
C2_96734	Cytoplasmic dynein 1 heavy chain 1	DYNC1H1	1.45±0.16	1.7±0.25	0.85
C2_7060	Cytoplasmic dynein 1 intermediate chain 2	Dync1i2	0.92±0.06	1.42±0.11	0.64
C2_2521	Cytoplasmic dynein 1 light intermediate chain 2	DYNC1LI2	1.11±0.07	0.95±0.31	1.17
C2_21796	Cytoplasmic FMR1-interacting protein 1 homolog	CYFIP1	1.56±0.26	2.65±0.43	0.59
C2_11304	Cytoplasmic FMR1-interacting protein 1 homolog	CYFIP1	1.3±0.45	1.47±0.28	0.89
C2_12482	Cytoskeleton-associated protein 5	CKAP5	0.97±0.07	1.11±0.15	0.88
C2_7146	Cytosol aminopeptidase	LAP3	0.89±0.05	0.45±0.04	1.98
C2_13504	Cytosolic 5'-nucleotidase 1B	NT5C1B	2.49±0.81	1.44±0.37	1.73
C2_12507	Cytosolic 5'-nucleotidase 3	NT5C3A	0.89±0.07	0.98±0.06	0.91
C2_3501	Cytosolic Fe-S cluster assembly factor narfl	NARFL	1±0.05	1.02±0.03	0.98
C2_928	Cytosolic non-specific dipeptidase	CNDP2	1.36±0.12	2.07±0.15	0.66
C2_10502	Cytosolic phospholipase A2 gamma	PLA2G4C	1.94±0.47	3.96±0.42	0.49
C2_32889	Cytosolic phospholipase A2 gamma	PLA2G4C	1.22±0.21	2.16±0.12	0.56
C2_5757	Cytosolic purine 5'-nucleotidase	NT5C2	0.84±0.09	0.32±0.03	2.60
C2_70910	Cytosolic sulfotransferase 1	SULT1A1	1.04±0.05	1.93±0.37	0.54
C2_528	Cytosolic sulfotransferase 2	SULT1A1	1.01±0.04	2.57±0.63	0.39
C2_2270	Cytosolic sulfotransferase 3	SULT1A1	1.09±0.12	3.56±0.61	0.31
C2_31660	D-2-hydroxyglutarate dehydrogenase, mitochondrial	D2HGDH	1.33±0.16	0.71±0.04	1.88
C2_7372	D-amino-acid oxidase	DAO	1.85±0.34	0.78±0.04	2.37
C2_2631	DCN1-like protein 2	DCUN1D2	1.1±0.11	1.57±0.13	0.70
C2_1763	D-dopachrome decarboxylase	DDT	0.92±0.05	1.2±0.11	0.77
C2_5628	DDRGK domain-containing protein 1	DDRGK1	1.03±0.05	1.89±0.3	0.54
C2_3999	Dehydrogenase/reductase SDR family member 1	DHRS1	1.35±0.21	0.85±0.09	1.58
C2_34491	Dehydrogenase/reductase SDR family member 11	DHRS11	0.83±0.12	0.58±0.06	1.44
C2_35778	Dehydrogenase/reductase SDR family member 12	DHRS12	1.37±0.14	1.18±0.1	1.16
C2_5726	Dehydrogenase/reductase SDR family member 13	DHRS13	1.19±0.15	0.54±0.02	2.21
C2_9536	Deleted in malignant brain tumors 1 protein	DMBT1	0.69±0.15	0.33±0.08	2.07
C2_12746	Delta(24)-sterol reductase	DHCR24	0.96±0.08	0.51±0.05	1.88
C2_17169	Delta-aminolevulinic acid dehydratase	ALAD	0.74±0.09	0.62±0.04	1.20
C2_734	DENN domain-containing protein 2D	DENND2D	0.82±0.08	0.78±0.12	1.05
C2_94566	Deoxyribonuclease-1	DNASE1	0.56±0.16	0.28±0.05	1.99
C2_19553	Deoxyribonuclease-2-alpha	DNASE2	1.38±0.36	0.95±0.05	1.46
C2_14136	Desmocollin-2	DSC2	0.92±0.05	0.84±0.08	1.10
C2_17928	Desmoplakin	DSP	0.86±0.05	1.15±0.17	0.75
C2_20476	Desmoplakin	DSP	1.19±0.09	1±0.07	1.19
C2_521	Diablo homolog, mitochondrial	DIABLO	1.3±0.21	2.32±0.21	0.56
C2_71353	Diacylglycerol kinase beta	DGKB	1.29±0.16	0.67±0.02	1.93
C2_14569	Diamine acetyltransferase 2	SAT2	0.94±0.04	0.71±0.02	1.31
C2_5593	Dihydrolipoyl dehydrogenase, mitochondrial	DLD	1.27±0.25	0.45±0.05	2.83
C2_11248	Dihydrolipoyllysine-residue acetyltransferase component of pyruvate dehydrogenase complex, mitochondrial	DLAT	1.45±0.29	0.69±0.03	2.10
C2_9692	Dihydrolipoyllysine-residue succinyltransferase component of 2-oxoglutarate dehydrogenase complex, mitochondrial	DLST	1.67±0.3	1.28±0.08	1.31
C2_51666	Dihydropyrimidinase	DPYS	0.78±0.11	0.46±0.03	1.72
C2_9224	Dimethylaniline monooxygenase [N-oxide-forming] 5	FMO5	1.45±0.21	0.63±0.01	2.30
C2_192	Dipeptidase 1	DPEP1	0.53±0.16	0.49±0.07	1.07
C2_366	Dipeptidyl peptidase 1	CTSC	0.83±0.14	0.46±0.1	1.80
C2_7138	Dipeptidyl peptidase 2	DPP7	0.74±0.11	0.5±0.03	1.48
C2_40681	Dipeptidyl peptidase 3	DPP3	0.96±0.09	0.7±0.06	1.38
C2_46432	Dipeptidyl peptidase 3	DPP3	0.9±0.13	0.58±0.06	1.56
C2_14794	Dipeptidyl peptidase 3	DPP3	0.96±0.14	0.55±0.06	1.75
C2_1242	Dipeptidyl peptidase 4	DPP4	0.69±0.14	0.45±0.07	1.53
C2_6203	Dipeptidyl peptidase 9	DPP9	1.03±0.04	1.24±0.18	0.84
C2_6030	Diphosphoinositol polyphosphate phosphohydrolase 1	NUDT3	1.17±0.16	2.37±0.18	0.50
C2_7518	Diphosphomevalonate decarboxylase	MVD	1.72±0.28	0.63±0.04	2.72
C2_32972	DIS3-like exonuclease 2	DIS3L2	1.08±0.03	0.99±0.1	1.09
C2_4400	Disabled homolog 2	DAB2	0.97±0.05	2.45±0.93	0.40
C2_15082	DmX-like protein 1	DMXL1	1.01±0.04	1.43±0.11	0.71
C2_20797	DNA damage-binding protein 1	DDB1	1.22±0.07	1.03±0.04	1.19
C2_54730	DNA damage-binding protein 1	DDB1	1.1±0.15	0.88±0.06	1.25
C2_15432	DNA damage-binding protein 1	DDB1	1.33±0.14	1.01±0.06	1.32
C2_2773	DNA polymerase beta	POLB	1.07±0.05	0.82±0.06	1.31
C2_17016	DNA topoisomerase 2-beta	TOP2B	0.83±0.07	0.99±0.17	0.84
C2_2056	DNA-(apurinic or apyrimidinic site) lyase	APEX1	0.92±0.03	0.99±0.06	0.93

C2_937	DnaJ homolog subfamily A member 2	DNAJA2	1.04±0.11	1.77±0.07	0.59
C2_4033	DnaJ homolog subfamily A member 2	DNAJA2	1.5±0.3	2.54±0.1	0.59
C2_7667	DnaJ homolog subfamily C member 13	DNAJC13	0.9±0.06	1.62±0.31	0.55
C2_1240	Dolichyl-diphosphooligosaccharide--protein glycosyltransferase 48 kDa subunit	DDOST	1.45±0.18	0.81±0.05	1.79
C2_37060	Dolichyl-diphosphooligosaccharide--protein glycosyltransferase subunit 1	RPN1	1.5±0.18	1.07±0.09	1.40
C2_3023	Dolichyl-diphosphooligosaccharide--protein glycosyltransferase subunit 1	RPN1	1.92±0.32	1.09±0.05	1.76
C2_1591	Dolichyl-diphosphooligosaccharide--protein glycosyltransferase subunit 2	RPN2	1.35±0.17	0.78±0.04	1.72
C2_26319	Dolichyl-diphosphooligosaccharide--protein glycosyltransferase subunit DAD1	DAD1	1.77±0.34	1.45±0.3	1.23
C2_1500	Dolichyl-diphosphooligosaccharide--protein glycosyltransferase subunit STT3A	STT3A	1.39±0.16	0.98±0.05	1.42
C2_8403	Drebrin-like protein B	DBN1	0.95±0.14	1.32±0.1	0.72
C2_10485	D-serine dehydratase	SRR	1.12±0.08	1.23±0.04	0.91
C2_6083	D-serine dehydratase	SRR	1.15±0.12	1.26±0.1	0.92
C2_9271	Dual specificity mitogen-activated protein kinase kinase 2	MAP2K2	0.77±0.1	0.95±0.32	0.81
C2_1808	Dual specificity protein phosphatase 23	DUSP23	1.28±0.22	3.04±0.5	0.42
C2_58934	Dynactin subunit 1	DCTN1	1.41±0.19	1.93±0.08	0.73
C2_3714	Dynactin subunit 1	DCTN1	0.69±0.11	0.7±0.08	0.99
C2_10012	Dynactin subunit 2	DCTN2	0.9±0.08	1.15±0.09	0.79
C2_64914	Dynactin subunit 3	DCTN3	1.17±0.08	2.5±0.13	0.47
C2_1825	Dynactin subunit 4	DCTN4	0.96±0.04	0.96±0.03	1.00
C2_3772	Dynamin-1-like protein	DNM1L	1.36±0.15	1.35±0.17	1.00
C2_6275	Dynamin-2	DNM2	1.33±0.12	2.78±0.61	0.48
C2_27580	Dynein light chain 2, cytoplasmic	DYNLL2	0.97±0.03	1.14±0.1	0.85
C2_121099	Dynein light chain roadblock-type 1	DYNLRB1	0.87±0.05	0.84±0.1	1.03
C2_26538	Dynein light chain Tctex-type 3	DYNLT3	1.08±0.04	0.91±0.08	1.18
C2_27865	E3 SUMO-protein ligase RanBP2	RGPD4	0.69±0.12	0.54±0.06	1.27
C2_7646	E3 ubiquitin-protein ligase CBL-B	CBLB	3.04±1	4.32±0.32	0.70
C2_62994	E3 ubiquitin-protein ligase HECTD1	HECTD1	1.09±0.1	1.22±0.25	0.89
C2_9198	E3 ubiquitin-protein ligase HECTD3	HECTD3	0.96±0.02	1.35±0.1	0.71
C2_18708	E3 ubiquitin-protein ligase HUWE1	HUWE1	0.85±0.06	1.41±0.31	0.61
C2_14467	E3 ubiquitin-protein ligase HUWE1	HUWE1	1.48±0.18	2.24±0.25	0.66
C2_73398	E3 ubiquitin-protein ligase HUWE1	HUWE1	1.2±0.14	1.72±0.12	0.70
C2_1338	E3 ubiquitin-protein ligase HUWE1	HUWE1	1.03±0.05	0.88±0.07	1.17
C2_14877	E3 ubiquitin-protein ligase NEDD4-like	NEDD4L	1.38±0.2	2.4±0.16	0.57
C2_5763	E3 ubiquitin-protein ligase NEDD4-like	NEDD4L	1.14±0.13	1.86±0.09	0.62
C2_2829	E3 ubiquitin-protein ligase UBR4	UBR4	1.01±0.03	1.27±0.04	0.80
C2_65482	E3 ubiquitin-protein ligase UBR4	UBR4	1.17±0.11	1.41±0.31	0.83
C2_4136	Echinoderm microtubule-associated protein-like 1	EML1	1.52±0.23	1.95±0.19	0.78
C2_5005	Echinoderm microtubule-associated protein-like 3	EML3	0.97±0.06	1.19±0.1	0.81
C3_c45339	echinoderm microtubule-associated protein-like 4-like isoform X1 [Maylandia zebra]	EML4	0.94±0.1	0.69±0.07	1.36
C2_43246	Ecto-NOX disulfide-thiol exchanger 2	ENOX2	3.5±1.24	0.54±0.08	6.49
FM150650	Ectonucleotide pyrophosphatase/phosphodiesterase family member 2	ENPP2	1.17±0.14	1.51±0.26	0.77
C3_c55223	Ectonucleotide pyrophosphatase/phosphodiesterase family member 2	ENPP2	0.87±0.04	0.98±0.1	0.89
C2_29850	Ectonucleotide pyrophosphatase/phosphodiesterase family member 3	ENPP3	0.67±0.12	0.38±0.04	1.74
C2_53228	Ectonucleotide pyrophosphatase/phosphodiesterase family member 3	ENPP3	0.67±0.14	0.29±0.02	2.33
C2_2589	Ectonucleotide pyrophosphatase/phosphodiesterase family member 6	ENPP6	0.6±0.14	0.52±0.05	1.15
C2_8279	Ectonucleotide pyrophosphatase/phosphodiesterase family member 7	ENPP7	0.75±0.13	0.47±0.04	1.61
C2_8820	EF-hand domain-containing protein D2	EFHD2	0.82±0.06	0.68±0.07	1.20
C2_2191	Egl nine homolog 1	EGLN1	0.8±0.09	1±0.14	0.81
C2_25319	EH domain-containing protein 1	EHD1	1.06±0.07	3.14±1	0.34
C2_20763	EH domain-containing protein 1	EHD1	0.9±0.05	2.43±0.77	0.37
C3_c44941	EH domain-containing protein 3	EHD3	1.29±0.16	2.87±0.46	0.45
C2_4289	Elastase-1	CELA1	0.61±0.18	0.29±0.06	2.09
C2_41087	Electrogenic sodium bicarbonate cotransporter 1	SLC4A4	1.91±0.37	0.84±0.12	2.29
C2_127	Electron transfer flavoprotein subunit alpha, mitochondrial	ETFA	1.13±0.19	0.71±0.14	1.59

C2_18	Elongation factor 1-alpha	EEF1A	1.61±0.29	2.35±1.01	0.69
C2_344	Elongation factor 1-beta	EEF1B2	1.38±0.16	2.67±0.55	0.52
C2_25907	Elongation factor 1-delta	EEF1D	1.39±0.13	1.36±0.03	1.03
C2_55	Elongation factor 1-gamma	EEF1G	1.66±0.31	2.44±0.17	0.68
C2_534	Elongation factor 2	EEF2	1.76±0.34	2.28±0.11	0.77
C2_43870	Elongation of very long chain fatty acids protein 6	ELOVL6	1.27±0.3	0.54±0.06	2.36
C2_5617	Endonuclease domain-containing 1 protein	ENDOD1	0.69±0.11	0.52±0.1	1.31
C2_4183	Endophilin-B1	SH3GLB1	0.76±0.09	0.96±0.09	0.79
C2_11409	Endoplasmic reticulum aminopeptidase 1	ERAP1	0.97±0.02	0.84±0.04	1.15
C2_21873	Endoplasmic reticulum aminopeptidase 2	ERAP2	1.01±0.05	0.97±0.17	1.05
C2_26383	Endoplasmic reticulum metallopeptidase 1	ERMP1	0.8±0.1	0.73±0.07	1.10
C2_15688	Endoplasmic reticulum resident protein 27	ERP27	1.07±0.04	1.38±0.11	0.78
C2_21801	Endoplasmic reticulum resident protein 44	ERP44	1.01±0.09	0.43±0.03	2.35
C2_1490	Endoplasmin	HSP90B1	1.27±0.12	1.43±0.04	0.89
C2_54975	Enhancer of mRNA-decapping protein 4	EDC4	1.11±0.16	1.32±0.07	0.84
C2_8738	Enhancer of mRNA-decapping protein 4	EDC4	0.84±0.06	0.94±0.07	0.90
C2_71750	Enhancer of mRNA-decapping protein 4	EDC4	0.78±0.09	0.79±0.06	0.99
C2_6952	Enhancer of rudimentary homolog	ERH	0.81±0.09	1.03±0.09	0.79
C2_13119	Enolase	ENO3	1±0.11	1.31±0.08	0.76
C2_5154	Enoyl-CoA delta isomerase 1, mitochondrial	ECI1	1.32±0.12	1.1±0.07	1.20
C2_64284	Enoyl-CoA hydratase domain-containing protein 2, mitochondrial	ECHDC2	1.26±0.1	1.12±0.05	1.13
C2_3722	Enoyl-CoA hydratase, mitochondrial	ECHS1	1.29±0.1	1.01±0.03	1.28
C2_81191	Enteropeptidase	TMPRSS15	0.68±0.15	0.28±0.07	2.41
C2_646	Eosinophil peroxidase	EPX	0.47±0.18	0.68±0.21	0.68
C2_118	Ependymin	EPD	0.69±0.15	0.34±0.03	2.01
C2_117429	Ependymin-2	EPD2	0.71±0.18	0.6±0.05	1.19
C2_14283	Epidermal growth factor receptor kinase substrate 8-like protein 2	EPS8L2	1.57±0.31	2.26±0.23	0.70
C2_5498	Epidermal growth factor receptor kinase substrate 8-like protein 3	EPS8L3	1.2±0.12	1.94±0.04	0.62
C2_782	Epididymal secretory protein E1	NPC2	0.73±0.16	0.4±0.06	1.85
C2_20845	Epiplakin	EPPK1	1.21±0.15	0.62±0.06	1.95
C2_80116	Epiplakin	EPPK1	1.47±0.28	0.46±0.01	3.17
C2_6684	Epoxide hydrolase 1	EPHX1	1.24±0.23	0.36±0.02	3.44
C2_22855	Epoxide hydrolase 2	EPHX2	1.34±0.15	1.03±0.06	1.29
C2_34000	ER degradation-enhancing alpha-mannosidase-like 3	EDEM3	1.01±0.13	0.63±0.02	1.61
C2_5519	ER lumen protein retaining receptor 2	KDELR2	1.07±0.19	0.41±0.08	2.60
C2_12878	Erlin-1	ERLIN1	1.27±0.12	1±0.09	1.27
C2_16588	Erlin-2	ERLIN2	1.13±0.11	0.4±0.01	2.84
C2_6918	ERO1-like protein alpha	ERO1L	0.98±0.07	1.44±0.18	0.68
C2_1732	Erythrocyte band 7 integral membrane protein	STOM	0.86±0.11	0.44±0.05	1.94
C2_800	ES1 protein homolog, mitochondrial	C21orf33	0.86±0.05	0.72±0.03	1.19
C2_4860	Ester hydrolase C11orf54 homolog	C11orf54	0.79±0.08	1.39±0.1	0.57
C2_12674	Estradiol 17-beta-dehydrogenase 12-B	HSD17B12	1.39±0.14	0.8±0.04	1.75
C2_3321	Ethanolamine-phosphate cytidylyltransferase	PCYT2	1.08±0.04	0.96±0.09	1.12
C2_406	Eukaryotic initiation factor 4A-I	EIF4A1	1.18±0.08	2.24±0.23	0.53
C2_746	Eukaryotic initiation factor 4A-II	EIF4A2	1.33±0.15	1.83±0.03	0.73
C2_1414	Eukaryotic initiation factor 4A-III	EIF4A3	1.15±0.06	1.19±0.11	0.97
C2_3344	Eukaryotic peptide chain release factor GTP-binding subunit ERF3A	GSPT1	0.78±0.09	1.03±0.28	0.76
C2_569	Eukaryotic peptide chain release factor subunit 1	ETF1	0.53±0.16	0.5±0.02	1.05
C2_3415	Eukaryotic translation elongation factor 1 epsilon-1	EEF1E1	1.09±0.08	2.7±0.17	0.40
C2_4011	Eukaryotic translation initiation factor 2 subunit 1	EIF2S1	1.28±0.13	1.53±0.05	0.84
C2_5966	Eukaryotic translation initiation factor 2 subunit 2	EIF2S2	1.03±0.05	1.68±0.07	0.61
C2_1614	Eukaryotic translation initiation factor 2 subunit 3	EIF2S3	1.23±0.09	1.59±0.06	0.77
C2_47079	Eukaryotic translation initiation factor 2A	EIF2A	1.19±0.07	0.87±0.06	1.36
C2_533	Eukaryotic translation initiation factor 3 subunit A	EIF3A	0.93±0.04	1.14±0.14	0.82
C2_630	Eukaryotic translation initiation factor 3 subunit B	EIF3B	1.13±0.05	1.02±0.08	1.11
C2_3032	Eukaryotic translation initiation factor 3 subunit C	EIF3C	2.44±0.66	4.69±0.34	0.52
C2_67438	Eukaryotic translation initiation factor 3 subunit C	EIF3C	1.07±0.03	1.16±0.02	0.92
C2_541	Eukaryotic translation initiation factor 3 subunit D	EIF3D	1.49±0.24	1.64±0.07	0.90
C2_114	Eukaryotic translation initiation factor 3 subunit E	EIF3E	0.96±0.06	1.62±0.42	0.59
C2_92	Eukaryotic translation initiation factor 3 subunit F	EIF3F	0.82±0.07	1.24±0.22	0.66
C2_1542	Eukaryotic translation initiation factor 3 subunit G	EIF3G	2.05±0.62	2.94±0.13	0.70
C2_413	Eukaryotic translation initiation factor 3 subunit H	EIF3H	0.89±0.09	1.45±0.25	0.61
C2_312	Eukaryotic translation initiation factor 3 subunit I	EIF3I	1.19±0.13	1.23±0.04	0.97
C2_19263	Eukaryotic translation initiation factor 3 subunit J-A	EIF3J	1.03±0.05	0.99±0.02	1.04
C2_5501	Eukaryotic translation initiation factor 3 subunit K	EIF3K	0.82±0.08	1.04±0.12	0.79
C2_372	Eukaryotic translation initiation factor 3 subunit L	EIF3L	1.28±0.13	1.68±0.09	0.76
C2_68	Eukaryotic translation initiation factor 3 subunit M	EIF3M	1.8±0.32	1.88±0.26	0.96

C2_2506	Eukaryotic translation initiation factor 4 gamma 1	EIF4G1	1.01±0.08	1.13±0.05	0.89
C2_8056	Eukaryotic translation initiation factor 4 gamma 2	EIF4G2	0.68±0.12	0.67±0.03	1.01
C2_8077	Eukaryotic translation initiation factor 4 gamma 3	EIF4G3	52.87±17.6	76.65±8.3	0.69
C2_51056	Eukaryotic translation initiation factor 4B	EIF4B	0.93±0.03	2.47±0.9	0.38
C2_1810	Eukaryotic translation initiation factor 4E	EIF4E	1.55±0.31	2.95±1.02	0.53
C2_2058	Eukaryotic translation initiation factor 4E type 2	EIF4E2	0.74±0.11	0.59±0.03	1.26
C2_1883	Eukaryotic translation initiation factor 4E type 3	EIF4E3	1.05±0.04	1.35±0.1	0.78
C2_41590	Eukaryotic translation initiation factor 4H	EIF4H	1.05±0.13	1.34±0.07	0.78
C2_6179	Eukaryotic translation initiation factor 5	EIF5	0.77±0.08	1.38±0.21	0.56
C2_37824	Eukaryotic translation initiation factor 5A-2	EIF5A2	0.95±0.03	2.81±1.96	0.34
C2_6319	Eukaryotic translation initiation factor 6	EIF6	1.1±0.1	6.98±2.01	0.16
C2_23794	Exocyst complex component 1	EXOC1	0.89±0.04	0.94±0.12	0.95
C2_5273	Exocyst complex component 2	EXOC2	1.03±0.11	1.78±0.16	0.58
C2_25949	Exocyst complex component 2	EXOC2	0.65±0.13	0.75±0.13	0.87
C2_2647	Exocyst complex component 3	EXOC3	0.83±0.09	1.39±0.25	0.60
C2_35997	Exocyst complex component 4	EXOC4	0.93±0.09	0.93±0.03	1.00
C2_7782	Exocyst complex component 5	EXOC5	0.7±0.11	0.8±0.2	0.87
C2_48178	Exocyst complex component 6	EXOC6	0.78±0.09	0.79±0.16	0.98
C2_3098	Exocyst complex component 7	EXOC7	0.74±0.09	0.97±0.11	0.76
C2_34349	Exportin-1	XPO1	1.34±0.14	2.68±0.56	0.50
C2_24267	Exportin-1	XPO1	1.09±0.07	1.18±0.06	0.93
C2_54734	Exportin-1	XPO1	1.37±0.19	1.43±0.15	0.96
C2_6241	Exportin-2	CSE1L	1.03±0.01	1.06±0.11	0.97
C2_10252	Extended synaptotagmin-1	ESYT1	0.96±0.03	1.27±0.13	0.75
C2_10706	Ezrin	EZR	0.92±0.03	3.6±2.06	0.25
C2_24358	Ezrin	EZR	1.29±0.14	2.52±0.67	0.51
C2_1226	FACT complex subunit SSRP1	SSRP1	1.04±0.07	1.23±0.05	0.85
C2_356	F-actin-capping protein subunit alpha-1	CAPZA1	1.03±0.05	1.46±0.15	0.71
C2_882	F-actin-capping protein subunit alpha-1	CAPZA1	0.87±0.05	0.91±0.05	0.95
C2_207	F-actin-capping protein subunit alpha-2	CAPZA2	1.66±0.22	1.76±0.08	0.94
C2_227	F-actin-capping protein subunit beta	CAPZB	1.97±0.33	2.29±0.1	0.86
C2_2375	Factor VIII intron 22 protein	F8A1	0.9±0.04	1.23±0.16	0.73
C2_12375	FAD synthase	FLAD1	0.68±0.11	0.64±0.03	1.07
C2_3159	FAD-linked sulfhydryl oxidase ALR	GFER	1.51±0.26	1.12±0.11	1.35
C2_17942	Far upstream element-binding protein 3	FUBP3	0.9±0.04	1.4±0.26	0.64
C2_10323	Farnesyl pyrophosphate synthase	FDPS	1.84±0.34	0.84±0.06	2.18
C2_7862	FAS-associated factor 1	FAF1	1.04±0.02	0.85±0.07	1.23
C2_750	Fatty acid binding protein 6	FABP6	0.85±0.06	25.85±2.24	0.03
C2_8342	Fatty acid-binding protein, brain	FABP7	0.9±0.1	0.57±0.08	1.60
C2_168	Fatty acid-binding protein, heart	FABP3	1.51±0.21	1.82±0.2	0.83
C2_51889	Fatty acid-binding protein, intestinal	FABP2	6.08±2.04	6.72±0.81	0.90
C2_28163	Fatty acid-binding protein, intestinal	FABP2	5.86±2.02	1.83±0.05	3.20
C2_23355	Fatty acid-binding protein, liver-type	FABP1	0.95±0.14	0.23±0.02	4.16
C2_5343	Fatty aldehyde dehydrogenase	ALDH3A2	0.99±0.19	0.16±0.01	6.06
C2_6597	F-box only protein 2	FBXO2	0.8±0.07	1.35±0.15	0.59
C2_10150	F-box only protein 22	FBXO22	1.62±0.21	1.27±0.08	1.27
C2_89103	F-box only protein 3	FBXO3	0.6±0.14	0.42±0.05	1.43
C2_58512	F-box/LRR-repeat protein 3	FBXL3	1±0.11	1.15±0.07	0.87
C2_32224	F-box/WD repeat-containing protein 12	FBXW12	0.94±0.09	0.65±0.08	1.44
C2_12659	F-box-like/WD repeat-containing protein TBL1XR1	TBL1XR1	1.28±0.18	1.59±0.17	0.81
C2_5555	FCH domain only protein 2	FCHO2	1.23±0.08	1.47±0.07	0.84
C2_37659	Female protein	WTAP	1.04±0.1	0.67±0.04	1.56
C2_76725	FERM domain-containing protein 4B	FRMD4B	0.67±0.23	0.78±0.39	0.85
C2_43673	FERM, RhoGEF and pleckstrin domain-containing protein 2	FARP2	0.79±0.11	0.6±0.04	1.31
C2_89675	Fermitin family homolog 1	FERMT1	1.12±0.06	1.01±0.01	1.11
C2_1658	Ferritin, heavy subunit	FTH1	0.82±0.06	0.92±0.21	0.89
C2_101117	Ferritin, middle subunit	FRIM	0.78±0.07	1.11±0.34	0.70
C2_43968	FGGY carbohydrate kinase domain-containing protein	FGGY	0.85±0.08	0.7±0.08	1.21
FP331537	Fibrinogen alpha chain	FGA	0.86±0.07	0.83±0.23	1.04
FP332538	Fibrinogen beta chain	FGB	0.86±0.1	0.83±0.13	1.02
FP339542	Fibrinogen beta chain	FGB	0.91±0.07	0.69±0.17	1.32
FP332283	Fibrinogen beta chain	FGB	0.75±0.09	0.55±0.15	1.37
C2_38689	Fibrinogen gamma chain	FGG	0.89±0.04	0.84±0.15	1.06
C2_5254	Fibronectin	FN1	1.18±0.07	1.05±0.16	1.13
C3_c35930	Fibronectin	FN1	1.16±0.06	0.94±0.14	1.23
C2_58470	Fibronectin	FN1	0.99±0.06	0.8±0.11	1.24
C2_63263	Fibronectin	FN1	0.92±0.03	0.61±0.08	1.51

C2_69917	Filamin-A	FLNA	0.79±0.07	1±0.3	0.79
C2_890	Filamin-A	FLNA	0.97±0.06	1.23±0.11	0.79
C2_6274	Filamin-A	FLNA	0.73±0.09	0.89±0.19	0.82
C2_45805	Filamin-A	FLNA	0.85±0.06	0.98±0.2	0.86
C2_79807	Filamin-B	FLNB	0.85±0.1	0.46±0.03	1.86
C2_65152	Filamin-C	FLNC	1.15±0.06	2.49±0.33	0.46
C2_22478	Filamin-C	FLNC	0.84±0.15	1.01±0.38	0.83
C2_6583	FK506-binding protein 1	FKBP4	1.67±0.31	2±0.04	0.84
C2_1227	Flavin reductase (NADPH)	BLVRB	1.07±0.09	1.76±0.33	0.61
C2_118260	Folate receptor beta	FOLR2	0.67±0.12	0.36±0.04	1.84
C2_13579	Formin-binding protein 1-like	Fnbp1l	1.02±0.02	1.71±0.06	0.60
C2_6545	Fructose-1,6-bisphosphatase 1	FBP1	2.78±0.76	1.23±0.09	2.25
C2_1600	Fructose-1,6-bisphosphatase isozyme 2	FBP2	2.58±0.97	3.48±0.79	0.74
C2_185	Fructose-bisphosphate aldolase B	ALDOB	2.11±0.41	1.63±0.24	1.29
C2_1285	Fructose-bisphosphate aldolase C-B	ALDOC	1±0.11	1.62±0.2	0.62
C2_23658	Fructose-bisphosphate aldolase, muscle type	FREM2	1.57±0.28	2.42±0.68	0.65
C2_32246	Fucolectin	FCL	0.66±0.11	0.64±0.04	1.02
C2_121698	Fucolectin-1	FCL1	0.81±0.07	0.7±0.03	1.17
C2_116478	Fucolectin-5	FCL5	0.99±0.04	1.3±0.23	0.77
C2_1866	Fucose mutarotase	FUOM	0.72±0.09	0.7±0.03	1.02
C2_5086	Fumarate hydratase, mitochondrial	FH	1.53±0.2	1.39±0.13	1.10
C2_3348	Fumarylacetoacetase	FAH	0.77±0.08	0.75±0.08	1.03
C2_24896	Fumarylacetoacetate hydrolase domain-containing protein 1	FAHD1	0.77±0.11	0.44±0.04	1.73
C2_1568	Fumarylacetoacetate hydrolase domain-containing protein 2	FAHD2B	0.76±0.1	0.79±0.06	0.96
C2_11237	GA-binding protein alpha chain	GABPA	0.92±0.06	0.85±0.09	1.09
C2_2035	Galactocerebrosidase	GALC	0.77±0.13	0.6±0.08	1.28
C2_14778	Galactokinase	GALK1	0.99±0.07	2.77±0.79	0.36
C2_11273	Galactose-1-phosphate uridylyltransferase	GALT	0.98±0.06	2±0.16	0.49
C2_28569	Galectin-3	LGALS3	0.84±0.09	0.41±0.05	2.05
C2_12854	Galectin-8	LGALS8	0.95±0.06	0.99±0.07	0.95
C2_985	Galectin-9	LGALS9B	1.01±0.06	1.01±0.12	1.01
C2_14958	Galectin-related protein	LGALSL	0.76±0.09	0.56±0.06	1.36
C2_226	Gamma-aminobutyric acid receptor-associated protein	GABARAPL1	0.8±0.07	0.97±0.04	0.82
C2_384	Gamma-aminobutyric acid receptor-associated protein-like 2	GABARAPL2	0.82±0.07	0.86±0.04	0.95
C2_5451	Gamma-butyrobetaine dioxygenase	BBOX1	0.71±0.12	0.24±0.02	3.01
C2_3265	Gamma-glutamyl hydrolase	GGH	0.82±0.07	0.94±0.08	0.87
AM954422	Gamma-glutamylaminecyclotransferase A	GGACT	0.87±0.07	0.64±0.05	1.36
C2_15180	Gamma-glutamylcyclotransferase	GGCT	0.94±0.08	0.9±0.05	1.04
C2_1354	Gamma-glutamyltranspeptidase 1	GGT1	0.69±0.12	0.53±0.05	1.29
C2_63	Gamma-interferon-inducible lysosomal thiol reductase	IFI30	0.71±0.1	0.85±0.19	0.83
C2_2606	Ganglioside GM2 activator	GM2A	0.67±0.12	0.46±0.08	1.46
C2_34628	Gastric intrinsic factor	GIF	0.55±0.17	0.18±0.01	3.10
C2_14681	GDH/6PGL endoplasmic bifunctional protein	H6PD	0.95±0.04	0.65±0.02	1.47
C2_11267	GDP-D-glucose phosphorylase C15orf58 homolog	GDPGP1	1.04±0.1	1.62±0.07	0.64
C2_3719	GDP-mannose 4,6 dehydratase	GMDS	0.78±0.12	0.42±0.02	1.87
C2_265	Gelsolin	GSN	1.14±0.1	1.03±0.06	1.11
C2_17159	Gephyrin	GPHN	0.9±0.05	0.86±0.04	1.05
C2_3592	Geranylgeranyl pyrophosphate synthase	GGPS1	0.89±0.05	0.87±0.05	1.02
C2_16413	Geranylgeranyl transferase type-1 subunit beta	PGGT1B	1.03±0.02	0.87±0.04	1.19
C2_16816	Geranylgeranyl transferase type-2 subunit beta	RABGGTB	0.86±0.05	1.03±0.05	0.83
C2_11265	Glia maturation factor beta	GMFB	1.22±0.2	2.7±0.42	0.45
C2_1692	Glia-derived nexin	SERPINE2	0.75±0.09	0.64±0.12	1.17
C2_2386	Glucosamine 6-phosphate N-acetyltransferase	GNPNAT1	1.08±0.08	0.41±0.05	2.66
C2_768	Glucosamine-6-phosphate isomerase 1	GNPDA1	1.87±0.32	4.27±0.19	0.44
C2_8719	Glucosamine--fructose-6-phosphate aminotransferase [isomerizing] 1	GNPDA1	0.98±0.06	0.93±0.14	1.05
C2_69259	Glucosamine--fructose-6-phosphate aminotransferase [isomerizing] 1	GNPDA1	0.84±0.18	0.55±0.1	1.52
C2_20259	Glucosamine--fructose-6-phosphate aminotransferase [isomerizing] 2	GNPDA2	1.25±0.26	1.65±0.72	0.76
C2_60606	Glucosamine--fructose-6-phosphate aminotransferase [isomerizing] 2	GNPDA2	1.6±0.42	1.4±0.11	1.14
C2_7677	Glucose-6-phosphatase 3	G6PC3	0.9±0.04	0.51±0.06	1.76
C2_5943	Glucose-6-phosphate 1-dehydrogenase	G6PD	1.08±0.09	1.4±0.14	0.77
C2_394	Glucose-6-phosphate isomerase	GPI	0.71±0.12	0.52±0.07	1.37
C2_2612	Glucosidase 2 subunit beta	PRKCSH	1.13±0.15	0.59±0.1	1.90
C2_1745	Glucosylceramidase	GBA	0.76±0.14	0.48±0.05	1.59

C2_10880	Glucuronokinase 1	GLCAK1	0.92±0.11	0.86±0.13	1.07
C2_23708	Glutamate carboxypeptidase 2	Folh1	0.77±0.1	0.53±0.01	1.47
C2_3924	Glutamate dehydrogenase, mitochondrial	GLUD1	3.02±0.98	0.93±0.02	3.25
C2_2794	Glutamate--cysteine ligase catalytic subunit	GCLC	1.19±0.14	1.57±0.1	0.76
C2_944	Glutamine synthetase	GLUL	0.88±0.05	2.14±0.43	0.41
C2_34856	Glutamine-dependent NAD(+) synthetase	NADSYN1	0.63±0.13	0.82±0.05	0.77
C2_2268	Glutaminyl-tRNA synthetase	Qars	1.03±0.07	1.77±0.34	0.58
C2_4118	Glutamyl aminopeptidase	ENPEP	0.74±0.1	0.61±0.07	1.22
C2_46090	Glutaredoxin	GLRX5	0.92±0.08	1.3±0.1	0.71
C2_6320	Glutaredoxin 3	GLRX3	1.07±0.14	2.77±0.6	0.39
C2_57075	Glutaredoxin 3	GLRX3	1.1±0.12	2.29±0.12	0.48
C2_3733	Glutaryl-CoA dehydrogenase, mitochondrial	GCDH	1.32±0.12	0.86±0.07	1.54
C2_345	Glutathione peroxidase 1	GPX1	0.76±0.11	0.29±0.03	2.64
C2_296	Glutathione peroxidase 2	GPX2	0.71±0.12	0.3±0.03	2.36
C2_4046	Glutathione peroxidase 3	GPX3	0.6±0.13	0.55±0.03	1.08
C2_4586	Glutathione peroxidase 4a	GPX4A	0.94±0.04	0.69±0.03	1.37
C2_19719	Glutathione reductase, mitochondrial	GSR	0.73±0.11	0.39±0.03	1.85
C2_5598	Glutathione S-transferase 3	GSTA3	0.92±0.09	0.7±0.06	1.32
C2_117130	Glutathione S-transferase A	GSTA1	1.48±0.35	1.16±0.1	1.27
C2_112663	Glutathione S-transferase A4	GSTA4	1.2±0.09	2.02±0.12	0.59
C2_112228	Glutathione S-transferase alpha M14	GSTAM14	1.32±0.11	2.13±0.16	0.62
C2_430	Glutathione S-transferase kappa 1	GSTK1	0.8±0.11	0.46±0.04	1.74
C2_868	Glutathione S-transferase Mu 3	Gstm3	1.23±0.08	1.16±0.08	1.06
C2_1065	Glutathione S-transferase omega-1	GSTO1	1.1±0.08	1.03±0.02	1.08
C2_1660	Glutathione S-transferase theta-1	Gstt1	1.51±0.3	2.89±0.1	0.52
C2_5198	Glutathione S-transferase theta-4	Gstt4	2.73±0.67	3.64±0.34	0.75
C2_109937	Glutathione S-transferase Yc	GSTA3	0.81±0.17	0.55±0.06	1.48
C2_25988	Glutathione synthetase	GSS	0.77±0.09	0.46±0.03	1.69
C2_29	Glyceraldehyde 3-phosphate dehydrogenase, testis-specific	GAPDH	0.72±0.1	1.02±0.17	0.71
C2_17	Glyceraldehyde-3-phosphate dehydrogenase	GAPDH	0.75±0.16	0.29±0.04	2.62
C2_688	Glycerol-3-phosphate dehydrogenase [NAD+], cytoplasmic	GPD1	1.17±0.09	1.17±0.23	1.00
C2_2813	Glycerol-3-phosphate dehydrogenase 1-like protein	GPD1L	1.58±0.3	1.73±0.31	0.91
C2_19238	Glycerophosphodiester phosphodiesterase 1	GDE1	0.84±0.11	0.45±0.03	1.86
C2_3169	Glycine amidinotransferase, mitochondrial	GATM	0.95±0.04	0.71±0.07	1.34
C2_1837	Glycine cleavage system H protein, mitochondrial	GCSH	1.05±0.04	1.12±0.15	0.94
C2_9479	Glycine cleavage system H protein, mitochondrial	GCSH	0.9±0.17	0.16±0.03	5.74
C3_c37918	Glycine dehydrogenase [decarboxylating], mitochondrial	GLDC	1.3±0.19	0.4±0.05	3.22
C2_23606	Glycine dehydrogenase [decarboxylating], mitochondrial	GLDC	1.29±0.14	0.39±0.04	3.32
C2_1076	Glycine N-acyltransferase-like protein 3	GLYATL3	1.32±0.19	4.21±0.2	0.31
C2_70872	Glycine N-acyltransferase-like protein Keg1	Keg1	0.8±0.07	0.68±0.06	1.18
C2_27493	Glycine N-methyltransferase	GNMT	3.35±0.92	1.11±0.16	3.01
C2_20446	Glycogen debranching enzyme	AGL	2.26±0.54	2.83±0.36	0.80
C2_15427	Glycogen debranching enzyme	AGL	1.12±0.06	1.29±0.03	0.87
C2_7766	Glycogen debranching enzyme	AGL	1.21±0.09	1.35±0.09	0.90
C2_8664	Glycogen debranching enzyme	AGL	1.1±0.04	0.5±0.08	2.21
C2_4003	Glycogen phosphorylase, liver form	PYGL	0.75±0.09	0.93±0.12	0.81
C2_75689	Glycogen phosphorylase, liver form	PYGL	0.96±0.03	0.73±0.07	1.31
C2_5276	Glycogen phosphorylase, muscle form	PYGM	0.92±0.04	1.32±0.12	0.70
C2_1350	Glycolipid transfer protein	GLTP	0.95±0.06	1.35±0.1	0.70
C2_637	Glycylpeptide N-tetradecanoyltransferase 1	NMT1	0.93±0.04	0.73±0.05	1.27
C2_1726	Glycyl-tRNA synthetase	GLYS	1.13±0.1	1.64±0.08	0.69
C2_2114	Glyoxalase domain-containing protein 4	GLOD4	1.59±0.24	1.95±0.05	0.82
C2_2680	Glyoxalase domain-containing protein 5	GLOD5	0.72±0.11	0.39±0.03	1.85
C2_1994	Glyoxylate reductase/hydroxypyruvate reductase	GRHPR	0.94±0.08	0.83±0.08	1.13
C2_1539	GMP synthase [glutamine-hydrolyzing]	GMPS	0.93±0.06	0.98±0.07	0.94
C2_25456	Golgi SNAP receptor complex member 2	GOSR2	0.75±0.08	0.62±0.04	1.21
C2_12302	Golgi-specific brefeldin A-resistance guanine nucleotide exchange factor 1	GBF1	0.94±0.04	1.04±0.06	0.91
C2_3971	Grancalcin	GCA	1.4±0.16	1.89±0.1	0.74
C2_4717	Growth factor receptor-bound protein 2	GRB2	1.87±0.48	2.94±0.11	0.63
C2_26978	GTPase IMAP family member 2	GIMAP2	1.29±0.11	1.25±0.04	1.03
C2_28934	GTPase IMAP family member 4	GIMAP4	1.02±0.09	1±0.06	1.02
C2_2112	GTPase KRas	KRAS	1.29±0.11	1.48±0.15	0.88
C2_1141	GTP-binding nuclear protein Ran	RAN	1.73±0.39	2.91±0.54	0.59
C2_1272	GTP-binding protein SAR1b	SAR1B	1.31±0.11	1.33±0.11	0.99
C2_762	Guanidinoacetate N-methyltransferase	GAMT	3.04±0.82	1.73±0.05	1.76
C2_18132	Guanine nucleotide exchange factor VAV2	VAV2	0.85±0.06	0.92±0.04	0.92

C2_3738	Guanine nucleotide-binding protein G(i) subunit alpha-1	GNAI1	0.93±0.05	1.2±0.14	0.77
C2_829	Guanine nucleotide-binding protein G(i) subunit alpha-2	GNAI2	0.92±0.03	1±0.08	0.92
C2_8162	Guanine nucleotide-binding protein G(I)/G(S)/G(T) subunit beta-1	GNB1	1.58±0.28	0.88±0.05	1.81
C2_3700	Guanine nucleotide-binding protein G(o) subunit alpha	GNAO1	0.78±0.07	0.98±0.02	0.80
C2_11577	Guanine nucleotide-binding protein G(olf) subunit alpha	GNAL	1.12±0.07	0.86±0.12	1.30
C2_17403	Guanine nucleotide-binding protein G(q) subunit alpha	GNAQ	1.18±0.08	0.88±0.04	1.33
C2_75053	Guanine nucleotide-binding protein subunit alpha-11	GNA11	1.15±0.07	1.12±0.05	1.03
C2_45	Guanine nucleotide-binding protein subunit beta-2-like 1	GNB2L1	1.76±0.39	1.67±0.17	1.05
C2_10055	Guanosine-3',5'-bis(diphosphate) 3'-pyrophosphohydrolase MESH1	HDDC3	1.38±0.22	2.22±0.14	0.62
C2_2205	H/ACA ribonucleoprotein complex subunit 4	DKC1	1.25±0.11	1.34±0.09	0.93
C2_4167	H-2 class I histocompatibility antigen, L-D alpha chain	HLA-A	1.44±0.16	0.92±0.05	1.57
C2_3408	Haloacid dehalogenase-like hydrolase domain-containing protein 2	HDHD2	0.81±0.11	0.55±0.06	1.46
C2_14618	Haptoglobin	HP	1.02±0.13	1.02±0.09	1.00
C2_5901	Harmonin	USH1C	1.3±0.29	2.8±0.25	0.47
C2_5365	HEAT repeat-containing protein 7A	MROH1	0.93±0.07	1.71±0.47	0.55
C2_15999	Heat shock 70 kDa protein 1	HSPA1L	1.1±0.08	1.17±0.12	0.94
C2_13612	Heat shock 70 kDa protein 4	HSPA4	0.98±0.1	1.56±0.61	0.63
C2_6720	Heat shock 70 kDa protein 4	HSPA4	1.23±0.09	1.59±0.31	0.77
C2_58	Heat shock cognate 70 kDa protein	Hspa1b	2.51±0.51	2.57±0.16	0.98
C2_330	Heat shock factor-binding protein 1	HSBP1	1.09±0.07	2.49±0.18	0.44
C2_2116	Heat shock protein 105 kDa	HSPH1	1.39±0.18	3.69±1.05	0.38
C2_25526	Heat shock protein 67B2	Tstd3	0.98±0.1	2.06±0.1	0.48
C2_11513	Heme-binding protein 1	HEBP1	1.27±0.11	1.7±0.03	0.75
C2_69269	Heme-binding protein 2	HEBP2	0.84±0.06	1.73±0.05	0.49
C2_2327	Hemicentin-1	HMCN1	1.36±0.21	1.55±0.2	0.88
C2_7127	Hemoglobin subunit alpha-A	HBAA	0.8±0.15	0.64±0.06	1.25
C2_5435	Hemoglobin subunit alpha-B	HBAB	0.79±0.15	0.66±0.08	1.21
C2_102093	Hemoglobin subunit beta	HBB	0.79±0.12	0.68±0.05	1.16
C2_105738	Hemoglobin subunit beta	HBB	0.72±0.13	0.51±0.02	1.42
C2_78448	Hemoglobin subunit beta-A	Hbb-b2	0.72±0.12	0.63±0.05	1.14
C2_27397	Hemopexin	HPX	0.85±0.08	0.96±0.14	0.89
C2_9919	Hepatocyte growth factor-regulated tyrosine kinase substrate	HGS	0.82±0.07	1.81±0.1	0.45
C2_7954	Hepatocyte nuclear factor 4-gamma	HNF4G	0.85±0.08	0.73±0.08	1.16
C2_1739	Hepatoma-derived growth factor	HDGF	1.43±0.28	2.82±0.13	0.51
FM151678	Hephaestin	HEPH	0.84±0.06	0.6±0.11	1.40
C2_6512	Heterogeneous nuclear ribonucleoprotein A/B	HNRNPAB	1.25±0.1	2.85±1.25	0.44
C2_1787	Heterogeneous nuclear ribonucleoprotein A0	HNRNPA0	1.11±0.12	2.27±0.2	0.49
C2_24145	Heterogeneous nuclear ribonucleoprotein A1	Hnrnpa1	1.29±0.21	1.79±0.1	0.72
C2_15328	Heterogeneous nuclear ribonucleoprotein C	HNRNPC	1.03±0.09	1.07±0.1	0.96
C2_3241	Heterogeneous nuclear ribonucleoprotein H	HNRNPH1	1.37±0.13	1.36±0.02	1.01
C2_15127	Heterogeneous nuclear ribonucleoprotein M	HNRNPM	1.1±0.04	2.84±0.85	0.39
C2_63887	Heterogeneous nuclear ribonucleoprotein M	HNRNPM	0.92±0.07	0.8±0.04	1.15
C2_26519	Heterogeneous nuclear ribonucleoprotein Q	SYNCRIP	1.01±0.03	1.19±0.22	0.85
C2_53445	Heterogeneous nuclear ribonucleoprotein R	HNRNPR	0.96±0.04	0.81±0.06	1.19
C2_46867	Heterogeneous nuclear ribonucleoprotein U	HNRNPU	1.01±0.05	1.06±0.13	0.95
C2_1517	Heterogeneous nuclear ribonucleoprotein U-like protein 1	HNRNPUL1	1.27±0.1	1.38±0.05	0.92
C2_75390	Hexokinase-1	HK1	0.91±0.05	1.15±0.31	0.79
C2_81938	Hexokinase-2	HK2	0.98±0.03	1.04±0.09	0.94
C2_4097	Hexosaminidase D	HEXDC	0.83±0.06	0.78±0.05	1.07
C2_8165	Hibernation-specific plasma protein HP-55	HP55	1.01±0.04	0.86±0.11	1.17
C2_13670	High affinity cGMP-specific 3',5'-cyclic phosphodiesterase 9A	PDE9A	1.19±0.32	1.67±0.48	0.72
C2_11749	High choriolytic enzyme 1	HCEA	0.64±0.17	0.31±0.07	2.09
C2_3658	Histamine N-methyltransferase	HNMT	0.83±0.06	0.69±0.04	1.21
C2_1967	Histamine N-methyltransferase A	HNMT	1.6±0.34	1.98±0.24	0.81
C2_2901	Histidine triad nucleotide-binding protein 1	HINT1	1.13±0.07	2.18±0.45	0.52
C2_9935	Histidyl-tRNA synthetase, cytoplasmic	HARS2	1.49±0.2	1.25±0.11	1.19
C2_65292	Histidyl-tRNA synthetase, cytoplasmic	HARS2	1.39±0.17	1.12±0.04	1.24
C2_16721	Histone deacetylase 10	HDAC10	1.61±0.25	2±0.19	0.80
C2_12456	Histone H2A	H2AFB3	1.17±0.08	3.02±0.92	0.39
C2_390	Histone H2A.V	H2AFV	1.13±0.22	1.56±0.26	0.73
C2_101057	Histone H2B 1/2	HIST2H2BE	2.47±0.54	1.79±0.21	1.38
C2_3081	Histone H3.3	H3F3A	5.3±1.68	2.29±0.24	2.32
C2_117463	Histone H4	HIST1H4A	5.48±2.24	2.08±0.28	2.63
C2_2830	Histone-binding protein RBBP7	RBBP7	1.05±0.05	1.25±0.08	0.84
C2_16287	Histone-lysine N-methyltransferase setd3	SETD3	0.92±0.11	0.98±0.11	0.94

C2_7824	Homocysteine S-methyltransferase ybgG	YBGG	1.19±0.17	1.09±0.06	1.09
C2_25525	Host cell factor 1	HCFC1	0.89±0.07	0.62±0.05	1.44
C2_16741	Host cell factor 1	HCFC1	0.86±0.11	0.56±0.04	1.53
C2_6226	Hsc70-interacting protein	ST13	0.98±0.04	2.45±0.65	0.40
C2_1435	Hsp70-binding protein 1	HSPBP1	0.98±0.11	1.14±0.15	0.86
C2_17788	Huntingtin	HTT	1.23±0.08	1.58±0.18	0.78
C2_39681	Hyaluronan-binding protein 2	HABP2	0.94±0.03	1.05±0.05	0.90
C2_23228	Hyaluronidase-1	HYAL1	0.66±0.13	0.47±0.05	1.39
C2_15683	Hyaluronidase-2	HYAL2	0.72±0.12	0.32±0.04	2.23
C2_15186	Hydroxyacid-oxoacid transhydrogenase, mitochondrial	ADHFE1	0.87±0.05	0.5±0.01	1.74
C2_465	Hydroxyacyl-coenzyme A dehydrogenase, mitochondrial	HADH	0.78±0.13	0.2±0.02	3.90
C2_610	Hydroxyacylglutathione hydrolase, mitochondrial	HAGH	1.16±0.06	1.27±0.07	0.91
C2_1134	Hydroxymethylglutaryl-CoA lyase	HMGCL	1.18±0.08	0.87±0.08	1.36
C2_17940	Hydroxymethylglutaryl-CoA synthase, cytoplasmic	HMGCS1	1.2±0.17	0.66±0.05	1.82
C3_c25627	hypothetical protein LOC100692391 [Oreochromis niloticus]	GIMAP8	0.83±0.12	0.34±0.03	2.47
C2_679	Hypoxanthine-guanine phosphoribosyltransferase	HPRT1	1.74±0.32	2.28±0.03	0.76
C2_6116	Hypoxia-inducible factor 1-alpha inhibitor	HIF1AN	1.67±0.27	0.9±0.14	1.86
C2_110618	Ig heavy chain Mem5	Ighg2a	0.9±0.07	1.04±0.1	0.87
C2_114371	Ig heavy chain V region XIG14	IGXIG14	0.94±0.03	1.03±0.12	0.91
C2_58543	Ig kappa chain V region 3315	IGK3315	0.75±0.1	1±0.12	0.75
C2_26869	Ig kappa-b4 chain C region	K-BAS	0.72±0.11	0.63±0.06	1.14
C2_107145	Ig lambda chain V-I region NEW	IGLV1-NEW	1±0.01	1±0.16	1.00
C2_112414	Ig lambda-3 chain C regions	IGLC3	1.12±0.2	1.2±0.21	0.94
C2_46036	Ig mu chain C region	IGHM	1.17±0.2	2.06±0.47	0.57
C2_4639	Ig mu chain C region membrane-bound form	IGHM	0.99±0.24	0.85±0.26	1.16
C2_13391	IgGFc-binding protein	FCGBP	0.63±0.14	0.39±0.05	1.60
C2_113099	Immunoglobulin lambda-like polypeptide 5	IGLL5	0.69±0.12	0.65±0.05	1.06
C2_2478	Immunoglobulin-binding protein 1	IGBP1	1.02±0.08	1.53±0.5	0.66
C2_19893	Importin subunit alpha-4	KPNA3	1.14±0.16	1.73±0.11	0.66
C2_2908	Importin subunit alpha-4	KPNA3	0.92±0.06	0.91±0.03	1.01
C3_lrc8695	Importin subunit beta-1	KPNB1	1.67±0.3	2.22±0.16	0.75
C3_c11824	Importin-11	IPO11	1.25±0.08	0.84±0.11	1.48
C2_92624	Importin-5	IPO5	0.97±0.06	1.93±0.39	0.50
C2_14281	Importin-7	IPO7	1.02±0.05	1.57±0.13	0.65
C3_c28568	Importin-9	IPO9	0.82±0.06	0.7±0.05	1.18
C2_126	Inorganic pyrophosphatase	PPA1	1.44±0.17	0.83±0.05	1.73
C2_1885	Inosine-5'-monophosphate dehydrogenase 2	IMPDH2	0.79±0.1	0.6±0.03	1.32
C2_12079	Inosine-uridine preferring nucleoside hydrolase	IUNH	1.86±0.36	0.63±0.04	2.94
C2_14114	Inositol monophosphatase 1	IMPA1	0.97±0.11	1.12±0.1	0.87
C2_4262	Inositol monophosphatase 1	IMPA1	0.95±0.04	0.92±0.05	1.03
C2_78142	Inositol monophosphatase 2	IMPA2	1.26±0.12	0.88±0.09	1.43
C2_19624	Inositol polyphosphate 1-phosphatase	INPP1	1.11±0.14	1.65±0.21	0.68
C2_13269	Inositol polyphosphate 5-phosphatase OCRL-1	OCRL	1.3±0.11	1.23±0.05	1.05
C3_c55528	Insulin-degrading enzyme	IDE	0.81±0.07	0.91±0.06	0.90
C2_42269	Insulin-degrading enzyme	IDE	0.83±0.06	0.82±0.04	1.01
C2_8119	Insulin-like growth factor binding protein complex acid liable subunit	IGFALS	1.08±0.09	1.25±0.13	0.86
C2_5183	Integrin alpha-M	ITGAM	0.86±0.05	0.7±0.06	1.23
C2_12256	Integrin beta-1	ITGB1	0.59±0.16	0.59±0.09	1.00
C2_15079	Integrin-linked protein kinase	ILK	1.05±0.15	1.37±0.18	0.77
C2_14668	Intelectin-1a	ITLN1	0.64±0.13	0.39±0.05	1.66
C2_36156	Intelectin-2	ITLN2	0.76±0.14	0.39±0.06	1.93
FP333792	Inter-alpha-trypsin inhibitor heavy chain H2	ITIH2	1.04±0.08	1.63±0.28	0.64
C2_12615	Inter-alpha-trypsin inhibitor heavy chain H3	ITIH3	1.54±0.37	2.12±0.3	0.73
C3_lrc30120	inter-alpha-trypsin inhibitor heavy chain H3-like [Takifugu rubripes]	ITIH3	1.29±0.17	1.71±0.19	0.75
C2_5524	Interferon regulatory factor 5	IRF5	1±0.07	1.29±0.16	0.77
C2_624	Interferon-induced 35 kDa protein homolog	IFI35	0.81±0.07	0.62±0.03	1.31
C2_16818	Interferon-induced GTP-binding protein Mx	MX1	1.05±0.02	1.02±0.15	1.03
C2_8010	Interleukin enhancer-binding factor 2 homolog	ILF2	1.37±0.18	2.05±0.05	0.67
C2_10673	Interleukin enhancer-binding factor 3 homolog	ILF3	0.91±0.06	0.95±0.04	0.96
C2_118270	Intermediate filament protein ON3	ION3	2.95±1.09	12.22±3.47	0.24
C3_c54887	Intersectin-1	ITSN1	1.19±0.14	0.97±0.06	1.22
C2_2049	Intestinal-type alkaline phosphatase	ALPI	0.89±0.07	0.38±0.02	2.34
C2_3089	Iron-responsive element-binding protein 2	IREB2	0.99±0.07	0.87±0.08	1.14
C2_4651	Isoamyl acetate-hydrolyzing esterase 1 homolog	IAH1	1.15±0.12	1.53±0.04	0.75
C2_11243	Isobutyryl-CoA dehydrogenase, mitochondrial	ACAD8	0.63±0.13	0.41±0.05	1.54

C2_17229	Isochorismatase domain-containing protein 2, mitochondrial	ISOC2	0.97±0.02	0.73±0.06	1.32
C2_1776	Isocitrate dehydrogenase [NADP] cytoplasmic	IDH1	3.2±0.84	3.69±0.13	0.87
C2_250	Isocitrate dehydrogenase [NADP], mitochondrial	IDH2	1.04±0.08	1.42±0.14	0.74
C3_c36816	Isoleucine--tRNA ligase, cytoplasmic	IARS	0.96±0.07	0.91±0.09	1.05
C2_58851	Isoleucyl-tRNA synthetase, cytoplasmic	IARS	1.28±0.11	1.61±0.03	0.80
C2_46456	Isoleucyl-tRNA synthetase, cytoplasmic	IARS	1.37±0.29	1.37±0.04	1.00
C2_2044	Isopentenyl-diphosphate Delta-isomerase 1	IDI1	1.39±0.31	0.38±0.02	3.64
C2_2160	Isovaleryl-CoA dehydrogenase, mitochondrial	IVD	0.7±0.11	0.35±0.04	2.00
C2_18025	IST1 homolog	IST1	0.89±0.08	1.38±0.24	0.64
C2_2147	Junction plakoglobin	JUP	1.24±0.08	2.46±0.3	0.50
C2_23019	Junction plakoglobin	JUP	1.03±0.09	1.71±0.1	0.60
C2_1814	Junctional adhesion molecule A	F11R	0.95±0.07	0.95±0.08	1.01
C2_6330	Kallikrein-8	KLK8	0.58±0.14	1.04±0.28	0.56
C2_5685	Kaptin	KPTN	0.84±0.06	0.41±0.01	2.06
C2_83200	Katanin p60 ATPase-containing subunit A-like 2	KATNAL2	1.92±0.34	3.36±0.33	0.57
C2_7027	KDEL motif-containing protein 1	KDELC1	0.88±0.04	0.73±0.1	1.20
C2_19579	Keratin, type I cytoskeletal 13	KRT13	1.14±0.19	2.19±0.12	0.52
C2_2372	Keratin, type I cytoskeletal 18	KRT18	0.84±0.06	0.72±0.08	1.17
C2_116635	Keratin, type I cytoskeletal 18-A	KRT18	0.77±0.16	0.31±0.05	2.46
C2_86457	Keratin, type I cytoskeletal 19	KRT19	1.94±0.54	3.78±0.18	0.51
C2_74897	Keratin, type I cytoskeletal 19	KRT19	1.59±0.21	2.82±1.33	0.56
C2_51684	Keratin, type I cytoskeletal 50 kDa	KRT5	0.84±0.08	1.64±0.63	0.52
C2_1442	Keratin, type II cytoskeletal 8	KRT8	0.82±0.09	0.5±0.04	1.65
C2_2275	Ketohexokinase	KHK	1.03±0.11	0.49±0.03	2.10
C2_1049	KH domain-containing, RNA-binding, signal transduction-associated protein 1	KHDRBS1	2.19±0.71	6.02±1.6	0.36
C2_4980	Kinectin	Ktn1	0.67±0.12	1.67±1.07	0.40
C2_3990	Kinectin	Ktn1	0.74±0.09	1.24±0.32	0.60
C2_7678	Kinesin light chain 1	KLC1	0.92±0.05	1.35±0.13	0.68
C2_38068	Kinesin-like protein KIF3A	KIF3A	0.57±0.15	0.54±0.08	1.07
C2_29495	Kininogen	Kng1	1.07±0.16	4.08±1.14	0.26
s_fjad0002a08_f_1	KLRAQ motif-containing protein 1	PPP1R21	0.87±0.09	1.87±0.2	0.47
C2_29253	Kynureninase	KYNU	0.86±0.06	0.69±0.06	1.24
C2_1795	Kynurenine--oxoglutarate transaminase 1	CCBL1	0.87±0.07	1.06±0.08	0.83
C2_5123	Lactase-phlorizin hydrolase	LCT	0.61±0.14	0.28±0.03	2.21
C2_17959	Lactase-phlorizin hydrolase	LCT	0.59±0.15	0.26±0.03	2.26
C2_380	Lactoylglutathione lyase	GLO1	0.76±0.11	0.52±0.05	1.46
C2_4354	Lambda-crystallin homolog	CRYL1	0.8±0.12	0.83±0.05	0.97
C2_19670	Lamin-B2	LMNB2	0.89±0.06	0.72±0.03	1.23
C2_23836	Laminin subunit alpha-3	LAMA3	0.98±0.04	0.49±0.03	2.00
C2_13399	LanC-like protein 2	LANCL2	0.76±0.09	0.6±0.06	1.25
C2_36474	Lanosterol synthase	LSS	2.16±0.66	0.49±0.04	4.37
C2_21093	L-asparaginase	ASPG	0.95±0.05	1.32±0.04	0.72
C2_141	Latexin	LXN	1.2±0.16	0.88±0.07	1.37
C2_10666	Lectin	LEC	0.87±0.07	1.91±0.42	0.45
C2_65791	Lethal(2) giant larvae protein homolog 2-like	LLGL2	0.93±0.03	1.23±0.04	0.76
C2_15081	Lethal(2) giant larvae protein homolog 2-like	LLGL2	0.9±0.03	1.09±0.03	0.82
C2_18261	Leucine-rich alpha-2-glycoprotein	LRG1	0.9±0.05	0.77±0.1	1.18
C2_12713	Leucine-rich repeat protein SHOC-2	SHOC2	0.83±0.1	0.5±0.07	1.66
C2_38024	Leucyl-tRNA synthetase, cytoplasmic	LARS	1.29±0.11	1.78±0.02	0.73
C2_74310	Leucyl-tRNA synthetase, cytoplasmic	LARS	1.31±0.1	1.43±0.07	0.91
C2_23571	Leukocyte cell-derived chemotaxin-2	LECT2	1.1±0.32	1.01±0.15	1.09
C2_2628	Leukocyte elastase inhibitor	SERPINB1	0.79±0.12	0.37±0.02	2.16
C2_7973	Leukocyte surface antigen CD53	CD53	1.18±0.14	0.82±0.05	1.44
C2_695	Leukotriene A-4 hydrolase	LTA4H	0.94±0.05	0.28±0.02	3.33
C2_109655	LIM and SH3 domain protein 1	LASP1	1.34±0.16	1.79±0.11	0.75
C2_95063	Lipid phosphate phosphohydrolase 1	Ppap2a	0.89±0.13	0.35±0.04	2.57
C2_22890	Lipocalin	LCN1	1.05±0.03	0.88±0.08	1.20
C2_80344	Lipopolysaccharide-responsive and beige-like anchor protein	LRBA	0.98±0.08	1.6±0.11	0.62
C2_17285	Lipopolysaccharide-responsive and beige-like anchor protein	LRBA	1.04±0.06	1.34±0.17	0.78
C2_60336	Lipopolysaccharide-responsive and beige-like anchor protein	LRBA	1.18±0.09	1.4±0.11	0.85
C2_22786	Lipopolysaccharide-responsive and beige-like anchor protein	LRBA	0.81±0.07	0.89±0.07	0.90
C2_25312	Lipopolysaccharide-responsive and beige-like anchor protein	LRBA	0.97±0.08	1.01±0.07	0.96
C2_61943	LisH domain and HEAT repeat-containing protein KIAA1468 homolog	KIAA1468	0.77±0.08	0.51±0.03	1.49
C2_17725	Lissencephaly-1 homolog A	PAFAH1B1	1.05±0.04	1±0.07	1.05
C2_6672	Liver carboxylesterase	CES1	0.79±0.15	0.11±0.01	6.93

C2_13242	Liver carboxylesterase 2	CES1	0.63±0.14	0.19±0.02	3.34
FP338966	Liver carboxylesterase 22	Ces1e	0.71±0.12	0.57±0.07	1.25
C2_1393	L-lactate dehydrogenase A chain	LDHA	1.59±0.26	2.91±0.19	0.55
C2_6197	L-lactate dehydrogenase B chain	LDHB	1.26±0.21	2.92±0.53	0.43
C2_117493	LON peptidase N-terminal domain and RING finger protein 2	LONRF2	1.04±0.04	1.37±0.22	0.76
C2_22040	Long-chain fatty acid transport protein 4	SLC27A4	1.51±0.2	0.64±0.03	2.36
C2_12825	Long-chain specific acyl-CoA dehydrogenase, mitochondrial	ACADL	0.99±0.06	0.7±0.03	1.42
C2_15955	Long-chain specific acyl-CoA dehydrogenase, mitochondrial	ACADL	0.93±0.07	0.53±0.02	1.76
C2_10278	Long-chain-fatty-acid--CoA ligase 5	ACSL5	1.33±0.15	1.43±0.09	0.93
C2_12320	Long-chain-fatty-acid--CoA ligase ACSBG2	ACSBG2	1.13±0.05	0.69±0.08	1.64
C2_57935	Low molecular weight phosphotyrosine protein phosphatase	ACP1	1.19±0.12	1.24±0.05	0.96
C2_18370	Low-density lipoprotein receptor-related protein 2	LRP2	0.79±0.07	1.63±0.25	0.49
C2_8471	Lumican	LUM	2.13±0.7	3.66±0.51	0.58
C2_7694	Lupus La protein homolog B	SSB	0.95±0.09	2.09±0.34	0.45
C2_17468	L-xylulose reductase	DCXR	0.95±0.03	1.29±0.21	0.73
C2_29482	Lymphocyte antigen 75	LY75	0.98±0.04	0.8±0.04	1.23
C2_17901	Lymphocyte antigen 75	LY75	1.06±0.07	0.77±0.07	1.38
C2_9308	Lysine-specific demethylase NO66	C14orf169	1.02±0.05	0.99±0.04	1.04
C2_3934	Lysophosphatidic acid phosphatase type 6	ACP6	0.75±0.09	1.27±0.12	0.59
C2_14764	Lysophospholipase-like protein 1	LYPLAL1	0.83±0.12	0.76±0.06	1.09
C2_2497	Lysosomal acid phosphatase	ACP2	0.76±0.11	0.54±0.07	1.40
C2_27657	Lysosomal alpha-mannosidase	MAN2B1	1.25±0.24	0.89±0.02	1.41
C2_6582	Lysosomal alpha-mannosidase	MAN2B1	0.83±0.16	0.58±0.06	1.42
C2_8076	Lysosomal protective protein	CTSA	0.73±0.12	0.46±0.05	1.58
C2_2318	Lysosomal Pro-X carboxypeptidase	PRCP	0.69±0.17	0.34±0.1	2.03
C2_3622	Lysozyme g	LYG1	2.92±1.14	5.86±0.55	0.50
C2_3105	Lysyl-tRNA synthetase	KARS	1.7±0.32	1.93±0.04	0.88
C2_7220	MACRO domain-containing protein 1	MACROD1	1.05±0.1	1.08±0.03	0.97
C2_439	Macrophage migration inhibitory factor	MIF	0.88±0.15	0.63±0.07	1.39
C2_5305	Macrophage-capping protein	CAPG	1.16±0.13	1.35±0.07	0.86
C2_121640	Major histocompatibility complex class I-related gene protein	MR1	0.93±0.03	0.86±0.06	1.08
C2_72990	Major vault protein	MVP	2.35±0.62	3.76±0.42	0.63
C2_11452	Malate dehydrogenase, cytoplasmic	MDH1	2.23±0.53	2.53±0.04	0.88
C2_612	Malate dehydrogenase, mitochondrial	MDH2	1.21±0.08	0.66±0.06	1.83
C2_1108	Malignant T cell-amplified sequence 1-A	MCTS1	1.55±0.25	2.12±0.06	0.73
C2_1846	Maltase-glucoamylase, intestinal	MGAM	0.72±0.15	0.25±0.03	2.91
C2_4465	Mannose-1-phosphate guanyltransferase	GMPPA	0.85±0.06	1.06±0.09	0.80
C2_3546	Mannose-1-phosphate guanyltransferase alpha-A	GMPPA	1.05±0.06	1.17±0.06	0.89
C2_23669	Mannose-1-phosphate guanyltransferase beta	GMPPB	0.93±0.03	1.23±0.1	0.75
C2_19667	Mannose-6-phosphate isomerase	MPI	0.97±0.07	0.73±0.02	1.34
C2_1921	Mannose-P-dolichol utilization defect 1 protein	MPDU1	0.92±0.15	0.58±0.05	1.59
C2_27538	Mannosyl-oligosaccharide 1,2-alpha-mannosidase IA	MAN1A1	1.59±0.28	1.05±0.06	1.51
C2_3778	MAU2 chromatid cohesion factor homolog	MAU2	0.76±0.12	0.92±0.12	0.82
C2_1554	Medium-chain specific acyl-CoA dehydrogenase, mitochondrial	ACADM	0.82±0.1	0.32±0.04	2.55
C2_4129	Melanotransferrin	MFI2	0.67±0.13	0.35±0.04	1.93
C2_2487	Membrane-associated progesterone receptor component 1	PGRMC1	0.89±0.08	0.5±0.05	1.76
C2_1403	Meprin A subunit alpha	MEP1A	0.47±0.18	0.33±0.05	1.44
C2_5461	Meprin A subunit beta	MEP1B	0.55±0.15	0.29±0.04	1.88
C2_1481	Mesoderm-specific transcript homolog protein	MEST	1.13±0.08	0.65±0.1	1.75
C2_1799	Metaxin-2	MTX2	0.79±0.13	0.49±0.12	1.60
C2_9097	Methionine aminopeptidase 2	METAP2	1.1±0.09	1.2±0.08	0.92
C2_6737	Methionyl-tRNA synthetase, cytoplasmic	MARS	0.96±0.02	0.96±0.08	1.00
C2_5987	Methylmalonate-semialdehyde dehydrogenase [acylating], mitochondrial	ALDH6A1	1.62±0.3	0.62±0.03	2.60
C2_45829	Methylmalonyl-CoA epimerase, mitochondrial	MCEE	1.56±0.22	1.38±0.11	1.13
C2_1030	Microfibril-associated glycoprotein 4	MFAP4	1.01±0.07	0.94±0.07	1.08
C2_830	Microsomal glutathione S-transferase 3	MGST3	1.42±0.3	0.52±0.05	2.70
C2_68919	Microsomal triglyceride transfer protein large subunit	MTTP	2.49±0.57	0.95±0.03	2.62
C2_7905	Microsomal triglyceride transfer protein large subunit	MTTP	1.74±0.27	0.59±0.02	2.92
C2_18367	Microtubule-associated protein 1B	MAP1B	0.84±0.1	0.79±0.04	1.07
C2_15543	Microtubule-associated protein RP/EB family member 1	MAPRE1	1.04±0.09	1.66±0.05	0.62
C2_8412	Mitochondrial enolase superfamily member 1	ENOSF1	0.7±0.1	0.98±0.04	0.71
C2_198	Mitochondrial fission 1 protein	FIS1	1.37±0.21	1.89±0.03	0.73
C2_2920	Mitochondrial import inner membrane translocase subunit Tim13	TIMM13	1.28±0.18	0.89±0.1	1.43
C2_4825	Mitochondrial import receptor subunit TOM7 homolog	TOMM7	1.33±0.35	0.63±0.09	2.12
C2_658	Mitochondrial peptide methionine sulfoxide reductase	MSRA	0.94±0.08	0.83±0.06	1.14
C2_10488	Mitogen-activated protein kinase 1	MAPK1	1.11±0.09	1.64±0.12	0.68

C2_18375	Mitogen-activated protein kinase 12	MAPK12	0.82±0.12	1.2±0.02	0.69
C2_3688	Mitogen-activated protein kinase 14A	MAPK14	1±0.09	1.68±0.31	0.60
C2_96207	Mitogen-activated protein kinase ERK-A	MAPK1	1.43±0.27	3.1±0.18	0.46
C2_2990	Mitotic checkpoint protein BUB3	BUB3	1.05±0.05	1.15±0.07	0.91
C2_2663	Mob-like protein phocein	MOB4	1.12±0.11	0.76±0.03	1.47
C2_86	Moesin	MSN	1.24±0.2	1.93±0.04	0.64
C2_37874	Molybdenum cofactor sulfurase	MOCOS	0.82±0.09	0.82±0.1	1.01
C2_11306	Molybdopterin synthase catalytic subunit	MOCS2	0.82±0.07	0.66±0.07	1.24
C2_4226	Monoacylglycerol lipase ABHD12b	ABHD12b	1.37±0.14	1.08±0.13	1.27
C2_99049	Monofunctional C1-tetrahydrofolate synthase, mitochondrial	MTHFD1L	1±0.09	2.83±0.95	0.35
C2_6893	MOSC domain-containing protein 1, mitochondrial	MARC1	1.03±0.05	0.94±0.08	1.09
C2_2814	Motile sperm domain-containing protein 2	MOSPD2	1.13±0.06	0.73±0.04	1.54
C2_19684	Mps one binder kinase activator-like 1A	MOB1A	0.71±0.17	2.01±0.15	0.35
C2_2802	mRNA cap guanine-N7 methyltransferase	RNMT	2.24±0.44	1.77±0.18	1.27
C2_1832	mRNA export factor	RAE1	1.13±0.06	0.78±0.05	1.44
C2_33045	mRNA-decapping enzyme 1A	DCP1A	0.92±0.19	0.63±0.07	1.48
C2_1615	Mucin-13	MUC13	0.72±0.1	0.53±0.06	1.35
C2_3396	Mucin-2	MUC2	0.83±0.1	0.98±0.1	0.85
C2_22932	Mucin-2-like	MUC2	1.05±0.14	2.15±0.3	0.49
C2_40374	Mucin-5AC	MUC5AC	0.6±0.14	0.62±0.09	0.98
C2_3786	Multifunctional protein ADE2	PAICS	1.29±0.21	1.76±0.15	0.73
C2_5717	Multiple inositol polyphosphate phosphatase 1	MINPP1	1.28±0.17	2.04±0.33	0.63
FP338380	Murinoglobulin-1	Mug1	1.03±0.04	1.06±0.2	0.96
C2_6452	Myelin P2 protein	PMP2	1.88±0.39	1.97±0.2	0.96
C3_c20295	myelin protein zero-like protein 2-like isoform X2 [Maylandia zebra]	MPZL2	1.04±0.08	0.93±0.08	1.12
C2_11684	Myosin light chain kinase, smooth muscle	MYLK	1.24±0.1	0.93±0.05	1.33
C2_3500	Myosin light polypeptide 6	MYL6	1.77±0.34	2.67±0.2	0.66
C2_838	Myosin regulatory light chain 2, smooth muscle minor isoform	MYL12B	1.18±0.08	1.13±0.09	1.04
C2_25606	Myosin-10	MYH10	1.2±0.17	2.76±0.62	0.44
C2_19728	Myosin-9	MYH9	1.16±0.15	2.14±0.24	0.54
C2_28126	Myosin-Ic	MYO1C	2.26±0.94	8.68±1.43	0.26
C2_62971	Myosin-Ic	MYO1C	1.4±0.2	3.15±0.22	0.45
C2_65856	Myosin-Ic	MYO1C	0.86±0.08	1.49±0.13	0.58
C2_62194	Myosin-VI	MYO6	1.59±0.28	1.88±0.31	0.84
C2_24166	Myosin-VIIa	MYO7A	1.26±0.11	1.45±0.18	0.87
C2_11673	Myosin-VIIb	MYO7B	0.79±0.09	1.03±0.09	0.77
C2_56371	Myosin-XV	MYO15A	1.07±0.04	1.39±0.13	0.77
C2_23167	Myosin-XV	MYO15A	0.99±0.04	1.2±0.05	0.82
C2_3970	Myotrophin	MTPN	0.81±0.11	1.14±0.29	0.71
C2_4307	Myotubularin	MTM1	0.77±0.09	0.98±0.04	0.78
C2_16255	Myotubularin-related protein 10	MTMR10	0.79±0.07	1.01±0.21	0.78
C2_39243	Myotubularin-related protein 6	MTMR6	0.77±0.09	0.75±0.04	1.04
C2_8201	N(4)-(beta-N-acetylglucosaminyl)-L-asparaginase	AGA	0.64±0.13	0.48±0.1	1.33
C2_21024	N(G),N(G)-dimethylarginine dimethylaminohydrolase 1	DDAH1	0.93±0.04	0.65±0.05	1.41
C2_24043	Na(+)/H(+) exchange regulatory cofactor NHE-RF1	SLC9A3R1	1.13±0.05	2.43±0.35	0.47
C2_3136	Na(+)/H(+) exchange regulatory cofactor NHE-RF3	PDZK1	1.21±0.1	1.68±0.1	0.72
C2_70260	Na(+)/H(+) exchange regulatory cofactor NHE-RF3	PDZK1	0.71±0.1	0.93±0.07	0.77
C2_6312	N-acetylated-alpha-linked acidic dipeptidase-like protein	NAALADL1	0.75±0.1	0.83±0.08	0.91
C2_2369	N-acetyl-D-glucosamine kinase	NAGK	0.9±0.12	1.12±0.11	0.80
C2_14558	N-acetylgalactosamine kinase	GALK2	1.03±0.03	3.7±0.92	0.28
C2_32023	N-acetylgalactosaminyltransferase 7	GALNT7	1.12±0.07	0.66±0.05	1.68
C2_11912	N-acetylneuraminate lyase	NPL	0.73±0.11	0.72±0.07	1.02
C2_11122	NACHT, LRR and PYD domains-containing protein 12	NLRP12	0.91±0.13	0.67±0.13	1.36
C2_105885	NACHT, LRR and PYD domains-containing protein 3	NLRP3	4.86±1.62	5.31±0.73	0.92
C2_117333	NACHT, LRR and PYD domains-containing protein 6	NLRP6	0.95±0.08	1.07±0.13	0.89
C2_14416	N-acylethanolamine-hydrolyzing acid amidase	NAAA	0.59±0.14	0.49±0.05	1.21
C2_1737	N-acylglucosamine 2-epimerase	RENBP	0.95±0.02	1.75±0.06	0.55
C2_51310	N-acylneuraminate cytidylyltransferase	CMAS	0.83±0.1	0.38±0.08	2.18
C2_13180	N-acylneuraminate-9-phosphatase	NANP	0.94±0.06	0.89±0.03	1.05
C2_2102	N-acyl-phosphatidylethanolamine-hydrolyzing phospholipase D	NAPEPLD	0.89±0.05	0.64±0.04	1.40
C2_4705	NAD-dependent malic enzyme, mitochondrial	ME2	1.09±0.05	0.62±0.02	1.75
C2_5740	NADH dehydrogenase [ubiquinone] 1 subunit C1, mitochondrial	NDUFC1	1.25±0.1	0.8±0.08	1.56
C2_357	NADH-cytochrome b5 reductase 2	CYB5R2	1.31±0.13	0.66±0.05	1.97
C2_568	NADH-cytochrome b5 reductase 3	Cyb5r3	1.69±0.37	1.11±0.09	1.51
C2_3984	NADP-dependent malic enzyme	ME1	2.25±0.48	1.45±0.05	1.55
C2_4956	NADP-dependent malic enzyme, mitochondrial	ME3	1.75±0.35	0.84±0.05	2.08

C2_2964	NADPH--cytochrome P450 reductase	POR	1.18±0.07	0.58±0.01	2.03
C2_9509	N-alpha-acetyltransferase 38, NatC auxiliary subunit	NAA38	0.81±0.08	0.64±0.1	1.26
C2_339	Nascent polypeptide-associated complex subunit alpha, muscle-specific form	NACA	1.54±0.26	2.1±0.19	0.73
C2_16315	Natterin-3	NATT3	0.71±0.1	0.65±0.07	1.10
C2_41935	Nck-associated protein 1	NCKAP1	1.2±0.11	1.8±0.37	0.67
C2_30543	Nck-associated protein 1	NCKAP1	0.97±0.09	0.89±0.02	1.09
C2_8636	NEDD4-like E3 ubiquitin-protein ligase WWP1	WWP1	1.04±0.11	1.19±0.07	0.87
C2_3449	NEDD8-conjugating enzyme UBE2F	UBE2F	1.73±0.3	3.31±0.53	0.52
C2_10265	Neudesin	NENF	1.41±0.19	1.12±0.16	1.26
C3_c46743	Neural Wiskott-Aldrich syndrome protein	Wasl	1.37±0.32	3.54±0.2	0.39
C2_43416	Neutral alpha-glucosidase AB	GANAB	1.04±0.19	0.49±0.03	2.11
C2_4237	Neutral alpha-glucosidase AB	GANAB	1±0.11	0.45±0.03	2.20
C2_4635	Neutral and basic amino acid transport protein rBAT	SLC3A1	0.84±0.12	0.55±0.05	1.51
C2_14365	Neutral ceramidase	ASAH2	0.69±0.11	0.41±0.04	1.70
C2_18906	Neutral ceramidase	ASAH2	0.67±0.11	0.31±0.03	2.18
C2_1117	Neutral cholesterol ester hydrolase 1	NCEH1	0.71±0.1	0.52±0.04	1.37
C2_57343	NFATC2-interacting protein	NFATC2IP	1.74±0.27	1.06±0.36	1.64
C2_26757	NHL repeat-containing protein 3	NHLRC3	0.71±0.12	0.57±0.03	1.25
C2_2429	NHP2-like protein 1	NHP2L1	1.06±0.12	0.96±0.06	1.11
C2_5958	Nicastrin	NCSTN	0.94±0.14	0.51±0.05	1.84
C2_1457	Nicotinamide phosphoribosyltransferase	NAMPT	1.31±0.13	1.72±0.42	0.76
C2_21618	Niemann-Pick C1-like protein 1	NPC1L1	1.52±0.22	1.06±0.09	1.43
C2_4414	Niemann-Pick C1-like protein 1	NPC1L1	0.58±0.15	0.38±0.07	1.50
C2_4636	NIF3-like protein 1	CTDSP1	0.59±0.14	0.32±0.03	1.84
C2_22874	Nitrilase homolog 1	NIT1	0.85±0.06	1.21±0.07	0.70
C2_781	N-myc-interactor	NMI	0.75±0.1	0.63±0.02	1.19
C2_76382	Nodal modulator 1	NOMO1	0.89±0.1	0.42±0.02	2.13
C2_75156	Nodal modulator 2	NOMO1	0.85±0.09	0.38±0.03	2.25
C2_14786	Non-lysosomal glucosylceramidase	GBA2	1.08±0.07	1.04±0.16	1.04
C2_183	Non-specific cytotoxic cell receptor protein 1 homolog	NCCRP1	0.91±0.14	0.43±0.04	2.12
C2_121344	Non-specific lipid-transfer protein	SCP2	1.23±0.14	1±0.05	1.24
C2_1496	Non-specific lipid-transfer protein	SCP2	1.03±0.14	0.65±0.07	1.57
C2_5466	NSFL1 cofactor p47	NSFL1C	0.94±0.17	0.94±0.04	1.00
C2_44175	Nuclear cap-binding protein subunit 1	NCBP1	1.88±0.43	3.2±0.28	0.59
C2_3542	Nuclear export mediator factor Nemf	NEMF	1.23±0.15	1.15±0.09	1.07
C2_14783	Nuclear factor 1 C-type	NFIC	4.11±1.1	2.81±0.34	1.46
C2_8048	Nuclear factor 7, brain	ARNTL2	1.95±0.44	3.23±0.13	0.60
C2_20656	Nuclear factor 7, ovary	EEF1A1	1.35±0.16	1.79±0.04	0.76
C2_82048	Nuclear factor NF-kappa-B p105 subunit	NFKB1	0.74±0.09	0.91±0.07	0.82
C2_1121	Nuclear migration protein nudC	NUDC	0.75±0.09	0.78±0.07	0.96
C2_25773	Nuclear pore complex protein Nup54	NUP54	0.93±0.04	0.82±0.09	1.13
C2_6152	Nuclear pore complex protein Nup85	NUP85	1.25±0.11	1.16±0.12	1.08
C2_14771	Nuclear pore glycoprotein p62	NUP62	0.54±0.15	0.44±0.05	1.25
C2_10636	Nuclear protein localization protein 4 homolog	NPLOC4	1.25±0.15	1.39±0.12	0.90
C2_9241	Nuclear receptor-binding protein	NRBP1	0.65±0.14	0.48±0.05	1.34
C2_493	Nuclear transport factor 2	NUTF2	0.77±0.08	0.88±0.15	0.88
C2_14540	Nucleobindin-2	NUCB2	2.38±0.55	2.05±0.17	1.16
C2_206	Nucleolar protein 56	NOP56	1.01±0.06	0.77±0.07	1.31
C2_769	Nucleolar protein 58	NOP58	1.26±0.17	1.56±0.19	0.81
C2_9442	Nucleolin	NCL	0.86±0.06	1.92±0.62	0.45
C2_27287	Nucleolysin TIA-1 isoform p40	TIA1	0.83±0.11	0.57±0.02	1.47
C2_2403	Nucleophosmin	NPM1	0.91±0.11	0.79±0.1	1.15
C2_20392	Nucleoredoxin	NXN	0.82±0.06	0.88±0.02	0.93
C2_113264	Nucleoside diphosphate kinase A1	NME1-1	1.88±0.43	1.59±0.08	1.18
C2_1536	Nucleoside diphosphate kinase B	NME2	0.93±0.11	0.98±0.1	0.94
C2_3380	Nucleosome assembly protein 1-like 1	NAP1L1	0.85±0.07	0.59±0.06	1.45
C2_19448	Nucleosome assembly protein 1-like 4	NAP1L4	1.08±0.08	1.2±0.05	0.90
C2_5052	Obg-like ATPase 1	OLA1	1.08±0.11	2.72±1.08	0.40
C2_5698	OCIA domain-containing protein 2	OCIAD2	1.56±0.4	0.86±0.07	1.82
C2_8505	Olfactomedin-4	OLFM4	0.67±0.12	0.42±0.04	1.59
C2_604	Oligoribonuclease, mitochondrial	REXO2	2.43±0.88	4.58±0.33	0.53
C2_5167	Omega-amidase NIT2	NIT2	1.01±0.05	1.06±0.04	0.95
C2_6267	O-phosphoseryl-tRNA(Sec) selenium transferase	SEPSECS	1.03±0.04	1.34±0.02	0.76
C2_846	Osteoclast-stimulating factor 1	OSTF1	1.01±0.02	1.52±0.33	0.66
C2_19895	Otoraplin	OTOR	0.97±0.09	0.78±0.14	1.24
C2_35266	Oxidoreductase HTATIP2	HTATIP2	1.05±0.06	1.24±0.12	0.84

C2_6623	Oxysterol-binding protein-related protein 3	OSBPL3	1.05±0.06	1.9±0.36	0.55
C2_557	Palmitoyl-protein thioesterase 1	PPT1	0.88±0.11	0.55±0.07	1.60
C2_3526	Pancreatic alpha-amylase	AMY2B	0.6±0.16	0.19±0.04	3.17
C2_6860	Pantothenate kinase 1	PANK1	1.17±0.11	1.63±0.11	0.72
C2_71084	Papilin	PAPLN	2.67±0.97	1.32±0.32	2.03
C2_119529	Parvalbumin alpha	PVALB	0.72±0.1	0.61±0.07	1.18
C2_592	Parvalbumin beta	PRVB	0.79±0.08	1.23±0.26	0.64
C2_120726	Parvalbumin beta 2	PRVB2	0.7±0.11	0.69±0.11	1.02
C2_50293	Parvalbumin beta 2	PRVB2	0.72±0.1	0.49±0.05	1.46
C2_2591	PCI domain-containing protein 2	PCID2	0.94±0.1	0.59±0.05	1.59
C2_5258	PDZ and LIM domain protein 1	PDLIM1	2.18±0.51	2.53±0.21	0.86
C2_6912	PDZ and LIM domain protein 5	PDLIM5	2.42±0.53	1.85±0.23	1.31
C2_8807	PDZ domain-containing protein GIPC1	GIPC1	0.89±0.13	1.74±0.06	0.51
C2_9030	Pentraxin fusion protein	PXN1	0.83±0.06	1±0.16	0.83
C2_16238	Peptide-N(4)-(N-acetyl-beta-D-glucosaminyl)asparagine amidase F	NGLY1	1.41±0.27	0.93±0.17	1.51
C2_6168	Peptide-N(4)-(N-acetyl-beta-glucosaminyl)asparagine amidase	NGLY1	0.96±0.1	1.14±0.09	0.84
C2_1296	Peptidyl-prolyl cis-trans isomerase	FKBP4	1.89±0.38	2.79±0.05	0.68
C2_428	Peptidyl-prolyl cis-trans isomerase	FKBP4	1.67±0.26	1.6±0.08	1.05
C2_1675	Peptidyl-prolyl cis-trans isomerase B	PPIB	2.09±0.53	2.23±0.07	0.93
C2_13338	Peptidyl-prolyl cis-trans isomerase D	PPID	0.86±0.07	0.58±0.04	1.48
C2_8702	Peptidyl-prolyl cis-trans isomerase FKBP1A	FKBP1A	1.42±0.27	2.16±0.13	0.66
C2_472	Peptidyl-prolyl cis-trans isomerase H	PPIH	1.04±0.02	0.92±0.12	1.13
C2_8872	Peptidyl-prolyl cis-trans isomerase NIMA-interacting 1	PIN1	1.73±0.43	3.06±0.09	0.57
C2_466	Peroxiredoxin-1	PRDX1	1.01±0.05	0.57±0.04	1.76
C2_10204	Peroxiredoxin-2	PRDX2	0.82±0.1	0.48±0.02	1.70
C2_579	Peroxiredoxin-4	PRDX4	0.78±0.12	0.28±0.03	2.77
C2_4821	Peroxiredoxin-5, mitochondrial	PRDX5	1.08±0.1	1.55±0.18	0.70
C2_199	Peroxiredoxin-6	PRDX6	1.15±0.13	2.8±1.05	0.41
C2_8400	Peroxisomal 2,4-dienoyl-CoA reductase	DECR2	1.05±0.05	0.77±0.07	1.35
C2_62152	Peroxisomal acyl-coenzyme A oxidase 3	ACOX3	2.59±0.57	0.58±0.07	4.50
C2_30152	Peroxisomal carnitine O-octanoyltransferase	CROT	0.92±0.08	0.59±0.02	1.55
C2_4577	Peroxisomal multifunctional enzyme type 2	HSD17B4	1.22±0.09	0.53±0.03	2.32
C2_4281	Peroxisomal N(1)-acetyl-spermine/spermidine oxidase	PAOX	1.36±0.17	0.88±0.04	1.54
C2_9766	Peroxisomal sarcosine oxidase	PIPOX	1.64±0.24	0.86±0.06	1.91
C2_50894	Peroxisomal targeting signal 2 receptor	PEX7	1.22±0.25	1.07±0.24	1.15
C2_4069	Phenazine biosynthesis-like domain-containing protein 1	PBLD	0.79±0.11	0.58±0.05	1.36
C2_2121	Phenylalanyl-tRNA synthetase alpha chain	FARSA	1.23±0.25	2.19±0.24	0.56
C2_2810	Phenylalanyl-tRNA synthetase beta chain	FARSB	1.35±0.15	2.26±0.15	0.60
C2_931	Phosducin-like protein 3	PDCL3	1.11±0.08	1.46±0.03	0.76
C2_3888	Phosphatidylcholine transfer protein	PCTP	0.85±0.09	1.29±0.08	0.66
C2_14981	Phosphatidylcholine-sterol acyltransferase	LCAT	1±0.06	0.86±0.01	1.15
C2_99	Phosphatidylethanolamine-binding protein 1	PEBP1	1.7±0.38	2.46±0.12	0.69
C2_4295	Phosphatidylinositide phosphatase SAC1-B	SACM1L	1.38±0.25	0.68±0.05	2.03
C2_14866	Phosphatidylinositol 3-kinase catalytic subunit type 3	PIK3C3	0.75±0.09	0.57±0.03	1.32
C2_18561	Phosphatidylinositol 3-kinase regulatory subunit alpha	PIK3R1	0.75±0.12	0.86±0.16	0.87
C2_1495	Phosphatidylinositol transfer protein alpha isoform	PITPNA	1.58±0.26	3.96±0.62	0.40
C2_5143	Phosphatidylinositol-4-phosphate 3-kinase C2 domain-containing subunit alpha	PIK3C2A	1.04±0.15	1.54±0.41	0.67
C2_8586	Phosphatidylinositol-binding clathrin assembly protein	PICALM	0.84±0.05	0.77±0.03	1.10
C2_19167	Phosphoacetylglucosamine mutase	PGM3	1.61±0.26	1.68±0.13	0.96
C2_12073	Phosphoenolpyruvate carboxykinase [GTP], mitochondrial	PCK2	1.82±0.33	0.68±0.03	2.67
C2_371	Phosphoglucomutase-1	PGM1	1.31±0.26	1.25±0.17	1.05
C2_6725	Phosphoglucomutase-2	PGM2	0.88±0.04	0.83±0.07	1.06
C2_12228	Phosphoglucomutase-like protein 5	PGM5	0.98±0.02	0.94±0.11	1.04
C2_1379	Phosphoglycerate kinase 1	PGK1	0.96±0.03	0.94±0.05	1.02
C2_2366	Phosphoglycerate mutase 1	PGAM1	1.27±0.18	2.69±0.1	0.47
C2_101	Phosphoglycerate mutase 2	PGAM2	0.73±0.1	0.75±0.12	0.97
C2_1462	Phospholipase A2 group 1B	PLA2G1B	1.11±0.05	5.78±0.69	0.19
C2_40269	Phospholipase A-2-activating protein	PLAA	0.75±0.12	0.42±0.04	1.78
C2_24128	Phospholipase B1, membrane-associated	PLB1	0.88±0.05	1.88±0.19	0.47
C2_12635	Phospholipase B1, membrane-associated	PLB1	0.84±0.06	1.4±0.17	0.60
C3_c15747	Phospholipase B1, membrane-associated	PLB1	0.67±0.11	0.44±0.02	1.52
C2_4374	Phospholipase B-like 1	PLBD1	0.78±0.11	0.58±0.04	1.34
C2_85	Phospholipid hydroperoxide glutathione peroxidase, mitochondrial	GPX4	0.75±0.11	0.26±0.01	2.91
C2_45244	Phospholipid transfer protein	PLTP	1.05±0.13	0.81±0.18	1.29
C2_1982	Phosphomannomutase 2	PMM2	1.03±0.05	0.77±0.05	1.33

C2_2665	Phosphopantothenate--cysteine ligase	PPCS	0.91±0.06	1.42±0.09	0.64
C2_1196	Phosphopantothenoylcysteine decarboxylase	PPCDC	0.87±0.04	0.86±0.07	1.01
C2_20987	Phosphoribosyl pyrophosphate synthase-associated protein 1	PRPSAP1	0.89±0.17	1±0.1	0.89
C2_19159	Phosphotriesterase-related protein	PTER	0.72±0.13	0.66±0.06	1.09
C2_31716	Phytanoyl-CoA dioxygenase domain-containing protein 1	PHYHD1	1.03±0.05	2.16±0.36	0.48
C2_7139	PITH domain-containing protein 1	PITHD1	0.78±0.12	0.58±0.05	1.34
C2_13726	Plakophilin-3	PKP3	1.02±0.06	1.93±0.78	0.53
C2_3079	Plasma kallikrein	KLKB1	1.23±0.23	1.25±0.07	0.98
C2_13716	Plasma membrane calcium-transporting ATPase 1	ATP2B1	1.47±0.18	2.35±0.16	0.62
C2_23687	Plasma protease C1 inhibitor	SERPING1	0.94±0.07	1.08±0.2	0.88
C2_26329	Plasma protease C1 inhibitor	SERPING1	0.97±0.09	0.99±0.18	0.99
C2_4248	Plastin-1	PLS1	1.3±0.15	2.46±0.13	0.53
C2_952	Plastin-2	LCP1	0.99±0.05	1±0.09	0.99
C2_6788	Plastin-3	PLS3	0.78±0.09	0.6±0.04	1.29
C2_5230	Platelet glycoprotein 4	CD36	0.66±0.13	0.35±0.04	1.88
C2_10874	Platelet glycoprotein Ib alpha chain	GP1BA	1.49±0.19	1.47±0.23	1.01
C2_10639	Platelet-activating factor acetylhydrolase	PLA2G7	1.51±0.23	1.18±0.07	1.28
C2_2316	Platelet-activating factor acetylhydrolase IB subunit gamma	PAFAH1B3	1.07±0.05	1.32±0.11	0.81
C2_21485	Plectin	PLEC	0.94±0.04	1.08±0.06	0.87
C2_3910	Plectin	PLEC	1.09±0.14	1.02±0.09	1.07
C3_c26450	Plectin	PLEC	0.87±0.05	0.68±0.11	1.27
C2_13614	Plectin	PLEC	0.9±0.13	0.69±0.02	1.31
C3_c30637	Plectin	PLEC	0.99±0.09	0.59±0.02	1.69
C2_16967	Plectin	PLEC	1.43±0.26	0.57±0.06	2.50
C2_2925	Pleiotropic regulator 1	PLRG1	0.84±0.11	0.54±0.06	1.57
C2_11515	Poly [ADP-ribose] polymerase 1	PARP1	1.33±0.15	1.47±0.05	0.90
C2_25980	Poly [ADP-ribose] polymerase 1	PARP1	0.78±0.1	0.8±0.1	0.97
C2_33629	Poly [ADP-ribose] polymerase 10	PARP10	0.94±0.08	0.65±0.08	1.46
C2_4201	Poly [ADP-ribose] polymerase 3	PARP3	0.76±0.09	0.82±0.11	0.93
C2_18821	Poly [ADP-ribose] polymerase 9	PARP9	0.83±0.09	0.81±0.1	1.02
C2_38350	Poly(ADP-ribose) glycohydrolase ARH3	ADPRHL2	0.99±0.02	0.9±0.04	1.09
C2_4828	Poly(rC)-binding protein 2	PCBP2	1.49±0.2	3.19±0.6	0.47
C2_2960	Poly(U)-binding-splicing factor PUF60	PUF60	0.78±0.08	0.61±0.06	1.27
C2_54396	Poly(U)-binding-splicing factor PUF60-B	PUF60-B	0.87±0.05	0.86±0.05	1.02
C2_6459	Poly(U)-specific endoribonuclease	ENDOU	0.59±0.14	0.4±0.05	1.48
C2_3168	Polyadenylate-binding protein 1	PABPC1	1.03±0.17	1.77±0.24	0.58
C2_12680	Polyadenylate-binding protein 2	PABPN1	0.99±0.03	0.93±0.05	1.07
C2_9708	Polypeptide N-acetylgalactosaminyltransferase 6	GALNT6	0.91±0.05	0.55±0.03	1.65
C2_8361	Polypyrimidine tract-binding protein 1	PTBP1	1.1±0.12	1.54±0.09	0.72
C2_3549	Polypyrimidine tract-binding protein 2	PTBP2	1.48±0.19	3.43±0.46	0.43
C2_6689	Polyserase-2	PRSS36	0.39±0.2	0.76±0.16	0.51
C2_4566	Porphobilinogen deaminase	HMBS	1.09±0.07	1.07±0.04	1.02
C2_1110	Prefoldin subunit 1	PFDN1	0.78±0.08	1.25±0.17	0.62
C2_653	Prefoldin subunit 2	PFDN2	0.96±0.03	1.46±0.24	0.66
C2_120921	Prefoldin subunit 3	VBP1	0.98±0.03	1.23±0.07	0.80
C2_4841	Prefoldin subunit 4	PFDN4	1.61±0.42	3.05±0.41	0.53
C2_473	Prefoldin subunit 5	PFDN5	1.11±0.08	1.27±0.04	0.87
C2_41817	Pregnancy zone protein	PZP	0.87±0.07	0.88±0.15	0.99
C2_17370	Pregnancy-specific beta-1-glycoprotein 8	PSG8	0.72±0.12	0.84±0.09	0.85
C2_17155	Prelamin-A/C	LMNA	1.18±0.2	1.24±0.21	0.95
C2_3804	Pre-mRNA-processing factor 39	PRPF39	1.22±0.15	1.74±0.17	0.70
C2_15123	Pre-mRNA-processing-splicing factor 8	PRPF8	1.25±0.13	1.5±0.12	0.83
C2_9221	Proactivator polypeptide	PSAPL1	1.02±0.13	0.56±0.09	1.84
C2_22286	Probable 2-ketogluconate reductase	tkrA	1.22±0.12	1.79±0.29	0.68
C2_18944	Probable 4-hydroxy-2-oxoglutarate aldolase, mitochondrial	HOGA1	0.71±0.1	0.59±0.08	1.20
C2_1677	Probable acyl coa dehydrogenase 6	acdh-6	1.21±0.08	1.06±0.06	1.14
C2_1682	Probable aminopeptidase NPEPL1	NPEPL1	0.78±0.13	0.57±0.05	1.37
C2_8194	Probable arylformamidase	FP0207	1.02±0.03	1.69±0.03	0.60
C2_68498	Probable ATP-dependent helicase lhr	TMBIM6	1.43±0.29	1.51±0.22	0.94
C2_2903	Probable ATP-dependent RNA helicase DDX23	DDX23	1.13±0.18	1.7±0.2	0.67
C2_2745	Probable ATP-dependent RNA helicase ddx6	DDX6	0.97±0.02	1.28±0.06	0.76
C2_4290	Probable carboxypeptidase PM20D1.2	PM20D1	0.84±0.09	0.55±0.02	1.54
C2_26495	Probable D-tyrosyl-tRNA(Tyr) deacylase 2	DTD2	1.08±0.06	1.06±0.06	1.02
C2_64430	Probable E3 ubiquitin-protein ligase MYCBP2	MYCB2	1.01±0.1	0.7±0.26	1.45
C2_13444	Probable E3 ubiquitin-protein ligase TRIP12	TRIP12	0.94±0.06	0.89±0.07	1.06
C2_6533	Probable ergosterol biosynthetic protein 28	C14orf1	1.59±0.21	1.24±0.15	1.28
C2_31924	Probable hydrolase PNKD	PNKD	0.96±0.1	0.76±0.07	1.26

C2_1359	Probable methylthioribulose-1-phosphate dehydratase	mtnAB	0.82±0.11	0.51±0.05	1.61
C2_20669	Probable peptidyl-tRNA hydrolase 2	pth2	0.86±0.1	0.69±0.04	1.25
C2_25307	Probable protein BRICK1	BRK1	0.91±0.14	2.11±0.37	0.43
C2_1150	Probable serine carboxypeptidase CPVL	CPVL	0.77±0.13	0.86±0.17	0.90
C2_242	Probable signal peptidase complex subunit 2	SPCS2	1.22±0.11	0.76±0.11	1.61
C2_11771	Probable thiopurine S-methyltransferase	TPMT	1.44±0.18	2.27±0.07	0.63
C2_8372	Probable threonyl-tRNA synthetase 2, cytoplasmic	TARSL2	1.26±0.1	1.13±0.03	1.11
C2_1594	Probable tRNA threonylcarbamoyladenosine biosynthesis protein osgep	OSGEPL1	1.06±0.06	1.82±0.29	0.58
C2_29865	Probable ubiquitin carboxyl-terminal hydrolase FAF-X	USP9X	1.36±0.17	1.9±0.19	0.72
C2_9255	Probable uridine nucleosidase 1	URH1	0.63±0.13	0.32±0.03	1.94
C2_7946	Profilin-2	PFN2	0.76±0.1	0.48±0.05	1.59
C2_22586	Programmed cell death 6-interacting protein	PDCD6IP	1.44±0.28	2.42±0.15	0.59
C2_20093	Programmed cell death protein 4	PDCD4	1.37±0.14	1.77±0.29	0.77
C2_576	Programmed cell death protein 6	PDCD6	1.09±0.03	0.94±0.11	1.16
C2_285	Prohibitin	PHB	1.28±0.27	0.37±0.07	3.43
C2_1796	Prolactin regulatory element-binding protein	PREB	1.13±0.15	0.47±0.05	2.39
C2_228	Proliferating cell nuclear antigen	PCNA	0.8±0.07	0.55±0.05	1.46
C2_447	Proliferation-associated protein 2G4	PA2G4	1.36±0.22	1.61±0.07	0.85
C2_816	Proline synthase co-transcribed bacterial homolog protein	PROSC	1.43±0.21	1.5±0.05	0.95
C2_35352	Prolyl endopeptidase	PREP	0.98±0.05	0.78±0.06	1.26
C2_19946	Prolyl endopeptidase	PREP	0.78±0.08	0.57±0.06	1.36
C2_45784	Prominin-1	PROM1	1.91±0.44	5.11±0.1	0.37
C2_6056	Propionyl-CoA carboxylase beta chain, mitochondrial	PCCB	0.61±0.14	0.65±0.09	0.94
C2_18955	Prostaglandin E synthase 2	PTGES2	0.96±0.06	0.77±0.05	1.24
C2_340	Prostaglandin E synthase 3	PTGES3	0.79±0.07	0.61±0.05	1.29
C2_69618	Prostaglandin reductase 1	PTGR1	1.8±0.37	0.56±0.04	3.22
C2_40152	Prostaglandin reductase 2	PTGR2	0.9±0.08	1.09±0.17	0.82
C2_6696	Prostaglandin-H2 D-isomerase	PTGDS	0.93±0.05	1.02±0.05	0.91
C2_4229	Prostamide/prostaglandin F synthase	FAM213B	1.04±0.05	1.76±0.21	0.59
C2_2509	Prostasin	PRSS8	0.37±0.21	0.71±0.2	0.52
C2_62037	Proteasomal ubiquitin receptor ADRM1	ADRM1	1.38±0.25	2.29±0.12	0.60
C2_19056	Proteasome activator complex subunit 1	PSME1	1.14±0.11	2.24±0.81	0.51
C2_52053	Proteasome activator complex subunit 2	PSME2	1.44±0.22	2.41±0.57	0.59
C2_8230	Proteasome assembly chaperone 2	PSMG2	0.57±0.17	0.49±0.08	1.17
C2_1050	Proteasome assembly chaperone 3	PSMG3	1.09±0.04	1.63±0.07	0.67
C2_276	Proteasome subunit alpha type-1	PSMA1	0.8±0.11	0.41±0.05	1.97
C2_39253	Proteasome subunit alpha type-2	PSMA2	0.85±0.11	0.43±0.05	1.99
C2_667	Proteasome subunit alpha type-3	PSMA3	0.84±0.14	0.38±0.05	2.17
C2_1506	Proteasome subunit alpha type-4	PSMA4	0.8±0.13	0.41±0.04	1.96
C2_89	Proteasome subunit alpha type-5	PSMA5	0.77±0.12	0.37±0.03	2.07
C2_979	Proteasome subunit alpha type-6	PSMA6	0.78±0.1	0.47±0.05	1.67
C2_486	Proteasome subunit alpha type-7	PSMA7	0.82±0.12	0.43±0.02	1.89
C2_303	Proteasome subunit beta type-1-B	PSMA1	0.79±0.11	0.41±0.05	1.94
C2_4220	Proteasome subunit beta type-2	PSMB2	0.8±0.12	0.43±0.04	1.87
C2_1113	Proteasome subunit beta type-3	PSMB3	0.89±0.16	0.43±0.05	2.07
C2_1989	Proteasome subunit beta type-4	PSMB4	0.84±0.14	0.41±0.04	2.04
C2_2719	Proteasome subunit beta type-5	PSMB5	0.93±0.09	0.58±0.04	1.62
C2_43253	Proteasome subunit beta type-6	PSMB6	1.12±0.11	0.59±0.07	1.88
C2_104936	Proteasome subunit beta type-6-B like protein	PSMB6L-B	0.83±0.1	0.52±0.04	1.59
C2_909	Proteasome subunit beta type-7	PSMB7	1.11±0.1	0.94±0.08	1.19
C2_21676	Proteasome subunit beta type-8	PSMB8	0.69±0.12	0.56±0.07	1.24
C2_121103	Proteasome subunit beta type-9	PSMB9	0.82±0.1	0.48±0.04	1.71
C2_2075	Protein archease	ZBTB8OS	1.04±0.04	1.08±0.07	0.96
C2_2985	Protein arginine N-methyltransferase 1	PRMT1	1.75±0.28	1.6±0.12	1.09
C2_5961	Protein argonaute-2	AGO2	1.16±0.1	1.58±0.11	0.73
C2_247	Protein C17orf37 homolog	MIEN1	0.67±0.12	0.5±0.05	1.35
C2_1344	Protein Churchill	CHURC1	0.79±0.07	0.63±0.04	1.24
C2_2192	Protein cornichon homolog 4	CNIH4	0.88±0.08	0.65±0.06	1.35
C2_17089	Protein diaphanous homolog 1	DIAPH1	0.94±0.03	1.1±0.06	0.85
C2_17097	Protein diaphanous homolog 1	DIAPH1	0.82±0.1	0.31±0.01	2.61
C2_3894	Protein disulfide-isomerase	P4HB	1.57±0.2	0.46±0.03	3.43
C2_251	Protein disulfide-isomerase A3	PDIA3	1.39±0.17	0.53±0.06	2.61
C2_2492	Protein disulfide-isomerase A4	PDIA4	1.26±0.11	0.6±0.04	2.11
C2_10370	Protein disulfide-isomerase A6	PDIA6	1.36±0.14	0.89±0.02	1.54
C3_c46794	protein disulfide-isomerase-like [Oryzias latipes]	P4HB	1.55±0.19	0.44±0.08	3.55
C2_229	Protein DJ-1	PARK7	1.03±0.04	2.03±0.1	0.50

C2_18250	Protein dopey-2	DOPEY2	0.69±0.12	0.94±0.11	0.73
C2_25777	Protein dpy-30 homolog	DPY30	1.19±0.17	1.96±0.17	0.61
C2_3442	Protein EFR3 homolog A	EFR3A	1.43±0.2	2.1±0.34	0.68
C2_3833	Protein ERGIC-53	LMAN1	1.07±0.06	0.83±0.05	1.29
C2_1216	Protein ETHE1, mitochondrial	ETHE1	1.27±0.11	2.05±0.18	0.62
C2_41607	Protein FAM115	FAM115C	1.15±0.08	0.91±0.14	1.26
C2_6080	Protein FAM115	FAM115C	0.83±0.13	0.57±0.03	1.46
C2_6182	Protein FAM151A	FAM151A	0.71±0.11	0.62±0.09	1.15
C2_6067	Protein FAM203A	HGH1	0.87±0.05	0.97±0.05	0.90
C2_9945	Protein FAM45A	FAM45A	0.98±0.2	1.14±0.39	0.86
C2_1887	Protein FAM49B	FAM49B	0.98±0.05	1.74±0.29	0.56
C2_11022	Protein farnesyltransferase subunit beta	FNTB	1.53±0.27	1.65±0.08	0.93
C2_1062	Protein farnesyltransferase/geranylgeranyltransferase type-1 subunit alpha	FNTA	0.98±0.02	0.96±0.03	1.02
C2_18576	Protein flightless-1 homolog	FLII	0.67±0.11	0.62±0.02	1.09
C2_2924	Protein HEG homolog 1	HEG1	0.85±0.05	1.17±0.04	0.73
C2_25893	Protein KHNYN	KHNYN	1.35±0.17	1.54±0.15	0.88
C2_28019	Protein kinase C and casein kinase substrate in neurons protein 1	PACSIN1	1.92±0.51	2.2±0.15	0.87
C2_2899	Protein kish-A	TMEM167A	1.1±0.05	1.25±0.2	0.88
C2_46645	Protein LAP2	ERBB2IP	0.88±0.06	1.03±0.1	0.85
C2_32269	Protein LAP2	ERBB2IP	1.06±0.07	1.2±0.07	0.89
C2_158	Protein LSM12 homolog A	LSM12	1.35±0.77	1.55±0.51	0.87
C2_24702	Protein mago nashi homolog 2	MAGOHB	1.63±0.24	1.41±0.08	1.16
C2_11790	Protein MEMO1	MEMO1	0.82±0.06	0.72±0.04	1.14
C2_20871	Protein MON2 homolog	MON2	1.16±0.15	1.89±0.07	0.61
C2_1083	Protein NDRG1	NDRG1	1.37±0.21	1.48±0.14	0.93
C2_2272	Protein NipSnap homolog 3A	NIPSNAP3A	1.08±0.04	0.68±0.05	1.58
C2_4429	Protein N-terminal asparagine amidohydrolase	NTAN1	0.99±0.01	0.77±0.03	1.28
C2_1971	Protein phosphatase 1 regulatory subunit 7	PPP1R7	1±0.06	1.18±0.04	0.85
C2_8910	Protein phosphatase 1A	PPM1A	1.17±0.17	1.96±0.39	0.60
C2_4186	Protein phosphatase Slingshot homolog	SSH1	0.97±0.05	0.92±0.05	1.05
C2_52239	Protein PRRC1	PRRC1	1.04±0.06	0.82±0.03	1.27
C2_8306	Protein prune homolog	PRUNE	0.89±0.12	1.37±0.08	0.65
C2_2956	Protein RER1	RER1	1.05±0.07	0.62±0.02	1.71
C2_47184	Protein rogdi homolog	ROGDI	0.71±0.12	0.73±0.07	0.97
C2_10121	Protein RUFY3	RUFY3	0.88±0.09	1.38±0.29	0.64
C2_1280	Protein S100-A10	S100A10	1.78±0.58	2.8±0.64	0.64
C2_19606	Protein S100-A14	S100A14	0.74±0.09	0.65±0.04	1.13
C2_111465	Protein S100-P	S100P	1.32±0.14	1.16±0.1	1.15
C2_4144	Protein SEC13 homolog	SEC13	0.8±0.07	0.36±0.04	2.21
C2_8005	Protein SET	SET	0.84±0.07	0.6±0.05	1.39
C2_3704	Protein TEX261	TEX261	2.03±0.6	0.75±0.1	2.72
C2_90346	Protein translation factor SUI1 homolog	EIF1	1.14±0.19	1±0.15	1.14
C2_15806	Protein transport protein Sec23A	SEC23A	0.76±0.12	0.75±0.06	1.01
C2_43443	Protein transport protein Sec23B	SEC23B	1.01±0.05	1.31±0.04	0.77
C2_7030	Protein transport protein Sec24A	SEC24A	3.82±1.1	4.22±0.43	0.90
C3_c55819	Protein transport protein Sec24C	SEC24C	1.48±0.23	1.31±0.14	1.13
C2_40599	Protein transport protein Sec24C	SEC24C	1.03±0.08	0.82±0.13	1.26
C2_35803	Protein transport protein Sec31A	SEC31A	1.32±0.15	2.35±0.15	0.56
C2_45211	Protein transport protein Sec31A	SEC31A	0.91±0.1	0.95±0.07	0.96
C2_324	Protein transport protein Sec61 subunit alpha-like 1	SEC61A1	1.47±0.22	0.93±0.08	1.58
C2_6339	Protein tyrosine phosphatase type IVA 2	PTP4A2	1.38±0.16	0.9±0.08	1.53
C2_7880	Protein XRP2	RP2	0.82±0.08	0.92±0.05	0.90
C2_6352	Protein Z-dependent protease inhibitor	SERPINA10	0.8±0.07	0.67±0.06	1.19
C3_c12027	Protein zer-1 homolog	ZER1	1.36±0.2	1.45±0.19	0.94
C2_36307	Protein zyg-11 homolog	ZYG11B	1.27±0.18	1.75±0.1	0.73
C2_13589	Protein-arginine deiminase type-2	PADI2	1.19±0.07	1.63±0.15	0.73
C2_39921	Protein-glutamine gamma-glutamyltransferase 2	TGM2	0.76±0.1	0.73±0.02	1.05
C2_13897	Proteoglycan 4	Prg4	1.07±0.03	1.03±0.34	1.04
C2_31468	Prothrombin	F2	1±0.05	1.7±0.58	0.59
C2_16527	Prothrombin	F2	1.17±0.09	1.55±0.43	0.76
C2_67727	Protocadherin Fat 3	FAT3	0.54±0.16	0.47±0.05	1.13
C2_13732	Protocadherin-like wing polarity protein stan	CELSR1	0.64±0.12	0.53±0.06	1.20
C2_7122	Pseudouridine-5'-monophosphatase	HDHD1	1.17±0.11	1.18±0.04	1.00
C2_16519	Pterin-4-alpha-carbinolamine dehydratase	PCBD1	0.8±0.09	1.09±0.01	0.74
C2_9779	Pterin-4-alpha-carbinolamine dehydratase 2	PCBD2	1.93±0.34	3.31±0.36	0.58
C2_21104	Puromycin-sensitive aminopeptidase	NPEPPS	0.81±0.09	0.75±0.06	1.08

C2_102229	Putative 60S ribosomal protein L37a	Rpl37a-ps1	1.71±0.29	1.15±0.05	1.49
C2_23472	Putative acyl-CoA dehydrogenase AidB	AIDB	0.94±0.05	0.58±0.07	1.62
C2_18767	Putative adenosylhomocysteinase 2	AHCYL1	1.13±0.05	1.49±0.14	0.76
C2_1163	Putative adenosylhomocysteinase 3	AHCYL2	1.25±0.14	1.56±0.43	0.81
C2_3029	Putative all-trans-retinol 13,14-reductase	RETSAT	1.53±0.19	0.57±0.02	2.70
C2_95323	Putative alpha-L-fucosidase	FUCA2	0.68±0.14	0.32±0.03	2.11
C2_962	Putative aminopeptidase W07G4.4	W07G4.4	0.72±0.13	0.46±0.04	1.56
C2_15646	Putative ATP-dependent RNA helicase an3	an3	1.31±0.13	3.22±0.47	0.41
C2_67091	Putative deoxyribonuclease TATDN3	TATDN3	0.7±0.16	0.49±0.05	1.43
C2_980	Putative deoxyribose-phosphate aldolase	DERA	0.89±0.05	1.38±0.05	0.65
C2_18015	Putative deoxyribose-phosphate aldolase	DERA	1.15±0.23	1.05±0.2	1.09
C2_62575	Putative helicase MOV-10	MOV10	0.94±0.05	0.61±0.05	1.54
C2_5188	Putative helicase mov-10-B.1	mov10b.1	1.14±0.09	0.95±0.07	1.19
C2_17207	Putative hexokinase HKDC1	HKDC1	1.24±0.08	1.69±0.08	0.73
C2_721	Putative hydroxypyruvate isomerase	HYI	0.8±0.08	0.8±0.06	1.00
C2_4760	Putative L-aspartate dehydrogenase	ASPDH	1.25±0.13	1.42±0.03	0.88
C2_37616	Putative leucine-rich repeat-containing protein DDB_G0290503	DDB_G0290503	0.73±0.1	0.64±0.08	1.13
C2_1758	Putative methyltransferase DDB_G0268948	DDB_G0290503	1.28±0.2	4.34±1.07	0.29
C2_9082	Putative N-acetylglucosamine-6-phosphate deacetylase	AMDHD2	1±0.03	1.35±0.04	0.74
C2_4695	Putative oxidoreductase GLYR1	GLYR1	1.14±0.11	1.67±0.18	0.68
C2_8640	Putative oxidoreductase yteT	yteT	1.09±0.09	1.2±0.06	0.91
C2_4527	Putative peptidyl-tRNA hydrolase PTRHD1	PTRHD1	0.66±0.12	0.58±0.05	1.12
C2_586	Putative phospholipase B-like 2	PLBD2	0.73±0.12	0.46±0.05	1.60
C2_105694	Putative protein PHLOEM PROTEIN 2-LIKE A3	IAN10	0.73±0.22	1.37±0.16	0.53
C2_2726	Putative serine protease K12H4.7	K12H4.7	0.6±0.14	0.43±0.05	1.39
C2_15791	Pyridine nucleotide-disulfide oxidoreductase domain-containing protein 2	PYROXD2	1.12±0.08	0.79±0.05	1.42
C2_8233	Pyridoxal kinase	PDXK	0.77±0.12	0.5±0.03	1.52
C2_18360	Pyridoxal phosphate phosphatase PHOSPHO2	PHOSPHO2	1.32±0.14	1.2±0.06	1.10
C2_4524	Pyridoxine-5'-phosphate oxidase	PNPO	1.32±0.14	1.22±0.06	1.08
C2_107720	Pyrin	MEFV	1.02±0.05	1.19±0.14	0.85
C2_2476	Pyroglutamyl-peptidase 1	PGPEP1	0.87±0.08	1.24±0.07	0.70
C2_9289	Pyrroline-5-carboxylate reductase 3	PYCRL	0.7±0.11	0.45±0.07	1.54
C2_2292	Pyruvate dehydrogenase E1 component subunit alpha, somatic form, mitochondrial	PDHA1	0.93±0.09	0.52±0.05	1.78
C2_2277	Pyruvate dehydrogenase E1 component subunit beta, mitochondrial	PDHB	0.97±0.09	0.41±0.03	2.37
C2_13692	Pyruvate kinase isozyme M1	PKM	1.39±0.15	2.07±0.14	0.67
C2_96589	Pyruvate kinase isozymes M1/M2	PKLR	1.17±0.07	1.46±0.07	0.80
C2_110881	Pyruvate kinase isozymes M1/M2	PKLR	1.9±0.35	2.2±0.15	0.87
C2_7049	Pyruvate kinase isozymes R/L	PKRL	2.19±0.46	3.12±0.13	0.70
C2_2874	Pyruvate kinase muscle isozyme	PKM	1.36±0.15	2±0.12	0.68
C2_1390	Quinone oxidoreductase	CRYZ	1.36±0.17	1.91±0.04	0.71
C2_32560	Rab GDP dissociation inhibitor alpha	GDI1	1.13±0.05	2.05±0.03	0.55
C2_455	Rab GDP dissociation inhibitor beta	GDI2	1.27±0.1	1.9±0.09	0.67
C2_5010	RAC-beta serine/threonine-protein kinase	AKT2	0.77±0.08	0.57±0.05	1.34
C2_81910	Ral GTPase-activating protein subunit beta	RALGAPB	0.83±0.06	0.85±0.07	0.98
C2_6063	Ran GTPase-activating protein 1	RANGAP1	0.65±0.14	0.58±0.04	1.12
C2_24742	Ran-specific GTPase-activating protein	RANBP1	1.05±0.16	1.78±0.18	0.59
C2_10857	Rap1 GTPase-GDP dissociation stimulator 1	RAP1GDS1	1.37±0.3	1.34±0.27	1.03
C2_9580	Ras GTPase-activating protein 1	RASA1	0.94±0.02	1.23±0.11	0.77
C2_35658	Ras GTPase-activating protein-binding protein 2	G3BP2	1.15±0.09	1±0.04	1.15
C2_25291	Ras GTPase-activating-like protein IQGAP1	IQGAP1	0.99±0.02	1.87±0.06	0.53
C2_54281	Ras GTPase-activating-like protein IQGAP1	IQGAP1	0.85±0.06	1.15±0.04	0.74
C2_5031	Ras GTPase-activating-like protein IQGAP1	IQGAP1	0.77±0.08	0.61±0.07	1.26
C2_93369	Ras GTPase-activating-like protein IQGAP2	IQGAP2	0.87±0.05	1.79±0.23	0.49
C2_7014	Ras GTPase-activating-like protein IQGAP2	IQGAP2	0.92±0.05	1.67±0.24	0.55
C2_1754	Ras suppressor protein 1	RSU1	1.1±0.14	2.12±0.13	0.52
C2_22704	Ras-like GTP-binding protein O-RHO	RHO_0	0.86±0.06	0.72±0.09	1.19
C2_1097	Ras-related C3 botulinum toxin substrate 1	RAC1	1.82±0.38	3.21±0.06	0.57
C2_121610	Ras-related C3 botulinum toxin substrate 2	RAC2	1.51±0.22	2.44±0.17	0.62
C2_954	Ras-related GTP-binding protein A	RRAGA	0.93±0.03	1.05±0.08	0.89
C2_6071	Ras-related protein Rab-10	RAB10	1±0.08	1.07±0.13	0.94
C2_3156	Ras-related protein Rab-11A	RAB11A	0.92±0.05	1.13±0.26	0.81
C2_2067	Ras-related protein Rab-11B	RAB11B	1.11±0.05	2.08±0.22	0.53
C2_11524	Ras-related protein Rab-13	RAB13	1.02±0.06	0.92±0.04	1.11
C2_10036	Ras-related protein Rab-14	RAB14	1.15±0.1	1.65±0.08	0.70

C2_46044	Ras-related protein Rab-18	RAB18	1.15±0.1	1.04±0.03	1.11
C2_7624	Ras-related protein Rab-1A	RAB1A	0.97±0.07	0.82±0.09	1.19
C2_67687	Ras-related protein Rab-1B	RAB1B	0.97±0.12	0.64±0.06	1.52
C2_6207	Ras-related protein Rab-20	RAB20	1.04±0.04	0.97±0.03	1.07
C2_1465	Ras-related protein Rab-2A	RAB2A	1.59±0.24	2.18±0.05	0.73
C2_4800	Ras-related protein Rab-32	RAB32	1.38±0.19	1.86±0.23	0.74
C2_6579	Ras-related protein Rab-5B	RAB5B	1.15±0.09	1.54±0.39	0.75
C2_7828	Ras-related protein Rab-5C	RAB5C	1.12±0.07	2.73±0.2	0.41
C2_30388	Ras-related protein Rab-6A	RAB6A	1.1±0.05	0.77±0.04	1.43
C2_10429	Ras-related protein Rab-7a	RAB7A	1.38±0.16	2.11±0.09	0.65
C2_4343	Ras-related protein Rab-8A	RAB8A	0.82±0.09	1.39±0.09	0.59
C2_37155	Ras-related protein Ral-A	RALA	1.02±0.12	0.58±0.07	1.77
C2_935	Ras-related protein Ral-B	RALB	0.96±0.17	1.12±0.17	0.86
C2_879	Ras-related protein Rap-1b	RAP1B	0.86±0.11	0.69±0.09	1.24
C2_7026	Ras-related protein R-Ras2	RRAS2	0.83±0.08	0.97±0.12	0.86
C2_1650	Receptor expression-enhancing protein 5	REEP5	0.93±0.11	1.03±0.04	0.90
C2_2387	Receptor-type tyrosine-protein phosphatase C	PTPRC	1.18±0.17	0.76±0.08	1.55
C2_21979	Receptor-type tyrosine-protein phosphatase F	PTPRF	0.76±0.16	0.24±0.04	3.12
C2_1647	Regucalcin	RGN	0.71±0.12	0.64±0.04	1.11
C2_2457	Regulator of microtubule dynamics protein 1	RMDN1	1.77±0.39	1.85±0.13	0.95
C2_66382	Regulator of nonsense transcripts 1	UPF1	1.09±0.12	1.01±0.07	1.08
C2_16594	Regulator of nonsense transcripts 2	UPF2	1.4±0.18	0.97±0.09	1.45
C2_12838	Renalase	RNLS	1.32±0.11	1.1±0.03	1.20
C2_8576	Replication protein A 14 kDa subunit	RPA3	0.8±0.07	0.53±0.09	1.50
C2_606	Reticulon-3	RTN3	1.26±0.16	1.67±0.11	0.75
C2_1570	Reticulon-4	RTN4	1.22±0.13	1.27±0.09	0.96
C2_25541	Retinal dehydrogenase 1	ALDH1A1	1.85±0.32	0.89±0.07	2.08
C2_933	Retinal dehydrogenase 2	ALDH1A2	0.71±0.11	0.18±0.02	3.99
C2_223	Retinoid-inducible serine carboxypeptidase	SCPEP1	1.01±0.18	0.72±0.08	1.41
C2_3697	Retinol dehydrogenase 12	RDH12	1.2±0.07	0.92±0.03	1.30
C2_9252	Retinol dehydrogenase 3	Rdh7	1.55±0.25	0.92±0.06	1.69
C2_41464	Retinol dehydrogenase 7	Rdh7	1.5±0.27	0.78±0.04	1.91
C2_5947	Retinol-binding protein 1	RBP1	1.29±0.21	1.29±0.04	1.00
C2_11901	Retinol-binding protein 2	RBP2	3.44±1.11	1.4±0.04	2.46
C2_14604	Retinol-binding protein 4-A	RBP4	1.12±0.11	0.92±0.09	1.22
C2_241	Rho GDP-dissociation inhibitor 1	ARHGDIA	1.57±0.21	1.72±0.02	0.91
C2_30696	Rho GTPase-activating protein 1	ARHGAP1	0.97±0.06	1.38±0.06	0.70
C2_31684	Rho GTPase-activating protein 10	ARHGAP10	1.15±0.07	0.98±0.21	1.17
C2_10795	Rho GTPase-activating protein 12	ARHGAP12	0.92±0.04	0.72±0.14	1.28
C2_58410	Rho GTPase-activating protein 17	ARHGAP17	1.09±0.16	2.71±0.36	0.40
C2_22447	Rho guanine nucleotide exchange factor 6	ARHGEF6	0.91±0.03	1.19±0.03	0.77
C2_30428	Rho-related GTP-binding protein RhoC	RHOC	1.22±0.13	1.64±0.15	0.75
C2_34617	Riboflavin kinase	RFK	1.63±0.28	2.41±0.48	0.68
C2_2718	Ribokinase	RBKS	0.69±0.13	0.39±0.05	1.77
C2_461	Ribonuclease H2 subunit B	RNASEH2B	0.91±0.04	1.47±0.09	0.61
C2_136	Ribonuclease T2	RNASET2	1.35±0.25	0.79±0.04	1.71
C2_8404	Ribonuclease UK114	HRSP12	1.04±0.16	0.78±0.08	1.33
C2_28116	Ribose-5-phosphate isomerase	RPIA	0.92±0.06	0.83±0.04	1.10
C2_3757	Ribose-phosphate pyrophosphokinase 2	PRPS2	1.1±0.06	1.31±0.08	0.83
C2_2840	Ribosomal protein S6 kinase 2 alpha	RPS6KA2	1.39±0.14	1.98±0.27	0.70
C2_38170	Ribosomal protein S6 kinase alpha-3	RPS6KA3	0.97±0.07	1.82±0.16	0.54
C2_1091	Ribosome maturation protein SBDS	SBDS	0.91±0.03	1.55±0.26	0.58
C3_c19359	Ribosyldihydronicotinamide dehydrogenase [quinone]	NQO2	1.09±0.06	0.5±0.03	2.18
C2_7254	RILP-like protein 1	RILPL1	0.94±0.03	1.14±0.07	0.82
C2_47038	RNA polymerase-associated protein CTR9 homolog	CTR9	0.46±0.18	0.32±0.06	1.42
C2_2005	RNA-binding protein 39	RBM39	0.63±0.13	0.65±0.08	0.96
C2_6486	RNA-binding protein 47	RBM47	1.36±0.22	3.07±0.43	0.44
C2_21199	RNA-binding protein 8A	RBM8A	1.5±0.19	2.4±0.2	0.63
C2_11034	RNA-binding protein with multiple splicing	RBPMS	0.97±0.07	2.17±0.91	0.45
C2_26961	RPE-retinal G protein-coupled receptor	RGR	1.29±0.1	1.41±0.17	0.91
C2_1576	RuvB-like 2	RUVBL2	0.9±0.05	1.3±0.08	0.69
C2_11264	S-acyl fatty acid synthase thioesterase, medium chain	OLAH	1.18±0.12	1.24±0.1	0.95
C2_1879	S-adenosylmethionine synthase isoform type-1	MAT1A	1.87±0.47	0.92±0.05	2.03
C2_972	S-adenosylmethionine synthase isoform type-2	MAT2A	1.3±0.11	1.19±0.05	1.09
C2_904	Sarcoplasmic/endoplasmic reticulum calcium ATPase 1	ATP2A1	1.23±0.13	0.82±0.1	1.50
C2_26743	Saxitoxin and tetrodotoxin-binding protein 1	PSBP1	1.14±0.15	2.89±0.84	0.39
C2_1565	Scavenger mRNA-decapping enzyme DcpS	DCPS	1±0.03	1.04±0.04	0.97

C2_13518	Scavenger receptor class B member 1	SCARB1	1.16±0.09	0.53±0.02	2.18
C2_82621	Sec1 family domain-containing protein 2	SCFD2	0.98±0.05	0.87±0.06	1.12
C2_5402	SEC23-interacting protein	SEC23IP	1.15±0.09	1.06±0.07	1.08
C2_55720	Secernin-2	SCRN2	0.99±0.03	1.31±0.07	0.75
C2_13742	Secernin-2	SCRN2	0.77±0.08	0.66±0.01	1.16
C2_1954	Sel1 repeat-containing protein 1	COA7	1.11±0.08	0.45±0.02	2.47
C2_19513	Selenide, water dikinase 1	SEPHS1	0.94±0.02	0.82±0.06	1.14
C2_6263	Selenide, water dikinase 2	SEPHS2	0.96±0.02	1.02±0.06	0.94
C2_4668	Selenium-binding protein 1	SELENBP1	1.09±0.09	0.68±0.02	1.60
C2_17882	Selenocysteine lyase	SCLY	0.68±0.11	0.49±0.02	1.38
C2_5773	Selenoprotein Pb	N327_07950	2.24±0.49	2.31±0.47	0.97
C2_150	Selenoprotein T1a	SELT	0.83±0.06	0.63±0.02	1.32
C2_16505	Septin-10	SEPT10	1.37±0.19	2.1±0.05	0.65
C2_5603	Septin-2	SEPT2	1.21±0.08	1.68±0.04	0.72
C2_22270	Septin-6	SEPT6	1.22±0.14	1.61±0.09	0.76
C2_8875	Septin-7	SEPT7	0.99±0.07	2.06±0.57	0.48
C2_9888	Septin-8-A	SEPT8	0.71±0.12	0.82±0.04	0.86
C2_5777	Septin-9	SEPT9	0.91±0.04	1.33±0.16	0.69
C2_5672	Ser/Thr-rich protein T10 in DGCR region	TANGO2	0.94±0.07	0.78±0.04	1.21
C2_10416	Serine dehydratase-like	SDSL	1.14±0.12	2.9±0.41	0.39
C2_1375	Serine hydroxymethyltransferase, cytosolic	SHMT1	0.85±0.07	1.02±0.08	0.84
C2_3144	Serine hydroxymethyltransferase, mitochondrial	SHMT2	0.88±0.07	0.37±0.01	2.40
C2_13008	Serine hydroxymethyltransferase, mitochondrial	SHMT2	0.79±0.1	0.25±0.02	3.12
C2_75706	Serine protease HTRA2, mitochondrial	HTRA2	1.14±0.09	0.64±0.15	1.79
C2_855	Serine/arginine-rich splicing factor 11	SRSF11	0.71±0.11	0.78±0.09	0.91
C2_19668	Serine/arginine-rich splicing factor 1B	SRSF1B	0.94±0.06	0.92±0.04	1.02
C2_8391	Serine/threonine-protein kinase 24	STK24	0.57±0.15	0.56±0.05	1.02
C2_11819	Serine/threonine-protein kinase MARK1	MARK1	0.6±0.16	0.29±0.01	2.07
C2_4306	Serine/threonine-protein kinase MST4	STK26	0.89±0.07	1.45±0.15	0.62
C3_c7700	Serine/threonine-protein kinase mTOR	MTOR	0.95±0.03	0.98±0.08	0.97
C2_16112	Serine/threonine-protein kinase OSR1	OXSR1	0.9±0.07	1.11±0.12	0.82
C2_3226	Serine/threonine-protein kinase PAK 2	PAK2	1.85±0.29	4.83±2.79	0.38
C2_7652	Serine/threonine-protein kinase Sgk1	SGK1	1.42±0.17	1.42±0.33	0.99
C2_19367	Serine/threonine-protein kinase TAO3	TAOK3	0.78±0.09	0.81±0.17	0.98
C2_3218	Serine/threonine-protein kinase TBK1	TBK1	1.24±0.11	1.47±0.1	0.85
C2_8036	Serine/threonine-protein phosphatase 2A 55 kDa regulatory subunit B alpha isoform	PPP2R2A	1±0.06	1.3±0.15	0.77
C2_16972	Serine/threonine-protein phosphatase 2A 56 kDa regulatory subunit alpha isoform	PPP2R5A	0.98±0.08	1.42±0.21	0.69
C2_16062	Serine/threonine-protein phosphatase 2A 56 kDa regulatory subunit epsilon isoform	PPP2R5E	1.11±0.09	2.5±0.29	0.44
C2_5266	Serine/threonine-protein phosphatase 2A 56 kDa regulatory subunit gamma isoform	Ppp2r5c	0.83±0.07	0.85±0.09	0.98
C2_7698	Serine/threonine-protein phosphatase 2A 65 kDa regulatory subunit A beta isoform	PPP2R1B	1.08±0.07	1.96±0.21	0.55
C2_4187	Serine/threonine-protein phosphatase 2A activator	PPP2R4	0.99±0.04	1.35±0.05	0.73
C2_11251	Serine/threonine-protein phosphatase 2A catalytic subunit alpha isoform	PPP2CA	0.84±0.08	1.14±0.1	0.74
C2_22338	Serine/threonine-protein phosphatase 2B catalytic subunit alpha isoform	PPP3CA	0.83±0.1	0.49±0.04	1.70
C3_c15023	Serine/threonine-protein phosphatase 2B catalytic subunit beta isoform	PPP3CB	1.46±0.21	1.74±0.12	0.84
C2_2026	Serine/threonine-protein phosphatase 4 catalytic subunit B	PPP4C	0.89±0.07	0.91±0.13	0.98
C2_7354	Serine/threonine-protein phosphatase 5	PPP5C	0.89±0.06	0.74±0.04	1.21
C2_2436	Serine/threonine-protein phosphatase 6 catalytic subunit	PPP6C	1.04±0.06	0.86±0.09	1.21
C2_29197	Serine/threonine-protein phosphatase 6 regulatory subunit 2	PPP6R2	1.21±0.1	2.35±0.07	0.51
C2_9266	Serine/threonine-protein phosphatase 6 regulatory subunit 3	PPP6R3	0.9±0.06	0.97±0.05	0.93
C2_53637	Serine/threonine-protein phosphatase PP1-alpha catalytic subunit	PPP1CA	0.78±0.07	0.45±0.05	1.73
C2_1167	Serine/threonine-protein phosphatase PP1-beta catalytic subunit	PPP1CB	0.66±0.12	0.59±0.01	1.11
C2_553	Serine/threonine-protein phosphatase PP1-gamma catalytic subunit	Ppp1cc	0.98±0.08	1.18±0.1	0.83
C2_1377	Serine--pyruvate aminotransferase, mitochondrial	AGXT	0.59±0.14	0.62±0.03	0.95
C2_866	Serine-threonine kinase receptor-associated protein	STRAP	0.86±0.05	0.83±0.05	1.04
C2_7158	Serotransferrin	TF	1.09±0.06	0.9±0.11	1.21
C2_21508	Serpin A3-5	SERPINA3	0.92±0.03	1.36±0.35	0.68
C2_112963	Serpin B6	SERPINB6	1.2±0.41	0.37±0.04	3.27

C2_110965	Serpin B8	SERPINB8	0.81±0.15	0.41±0.03	1.96
C2_14608	Serum amyloid P-component	APCS	0.81±0.09	1.22±0.49	0.66
C2_7237	Seryl-tRNA synthetase, cytoplasmic	SARS	1.28±0.11	1.72±0.44	0.74
C2_5020	Sex hormone-binding globulin	SHBG	0.99±0.06	1.35±0.19	0.74
C2_778	S-formylglutathione hydrolase	ESD	0.97±0.12	0.64±0.06	1.51
C2_122	SH3 domain-binding glutamic acid-rich-like protein	SH3BGRL	1.06±0.04	2.13±0.18	0.50
C2_107681	SH3 domain-binding glutamic acid-rich-like protein 3	SH3BGRL3	52.87±17.6	76.65±8.3	0.69
C2_4801	SHC-transforming protein 1	SHC1	0.86±0.11	1.61±0.31	0.54
C2_2197	Sialic acid synthase	NANS	1.7±0.37	3.74±0.11	0.45
C2_1438	Sialidase-1	NEU1	0.73±0.12	0.43±0.04	1.69
C2_10902	Signal peptide peptidase-like 2A	SPPL2A	0.51±0.17	0.18±0.06	2.88
C2_672	Signal recognition particle 14 kDa protein	SRP14	1.1±0.12	0.73±0.04	1.51
C2_2849	Signal recognition particle 54 kDa protein	SRP54	0.73±0.09	0.63±0.24	1.16
C2_45522	Signal recognition particle 68 kDa protein	SRP68	0.92±0.03	0.79±0.03	1.16
C2_627	Signal recognition particle receptor subunit beta	SRPRB	0.86±0.05	0.47±0.05	1.83
C2_2871	Signal transducer and activator of transcription 3	STAT3	1.11±0.08	1.68±0.04	0.66
C2_74614	Signal transducer and activator of transcription 5B	STAT5B	0.46±0.2	0.38±0.07	1.19
C2_23082	Signal-transducing adaptor protein 2	STAP2	0.7±0.11	0.5±0.02	1.38
C2_2831	Single-stranded DNA-binding protein, mitochondrial	SSBP1	1.13±0.06	1.06±0.09	1.06
C2_4819	Small nuclear ribonucleoprotein E	SNRPE	0.84±0.1	0.6±0.05	1.39
C2_27604	Small nuclear ribonucleoprotein F	SNRPF	0.88±0.11	0.54±0.03	1.63
C2_44786	Small nuclear ribonucleoprotein G	Snrpg	0.77±0.09	0.48±0.15	1.62
C2_68502	Small nuclear ribonucleoprotein Sm D1	SNRPD1	0.95±0.09	0.71±0.04	1.35
C2_46947	Small nuclear ribonucleoprotein Sm D2	SNRPD2	0.79±0.1	0.45±0.04	1.74
C2_7038	Small nuclear ribonucleoprotein Sm D3	SNRPD3	1.14±0.05	0.92±0.07	1.24
C3_c22073	Small nuclear ribonucleoprotein-associated protein B&apos	SNRPB	0.79±0.11	0.38±0.05	2.09
C2_957	Small ubiquitin-related modifier 2	SUMO2	1.12±0.22	2.92±0.2	0.38
C2_6185	S-methyl-5'-thioadenosine phosphorylase	MTAP	0.79±0.07	0.74±0.04	1.07
C2_17880	Smoothelin-like protein 1	SMTNL1	0.98±0.13	1.67±0.2	0.58
C2_837	Sodium/potassium-transporting ATPase subunit alpha-1	ATP1A1	1.6±0.29	1.98±0.12	0.81
C2_117537	Sodium/potassium-transporting ATPase subunit beta-1	ATP1B1	2.06±0.52	0.47±0.07	4.37
C2_1659	Sodium-dependent neutral amino acid transporter B(0)AT3	SLC6A18	0.76±0.13	0.48±0.17	1.59
C2_7224	Sodium-dependent phosphate transport protein 2B	SLC34A2	0.43±0.19	0.31±0.02	1.42
C2_1685	Solute carrier family 12 member 1	SLC12A1	1.14±0.08	0.96±0.03	1.19
C2_1662	Solute carrier family 13 member 2	SLC13A2	1.2±0.11	2.3±0.21	0.52
C2_5557	Solute carrier family 15 member 2	SLC15A2	0.81±0.15	1.17±0.11	0.70
C2_1290	Sorbitol dehydrogenase	SORD	0.85±0.11	0.47±0.05	1.82
C2_408	Sorcin	SRI	0.81±0.08	1.99±0.35	0.40
C2_2983	Sorting nexin-1	SNX1	0.87±0.07	1.81±0.49	0.48
C2_629	Sorting nexin-12	SNX12	0.92±0.05	1.74±0.23	0.53
C2_28449	Sorting nexin-27	SNX27	0.66±0.12	0.73±0.09	0.90
C2_1195	Sorting nexin-3	SNX3	0.86±0.08	1.02±0.27	0.84
C2_3908	Sorting nexin-4	SNX4	1±0.07	2.14±0.38	0.47
C2_1441	Sorting nexin-5	SNX5	1.22±0.14	3.46±0.63	0.35
C2_5922	Sorting nexin-7	SNX7	0.9±0.07	1.09±0.05	0.82
C2_10159	Sorting nexin-8	SNX8	0.91±0.07	2±0.32	0.46
C2_2143	Sorting nexin-9	SNX9	1.02±0.03	1.31±0.17	0.78
C2_46272	Spartin	Spg20	0.81±0.09	0.65±0.02	1.25
C2_46691	Spectrin alpha chain, brain	SPTAN1	1.09±0.14	2.26±0.1	0.48
C2_95121	Spectrin alpha chain, brain	SPTAN1	1.15±0.06	1.81±0.21	0.63
C2_46348	Spectrin alpha chain, brain	SPTAN1	1.33±0.2	1.88±0.18	0.71
C2_84720	Spectrin alpha chain, brain	SPTAN1	1.17±0.15	1.18±0.12	0.99
C2_963	Spectrin alpha chain, brain	SPTAN1	1.27±0.13	1.23±0.08	1.03
C3_c42041	Spectrin alpha chain, non-erythrocytic 1	SPTAN1	0.88±0.05	1.03±0.04	0.86
C2_46693	Spectrin beta chain, brain 1	SPTBN1	0.95±0.04	1.64±0.2	0.58
C2_31619	Spectrin beta chain, brain 1	SPTBN1	0.64±0.15	1.03±0.09	0.62
C2_8146	Spermidine synthase	SRM	1.13±0.14	0.79±0.06	1.44
C2_159	S-phase kinase-associated protein 1	SKP1	0.88±0.05	1.14±0.03	0.77
C2_34246	Sphingomyelin phosphodiesterase 2	SMPD2	1.06±0.05	0.76±0.06	1.40
C2_4387	Sphingosine-1-phosphate lyase 1	SGPL1	1.47±0.19	0.72±0.04	2.04
C2_7011	Spliceosome RNA helicase DDX39B homolog	DDX39B	1.22±0.13	1.6±0.25	0.76
C2_4049	Splicing factor 3A subunit 1	SF3A1	1.13±0.11	1.05±0.08	1.07
C2_1100	Splicing factor 3A subunit 3	SF3A3	0.95±0.08	1.13±0.13	0.85
C2_991	Splicing factor 3B subunit 1	SF3B1	1.05±0.08	1.26±0.09	0.83
C2_17969	Splicing factor 3B subunit 3	SF3B3	0.66±0.13	0.36±0.04	1.83
C3_c39473	Splicing factor 3B subunit 4	SF3B4	0.87±0.05	0.67±0.05	1.30
C2_7753	Splicing factor U2AF 65 kDa subunit	U2AF2	1.02±0.07	1.06±0.05	0.97

C2_5025	Splicing factor, proline- and glutamine-rich	SFPQ	1.06±0.04	1.2±0.04	0.89
C2_121417	Sporulation-specific protein 15	SPO15	0.63±0.13	0.47±0.02	1.36
C2_25237	Squamous cell carcinoma antigen recognized by T-cells 3	SART3	0.93±0.06	0.74±0.04	1.25
C2_9168	STAM-binding protein-like A	STAMBP	0.99±0.02	0.71±0.07	1.39
C2_1302	Staphylococcal nuclease domain-containing protein 1	SND1	1.15±0.08	1.02±0.06	1.12
C2_20724	Stonustoxin subunit alpha	SNTX-A	0.69±0.17	1.02±0.07	0.67
C2_82883	Stress-70 protein, mitochondrial	HSPA9	0.84±0.06	0.94±0.04	0.89
C2_8732	Stress-induced-phosphoprotein 1	STIP1	0.94±0.11	1.37±0.11	0.68
C2_23619	Striatin	STRN	0.93±0.05	1.22±0.07	0.76
C2_56395	Striatin-3	STRN3	1.23±0.09	1.33±0.03	0.92
C2_32020	Stromal membrane-associated protein 1	SMAP1	1±0.2	1.02±0.11	0.98
C2_4823	Structural maintenance of chromosomes protein 3	SMC3	0.73±0.09	0.66±0.03	1.12
C2_1571	Succinate dehydrogenase [ubiquinone] flavoprotein subunit, mitochondrial	SDHA	1.44±0.29	0.7±0.03	2.07
C2_255	Succinyl-CoA ligase [ADP/GDP-forming] subunit alpha, mitochondrial	SUCLG1	1.05±0.05	0.92±0.04	1.14
C2_11441	Succinyl-CoA ligase [GDP-forming] subunit beta, mitochondrial	SUCLG2	1.37±0.2	1.33±0.17	1.03
C2_10991	Sucrase-isomaltase, intestinal	SI	0.76±0.16	0.26±0.03	2.98
s_rl0002d06_f_1	Sulfhydryl oxidase 1	QSOX1	0.61±0.14	0.47±0.04	1.29
C2_5930	Sulfide:quinone oxidoreductase, mitochondrial	SQRDL	1.33±0.16	0.9±0.05	1.49
C3_c52929	Sulfite oxidase	SUOX	0.9±0.13	1.3±0.17	0.70
C2_4747	Sulfite oxidase, mitochondrial	SUOX	0.88±0.08	1.53±0.14	0.58
C2_1085	Sulfotransferase 1C1	SULT1C3	0.89±0.07	2.51±0.56	0.35
C2_3986	Sulfotransferase 1C2	SULT1C2	0.84±0.07	3.78±2.04	0.22
C2_7228	Sulfotransferase 6B1	SULT6B1	1.32±0.12	2.35±0.16	0.56
C2_8495	Sulfotransferase family cytosolic 2B member 1	SULT2B1	1.63±0.23	1.65±0.28	0.99
C2_6487	SUMO-activating enzyme subunit 1	SAE1	1±0.02	1.41±0.16	0.71
C2_6124	SUMO-activating enzyme subunit 2	UBA2	0.87±0.07	1.49±0.42	0.58
C2_11156	SUMO-conjugating enzyme UBC9	UBE2I	1.5±0.18	2.05±0.26	0.73
C2_368	Superoxide dismutase [Cu-Zn]	SOD1	0.75±0.14	0.3±0.03	2.47
C2_1642	Superoxide dismutase [Mn], mitochondrial	SOD2	0.74±0.12	0.29±0.02	2.54
C2_1686	Suppressor of G2 allele of SKP1 homolog	SUGT1	1.24±0.1	1.56±0.04	0.79
C2_4089	Sushi domain-containing protein 2	SUSD2	0.7±0.11	0.59±0.06	1.17
C2_75529	SWI/SNF complex subunit SMARCC1	SMARCC1	0.9±0.11	1.12±0.18	0.80
C2_5248	SWI/SNF-related matrix-associated actin-dependent regulator of chromatin subfamily D member 2	SMARCD2	0.96±0.06	1.15±0.2	0.84
C2_31339	Synaptic vesicle membrane protein VAT-1 homolog	VAT1	1.06±0.14	1.03±0.09	1.03
C2_2785	Synaptobrevin homolog YKT6	YKT6	1.4±0.24	1.55±0.04	0.90
C2_3925	Synaptophysin-like protein 1	SYPL1	0.93±0.12	0.3±0.03	3.05
C2_3072	Synaptophysin-like protein 2	SYPL2	1.72±0.3	1.07±0.36	1.60
C2_3553	Syntaxin-7	STX7	0.8±0.07	1.44±0.55	0.56
C2_3302	Syntaxin-binding protein 2	STXBP2	0.82±0.09	0.89±0.09	0.93
C2_1047	Syntenin-1	SDCBP	1.14±0.09	1.71±0.25	0.66
C2_15378	Talin-1	TLN1	1.23±0.11	2.9±0.09	0.42
C2_40101	Talin-1	TLN1	1.23±0.13	1.95±0.14	0.63
C2_76944	Talin-1	TLN1	1.01±0.06	1.11±0.05	0.91
C2_5352	Talin-1	TLN1	0.94±0.03	0.89±0.03	1.05
C2_83924	Talin-1	TLN1	1.13±0.06	1.01±0.08	1.12
C2_41984	Talin-2	TLN2	0.94±0.05	1.27±0.02	0.74
C2_4568	TAR DNA-binding protein 43	TARDBP	1.23±0.21	1.97±0.25	0.62
C2_8321	Target of Myb protein 1	TOM1	1.02±0.1	1.98±0.2	0.52
C2_13458	Tax1-binding protein 3	TAX1BP3	0.88±0.06	0.69±0.11	1.28
C2_8193	TBC1 domain family member 13	TBC1D13	1.16±0.13	1.69±0.13	0.69
C2_4562	TBC1 domain family member 17	TBC1D17	1.22±0.11	2.02±0.05	0.60
C2_48672	TBC1 domain family member 24	TBC1D24	0.75±0.1	1.37±0.04	0.55
C2_342	T-complex protein 1 subunit alpha	TCP1	0.97±0.04	1.19±0.07	0.82
C2_2982	T-complex protein 1 subunit beta	CCT2	1.04±0.14	1.72±0.28	0.61
C2_4858	T-complex protein 1 subunit delta	CCT4	1.17±0.09	1.55±0.05	0.76
C2_298	T-complex protein 1 subunit epsilon	CCT5	1.15±0.09	1.6±0.23	0.72
C2_1691	T-complex protein 1 subunit eta	CCT7	1±0.05	1.19±0.14	0.84
C2_1250	T-complex protein 1 subunit gamma	CCT3	1.09±0.07	1.49±0.14	0.73
C2_397	T-complex protein 1 subunit theta	CCT8	1.38±0.16	2.04±0.22	0.68
C2_3533	T-complex protein 1 subunit zeta	CCT6A	1.05±0.05	1.6±0.21	0.66
C2_77	Tetraspanin-1	TSPAN1	0.68±0.13	0.56±0.06	1.23
C2_652	Tetratricopeptide repeat protein 35	EMC2	0.81±0.07	0.67±0.04	1.21
C2_2347	Tetratricopeptide repeat protein 38	TTC38	1.12±0.07	2.46±0.12	0.45
C2_7513	THAP domain-containing protein 4	THAP4	0.69±0.11	0.74±0.1	0.93

C2_7496	Thiamin pyrophosphokinase 1	TPK1	1±0.09	0.8±0.05	1.24
C2_15542	Thimet oligopeptidase	THOP1	0.78±0.08	0.49±0.05	1.60
C2_71169	Thimet oligopeptidase	THOP1	0.82±0.09	0.46±0.07	1.79
C2_1158	Thioredoxin	TXN	3.24±0.99	8.79±0.88	0.37
C2_4240	Thioredoxin domain-containing protein 12	TXNDC12	1.19±0.09	1.16±0.04	1.02
C2_2180	Thioredoxin domain-containing protein 17	TXNDC17	1.75±0.27	2.93±0.15	0.60
C2_5341	Thioredoxin domain-containing protein 5	TXNDC5	2.13±0.47	2.04±0.19	1.05
C2_334	Thioredoxin domain-containing protein 9	TXNDC9	0.88±0.12	1.14±0.23	0.77
C2_1093	Thioredoxin reductase 3	TXNRD3	0.77±0.09	0.47±0.03	1.62
C2_1183	Thioredoxin-like protein 1	TXNL1	0.81±0.08	0.48±0.04	1.71
C3_c44742	Thioredoxin-related transmembrane protein 1	TMX1	1.45±0.27	0.94±0.03	1.54
C2_13380	Thiosulfate sulfurtransferase/rhodanese-like domain-containing protein 1	TSTD1	2.86±1.24	5.27±0.46	0.54
C2_6564	THO complex subunit 4	ALYREF	1.07±0.06	1.21±0.04	0.88
C2_7672	Threonine synthase-like 2	THNSL2	0.83±0.07	0.55±0.04	1.51
C2_3045	Threonyl-tRNA synthetase, cytoplasmic	TARS	1.56±0.19	1.47±0.07	1.06
C2_11844	THUMP domain-containing protein 1	THUMPD1	1.14±0.19	1.07±0.15	1.06
C2_34441	Thymidine phosphorylase	TYMP	0.92±0.06	1.59±0.26	0.58
C2_21128	Thymidylate kinase	DTYMK	1.02±0.18	0.85±0.11	1.21
C2_4641	Thymocyte nuclear protein 1	THYN1	1.32±0.19	2.34±0.15	0.57
C2_20231	Thyroid hormone-induced protein B	NCOA3	0.78±0.11	0.48±0.04	1.64
C2_14436	Tight junction protein ZO-2	TJP2	0.95±0.04	1.18±0.03	0.81
C2_1981	Tissue alpha-L-fucosidase	FUCA1	0.85±0.14	0.48±0.03	1.75
C2_8546	Tissue factor	F3	0.74±0.12	0.34±0.03	2.17
C2_6212	Tissue factor pathway inhibitor	TFPI	0.61±0.13	0.88±0.13	0.69
C2_1974	Toll-interacting protein	TOLLIP	1.5±0.17	1.89±0.04	0.80
C2_63548	TOM1-like protein 2	TOM1L2	1.87±0.44	3.3±0.44	0.57
C2_4702	TP53RK-binding protein	TPRKB	1.1±0.04	2.17±0.37	0.51
C2_10331	Trafficking protein particle complex subunit 1	TRAPPC1	0.97±0.01	1.08±0.03	0.90
C2_38920	Trafficking protein particle complex subunit 11	TRAPPC11	1±0.22	1.96±0.26	0.51
C2_7033	Trafficking protein particle complex subunit 11	TRAPPC11	0.75±0.09	0.71±0.06	1.05
C2_3017	Trafficking protein particle complex subunit 2	TRAPPC2	0.98±0.09	0.85±0.06	1.15
C2_23806	Trafficking protein particle complex subunit 3	TRAPPC3	0.96±0.19	1.68±0.24	0.57
C2_554	Trafficking protein particle complex subunit 4	TRAPPC4	1.44±0.16	1.57±0.05	0.91
C2_16861	Trafficking protein particle complex subunit 5	TRAPPC5	1.07±0.09	1.02±0.21	1.06
C2_15018	Trafficking protein particle complex subunit 9	TRAPPC9	0.84±0.06	0.74±0.14	1.13
C2_18467	Trans-1,2-dihydrobenzene-1,2-diol dehydrogenase	DHDH	1.19±0.07	1.14±0.02	1.05
C2_564	Transaldolase	TALDO1	1.59±0.22	1.9±0.05	0.84
C2_18811	Transcobalamin-2	TCN2	0.64±0.12	0.66±0.1	0.98
C2_529	Transcription elongation factor B polypeptide 1	TCEB1	1.37±0.18	1.86±0.13	0.74
C2_942	Transcription elongation factor B polypeptide 2	Tceb2	1.13±0.1	1.4±0.07	0.81
C2_5103	Transcription elongation factor SPT5	SUPT5H	0.91±0.05	0.88±0.05	1.04
C2_87436	Transcription elongation factor SPT6	SUPT6H	1.13±0.22	0.94±0.07	1.20
C2_14940	Transcription factor BTF3 homolog 4	BTF3L4	1.07±0.08	1.24±0.22	0.86
C2_4032	Transcription factor p65	RELA	0.87±0.07	0.8±0.04	1.09
C2_6927	Transcription initiation factor IIA subunit 2	GTF2A2	0.61±0.19	0.33±0.04	1.84
C2_1687	Transcriptional activator protein Pur-beta-B	PURB	0.68±0.11	0.82±0.03	0.83
C2_1468	Transducin beta-like protein 2	TBL2	0.83±0.07	0.61±0.05	1.36
C2_3567	Transferrin receptor protein 1	TFRC	0.91±0.07	0.64±0.08	1.42
C2_4791	Transgelin	TAGLN	1.12±0.16	2.28±0.58	0.49
C2_15562	Transitional endoplasmic reticulum ATPase	VCP	1.89±0.34	3.05±0.31	0.62
C3_c25177	Transitional endoplasmic reticulum ATPase	VCP	0.7±0.12	0.56±0.05	1.26
C3_c6442	Transketolase	TKT	0.84±0.14	0.57±0.06	1.47
C2_824	Transketolase-like protein 2	TKTL2	0.77±0.08	0.95±0.08	0.81
C2_44506	Translational activator GCN1	GCN1L1	0.99±0.09	1.22±0.23	0.82
C2_58570	Translational activator GCN1	GCN1L1	0.77±0.09	0.93±0.17	0.83
C2_16615	Translational activator GCN1	GCN1L1	0.75±0.11	0.74±0.13	1.01
C2_8982	Translational activator GCN1	GCN1L1	0.81±0.08	0.56±0.03	1.44
C2_15	Translationally-controlled tumor protein homolog	TPT1	0.82±0.07	0.8±0.06	1.03
C2_23686	Translin	TSN	0.81±0.11	0.47±0.07	1.71
C2_28067	Translin-associated protein X	TSNAX	0.74±0.14	0.42±0.05	1.76
C2_258	Translocon-associated protein subunit beta	SSR2	1.54±0.22	0.82±0.07	1.88
C2_49400	Transmembrane 6 superfamily member 2	TM6SF2	1.25±0.2	0.7±0.05	1.79
C2_3796	Transmembrane 9 superfamily member 3	TM9SF3	1.53±0.29	0.85±0.05	1.80
C2_671	Transmembrane and immunoglobulin domain-containing protein 1	TMIGD1	0.88±0.11	1.31±0.13	0.67
C2_39395	Transmembrane channel-like protein 8	TMC8	1.13±0.13	0.76±0.13	1.48

C2_12865	Transmembrane emp24 domain-containing protein 2	TMED2	0.96±0.02	0.53±0.06	1.81
C2_2279	Transmembrane emp24 domain-containing protein 7	TMED7	1.36±0.21	1.21±0.1	1.13
C2_48280	Transmembrane protease serine 4	TMPRSS4	0.93±0.06	0.61±0.05	1.51
C2_2859	Transmembrane protein 135	TMEM135	1.17±0.11	1.74±0.23	0.67
C2_4100	Transmembrane protein 14A	TMEM14A	0.72±0.13	0.3±0.02	2.39
C2_25549	Transmembrane protein C10orf57 homolog	CJ057	1.04±0.16	0.6±0.19	1.72
C2_43636	Transportin-2	TNPO2	0.93±0.07	1.91±0.32	0.49
C2_22440	Transportin-3	TNPO3	0.95±0.08	1.32±0.18	0.72
C2_75383	Transthyretin	TTR	1.03±0.08	0.68±0.07	1.52
C2_5754	Trehalase	TREH	0.5±0.17	0.31±0.04	1.60
C2_10341	Tricarboxylate transport protein, mitochondrial	Slc25a1	1.25±0.24	0.41±0.07	3.05
C2_3043	Trifunctional enzyme subunit alpha, mitochondrial	HADHA	1.43±0.3	0.8±0.04	1.78
C2_2435	Trifunctional enzyme subunit beta, mitochondrial	HADHB	1.28±0.21	1.15±0.06	1.11
C2_14515	Trimethyllysine dioxygenase, mitochondrial	TMLHE	0.89±0.09	1.28±0.11	0.70
C2_52	Triosephosphate isomerase B	TPI1	0.98±0.09	0.5±0.05	1.95
C2_114171	Tripartite motif-containing protein 14	TRIM14	0.98±0.1	0.67±0.05	1.47
C2_7955	Tripartite motif-containing protein 16	TRIM16	0.77±0.08	0.78±0.05	0.99
C2_10685	Tripartite motif-containing protein 29	TRIM29	1.32±0.14	2.01±0.07	0.65
C2_1412	Tripeptidyl-peptidase 1	TPP1	0.9±0.07	0.56±0.03	1.60
C2_27720	Tripeptidyl-peptidase 2	TPP2	0.98±0.08	0.99±0.07	0.99
C2_26986	tRNA (adenine-N(1)-)-methyltransferase non-catalytic subunit TRM6	TRMT6	1.31±0.12	1.13±0.12	1.15
C2_15864	tRNA-splicing ligase RtcB homolog	RTCB	0.9±0.03	1.21±0.04	0.75
C2_44653	Tropomyosin alpha-4 chain	Tpm4	4.79±2.01	23.02±8.65	0.21
C2_437	Tropomyosin beta chain	Tpm2	1.26±0.21	2.31±0.47	0.55
C2_1213	Trypsin	2210010C04Rik	0.56±0.17	0.31±0.08	1.85
C2_84054	Trypsin-1	PRSS1	0.47±0.19	0.18±0.06	2.58
C2_121419	Trypsin-2	PRSS2	0.57±0.17	0.29±0.08	1.98
C2_4459	Trypsin-3	PRSS3	0.55±0.17	0.25±0.05	2.20
C3_c41957	Trypsinogen-like protein 3	PRSS3	0.46±0.18	0.31±0.04	1.49
C2_1950	Tryptase	Tpsab1	0.67±0.11	0.48±0.04	1.41
C2_1646	Tryptophanyl-tRNA synthetase, cytoplasmic	WARS	0.79±0.08	0.85±0.03	0.93
C2_101373	Tubulin alpha-1 chain	TUBA1A	0.95±0.06	1.03±0.06	0.93
C2_38179	Tubulin alpha-1A chain	TUBA1A	1.55±0.33	1.86±0.28	0.83
C2_7711	Tubulin alpha-1B chain	TUBA1B	1.42±0.23	2.23±0.46	0.64
C2_100383	Tubulin alpha-2 chain	TUBA2	1.13±0.05	1.22±0.07	0.93
C2_20113	Tubulin alpha-4A chain	TUBA4A	1.48±0.3	2.96±1.21	0.50
C2_905	Tubulin beta chain	TUBB	0.82±0.08	1.29±0.32	0.64
C2_90	Tubulin beta-1 chain	TUBB1	1.4±0.25	3.07±1.7	0.46
C2_8011	Tubulin beta-7 chain	TUBB7	0.88±0.04	1.05±0.17	0.84
C2_2978	Tubulin-folding cofactor B	TBCB	1.28±0.21	2.22±0.06	0.58
C2_1292	Tubulin-specific chaperone A	TBCA	0.73±0.1	0.99±0.24	0.73
C2_2326	Tumor necrosis factor receptor type 1-associated DEATH domain protein	TRADD	0.81±0.09	1.11±0.22	0.73
C2_6149	Tumor protein D52	TPD52	0.86±0.18	0.65±0.13	1.33
C2_20971	Tumor protein D54	TPD52L2	0.6±0.14	0.33±0.02	1.84
C2_6726	Tumor susceptibility gene 101 protein	TSG101	1.27±0.11	1.33±0.07	0.96
C2_877	Tumor-associated calcium signal transducer 2	TACSTD2	1.13±0.13	0.94±0.03	1.21
C2_6143	Twinfilin-1	TWF1	1.22±0.14	2.81±0.45	0.43
C2_3281	Type II inositol-1,4,5-trisphosphate 5-phosphatase	INPP5B	1±0.02	0.9±0.03	1.11
C2_12783	Type-4 ice-structuring protein LS-12	AFP4	1.02±0.12	2.41±1.81	0.42
C2_2163	Tyrosine-protein kinase CSK	CSK	0.97±0.03	0.94±0.07	1.02
C2_90526	Tyrosine-protein kinase Yes	YES1	1.26±0.22	1.51±0.11	0.84
C2_16846	Tyrosine-protein phosphatase non-receptor type 11	PTPN11	1.34±0.2	1.67±0.14	0.80
C2_731	Tyrosine-protein phosphatase non-receptor type 6	PTPN6	1.49±0.29	1.25±0.06	1.19
C2_28595	U1 small nuclear ribonucleoprotein 70 kDa	SNRNP70	0.65±0.13	0.76±0.17	0.86
C2_6732	U1 small nuclear ribonucleoprotein A	Snrpa	0.78±0.07	0.64±0.06	1.22
C2_20155	U5 small nuclear ribonucleoprotein 200 kDa helicase	SNRNP200	1.02±0.06	1±0.07	1.02
C2_31571	U5 small nuclear ribonucleoprotein 200 kDa helicase	SNRNP200	2.39±0.6	2.13±0.3	1.12
C2_5077	U5 small nuclear ribonucleoprotein 200 kDa helicase	SNRNP200	1.02±0.19	0.65±0.18	1.57
C2_3229	U6 snRNA-associated Sm-like protein LSm1	LSM1	0.99±0.03	0.99±0.02	1.00
C2_28803	U6 snRNA-associated Sm-like protein LSm2	LSM2	0.78±0.1	0.52±0.05	1.50
C2_26124	U6 snRNA-associated Sm-like protein LSm3	LSM3	0.98±0.01	0.92±0.04	1.06
C2_4328	U6 snRNA-associated Sm-like protein LSm4	LSM4	0.89±0.05	0.72±0.09	1.23
C2_55073	U6 snRNA-associated Sm-like protein LSm5	LSM5	0.98±0.08	0.82±0.04	1.20
C2_53085	U6 snRNA-associated Sm-like protein LSm6	LSM6	0.81±0.07	0.66±0.02	1.23
C2_3362	U6 snRNA-associated Sm-like protein LSm7	LSM7	0.72±0.1	0.63±0.04	1.13

C2_19544	Ubiquilin-1	UBQLN1	0.93±0.06	1.01±0.06	0.92
C2_2288	Ubiquilin-2	UBQLN2	1.25±0.17	1.41±0.04	0.88
C2_3405	Ubiquinone biosynthesis protein COQ9, mitochondrial	COQ9	1.35±0.16	0.64±0.04	2.09
C2_6289	Ubiquitin carboxyl-terminal hydrolase 10	USP10	0.51±0.16	0.48±0.1	1.07
C2_1964	Ubiquitin carboxyl-terminal hydrolase 14	USP14	1.26±0.13	2.21±0.25	0.57
C2_107070	Ubiquitin carboxyl-terminal hydrolase 21	USP21	1.61±0.29	10.1±5.41	0.16
C2_44713	Ubiquitin carboxyl-terminal hydrolase 4	USP4	1.2±0.09	1.72±0.06	0.70
C2_27032	Ubiquitin carboxyl-terminal hydrolase 47	USP47	1.16±0.15	1.37±0.04	0.85
C2_20830	Ubiquitin carboxyl-terminal hydrolase 5	USP5	1.28±0.14	1.97±0.33	0.65
C2_48312	Ubiquitin carboxyl-terminal hydrolase 7	USP7	0.95±0.19	2.53±0.77	0.38
C2_18831	Ubiquitin carboxyl-terminal hydrolase 7	USP7	0.88±0.09	0.62±0.07	1.42
C2_660	Ubiquitin carboxyl-terminal hydrolase isozyme L3	UCHL3	1.29±0.15	2.13±0.02	0.61
C2_1022	Ubiquitin carboxyl-terminal hydrolase isozyme L5	UCHL5	1.22±0.12	2.3±0.15	0.53
C2_13539	Ubiquitin conjugation factor E4 A	UBE4A	1.08±0.07	0.81±0.1	1.33
C2_7265	Ubiquitin conjugation factor E4 B	UBE4B	0.74±0.11	1.15±0.16	0.65
C2_470	Ubiquitin fusion degradation protein 1 homolog	UFD1L	0.97±0.06	0.59±0.06	1.64
C2_155	Ubiquitin thioesterase OTUB1	Otub1	1.11±0.07	1.96±0.17	0.56
C2_648	Ubiquitin-60S ribosomal protein L40	Uba52	0.91±0.18	0.49±0.07	1.84
C2_10454	Ubiquitin-associated protein 1	UBAP1	0.67±0.11	0.61±0.05	1.10
C2_5227	Ubiquitin-conjugating enzyme E2 D2	UBE2D2	1.23±0.13	1.56±0.1	0.79
C2_4604	Ubiquitin-conjugating enzyme E2 K	UBE2K	0.9±0.05	1.27±0.05	0.71
C2_6947	Ubiquitin-conjugating enzyme E2 L3	UBE2L3	1.52±0.35	2.56±0.19	0.59
C2_17030	Ubiquitin-conjugating enzyme E2 N	UBE2N	1.07±0.08	1.95±0.41	0.55
C2_1249	Ubiquitin-conjugating enzyme E2 variant 1	UBE2V1	0.87±0.11	0.78±0.09	1.12
C2_1363	Ubiquitin-conjugating enzyme E2 variant 2	UBE2V2	1.19±0.18	2.52±0.38	0.47
C2_112839	Ubiquitin-conjugating enzyme E2-18 kDa	UBE2G2	0.84±0.09	1.05±0.19	0.80
C2_9630	Ubiquitin-fold modifier 1	UFM1	0.87±0.05	1.29±0.19	0.67
C2_8231	Ubiquitin-like modifier-activating enzyme 1	UBA1	1.21±0.12	1.96±0.13	0.62
C2_39433	Ubiquitin-like modifier-activating enzyme 6	UBA6	0.7±0.1	0.76±0.07	0.92
C2_6707	Ubiquitin-like modifier-activating enzyme ATG7	ATG7	0.87±0.08	1.58±0.25	0.55
C2_4528	Ubiquitin-like protein 3	UBL3	1.03±0.13	0.67±0.06	1.54
C2_29802	Ubiquitin-like protein FUBI	FAU	2.2±0.57	2.19±0.26	1.00
C2_5019	Ubiquitin-like-conjugating enzyme ATG3	ATG3	0.97±0.04	1.49±0.22	0.65
C2_13734	UBX domain-containing protein 1	UBXN1	1.67±0.43	2.9±0.31	0.58
C2_7714	UDP-glucose 4-epimerase	GALE	1.04±0.03	1.16±0.05	0.89
C2_11633	UDP-glucose 6-dehydrogenase	UGDH	3.23±0.85	1.5±0.16	2.15
C2_57655	UDP-glucose:glycoprotein glucosyltransferase 1	UGGT1	5.35±1.58	2.86±0.36	1.87
C2_3599	UDP-glucuronosyltransferase	UGT1A1	1.36±0.21	0.26±0.01	5.27
C2_15625	UDP-glucuronosyltransferase 1-6	UGT1A6	1.12±0.09	0.64±0.04	1.74
C2_12213	UDP-glucuronosyltransferase 1-9	UGT1A9	1.25±0.13	0.75±0.09	1.66
C2_7856	UDP-glucuronosyltransferase 2A1	UGT2A1	1.25±0.16	0.48±0.02	2.59
C2_5354	UDP-glucuronosyltransferase 2A2	UGT2A2	1.11±0.12	0.39±0.02	2.86
C2_24614	UDP-glucuronosyltransferase 2A3	UGT2A3	0.63±0.13	0.45±0.05	1.42
C2_54573	UDP-glucuronosyltransferase 2B15	UGT2B7	1.16±0.12	0.35±0.02	3.33
C2_16491	UDP-glucuronosyltransferase 2B17	UGT2B17	1.27±0.09	0.87±0.09	1.46
C2_111727	UDP-glucuronosyltransferase 2B17	UGT2B17	1.13±0.13	0.41±0.01	2.73
C2_4592	UDP-N-acetylglucosamine--dolichyl-phosphate N-acetylglucosaminephosphotransferase	DPAGT1	1.3±0.29	0.56±0.12	2.31
C2_12651	UDP-N-acetylhexosamine pyrophosphorylase	UAP1	2.02±0.42	2.31±0.39	0.88
C2_7433	UDP-N-acetylhexosamine pyrophosphorylase-like protein 1	UAP1L1	0.81±0.07	1.59±0.19	0.51
C2_9914	Ufm1-specific protease 2	UFSP2	0.84±0.06	0.86±0.04	0.97
C2_6090	UMP-CMP kinase	CMPK1	1.04±0.09	2.61±1.46	0.40
C2_5879	Uncharacterized methyltransferase ydaC	ydaC	0.84±0.09	0.59±0.04	1.42
C2_3672	Uncharacterized oxidoreductase C663.06c	SPCC663.06c	1.39±0.16	0.74±0.03	1.87
C2_10013	Uncharacterized oxidoreductase YtbE	ytbE	1.03±0.03	1.34±0.12	0.76
C2_12162	Uncharacterized protein C18H10.09	SPBC18H10.09	0.78±0.08	0.72±0.04	1.09
C2_79778	Uncharacterized protein C6orf106	C6orf106	0.69±0.12	0.46±0.08	1.51
C2_23974	Uncharacterized protein KIAA2013 homolog	KIAA2013	0.99±0.12	0.49±0.02	2.03
C2_12864	Uncharacterized protein LOC388588 homolog	LOC388588	1.29±0.25	0.48±0.06	2.72
C2_11954	Uncharacterized protein MJ0240	BN478_00635	1.84±0.45	2.28±0.12	0.80
C2_35646	Uncharacterized protein R102.4	Tha1	0.53±0.16	0.47±0.08	1.13
C3_c35178	Unconventional myosin-VIIa	MYO7A	1.59±0.33	1.5±0.11	1.06
C2_9334	UPF0160 protein MYG1, mitochondrial	C12orf10	0.81±0.1	0.43±0.03	1.88
C2_6389	UPF0308 protein C9orf21	C9orf21	1.13±0.15	0.98±0.21	1.15
C2_5361	UPF0364 protein C6orf211 homolog	C6orf211	1.14±0.11	1.63±0.21	0.70
C2_5574	UPF0366 protein C11orf67 homolog	C11orf67	0.67±0.11	0.86±0.03	0.78
C2_53467	UPF0405 protein C3orf75 homolog	ELP6	1.17±0.23	2.53±0.26	0.46

C2_5877	UPF0462 protein C4orf33 homolog	C4orf33	0.49±0.17	0.33±0.06	1.49
C2_6009	UPF0466 protein C22orf32 homolog, mitochondrial	SMDT1	1.41±0.16	0.93±0.14	1.51
C2_2787	UPF0468 protein C16orf80 homolog	C16orf80	1.12±0.12	0.55±0.08	2.03
C2_41651	UPF0505 protein C16orf62 homolog	C16orf62	0.94±0.04	1.86±0.45	0.50
C2_83574	UPF0505 protein C16orf62 homolog	C16orf62	0.93±0.06	1.61±0.09	0.58
C2_9851	UPF0533 protein C5orf44 homolog	C5orf44	52.87±17.6	76.65±8.3	0.69
C2_19805	UPF0552 protein C15orf38 homolog	C15orf38	1.44±0.22	2.42±0.16	0.60
C2_31615	UPF0553 protein C9orf64 homolog	C9orf64	1.09±0.03	0.79±0.06	1.37
C2_10677	UPF0554 protein C2orf43 homolog	C2orf43	1.09±0.09	0.77±0.04	1.41
C2_2220	UPF0556 protein C19orf10 homolog	C19orf10	0.89±0.2	0.24±0.04	3.72
C2_2379	UPF0568 protein C14orf166 homolog	C14orf166	0.97±0.07	1.19±0.26	0.81
C2_5897	UPF0587 protein C1orf123	C1orf123	0.79±0.08	0.69±0.03	1.14
C2_22308	UPF0668 protein C10orf76 homolog	C10orf76	0.8±0.09	0.71±0.1	1.12
C2_2273	UPF0765 protein C10orf58 homolog	C10orf58	1.09±0.1	0.38±0.04	2.85
C2_15956	Up-regulator of cell proliferation	URGCP	0.98±0.05	0.76±0.07	1.29
C2_1340	Uridine phosphorylase 1	UPP1	1.01±0.03	1.32±0.13	0.77
C2_491	Uroporphyrinogen decarboxylase	UROD	1.01±0.03	1.22±0.05	0.82
C2_355	Uroporphyrinogen-III synthase	UROS	0.98±0.04	1.02±0.18	0.96
C2_4863	UTP--glucose-1-phosphate uridylyltransferase	UGP2	1.55±0.28	2.94±0.17	0.53
C2_20296	UV excision repair protein RAD23 homolog A	RAD23A	1.63±0.26	3.06±0.01	0.53
C2_15352	UV excision repair protein RAD23 homolog B	RAD23B	0.64±0.14	0.89±0.28	0.72
C2_4908	Vacuolar protein sorting-associated protein 11 homolog	VPS11	0.81±0.09	0.86±0.08	0.93
C2_14960	Vacuolar protein sorting-associated protein 13A	VPS13A	1.08±0.04	0.85±0.05	1.27
C2_12800	Vacuolar protein sorting-associated protein 13C	VPS13C	1.05±0.06	1.05±0.11	1.00
C2_81516	Vacuolar protein sorting-associated protein 13D	VPS13D	1.66±0.32	2.13±0.34	0.78
C2_3947	Vacuolar protein sorting-associated protein 16 homolog	VPS16	0.89±0.11	0.62±0.03	1.44
C2_4890	Vacuolar protein sorting-associated protein 18 homolog	VPS18	0.84±0.08	0.83±0.04	1.01
C2_13056	Vacuolar protein sorting-associated protein 26B-B	VPS26B	1.38±0.14	1.82±0.04	0.76
C2_8488	Vacuolar protein sorting-associated protein 26B-like	VPS26BL	1.18±0.07	1.66±0.12	0.71
C2_1930	Vacuolar protein sorting-associated protein 28 homolog	VPS28	1.02±0.04	1.38±0.19	0.74
C2_6316	Vacuolar protein sorting-associated protein 29	VPS29	0.96±0.05	0.81±0.06	1.18
C2_2873	Vacuolar protein sorting-associated protein 33A	VPS33A	0.71±0.1	0.85±0.09	0.83
C2_3610	Vacuolar protein sorting-associated protein 35	VPS35	0.95±0.02	1.71±0.17	0.56
C2_23478	Vacuolar protein sorting-associated protein 35	VPS35	1.03±0.05	1.56±0.18	0.66
C2_12026	Vacuolar protein sorting-associated protein 37A	VPS37A	0.83±0.08	0.71±0.06	1.17
C2_3186	Vacuolar protein sorting-associated protein 41 homolog	VPS41	0.53±0.16	0.38±0.07	1.41
C2_7439	Vacuolar protein sorting-associated protein 45	VPS45	1.18±0.16	0.97±0.21	1.21
C2_2867	Vacuolar protein sorting-associated protein 4B	VPS4B	0.98±0.02	1.93±0.17	0.51
C2_19718	Vacuolar protein sorting-associated protein 53 homolog	VPS53	1.01±0.07	1.76±0.31	0.57
C2_36362	Vacuolar protein sorting-associated protein 8 homolog	VPS8	0.84±0.08	0.97±0.18	0.86
C2_9965	Vacuolar protein sorting-associated protein VTA1 homolog	VTA1	0.87±0.05	1.08±0.06	0.81
C2_1783	Vacuolar protein-sorting-associated protein 25	VPS25	1.12±0.07	1.54±0.04	0.73
C2_4706	Vacuolar protein-sorting-associated protein 36	VPS36	1.16±0.06	1.56±0.1	0.74
C2_6246	Vacuolar-sorting protein SNF8	SNF8	0.76±0.12	0.65±0.12	1.17
C2_5432	Valyl-tRNA synthetase	VARS2	1.04±0.03	0.85±0.07	1.22
C2_57229	Vasodilator-stimulated phosphoprotein	VASP	1.88±0.38	3.06±0.18	0.61
C2_6700	Very long-chain acyl-CoA synthetase	SLC27A2	1.2±0.08	1.15±0.07	1.04
C2_47981	Very long-chain specific acyl-CoA dehydrogenase, mitochondrial	ACADVL	0.91±0.05	0.45±0.04	2.01
C2_5562	Very long-chain specific acyl-CoA dehydrogenase, mitochondrial	ACADVL	0.85±0.12	0.3±0.02	2.84
C2_7834	Vesicle-associated membrane protein-associated protein A	VAPA	1.27±0.17	0.74±0.05	1.72
C2_3642	Vesicle-associated membrane protein-associated protein B	VAPB	1.58±0.29	1.42±0.09	1.11
C2_4322	Vesicle-associated membrane protein-associated protein B/C	VAPB	1.73±0.33	1.16±0.09	1.48
C2_8296	Vesicle-fusing ATPase	NSF	1.18±0.11	1.55±0.07	0.76
C2_65101	Vesicle-fusing ATPase	NSF	1±0.2	0.96±0.22	1.04
C2_3873	Vesicular integral-membrane protein VIP36	LMAN2	1.11±0.06	0.59±0.05	1.88
C2_1304	Vigilin	HDLBP	1.02±0.03	1.18±0.23	0.87
C2_10632	Villin-1	VIL1	2.02±0.45	2.09±0.17	0.97
C2_61501	Villin-1	VIL1	0.9±0.11	0.93±0.09	0.97
C2_9594	Vinculin	VCL	1.31±0.13	1.92±0.11	0.68
C2_44280	Vinculin	VCL	0.94±0.05	1.28±0.09	0.73
FP335499	Vitamin K-dependent protein S	PROS1	1.04±0.04	1.03±0.13	1.02
C2_318	Voltage-dependent anion-selective channel protein 1	VDAC1	1.25±0.19	0.68±0.06	1.83
C2_661	Voltage-dependent anion-selective channel protein 2	VDAC2	1.55±0.31	0.46±0.04	3.35
C2_1494	Voltage-dependent anion-selective channel protein 2	VDAC2	1.64±0.43	0.47±0.04	3.49
C2_119048	Voltage-dependent anion-selective channel protein 3	VDAC3	2±0.48	0.39±0.02	5.09
C2_15635	von Willebrand factor A domain-containing protein 5A	VWA5A	0.89±0.05	0.64±0.02	1.39
C2_2397	von Willebrand factor A domain-containing protein 5A	VWA5A	0.77±0.08	0.35±0.03	2.21

C2_112999	V-set domain containing T-cell activation inhibitor 1	VTCN1	0.82±0.1	0.63±0.07	1.29
C2_3806	V-type proton ATPase catalytic subunit A	ATP6V1A	1±0.03	2.04±0.38	0.49
C2_1084	V-type proton ATPase subunit B	ATP6V0A4	0.99±0.04	1.63±0.52	0.61
C2_46438	V-type proton ATPase subunit C 1	ATP6V1C1	1.01±0.11	1.47±0.21	0.69
C2_5388	V-type proton ATPase subunit C 1-A	ATP6V1C1	1.26±0.16	2.4±0.08	0.53
C2_5241	V-type proton ATPase subunit E 1	ATP6V0E1	0.92±0.04	1.33±0.22	0.69
C2_1898	V-type proton ATPase subunit F	ATP6V1F	0.94±0.14	1.29±0.15	0.73
C2_18009	V-type proton ATPase subunit G 1	ATP6V1G1	1.38±0.16	4.08±1.24	0.34
C2_275	V-type proton ATPase subunit H	ATP6V1H	0.89±0.04	1.39±0.17	0.64
C2_9309	WASH complex subunit 7	KIAA1033	0.99±0.01	1.29±0.15	0.76
C2_9579	WASH complex subunit strumpellin	KIAA0196	0.82±0.06	1.27±0.07	0.65
C2_19854	WASH complex subunit strumpellin	KIAA0196	1.07±0.05	1.4±0.19	0.76
C2_27168	WD repeat and FYVE domain-containing protein 1	WDFY1	0.74±0.09	0.45±0.07	1.64
C2_2247	WD repeat domain phosphoinositide-interacting protein 2	WIPI2	0.98±0.06	0.87±0.11	1.13
C2_15764	WD repeat domain phosphoinositide-interacting protein 3	WDR45B	0.95±0.06	1.48±0.05	0.64
C2_1420	WD repeat-containing protein 1	WDR1	0.77±0.09	0.44±0.05	1.75
C2_15039	WD repeat-containing protein 26	WDR26	1.41±0.14	3.07±0.3	0.46
C2_97790	WD repeat-containing protein 26	WDR26	0.93±0.06	0.53±0.04	1.75
C2_5847	WD repeat-containing protein 5	WDR5	0.79±0.09	0.6±0.06	1.32
C2_1995	WD repeat-containing protein 61	WDR61	0.76±0.08	0.65±0.05	1.18
C2_7451	WD repeat-containing protein 82	WDR82	0.96±0.1	0.78±0.04	1.23
C2_3782	WD repeat-containing protein 91	WDR91	1.2±0.08	1.86±0.05	0.64
C2_67434	WW domain-binding protein 2	WBP2	0.86±0.05	0.93±0.05	0.93
C2_4369	Xaa-Pro aminopeptidase 1	XPNPEP1	0.86±0.09	0.86±0.09	1.00
C2_2468	Xaa-Pro aminopeptidase 2	XPNPEP2	0.52±0.16	0.42±0.06	1.24
C2_1116	Xaa-Pro dipeptidase	PEPD	0.77±0.16	0.9±0.09	0.85
C2_17124	Xanthine dehydrogenase/oxidase	XDH	0.87±0.12	0.81±0.03	1.08
C2_10177	Xylose isomerase	XYLA	0.85±0.12	0.9±0.1	0.94
C2_8299	Xylulose kinase	XYLB	0.97±0.06	1.44±0.18	0.67
C2_35539	Zinc finger protein RFP	TRIM27	0.85±0.06	0.87±0.04	0.97
C3_c25709	Zinc finger ZZ-type and EF-hand domain-containing protein 1	ZZEF1	0.88±0.08	0.91±0.08	0.97
C2_46479	Zinc finger ZZ-type and EF-hand domain-containing protein 1	ZZEF1	0.79±0.07	0.68±0.03	1.17
C2_16098	Zinc-binding alcohol dehydrogenase domain-containing protein 2	ZADH2	1.25±0.09	0.72±0.03	1.75
C2_90139	Zonadhesin	ZAN	0.78±0.08	0.59±0.03	1.32
C2_2605	Zonadhesin	ZAN	0.77±0.09	0.54±0.02	1.44
C2_39339	Zonadhesin	ZAN	0.6±0.14	0.37±0.04	1.63
