# Supplementary material for: Under control: how a dietary additive can restore the gut microbiome and proteomic profile, and improve disease resilience in a marine teleostean fish fed vegetable diets
Source: Microbiome. 2017 Dec 28;5:164. doi: 10.1186/s40168-017-0390-3 (PMC5745981; doi:10.1186/s40168-017-0390-3)
Supplement: Supplementary file 8 — List of proteins detected in anterior intestine samples. Data on protein expression are mean ± SEM of 6 fish fed the experimental diets. The number of the contig in the Sea Bream Database (http://nutrigroup-iats.org/seabreamdb) is indicated. (PDF 562 kb) [file 40168_2017_390_MOESM8_ESM.pdf]

Table S3. List of proteins detected in anterior intestine samples. Data on protein expression are mean ± SEM of 6 fish fed the experimental diets. The number of the contig in the Sea Bream Database (http://nutrigroup-iats.org/seabreamdb) is indicated.

			Diet			
**Contig**	**Protein Description**	**Symbol**	**D1**	**D2**	**D3**	**D4**
C2_299	14-3-3 protein beta/alpha-1	YWHAB	0.93±0.30	1.66±0.38	1.50±0.24	1.26±0.28
C2_268	14-3-3 protein epsilon	YWHAE	0.91±0.07	1.31±0.22	0.92±0.07	1.12±0.15
C2_2474	14-3-3 protein gamma-1	YWHAG	0.77±0.08	1.39±0.32	1.02±0.16	1.28±0.20
C2_1017	14-3-3 protein zeta	YWHAZ	0.65±0.11	0.91±0.09	0.90±0.13	0.83±0.09
C2_34474	14-3-3-like protein 2	YWHAQ	1.09±0.19	0.73±0.18	1.36±0.41	1.25±0.40
C2_4902	17-beta-hydroxysteroid dehydrogenase 14	HSD17B14	1.09±0.30	1.16±0.18	0.93±0.05	1.16±0.11
C2_15440	1-phosphatidylinositol phosphodiesterase	PLCA	1.43±0.14	0.77±0.05	0.63±0.14	0.97±0.26
C2_12986	1-phosphatidylinositol-4,5-bisphosphate phosphodiesterase delta-1	PLCD1	0.75±0.07	0.83±0.10	1.25±0.37	1.12±0.21
C2_3170	2,4-dienoyl-CoA reductase, mitochondrial	DECR1	0.97±0.17	1.08±0.28	1.71±0.60	1.34±0.32
C2_1520	26S protease regulatory subunit 10B	PSMC6	0.77±0.04	1.13±0.14	1.59±0.20	1.11±0.21
C2_1666	26S protease regulatory subunit 6A	PSMC3	1.27±0.13	1.36±0.11	1.95±0.40	1.45±0.16
C2_3002	26S protease regulatory subunit 7	PSMC2	1.32±0.17	0.71±0.07	0.81±0.20	0.96±0.14
C2_514	26S protease regulatory subunit 8	PSMC5	1.42±0.22	1.17±0.23	1.38±0.23	1.64±0.45
C2_2728	26S proteasome non-ATPase regulatory subunit 1	PSMD1	0.84±0.11	1.01±0.11	1.21±0.15	1.00±0.18
C2_2261	26S proteasome non-ATPase regulatory subunit 11	PSMD11	1.14±0.14	0.95±0.14	1.47±0.46	1.59±0.25
C2_1392	26S proteasome non-ATPase regulatory subunit 12	PSMD12	0.91±0.11	1.03±0.24	1.28±0.26	1.37±0.26
C2_790	26S proteasome non-ATPase regulatory subunit 13	PSMD13	0.65±0.12	0.82±0.18	0.84±0.17	0.82±0.16
C2_4556	26S proteasome non-ATPase regulatory subunit 2	PSMD2	0.79±0.07	1.15±0.24	1.13±0.19	1.04±0.11
C2_1006	26S proteasome non-ATPase regulatory subunit 3	PSMD3	0.81±0.09	0.87±0.08	0.99±0.15	0.89±0.14
C2_8032	26S proteasome non-ATPase regulatory subunit 6	PSMD6	0.92±0.06	1.06±0.16	1.19±0.25	1.24±0.21
C2_364	26S proteasome non-ATPase regulatory subunit 7	PSMD7	0.78±0.11	1.21±0.23	1.29±0.17	1.14±0.27
C2_58026	26S proteasome non-ATPase regulatory subunit 8	PSMD8	0.98±0.06	0.93±0.13	0.88±0.24	1.03±0.09
C2_6795	2-acylglycerol O-acyltransferase 2-A	MOGAT2	0.96±0.16	0.67±0.09	0.96±0.15	1.06±0.11
C2_8379	2-amino-3-carboxymuconate-6-semialdehyde decarboxylase	ACMSD	1.08±0.16	1.68±0.18	1.31±0.32	1.18±0.18
C2_34981	3'(2'),5'-bisphosphate nucleotidase 1	BPNT1	1.12±0.27	1.82±0.17	1.94±0.32	1.46±0.34
C2_7568	3-hydroxyacyl-CoA dehydrogenase type-2	HSD17B10	1.01±0.18	1.05±0.29	1.41±0.14	1.89±0.55
C2_1004	3-hydroxyanthranilate 3,4-dioxygenase	HAAO	1.03±0.14	1.12±0.12	1.14±0.13	1.04±0.18
C2_850	3-hydroxybutyrate dehydrogenase type 2	BDH2	1.75±0.18	1.21±0.10	1.05±0.15	1.23±0.16
C2_1580	3-hydroxyisobutyrate dehydrogenase, mitochondrial	HIBADH	1.95±0.29	1.46±0.29	1.27±0.22	1.07±0.15
C2_4404	3-hydroxyisobutyryl-CoA hydrolase, mitochondrial	HIBCH	1.37±0.19	1.14±0.14	1.38±0.37	1.33±0.27
C2_7520	3-ketoacyl-CoA thiolase B, peroxisomal	Acaa1b	1.12±0.28	0.92±0.17	1.20±0.30	1.27±0.48
C2_1174	3-ketoacyl-CoA thiolase, mitochondrial	ACAA2	0.70±0.14	1.07±0.33	1.26±0.55	0.98±0.30
C2_742	3-oxo-5-beta-steroid 4-dehydrogenase	AKR1D1	1.15±0.04	1.00±0.05	1.07±0.14	1.32±0.18
C2_8805	40S ribosomal protein S10	RPS10	0.68±0.15	0.77±0.27	1.70±0.47	0.97±0.30
C2_14171	40S ribosomal protein S13	RPS13	0.63±0.09	0.75±0.11	1.58±0.39	0.97±0.34
C2_414	40S ribosomal protein S15a	RPS15A	0.53±0.03	0.93±0.18	1.37±0.35	0.82±0.16
C2_7337	40S ribosomal protein S16	RPS16	0.73±0.08	0.89±0.21	1.50±0.30	1.08±0.11
C2_971	40S ribosomal protein S17	RPS17	1.04±0.26	1.26±0.39	6.43±2.19	1.80±0.75
C2_9151	40S ribosomal protein S18	RPS18	0.50±0.08	0.96±0.32	1.83±0.51	1.05±0.29
C2_955	40S ribosomal protein S19	RPS19	0.58±0.05	1.44±0.48	3.17±1.08	1.94±0.71
C2_684	40S ribosomal protein S2	RPS2	0.69±0.06	0.93±0.16	1.45±0.31	1.09±0.32
C2_232	40S ribosomal protein S20	RPS20	1.32±0.34	1.08±0.17	2.58±0.79	1.53±0.21
C2_1271	40S ribosomal protein S24	RPS24	0.88±0.04	0.99±0.09	1.70±0.36	1.35±0.29
C2_310	40S ribosomal protein S25	RPS25	0.80±0.03	0.86±0.20	2.01±0.51	1.39±0.39
C2_698	40S ribosomal protein S26	RPS26	0.74±0.10	0.91±0.14	1.46±0.26	1.09±0.19
C2_3275	40S ribosomal protein S28	RPS28	0.66±0.18	0.59±0.19	1.23±0.42	0.85±0.15
C2_23479	40S ribosomal protein S29	RPS29	0.51±0.07	0.63±0.16	1.19±0.43	1.39±0.36
C2_17873	40S ribosomal protein S3	RPS3	0.63±0.04	0.82±0.16	1.34±0.26	0.90±0.23
C2_2139	40S ribosomal protein S3a	Rps3a1	0.82±0.06	0.67±0.11	1.11±0.16	1.15±0.23
C2_593	40S ribosomal protein S4	GM15013	0.61±0.05	0.85±0.13	1.71±0.35	1.08±0.33
C2_433	40S ribosomal protein S5	RPS5	0.50±0.10	0.95±0.34	1.79±0.64	0.87±0.17
C2_164	40S ribosomal protein S6	RPS6	0.64±0.12	0.59±0.10	1.12±0.22	1.24±0.39
C2_133	40S ribosomal protein S7	RPS7	0.83±0.10	0.85±0.10	1.66±0.30	1.37±0.43
C2_4273	40S ribosomal protein S8	RPS8	0.70±0.06	0.69±0.14	1.34±0.27	1.03±0.28
C2_252	40S ribosomal protein S9	RPS9	0.47±0.04	0.74±0.18	1.29±0.32	1.05±0.28
C2_16	40S ribosomal protein SA	RPSA	0.86±0.11	0.76±0.15	1.24±0.22	0.93±0.21
C2_4721	4-aminobutyrate aminotransferase, mitochondrial	ABAT	1.19±0.11	1.20±0.12	1.88±0.40	1.38±0.37
C2_2062	4F2 cell-surface antigen heavy chain	SLC3A2	1.02±0.06	1.05±0.15	1.75±0.40	0.96±0.09
C2_5124	5'(3')-deoxyribonucleotidase, mitochondrial	NT5M	0.93±0.07	1.51±0.14	1.28±0.13	1.14±0.14
C2_58937	5-oxoprolinase	OPLAH	0.94±0.20	1.17±0.02	1.50±0.17	1.07±0.18
C2_4852	6.8 kDa mitochondrial proteolipid	C14orf2	0.70±0.21	1.39±0.23	1.64±0.45	0.96±0.27
C2_5222	60 kDa heat shock protein, mitochondrial	HSPD1	1.00±0.11	0.81±0.12	1.05±0.16	1.38±0.21
C2_47	60S acidic ribosomal protein P0	RPLP0	0.83±0.11	0.84±0.12	1.41±0.34	1.29±0.17
C2_46323	60S acidic ribosomal protein P1	Rplp1	0.71±0.16	1.49±0.06	1.66±0.28	1.00±0.26
C2_6923	60S acidic ribosomal protein P2	RPLP2	1.26±0.11	3.01±0.42	2.95±0.80	1.69±0.29
C2_167	60S ribosomal protein L10	RPL10	0.52±0.05	0.70±0.23	1.31±0.41	1.09±0.27
C2_67	60S ribosomal protein L10a	RPL10A	0.61±0.17	1.12±0.45	1.36±0.49	1.37±0.33
C2_236	60S ribosomal protein L11	RPL11	0.71±0.23	1.12±0.45	1.17±0.38	1.65±0.56
C2_453	60S ribosomal protein L13	RPL13	0.74±0.17	0.83±0.23	1.50±0.32	1.42±0.21
C2_441	60S ribosomal protein L13a	RPL13A	0.62±0.19	1.26±0.55	1.25±0.45	1.41±0.34
C2_142	60S ribosomal protein L14	RPL14	1.09±0.14	1.08±0.27	1.62±0.47	1.70±0.18
C2_279	60S ribosomal protein L15	RPL15	0.58±0.22	1.60±0.89	1.71±0.73	1.45±0.42
C2_1244	60S ribosomal protein L17	RPL17	0.63±0.16	1.35±0.67	1.39±0.42	1.50±0.32

C2_842	60S ribosomal protein L18	RPL18	0.77±0.16	1.18±0.41	1.28±0.53	1.38±0.41
C2_12358	60S ribosomal protein L18a	RPL18A	0.61±0.27	1.40±0.75	1.71±0.76	1.57±0.53
C2_700	60S ribosomal protein L19	RPL19	0.50±0.14	1.14±0.49	1.36±0.37	1.36±0.30
C2_94336	60S ribosomal protein L21	RPL21	0.89±0.12	1.13±0.39	1.76±0.55	1.79±0.30
C2_39656	60S ribosomal protein L22	RPL22	0.72±0.12	0.86±0.06	1.22±0.30	1.14±0.30
C2_373	60S ribosomal protein L23	RPL23	0.88±0.12	1.28±0.50	2.07±0.56	1.89±0.32
C2_1007	60S ribosomal protein L23a	Rpl23a	0.79±0.10	1.05±0.23	2.51±0.82	1.72±0.31
C2_119969	60S ribosomal protein L24	RPL24	0.41±0.12	1.51±1.16	1.85±0.72	1.41±0.32
C2_392	60S ribosomal protein L26	RPL26	0.60±0.15	1.17±0.52	1.40±0.47	1.38±0.43
C2_121623	60S ribosomal protein L27	RPL27	0.69±0.09	0.82±0.29	1.64±0.47	1.09±0.22
C2_343	60S ribosomal protein L27a	RPL27A	0.90±0.13	1.04±0.22	2.23±0.54	1.87±0.30
C2_12	60S ribosomal protein L3	RPL3	0.67±0.10	0.97±0.36	1.13±0.35	1.21±0.36
C2_17126	60S ribosomal protein L30	RPL30	0.94±0.09	1.21±0.27	1.89±0.47	1.04±0.11
C2_111961	60S ribosomal protein L31	RPL31	0.93±0.31	0.69±0.17	1.06±0.26	1.75±0.29
C2_361	60S ribosomal protein L32	Rpl32	0.78±0.15	1.25±0.52	1.38±0.40	1.60±0.44
C2_72598	60S ribosomal protein L34	Rpl34	0.44±0.08	1.39±0.54	3.26±1.37	1.27±0.45
C2_89835	60S ribosomal protein L35	RPL35	0.77±0.08	1.01±0.24	1.74±0.44	1.51±0.25
C2_3033	60S ribosomal protein L35a	RPL35A	0.98±0.18	1.45±0.45	2.30±0.58	2.40±0.40
C2_9484	60S ribosomal protein L38	RPL38	0.81±0.07	0.91±0.38	2.70±1.00	1.43±0.50
C2_25	60S ribosomal protein L4	RPL4	0.63±0.19	1.16±0.51	1.60±0.57	1.77±0.48
C2_143	60S ribosomal protein L5	RPL5	0.64±0.19	0.93±0.27	0.93±0.23	1.35±0.39
C2_827	60S ribosomal protein L6	RPL6	0.56±0.17	1.19±0.53	1.42±0.45	1.69±0.56
C2_434	60S ribosomal protein L7	RPL7	0.70±0.20	1.45±0.69	1.83±0.81	1.76±0.55
C2_98	60S ribosomal protein L7a	RPL7A	0.92±0.09	1.02±0.24	1.48±0.38	1.06±0.10
C2_174	60S ribosomal protein L8	RPL8	0.80±0.14	1.20±0.33	1.98±0.55	1.93±0.35
C2_156	60S ribosomal protein L9	RPL9	0.68±0.13	1.08±0.37	1.44±0.55	1.81±0.57
C2_11947	6-phosphofructo-2-kinase/fructose-2,6-biphosphatase 4	PFKFB4	17.22±16.56	32.12±19.84	27.88±17.61	17.32±16.54
C2_3317	6-phosphofructokinase type C	PFKP	0.74±0.13	1.14±0.07	1.03±0.10	0.80±0.14
C2_4969	6-phosphofructokinase, liver type	PFKL	1.08±0.12	0.81±0.05	1.17±0.23	1.26±0.28
C2_531	6-phosphogluconate dehydrogenase, decarboxylating	PGD	0.88±0.07	1.32±0.18	1.14±0.18	1.25±0.14
C2_6550	6-phosphogluconolactonase	PGLS	2.45±0.57	1.53±0.26	1.42±0.16	1.41±0.19
C2_14692	6-pyruvoyl tetrahydrobiopterin synthase	PTS	1.63±0.21	1.12±0.15	0.87±0.07	1.29±0.22
C2_25027	78 kDa glucose-regulated protein	HSPA5	0.85±0.08	1.23±0.14	2.71±0.93	1.24±0.23
C2_14888	78 kDa glucose-regulated protein	HSPA5	1.05±0.07	1.22±0.19	1.73±0.36	1.28±0.11
C2_42	90kDa heat shock protein beta	HSP90AB1	0.65±0.05	0.91±0.14	1.09±0.37	1.10±0.24
C2_40874	Abhydrolase domain-containing protein 14B	ABHD14B	0.86±0.17	1.00±0.24	1.18±0.26	1.20±0.28
C2_7286	Abhydrolase domain-containing protein 14B	ABHD14B	0.90±0.34	0.84±0.24	0.87±0.28	0.99±0.22
C2_13751	Acetolactate synthase-like protein	ILVBL	0.93±0.14	0.90±0.09	1.65±0.22	1.63±0.54
C2_98839	Acetolactate synthase-like protein	ILVBL	1.25±0.23	1.00±0.18	1.59±0.34	1.44±0.19
C2_10641	Acetyl-coenzyme A synthetase, cytoplasmic	ACSS2	0.90±0.26	0.73±0.20	0.86±0.31	1.21±0.38
C2_2879	Acid ceramidase	ASAH1	1.25±0.12	1.27±0.16	0.98±0.19	1.30±0.37
C2_3009	Acid sphingomyelinase-like phosphodiesterase 3b	SMPDL3B	2.10±0.51	1.20±0.24	1.00±0.20	1.02±0.23
C2_18917	Acidic leucine-rich nuclear phosphoprotein 32 family member E	Anp32e	0.93±0.16	1.48±0.16	1.01±0.22	0.77±0.15
C3_lrc63576	Acidic mammalian chitinase	CHIA	3.64±1.16	1.53±0.60	1.21±0.89	0.92±0.30
C2_18809	Aconitate hydratase, mitochondrial	ACO2	1.11±0.14	1.21±0.09	2.23±0.68	1.52±0.31
C2_210	Actin, cytoplasmic 1	ACTB	0.96±0.12	1.25±0.18	2.17±0.65	1.79±0.68
C2_102126	Actin, cytoplasmic 2	ACTG1	1.36±0.10	1.96±0.36	4.05±1.94	4.98±2.72
C2_8398	Actin-related protein 10	ACTR10	0.73±0.12	1.42±0.44	1.37±0.32	1.26±0.50
C2_4166	Actin-related protein 2/3 complex subunit 3	ARPC3	0.66±0.07	1.90±0.74	1.58±0.58	1.74±0.59
C2_427	Actin-related protein 2/3 complex subunit 4	ARPC4	0.80±0.05	1.07±0.16	1.16±0.16	1.07±0.30
C2_503	Actin-related protein 2/3 complex subunit 5	ARPC5	0.82±0.09	1.79±0.46	1.57±0.34	1.51±0.19
C2_3453	Actin-related protein 2-A	ACTR2	0.93±0.14	1.29±0.22	1.19±0.03	1.18±0.28
C2_1771	Actin-related protein 3	ACTR3	0.76±0.13	1.03±0.30	1.05±0.29	1.00±0.23
C2_2259	Activated RNA polymerase II transcriptional coactivator p15	SUB1	2.14±0.31	1.22±0.20	0.96±0.19	1.83±0.52
C2_20973	Active breakpoint cluster region-related protein	ABR	1.83±0.88	1.29±0.29	1.35±0.15	1.22±0.26
C2_11239	Acyl carrier protein, mitochondrial	NDUFAB1	1.03±0.07	1.27±0.16	1.47±0.33	0.84±0.12
C2_2532	Acylamino-acid-releasing enzyme	APEH	1.70±0.20	1.01±0.15	0.86±0.15	1.37±0.34
C2_47266	Acyl-CoA dehydrogenase family member 11	ACAD11	1.46±0.16	0.88±0.20	0.98±0.15	1.43±0.52
C2_3692	Acyl-CoA dehydrogenase family member 9, mitochondrial	ACAD9	1.57±0.22	1.05±0.23	1.06±0.20	1.17±0.28
C2_8427	Acyl-CoA synthetase family member 2, mitochondrial	ACSF2	1.46±0.17	1.09±0.11	1.34±0.38	0.98±0.11
C2_649	Acyl-CoA-binding protein	DBI	0.60±0.12	0.66±0.25	1.14±0.54	0.97±0.40
C2_1332	Acyl-CoA-binding protein homolog	DBI	0.49±0.06	1.01±0.35	1.07±0.34	0.89±0.30
C2_9418	Acyl-coenzyme A thioesterase 3	ACOT1	0.88±0.12	1.22±0.26	1.04±0.21	0.89±0.09
C2_4253	Acyl-protein thioesterase 2	LYPLA2	0.59±0.17	0.86±0.17	0.90±0.29	1.10±0.15
C2_11692	Adenine phosphoribosyltransferase	APRT	1.03±0.18	1.32±0.17	1.62±0.46	2.07±0.55
C2_7078	Adenosine deaminase	ADA	1.54±0.16	0.97±0.09	0.85±0.09	1.26±0.23
C2_5126	Adenosine kinase	ADK	1.37±0.14	1.30±0.07	1.10±0.09	1.15±0.06
C2_39087	Adenosine kinase 1	ADK1	1.71±0.27	1.28±0.19	1.11±0.07	1.02±0.10
C2_2187	Adenosylhomocysteinase A	AHCY	2.63±0.60	1.49±0.20	1.34±0.28	1.87±0.52
C2_6546	Adenylosuccinate lyase	ADSL	1.26±0.20	1.27±0.11	1.08±0.05	0.97±0.10
C2_41283	Adenylyl cyclase-associated protein 1	CAP1	1.13±0.20	2.46±0.17	2.42±0.50	1.59±0.44
C2_75777	Adenylyl cyclase-associated protein 1	CAP1	1.73±0.28	1.61±0.24	1.53±0.31	1.32±0.14
C2_2038	ADP-ribosylation factor 4	ARF4	0.58±0.15	0.66±0.15	1.40±0.60	1.42±0.55
C2_12596	ADP-ribosylation factor GTPase-activating protein 2	ARFGAP2	1.99±0.30	1.66±0.34	1.48±0.54	1.02±0.32
C2_1966	ADP-ribosylation factor-like protein 1	ARL1	0.91±0.11	0.87±0.19	1.25±0.27	1.29±0.22
C2_3920	ADP-sugar pyrophosphatase	NUDT5	4.00±2.25	3.71±1.54	1.63±0.65	2.00±0.43
C2_970	Aflatoxin B1 aldehyde reductase member 2	AKR7A2	0.97±0.11	1.21±0.16	1.21±0.07	1.16±0.13
C2_4151	Agmatinase, mitochondrial	AGMAT	2.47±0.62	1.05±0.20	1.30±0.39	1.28±0.53

C2_9243	Alanine--glyoxylate aminotransferase 2-like 2	AGXT2	1.04±0.14	0.78±0.08	0.71±0.06	1.10±0.28
C2_4628	Alanyl-tRNA synthetase, cytoplasmic	AARS	1.56±0.22	1.30±0.13	1.20±0.13	1.35±0.12
C2_8373	Alanyl-tRNA synthetase, cytoplasmic	AARS	1.07±0.20	1.43±0.19	1.27±0.22	1.03±0.27
C2_996	Alcohol dehydrogenase [NADP+] B	AKR1A1	1.09±0.15	1.39±0.11	1.20±0.17	0.99±0.18
C2_189	Alcohol dehydrogenase 1	ADH1C	2.62±0.74	1.46±0.21	1.12±0.22	1.91±0.44
C2_288	Alcohol dehydrogenase class-3	ADH3	2.06±0.44	1.09±0.17	0.85±0.13	1.55±0.41
C2_43704	Alcohol dehydrogenase class-3 chain H	ADH3	2.62±0.50	1.71±0.40	1.67±0.38	2.88±0.82
C2_33526	Alcohol dehydrogenase class-3 chain L	ADH3	1.83±0.19	0.79±0.17	0.81±0.25	1.90±0.63
C2_2085	Aldehyde dehydrogenase family 1 member L1	ALDH1L1	1.02±0.29	1.53±0.17	1.17±0.15	1.14±0.22
C2_53791	Aldehyde dehydrogenase family 8 member A1	ALDH8A1	1.50±0.16	0.97±0.12	0.66±0.05	1.22±0.51
C2_77670	Aldehyde dehydrogenase family 8 member A1	ALDH8A1	1.59±0.47	1.79±0.41	1.22±0.35	2.05±0.53
C2_3814	Aldehyde dehydrogenase family 9 member A1	ALDH9A1	0.72±0.06	1.15±0.16	1.49±0.19	0.84±0.16
C2_399	Aldehyde dehydrogenase, mitochondrial	ALDH2	0.98±0.11	1.22±0.13	1.64±0.55	1.51±0.31
C2_11977	Aldehyde oxidase	AOX1	1.73±0.21	1.24±0.24	1.03±0.10	1.37±0.25
C2_83262	Aldehyde oxidase	AOX1	1.75±0.29	1.68±0.38	2.46±0.50	1.55±0.44
C2_97934	Aldehyde oxidase	AOX1	1.67±0.17	1.33±0.19	1.15±0.23	1.56±0.35
C2_3792	Aldose 1-epimerase	GALM	1.74±0.25	1.06±0.14	0.86±0.14	1.21±0.15
C2_1189	Aldose reductase	AKR1B1	1.21±0.12	1.55±0.25	1.72±0.19	2.35±0.80
C2_27912	Allograft inflammatory factor 1-like	AIF1L	0.68±0.22	1.32±0.48	0.60±0.19	1.08±0.18
C2_14294	Alpha-1-antitrypsin homolog	SERPINA1	1.66±0.40	1.28±0.23	0.96±0.28	1.30±0.36
C2_6331	Alpha-1-macroglobulin	A1M	1.27±0.24	1.39±0.24	0.96±0.31	1.12±0.36
C2_9982	Alpha-2-antiplasmin	SERPINF2	1.24±0.13	1.40±0.20	1.03±0.29	1.31±0.39
C2_22548	Alpha-2-HS-glycoprotein	AHSG	1.49±0.21	2.63±1.04	2.03±0.62	1.55±0.33
s_flp0005a11_f_1	Alpha-2-macroglobulin	A2M	1.33±0.28	1.09±0.14	0.85±0.25	1.20±0.37
C2_23524	Alpha-2-macroglobulin-like protein 1	A2ML1	1.26±0.29	0.82±0.16	0.83±0.07	0.80±0.17
C2_1801	Alpha-actinin-3	Actn3	1.08±0.20	2.83±0.88	2.43±0.87	1.32±0.17
C2_26557	Alpha-actinin-4	ACTN4	0.96±0.07	1.62±0.23	1.44±0.22	1.22±0.28
C2_1946	Alpha-aminoadipic semialdehyde dehydrogenase	ALDH7A1	1.94±0.28	1.30±0.20	1.15±0.19	1.68±0.37
C2_16242	Alpha-aspartyl dipeptidase	AAD-A	1.13±0.11	1.06±0.15	1.52±0.37	1.64±0.31
C2_440	Alpha-enolase	ENO1	0.97±0.12	1.59±0.18	1.25±0.14	1.09±0.19
C2_23595	Alpha-glucosidase 2	GAA	1.70±0.46	1.08±0.14	0.91±0.19	0.95±0.21
C2_17154	Alpha-mannosidase 2C1	MAN2C1	1.26±0.15	0.83±0.07	0.72±0.16	1.05±0.25
C2_52709	Alpha-methylacyl-CoA racemase	AMACR	1.70±0.22	0.96±0.16	0.96±0.24	0.90±0.21
C2_7302	Alpha-N-acetylglucosaminidase	NAGLU	1.62±0.30	0.92±0.21	0.96±0.33	1.27±0.42
C2_2739	Amiloride-sensitive amine oxidase [copper-containing]	AOC1	1.37±0.08	1.34±0.09	1.21±0.12	1.16±0.06
C2_7596	Amine oxidase [flavin-containing]	MAOB	0.70±0.06	0.90±0.18	1.53±0.39	0.81±0.16
C2_23714	Amine oxidase [flavin-containing] A	MAOA	1.68±0.28	1.15±0.29	1.15±0.27	1.46±0.35
C2_616	Aminoacyl tRNA synthase complex-interacting multifunctional protein 1	AIMP1	1.04±0.18	1.15±0.16	0.98±0.15	0.94±0.14
C2_1372	Aminoacylase-1	ACY1	1.71±0.19	1.15±0.16	0.97±0.14	1.41±0.32
C2_73930	Aminoglycoside phosphotransferase domain-containing protein 1	HYKK	1.47±0.20	5.42±2.27	2.29±1.02	2.39±0.77
C3_c17712	Aminomethyltransferase, mitochondrial	AMT	1.11±0.12	1.15±0.09	1.28±0.07	1.21±0.31
C2_7522	Aminopeptidase N	ANPEP	1.87±0.28	1.30±0.29	0.85±0.17	1.17±0.41
C2_16955	AMP deaminase 2	AMPD2	0.71±0.09	1.18±0.34	0.85±0.08	0.92±0.11
C2_94051	Angiotensin-converting enzyme	ACE	2.43±0.37	1.26±0.22	0.91±0.26	1.24±0.34
C2_31056	Angiotensin-converting enzyme	ACE	2.66±0.55	1.34±0.32	1.08±0.37	1.11±0.38
C2_49201	Angiotensin-converting enzyme	ACE	2.32±0.37	1.04±0.28	0.71±0.23	0.92±0.35
C2_2687	Angiotensin-converting enzyme	ACE	1.96±0.30	1.24±0.30	0.86±0.26	1.08±0.35
C2_1538	Angiotensin-converting enzyme 2	ACE2	2.22±0.41	0.83±0.09	0.81±0.26	1.03±0.24
C2_89317	Angiotensin-converting enzyme 2	ACE2	2.08±0.41	0.71±0.10	0.73±0.31	0.90±0.24
C2_1919	Anionic trypsin-2	PRSS2	2.74±0.46	0.84±0.12	0.86±0.30	1.59±0.50
C2_98249	Ankyrin repeat and FYVE domain-containing protein 1	ANKFY1	1.33±0.30	0.97±0.21	0.99±0.21	1.27±0.19
C3_c47959	Ankyrin-3	ANK3	0.84±0.16	1.33±0.30	0.81±0.19	0.95±0.08
C2_2096	Annexin A11	ANXA11	0.93±0.21	1.18±0.21	1.03±0.22	1.00±0.17
C2_3972	Annexin A13	ANXA13	0.82±0.13	0.91±0.12	1.33±0.42	1.00±0.17
C2_2251	Annexin A2	ANXA2	0.96±0.06	2.92±0.97	1.34±0.76	1.03±0.13
C2_5883	Annexin A2-A	ANXA2-A	0.71±0.14	1.79±0.90	1.86±0.37	1.24±0.22
C2_930	Annexin A3	ANXA3	1.22±0.17	1.02±0.18	1.51±0.18	1.10±0.19
C2_88	Annexin A4	ANXA4	1.34±0.25	1.06±0.24	1.29±0.31	1.14±0.34
C2_772	Annexin A5	ANXA5	0.83±0.14	1.24±0.21	1.24±0.30	0.86±0.13
C2_4218	Anterior gradient protein 2 homolog	AGR2	1.42±0.16	1.05±0.11	1.32±0.30	1.62±0.21
C3_c16373	Antithrombin-III	SERPINC1	1.27±0.14	1.44±0.25	1.20±0.21	1.29±0.27
C2_37278	AP-1 complex subunit beta-1	AP1B1	0.96±0.14	1.27±0.11	1.38±0.13	1.35±0.21
C2_28545	AP-1 complex subunit beta-1	AP1B1	1.10±0.05	1.25±0.14	0.99±0.12	1.13±0.13
C2_11830	AP-1 complex subunit beta-1	AP1B1	0.79±0.08	0.88±0.20	1.00±0.28	1.08±0.16
C2_8787	AP-1 complex subunit gamma-1	AP1G1	0.78±0.14	1.36±0.23	1.38±0.19	0.92±0.12
C2_2248	AP-1 complex subunit mu-2	AP1M2	0.93±0.14	1.01±0.07	0.94±0.13	0.90±0.12
C2_13693	AP-2 complex subunit alpha-1	AP2A1	0.91±0.14	1.13±0.08	0.80±0.09	0.86±0.14
C2_5784	AP-2 complex subunit beta	Ap2b1	1.42±0.11	1.22±0.18	0.92±0.13	1.19±0.18
C2_79275	AP-3 complex subunit delta-1	AP3D1	0.92±0.16	0.72±0.17	0.85±0.23	0.91±0.24
C2_6208	Apical endosomal glycoprotein	MAMDC4	1.28±0.12	0.70±0.09	0.65±0.11	0.86±0.19
C2_120884	Apolipoprotein A-I	APOA1	0.74±0.07	3.18±1.30	1.88±0.63	1.47±0.38
C2_4177	Apolipoprotein A-I-binding protein	APOA1BP	1.09±0.18	1.33±0.16	0.90±0.13	0.89±0.16
C2_23243	Apolipoprotein A-IV	APOA4	0.55±0.08	0.38±0.07	1.42±0.69	1.32±0.56
C3_lrc60236	Apolipoprotein A-IV-like isoform X1	APOA4	0.61±0.15	0.47±0.08	1.26±0.64	0.78±0.24
C3_c28587	Apolipoprotein B-100	APOB	2.05±0.51	1.83±0.32	0.92±0.13	1.15±0.14
C2_15164	Apolipoprotein B-100	APOB	1.08±0.16	0.90±0.07	1.56±0.32	1.34±0.21
C2_5614	Apolipoprotein B-100	APOB	0.85±0.03	0.85±0.12	1.26±0.22	1.07±0.16

C2_28992	Apolipoprotein B-100	APOB	1.73±0.41	1.19±0.25	1.58±0.36	1.72±0.47
C3_c2363	Apolipoprotein B-100-like	APOB-L	1.29±0.15	1.39±0.26	0.56±0.10	0.65±0.11
C2_5591	Apolipoprotein Eb	APOEB	0.68±0.03	0.53±0.04	1.38±0.60	1.40±0.49
C2_9385	Apoptosis inhibitor 5	API5	0.80±0.18	0.99±0.09	1.06±0.12	0.76±0.08
C2_322	Apoptosis-associated speck-like protein containing a CARD	PYCARD	0.75±0.19	1.60±0.55	0.83±0.26	1.23±0.40
C2_1626	Apoptosis-associated speck-like protein containing a CARD	PYCARD	0.79±0.14	1.07±0.26	1.15±0.31	0.88±0.24
C2_6260	Apoptosis-inducing factor 1, mitochondrial	AIFM1	1.23±0.18	1.18±0.26	1.11±0.11	1.15±0.25
C2_6887	Apoptosis-inducing factor 3	AIFM3	0.76±0.34	2.45±0.64	1.78±0.22	1.12±0.26
C2_489	Arachidonate 12-lipoxygenase, 12R-type	ALOX12B	1.52±0.36	1.04±0.14	1.05±0.15	0.93±0.15
C2_857	Arsenite methyltransferase	AS3MT	0.61±0.07	1.04±0.29	1.41±0.36	1.07±0.30
C2_3390	Asparaginyl-tRNA synthetase, cytoplasmic	NARS	1.35±0.40	0.82±0.13	1.19±0.16	1.14±0.21
C2_14096	Aspartate aminotransferase, cytoplasmic	GOT1	1.30±0.12	0.95±0.11	0.81±0.21	0.97±0.11
C2_645	Aspartate aminotransferase, mitochondrial	GOT2	2.00±0.33	1.02±0.14	0.98±0.22	1.38±0.34
C2_4570	Aspartoacylase	ASPA	1.65±0.12	1.01±0.17	0.88±0.21	1.11±0.21
C2_1059	Aspartoacylase-2B	ACY3	1.54±0.23	1.00±0.07	0.99±0.16	1.24±0.18
C2_15853	Aspartyl aminopeptidase	DNPEP	2.21±0.39	0.95±0.22	0.63±0.15	1.71±0.55
C2_2153	Aspartyl-tRNA synthetase, cytoplasmic	ARSA	1.03±0.06	0.85±0.05	1.00±0.16	1.16±0.14
C2_176	ATP synthase subunit b, mitochondrial	ATP5A1	1.00±0.31	0.89±0.21	1.35±0.39	0.94±0.31
C2_2323	ATP-binding cassette sub-family E member 1	ABCE1	1.16±0.11	1.03±0.06	1.41±0.32	1.08±0.11
C2_1676	ATP-binding cassette sub-family F member 1	ABCF1	1.35±0.30	1.11±0.25	1.71±0.22	1.39±0.24
C3_c19506	ATP-citrate synthase	ACLY	0.97±0.10	0.97±0.05	1.00±0.17	1.34±0.22
C2_8958	ATP-citrate synthase	ACLY	0.72±0.05	0.90±0.08	1.41±0.47	1.29±0.40
C2_1283	ATP-dependent RNA helicase DDX39A	DDX39A	0.54±0.09	1.24±0.35	1.35±0.36	1.25±0.29
C2_214	Bactericidal permeability-increasing protein	BPI	1.16±0.13	1.32±0.22	1.07±0.24	1.11±0.24
C2_1409	Barrier-to-autointegration factor	BANF1	1.59±0.28	2.29±0.52	3.03±0.50	1.65±0.66
C2_10232	Barrier-to-autointegration factor-like protein	BANF2	2.58±1.23	2.94±0.60	3.89±1.28	1.51±0.62
C2_8263	Beta,beta-carotene 15,15'-monooxygenase	BCO1	0.95±0.19	1.03±0.24	1.20±0.19	1.30±0.36
C2_28	Beta-2-microglobulin	B2M	1.44±0.28	1.26±0.21	1.73±0.26	1.46±0.29
C2_19834	Beta-actin-like protein 2	ACTBL2	0.96±0.06	0.93±0.27	1.90±0.72	1.55±0.57
C2_69899	Beta-centractin	ACTR1B	0.89±0.15	0.74±0.07	1.29±0.43	1.01±0.21
C2_370	Beta-enolase	ENO3	1.45±0.33	1.69±0.25	1.21±0.15	1.02±0.20
C2_26906	Beta-galactosidase-1-like protein 2	GLB1L2	1.08±0.19	1.22±0.20	1.08±0.27	1.38±0.43
C2_8457	Beta-glucuronidase	GUSB	1.56±0.17	1.13±0.24	1.00±0.17	1.44±0.61
C2_3871	Beta-hexosaminidase subunit beta	HEXB	1.84±0.38	1.10±0.16	0.86±0.19	1.71±0.70
C2_79951	Betaine--homocysteine S-methyltransferase 2	BHMT	1.30±0.44	1.30±0.21	0.92±0.20	0.99±0.30
C2_5459	Beta-ureidopropionase	UPB1	1.74±0.09	1.01±0.18	0.88±0.22	1.32±0.31
C2_3176	Bifunctional 3'-phosphoadenosine 5'-phosphosulfate synthase 2	PAPSS2	1.03±0.12	0.77±0.03	1.52±0.41	1.40±0.25
C2_4884	Bifunctional aminoacyl-tRNA synthetase	EPRS	0.97±0.17	0.75±0.11	0.77±0.19	1.20±0.22
C2_48204	Bifunctional aminoacyl-tRNA synthetase	EPRS	0.55±0.22	1.10±0.29	1.29±0.45	1.02±0.18
C2_6394	Bifunctional polynucleotide phosphatase/kinase	PNKP	1.20±0.24	1.28±0.15	1.21±0.22	1.29±0.33
C2_4731	Bifunctional protein NCOAT	MGEA5	0.77±0.07	0.89±0.20	1.25±0.47	1.26±0.37
C2_4254	Bifunctional purine biosynthesis protein PURH	ATIC	1.17±0.09	1.13±0.12	0.90±0.08	1.08±0.13
C2_73027	Bile salt-activated lipase	CEL	1.34±0.19	0.65±0.10	0.69±0.07	0.68±0.12
C2_29998	Biotinidase	BTD	1.51±0.16	1.46±0.21	1.17±0.12	1.33±0.24
C2_885	Bleomycin hydrolase	BLMH	2.04±0.29	1.06±0.18	0.95±0.16	1.44±0.33
C2_112893	BOLA class I histocompatibility antigen, alpha chain BL3-7	HA1B	1.28±0.21	1.38±0.16	2.00±0.71	1.35±0.47
C2_15564	Brain-specific angiogenesis inhibitor 1-associated protein 2-like protein 2	BAIAP2L2	0.86±0.16	0.87±0.12	0.82±0.15	1.02±0.25
C2_27587	Butyrophilin-like protein 2	BTNL2	1.25±0.07	0.91±0.19	1.24±0.29	1.17±0.21
C2_5533	CAAX prenyl protease 1 homolog	ZMPSTE24	1.54±0.27	1.09±0.24	1.00±0.30	1.48±0.47
C2_48101	Cadherin EGF LAG seven-pass G-type receptor 1	CELSR1	1.97±0.45	1.60±0.32	1.50±0.40	1.22±0.15
C2_2045	Cadherin-1	CDH1	1.60±0.24	1.11±0.16	1.15±0.14	1.52±0.27
C2_3038	Cadherin-17	CDH17	1.27±0.17	1.06±0.16	1.83±0.44	1.07±0.13
C2_20741	Cadherin-23	CDH23	1.99±0.70	0.66±0.15	0.34±0.07	0.61±0.21
C2_5783	Cadherin-related family member 2	CDHR2	1.92±0.27	0.94±0.25	0.70±0.14	1.02±0.26
C2_25329	Cadherin-related family member 2	CDHR2	2.04±0.26	1.16±0.20	1.22±0.27	1.20±0.36
C2_70214	Cadherin-related family member 2	CDHR2	1.40±0.30	0.99±0.07	1.18±0.31	1.06±0.21
C2_33603	Calcium/calmodulin-dependent protein kinase type 1	CAMK1	0.72±0.31	3.15±1.49	1.68±0.46	1.10±0.32
C2_54503	Calcium/calmodulin-dependent protein kinase type II delta chain	CAMK2D	1.26±0.29	1.33±0.23	0.91±0.10	0.96±0.19
C2_22563	Calcium/calmodulin-dependent protein kinase type II subunit gamma	CAMK2G	1.06±0.12	1.27±0.31	1.31±0.26	1.36±0.40
C2_1929	Calcium-binding protein p22	CHP1	1.44±0.17	0.98±0.15	1.34±0.23	1.39±0.29
C2_103184	Calmodulin	CALM1	0.53±0.06	1.31±0.16	1.13±0.18	0.93±0.22
C2_98055	Calmodulin-like protein 4	CALML4	0.88±0.23	1.06±0.30	1.08±0.41	2.06±0.73
C2_19770	Calnexin	CANX	0.84±0.08	1.46±0.39	1.82±0.45	1.08±0.16
C2_5497	Calpain small subunit 1	CAPNS1	0.99±0.12	1.59±0.44	2.09±1.28	1.04±0.26
C2_4520	Calpain-2 catalytic subunit	CAPN2	0.90±0.20	0.91±0.13	0.77±0.11	0.85±0.17
C2_1023	Calreticulin	CALR	0.86±0.08	1.24±0.13	2.01±0.35	1.31±0.31
C2_15813	cAMP-dependent protein kinase catalytic subunit alpha	PRKACA	0.74±0.16	1.03±0.10	0.90±0.15	0.69±0.10
C2_6010	cAMP-dependent protein kinase type II-alpha regulatory subunit	PRKAR2A	0.91±0.05	0.97±0.10	1.11±0.08	0.94±0.10
C2_45801	Caprin-2	CAPRIN2	1.21±0.27	1.31±0.39	1.06±0.24	1.07±0.46
C2_2482	Carbohydrate kinase domain-containing protein	CARKD	1.53±0.21	1.57±0.35	1.66±0.49	1.27±0.40
C2_1891	Carbonic anhydrase 1	CA1	0.91±0.14	2.03±0.26	1.69±0.37	1.52±0.37
C2_14726	Carbonyl reductase [NADPH] 1	CBR1	0.73±0.08	1.33±0.31	1.09±0.25	0.91±0.15
C2_1812	Carboxymethylenebutenolidase homolog	CMBL	2.32±0.49	1.25±0.25	0.92±0.22	1.37±0.33
C2_936	Carboxypeptidase A1	CPA1	2.27±0.46	0.75±0.17	1.08±0.67	2.80±1.16

C2_5650	Carboxypeptidase A2	CPA2	1.73±0.20	0.91±0.10	0.85±0.29	1.74±0.64
C2_2018	Carboxypeptidase B	CPB1	2.13±0.25	0.99±0.14	1.03±0.38	1.94±0.64
C2_4458	Carboxypeptidase O	CPO	2.02±0.28	1.41±0.35	0.92±0.29	1.05±0.33
C2_30675	Carcinoembryonic antigen-related cell adhesion molecule 1	CEACAM1	1.51±0.33	1.29±0.26	0.81±0.27	0.93±0.21
C2_6038	Carnitine O-acetyltransferase	CRAT	0.95±0.14	1.06±0.10	1.30±0.24	1.12±0.19
C2_4920	Carnitine O-palmitoyltransferase 1, liver isoform	CPT1A	1.82±0.56	2.19±0.64	2.82±0.92	3.00±1.47
C2_2166	Carnitine O-palmitoyltransferase 2, mitochondrial	CPT2	0.59±0.14	0.84±0.14	1.43±0.59	0.72±0.10
C2_7099	Casein kinase I isoform alpha	CSNK1A1	1.19±0.22	0.81±0.17	0.92±0.14	0.89±0.10
C2_540	Casein kinase II subunit alpha	CSNK2A1	0.64±0.07	0.85±0.09	0.81±0.12	0.80±0.12
C2_16670	Caspase-1	CASP1	0.77±0.18	0.95±0.28	1.10±0.36	1.09±0.30
C2_85583	Caspase-1-A	CASP1-A	1.16±0.39	1.83±0.62	1.86±0.63	2.41±0.50
C2_5709	Caspase-6	CASP6	1.42±0.49	1.85±0.19	1.30±0.14	1.07±0.19
C2_997	Catalase	CAT	0.64±0.05	1.01±0.09	1.16±0.15	0.88±0.16
C2_872	Catechol O-methyltransferase domain-containing protein 1	COMTD1	2.39±0.36	1.65±0.25	1.28±0.13	1.87±0.38
C2_78432	Catenin alpha-1	CTNNA1	1.50±0.13	1.14±0.11	1.50±0.21	1.23±0.19
C2_13472	Catenin alpha-1	CTNNA1	0.94±0.07	1.04±0.21	1.77±0.51	1.53±0.47
C2_23957	Catenin delta-1	CTNND1	0.54±0.08	0.62±0.09	1.46±0.14	0.87±0.18
C2_11960	Cathepsin D	CTSD	2.32±0.46	1.04±0.20	0.75±0.16	1.34±0.58
C2_21	Cathepsin L	CTSL	2.15±0.48	1.73±0.37	1.55±0.40	1.22±0.27
C2_108360	Cathepsin S	CTSS	2.02±0.50	1.58±0.20	1.42±0.27	1.33±0.40
C2_2412	Cell differentiation protein RCD1 homolog	RQCD1	0.90±0.17	1.18±0.23	1.04±0.12	1.15±0.29
C2_12260	cGMP-dependent protein kinase 2	PRKG1	0.94±0.16	0.88±0.09	0.98±0.22	1.15±0.18
C2_1437	Chloride intracellular channel protein 4	CLIC4	0.55±0.12	1.45±0.36	0.93±0.13	0.84±0.25
C2_7684	Chloride intracellular channel protein 5	CLIC5	0.81±0.19	2.77±1.46	5.15±2.70	2.04±0.99
C2_5331	Choline kinase alpha	CHKA	1.83±0.35	1.05±0.20	0.94±0.21	1.13±0.40
C2_7664	Cholinesterase	BCHE	1.27±0.19	0.80±0.10	0.83±0.27	0.87±0.13
C2_12123	Chymotrypsin A	CTRB1	3.18±0.58	0.81±0.10	1.02±0.40	1.77±0.63
C2_4647	Chymotrypsin B	CTRB2	4.32±1.10	0.74±0.10	0.71±0.21	2.69±1.23
C2_8139	Chymotrypsin-C	CTRC	2.19±0.19	0.67±0.09	0.62±0.20	1.75±0.80
C2_9094	Chymotrypsin-like elastase family member 2A	CELA2A	2.14±0.45	0.52±0.08	0.91±0.33	1.41±0.59
C2_6507	Chymotrypsin-like elastase family member 3B	CELA3B	2.84±0.64	0.70±0.11	0.56±0.10	1.29±0.40
C2_4909	Chymotrypsin-like protease CTRL-1	CTRL	2.88±0.68	0.66±0.15	0.81±0.27	1.28±0.42
C2_2740	Citrate synthase, mitochondrial	CS	1.17±0.24	1.25±0.19	1.31±0.41	1.15±0.40
C2_15910	Clathrin heavy chain 1	CLTC	0.90±0.05	1.02±0.10	0.93±0.11	1.10±0.08
C2_18775	Clathrin heavy chain 1	CLTC	0.54±0.07	0.76±0.22	0.92±0.25	0.92±0.25
C2_13187	Clathrin heavy chain 1	CLTC	0.73±0.09	0.85±0.17	1.09±0.24	1.03±0.24
C2_19700	Clathrin interactor 1	CLINT1	0.90±0.22	0.99±0.14	0.83±0.12	0.85±0.04
C2_1094	Cleavage and polyadenylation specificity factor subunit 5	NUDT21	1.05±0.22	1.62±0.43	1.82±0.69	1.07±0.29
C2_76527	Coactosin-like protein	COTL1	1.30±0.14	1.55±0.41	1.80±0.48	1.20±0.21
C2_5592	Coatomer subunit alpha	COPA	0.97±0.16	1.46±0.18	1.18±0.11	1.10±0.25
C2_66184	Coatomer subunit alpha	COPA	0.69±0.20	0.81±0.15	1.10±0.25	1.02±0.23
C2_4111	Coatomer subunit beta	COPB1	0.83±0.07	1.19±0.12	1.64±0.35	1.03±0.19
C2_27574	Coatomer subunit beta	COPB1	0.81±0.10	1.00±0.11	1.03±0.17	0.91±0.17
C2_6399	Coatomer subunit beta'	COPB2	1.07±0.04	1.09±0.10	0.99±0.09	1.11±0.12
C2_5687	Coatomer subunit delta	ARCN1	0.89±0.08	0.98±0.17	0.85±0.15	1.13±0.17
C2_191	Coatomer subunit epsilon	COPE	0.78±0.11	1.19±0.18	1.20±0.08	1.04±0.26
C2_3555	Coatomer subunit gamma-2	COPG2	1.08±0.13	1.03±0.08	1.38±0.19	1.10±0.12
C2_120873	Cocaine esterase	COCE	1.51±0.29	1.38±0.13	0.92±0.22	1.40±0.26
C2_1192	Cofilin-2	CFL2	0.75±0.11	1.06±0.15	2.01±0.74	2.12±0.78
C2_7703	Coiled-coil domain-containing protein 22	CCDC22	1.50±1.26	1.24±0.89	1.77±1.14	1.54±0.72
C2_36566	Cold-inducible RNA-binding protein A	CIRBP	0.71±0.22	0.68±0.11	0.93±0.30	1.03±0.41
C2_6735	Collagen type IV alpha-3-binding protein	COL4A3BP	1.08±0.20	0.90±0.10	1.06±0.21	1.27±0.17
C2_15503	Collectin-12	COLEC12	2.01±0.32	3.85±1.11	1.93±0.25	1.91±0.38
C2_481	COMM domain-containing protein 2	COMMD2	1.50±0.64	2.14±0.92	3.82±1.84	3.85±1.50
C2_1725	COMM domain-containing protein 7	COMMD7	1.23±0.15	1.54±0.35	1.12±0.19	1.81±0.54
C2_2765	Complement C1q tumor necrosis factor-related protein 3	C1QTNF3	1.60±0.45	5.42±2.13	1.65±0.76	0.95±0.18
C2_107825	Complement C1q-like protein 2	C1QL2	2.31±0.33	0.97±0.20	1.65±0.84	1.31±0.39
C2_1398	Complement C3	C3	1.06±0.11	2.29±0.55	0.93±0.12	0.85±0.12
C2_23904	Complement C4-B	C4A/C4B	1.11±0.10	1.14±0.14	0.96±0.17	0.78±0.14
C2_2093	Complement component C6	C6	0.69±0.06	1.59±0.44	1.70±0.45	0.83±0.08
C2_9760	Complement factor B	CFB	0.84±0.12	1.97±0.50	0.95±0.14	0.91±0.10
C2_121665	Complement factor D	CFD	1.19±0.29	1.36±0.32	1.17±0.22	1.16±0.23
C2_2374	Complement factor H	CFH	0.91±0.16	1.04±0.16	0.79±0.11	0.80±0.11
C2_1203	COP9 signalosome complex subunit 5	COPS5	0.81±0.22	1.20±0.22	1.33±0.28	0.60±0.14
C2_6548	Copine-1	CPNE1	1.18±0.16	0.83±0.15	1.37±0.23	1.07±0.33
C2_2684	Copine-3	CPNE3	0.88±0.15	0.83±0.15	1.07±0.19	1.03±0.18
C2_3222	Copper homeostasis protein cutC homolog	CUTC	1.26±0.20	1.32±0.06	1.15±0.06	1.05±0.14
C2_113919	Copper transport protein ATOX1	ATOX1	1.05±0.18	1.24±0.25	2.53±0.85	1.61±0.34
C2_2051	Coproporphyrinogen-III oxidase, mitochondrial	CPOX	1.64±0.25	1.01±0.16	1.01±0.16	1.41±0.41
C2_451	Coronin-1A	CORO1A	1.26±0.17	1.00±0.10	0.93±0.09	1.19±0.15
C2_690	Creatine kinase B-type	CKB	1.13±0.25	1.68±0.28	1.33±0.19	1.19±0.27
C2_24223	Creatine kinase U-type, mitochondrial	CKMT1A	2.00±0.39	1.06±0.19	1.11±0.29	1.87±0.44
C2_29254	Crk-like protein	CRKL	1.48±0.20	1.01±0.12	0.98±0.24	1.15±0.26
C2_1078	Crystallin J1C	CJ1C	1.03±0.12	1.04±0.14	1.24±0.22	1.28±0.22
C2_1820	Cystathionine beta-synthase	CBS	1.36±0.31	1.91±0.44	1.73±0.34	1.37±0.32
C2_2620	Cystathionine gamma-lyase	CTH	1.18±0.13	2.02±0.23	1.71±0.26	1.45±0.33
C2_617	Cystatin-B	CSTB	1.19±0.13	1.25±0.17	1.31±0.23	1.21±0.09
C2_9620	Cysteine sulfinic acid decarboxylase	CSAD	0.84±0.21	1.34±0.31	0.95±0.05	0.87±0.12
C2_42628	Cysteine-rich protein 1	CRIP1	0.81±0.21	1.43±0.40	1.35±0.25	1.79±0.44

C2_4479	Cysteinyl-tRNA synthetase, cytoplasmic	CARS	0.84±0.11	1.21±0.16	0.84±0.12	0.95±0.12
C2_12715	Cytochrome b5	CYB5A	1.55±0.23	1.08±0.19	1.19±0.22	1.27±0.22
C2_2189	Cytochrome c	LOC690675	1.41±0.31	1.20±0.24	1.28±0.33	1.62±0.45
C2_785	Cytochrome c1, heme protein, mitochondrial	CYC1	32.67±19.93	10.75±9.69	7.98±6.81	28.71±17.47
C2_46314	Cytochrome c-b	CYCB	1.59±0.36	1.13±0.20	1.09±0.29	1.54±0.39
C2_13986	Cytochrome P450 2D26	Cyp2d26	1.27±0.09	1.02±0.15	1.04±0.07	1.22±0.14
C2_4087	Cytochrome P450 2J2	CYP2J2	0.77±0.08	0.95±0.25	1.02±0.26	0.98±0.17
C2_57426	Cytochrome P450 2K3	CYP2K3	1.29±0.18	2.14±0.60	1.57±0.47	1.86±0.61
C2_2493	Cytochrome P450 3A40	CYP3A40	0.70±0.12	0.80±0.11	0.81±0.20	0.93±0.19
C2_2851	Cytoplasmic aconitate hydratase	ACO1	1.18±0.10	1.04±0.10	0.87±0.11	0.87±0.08
C2_68872	Cytoplasmic aconitate hydratase	ACO1	0.86±0.09	1.16±0.11	0.92±0.09	0.97±0.11
s_flp0006b09_f_1	Cytoplasmic aconitate hydratase	ACO1	0.99±0.08	1.45±0.23	2.21±0.69	1.89±0.50
C2_16351	Cytoplasmic dynein 1 heavy chain 1	DYNC1H1	0.75±0.08	0.91±0.07	1.16±0.18	0.83±0.17
C2_39955	Cytoplasmic dynein 1 heavy chain 1	DYNC1H1	0.64±0.12	0.57±0.03	0.93±0.27	0.91±0.13
C2_5034	Cytoplasmic dynein 1 heavy chain 1	DYNC1H1	0.71±0.08	0.78±0.06	0.98±0.20	0.86±0.09
C2_82382	Cytoplasmic dynein 1 heavy chain 1	DYNC1H1	1.18±0.15	0.75±0.20	0.77±0.29	1.09±0.27
C2_7060	Cytoplasmic dynein 1 intermediate chain 2	Dync1i2	0.97±0.08	1.01±0.13	1.13±0.27	1.57±0.28
C2_928	Cytosolic non-specific dipeptidase	CNDP2	0.86±0.08	1.09±0.20	1.24±0.24	1.13±0.18
C2_5757	Cytosolic purine 5'-nucleotidase	NT5C2	1.60±0.36	1.43±0.23	0.98±0.14	1.10±0.21
C2_70910	Cytosolic sulfotransferase 1	SOT1	0.76±0.13	0.76±0.11	0.98±0.17	1.16±0.30
C2_2270	Cytosolic sulfotransferase 3	SOT3	0.69±0.06	0.80±0.13	1.18±0.29	1.16±0.32
C2_7372	D-amino-acid oxidase	DAO	0.76±0.09	1.04±0.22	1.26±0.21	0.92±0.20
C2_1763	D-dopachrome decarboxylase	DDT	1.82±0.28	1.26±0.18	0.86±0.12	1.17±0.24
C2_3999	Dehydrogenase/reductase SDR family member 1	DHRS1	0.99±0.08	0.85±0.13	0.97±0.07	1.09±0.17
C2_34491	Dehydrogenase/reductase SDR family member 11	DHRS11	2.02±0.36	1.26±0.16	1.28±0.12	1.47±0.16
C2_35778	Dehydrogenase/reductase SDR family member 12	DHRS12	1.07±0.14	1.22±0.11	0.80±0.13	1.10±0.07
C2_43533	Dehydrogenase/reductase SDR family member 7B	DHRS7B	1.95±0.43	1.31±0.40	1.31±0.21	1.64±0.43
C2_9536	Deleted in malignant brain tumors 1 protein	DMBT1	3.78±1.85	0.75±0.29	0.68±0.21	1.60±0.95
C2_12746	Delta(24)-sterol reductase	DHCR24	1.28±0.35	1.00±0.19	2.16±0.52	1.68±0.28
C2_17169	Delta-aminolevulinic acid dehydratase	ALAD	1.49±0.28	1.23±0.19	0.87±0.16	1.01±0.20
C2_94566	Deoxyribonuclease-1	DNASE1	2.29±0.39	0.65±0.12	0.67±0.26	1.11±0.42
C2_20476	Desmoplakin	DSP	1.05±0.17	1.12±0.19	0.87±0.10	0.96±0.20
C2_521	Diablo homolog, mitochondrial	DIABLO	17.66±16.47	32.38±19.76	27.74±17.65	17.98±16.41
C2_83822	Diacylglycerol kinase alpha	DGKA	0.92±0.45	1.65±0.23	1.33±0.30	0.99±0.37
C2_5593	Dihydrolipoyl dehydrogenase, mitochondrial	DLD	1.69±0.40	1.41±0.33	1.41±0.37	1.68±0.39
C2_9224	Dimethylaniline monooxygenase [N-oxide-forming] 5	FMO5	0.94±0.20	0.74±0.23	0.90±0.30	1.24±0.25
C2_192	Dipeptidase 1	DPEP1	2.06±0.44	1.47±0.36	0.84±0.21	0.96±0.25
C2_14794	Dipeptidyl peptidase 3	DPP3	1.22±0.20	0.96±0.14	0.70±0.11	1.24±0.32
C2_40681	Dipeptidyl peptidase 3	DPP3	1.37±0.24	1.12±0.20	0.98±0.26	1.55±0.45
C2_46432	Dipeptidyl peptidase 3	DPP3	1.43±0.15	1.18±0.17	1.12±0.33	1.34±0.29
C2_1242	Dipeptidyl peptidase 4	DPP4	2.36±0.34	1.10±0.17	0.90±0.22	1.33±0.42
C2_7518	Diphosphomevalonate decarboxylase	MVD	0.86±0.10	1.22±0.18	1.20±0.09	1.08±0.17
C2_4400	Disabled homolog 2	DAB2	0.96±0.16	1.17±0.13	1.48±0.45	1.78±0.61
C2_15432	DNA damage-binding protein 1	DDB1	1.04±0.15	1.16±0.14	0.94±0.14	0.81±0.12
C2_2056	DNA-(apurinic or apyrimidinic site) lyase	APEX1	1.86±0.51	2.32±0.31	1.50±0.11	1.81±0.49
C2_1240	Dolichyl-diphosphooligosaccharide--protein glycosyltransferase 48 kDa subunit	DDOST	0.83±0.11	0.73±0.13	1.03±0.21	0.96±0.17
C2_37060	Dolichyl-diphosphooligosaccharide--protein glycosyltransferase subunit 1	RPN1	0.95±0.22	1.08±0.14	1.70±0.38	1.25±0.32
C2_3023	Dolichyl-diphosphooligosaccharide--protein glycosyltransferase subunit 1	RPN1	1.13±0.20	0.85±0.15	1.17±0.21	1.28±0.28
C2_1591	Dolichyl-diphosphooligosaccharide--protein glycosyltransferase subunit 2	RPN2	1.01±0.10	1.01±0.14	1.42±0.22	1.22±0.36
C2_1500	Dolichyl-diphosphooligosaccharide--protein glycosyltransferase subunit STT3A	STT3A	1.13±0.09	1.02±0.19	1.56±0.45	1.13±0.29
C2_8403	Drebrin-like protein B	DBN1	1.01±0.19	1.16±0.30	2.12±1.04	0.93±0.06
C2_10485	D-serine dehydratase	SRR	1.16±0.20	1.21±0.13	1.01±0.16	0.95±0.09
C2_6083	D-serine dehydratase	SRR	1.54±0.40	1.72±0.17	1.55±0.14	1.56±0.23
C2_1808	Dual specificity protein phosphatase 23	DUSP23	1.12±0.26	1.36±0.44	1.18±0.35	1.14±0.25
C2_10012	Dynactin subunit 2	DCTN2	0.79±0.17	0.67±0.11	0.97±0.27	1.17±0.25
C2_64914	Dynactin subunit 3	DCTN3	0.59±0.09	0.74±0.24	1.93±0.92	2.01±0.95
C2_3772	Dynamin-1-like protein	DNM1L	0.87±0.18	1.02±0.20	2.05±0.44	1.20±0.48
C2_6275	Dynamin-2	DNM2	0.89±0.11	0.79±0.08	1.29±0.44	1.45±0.38
C2_27580	Dynein light chain 2, cytoplasmic	DYNLL2	0.84±0.11	1.32±0.34	1.12±0.10	1.19±0.31
C2_1338	E3 ubiquitin-protein ligase HUWE1	HUWE1	0.87±0.09	1.26±0.08	0.98±0.06	0.96±0.15
C2_73398	E3 ubiquitin-protein ligase HUWE1	HUWE1	0.61±0.17	1.20±0.14	1.67±0.48	1.05±0.32
C2_4136	Echinoderm microtubule-associated protein-like 1	EML1	0.82±0.08	1.16±0.21	0.84±0.06	1.03±0.12
C2_29850	Ectonucleotide pyrophosphatase/phosphodiesterase family member 3	ENPP3	2.48±0.47	1.02±0.27	0.62±0.16	1.23±0.47
C2_53228	Ectonucleotide pyrophosphatase/phosphodiesterase family member 3	ENPP3	1.83±0.19	1.04±0.13	0.89±0.23	1.14±0.34
C2_2589	Ectonucleotide pyrophosphatase/phosphodiesterase family member 6	ENPP6	1.87±0.21	1.08±0.17	0.81±0.16	0.88±0.14
C2_8279	Ectonucleotide pyrophosphatase/phosphodiesterase family member 7	ENPP7	1.54±0.13	1.16±0.27	1.23±0.24	1.35±0.29
C2_8820	EF-hand domain-containing protein D2	EFHD2	1.03±0.08	1.27±0.18	1.30±0.21	0.78±0.16
C2_25319	EH domain-containing protein 1	EHD1	0.83±0.04	0.97±0.10	1.23±0.26	1.18±0.21
C2_4289	Elastase-1	CELA1	3.18±0.62	0.60±0.06	1.06±0.62	1.84±0.70
C2_18	Elongation factor 1-alpha	EEF1A	0.91±0.09	0.84±0.09	1.59±0.46	1.88±0.69

C2_344	Elongation factor 1-beta	EEF1B2	0.61±0.09	0.81±0.21	1.21±0.50	1.10±0.30
C2_25907	Elongation factor 1-delta	EEF1D	1.19±0.17	1.15±0.16	1.12±0.10	1.39±0.19
C2_55	Elongation factor 1-gamma	EEF1G	0.83±0.08	1.05±0.18	0.97±0.16	1.20±0.17
C2_534	Elongation factor 2	EEF2	0.73±0.06	0.96±0.18	1.45±0.35	1.19±0.27
C2_11409	Endoplasmic reticulum aminopeptidase 1	ERAP1	0.68±0.10	0.77±0.21	0.90±0.22	0.92±0.23
C2_21801	Endoplasmic reticulum resident protein 44	ERP44	1.08±0.23	1.36±0.25	2.04±0.44	1.41±0.41
C2_13119	Enolase	ENO	0.83±0.23	1.47±0.17	0.97±0.22	0.75±0.16
C2_3722	Enoyl-CoA hydratase, mitochondrial	ECHS1	0.95±0.13	0.57±0.12	0.78±0.21	1.12±0.29
C2_646	Eosinophil peroxidase	EPX	2.16±1.04	0.77±0.17	0.74±0.21	0.58±0.14
C2_118	Ependymin	EPD	3.10±0.68	2.07±0.28	1.99±0.67	1.86±0.46
C2_117429	Ependymin-2	EPD2	5.14±1.28	2.90±0.38	5.13±2.27	3.41±0.88
C2_14283	Epidermal growth factor receptor kinase substrate 8-like protein 2	EPS8L2	1.05±0.18	1.01±0.19	1.21±0.25	1.05±0.21
C2_5498	Epidermal growth factor receptor kinase substrate 8-like protein 3	EPS8L3	0.69±0.07	1.24±0.29	0.99±0.08	1.05±0.18
C2_782	Epididymal secretory protein E1	NPC2	3.76±1.13	3.46±0.64	3.31±1.27	2.17±0.58
C2_6684	Epoxide hydrolase 1	EPHX1	1.23±0.22	0.90±0.37	1.36±0.34	1.28±0.49
C2_22855	Epoxide hydrolase 2	EPHX2	1.31±0.13	1.65±0.33	1.92±0.43	2.18±0.35
C2_1732	Erythrocyte band 7 integral membrane protein	STOM	1.51±0.22	1.17±0.07	1.19±0.22	1.55±0.24
C2_800	ES1 protein homolog, mitochondrial	C21orf33	1.39±0.18	1.23±0.10	0.88±0.10	1.18±0.22
C2_4860	Ester hydrolase C11orf54 homolog	C11orf54	1.65±0.36	1.43±0.17	1.04±0.06	1.30±0.18
C2_12674	Estradiol 17-beta-dehydrogenase 12-B	HSD17B12	1.27±0.15	1.37±0.34	1.45±0.20	1.50±0.19
C2_3321	Ethanolamine-phosphate cytidylyltransferase	PCYT2	1.12±0.19	1.44±0.24	1.51±0.52	1.46±0.26
C2_406	Eukaryotic initiation factor 4A-I	EIF4A1	0.73±0.15	0.85±0.19	1.05±0.30	1.19±0.31
C2_746	Eukaryotic initiation factor 4A-II	EIF4A2	1.00±0.15	1.10±0.13	0.80±0.09	0.90±0.16
C2_4011	Eukaryotic translation initiation factor 2 subunit 1	EIF2S1	0.86±0.15	1.56±0.34	1.29±0.13	1.12±0.27
C2_533	Eukaryotic translation initiation factor 3 subunit A	EIF3A	0.59±0.05	1.17±0.28	0.78±0.09	0.89±0.10
C2_630	Eukaryotic translation initiation factor 3 subunit B	EIF3B	1.22±0.19	1.29±0.14	0.92±0.06	0.97±0.12
C2_541	Eukaryotic translation initiation factor 3 subunit D	EIF3D	0.94±0.13	1.05±0.10	1.02±0.05	0.97±0.16
C2_114	Eukaryotic translation initiation factor 3 subunit E	EIF3E	2.20±1.19	1.42±0.45	1.28±0.29	1.63±0.26
C2_92	Eukaryotic translation initiation factor 3 subunit F	EIF3F	0.94±0.12	0.91±0.06	1.03±0.18	1.00±0.12
C2_19263	Eukaryotic translation initiation factor 3 subunit J-A	EIF3J	0.86±0.23	1.03±0.13	0.93±0.18	0.88±0.14
C2_372	Eukaryotic translation initiation factor 3 subunit L	EIF3L	0.81±0.13	1.43±0.21	1.23±0.29	1.04±0.19
C2_68	Eukaryotic translation initiation factor 3 subunit M	EIF3M	0.44±0.13	1.39±0.19	1.33±0.30	0.76±0.19
C2_2506	Eukaryotic translation initiation factor 4 gamma 1	EIF4G1	1.02±0.08	0.89±0.20	0.65±0.15	0.96±0.10
C2_6179	Eukaryotic translation initiation factor 5	EIF5	1.03±0.13	1.26±0.22	1.65±0.68	1.65±0.34
C2_6319	Eukaryotic translation initiation factor 6	EIF6	1.17±0.16	0.92±0.12	0.82±0.13	0.85±0.13
C2_54734	Exportin-1	XPO1	0.63±0.34	2.12±0.62	1.34±0.31	0.97±0.28
C2_10706	Ezrin	EZR	0.90±0.12	0.64±0.05	1.12±0.26	1.53±0.48
C2_24358	Ezrin	EZR	0.65±0.07	0.90±0.14	1.07±0.19	0.99±0.19
C2_356	F-actin-capping protein subunit alpha-1	CAPZA1	1.21±0.18	1.07±0.20	1.18±0.18	1.35±0.29
C2_207	F-actin-capping protein subunit alpha-2	CAPZA2	0.91±0.17	1.80±0.25	1.34±0.09	1.26±0.12
C2_227	F-actin-capping protein subunit beta	CAPZB	0.79±0.06	1.48±0.17	1.09±0.07	1.03±0.13
C2_10323	Farnesyl pyrophosphate synthase	FDPS	1.27±0.47	1.92±0.73	2.35±1.05	2.32±0.72
C2_7862	FAS-associated factor 1	FAF1	1.14±0.16	1.22±0.12	1.06±0.12	0.90±0.24
C2_8342	Fatty acid-binding protein, brain	FABP7	1.78±0.49	2.27±0.41	1.39±0.16	1.80±0.56
C2_168	Fatty acid-binding protein, heart	FABP3	0.64±0.06	1.29±0.37	1.07±0.17	1.27±0.53
C2_28163	Fatty acid-binding protein, intestinal	FABP2	0.41±0.06	1.51±0.52	1.55±0.46	1.80±0.60
C2_51889	Fatty acid-binding protein, intestinal	FABP2	0.57±0.06	2.55±0.96	2.62±0.91	3.14±1.21
C2_23355	Fatty acid-binding protein, liver-type	FABP1	2.04±0.33	2.09±0.32	1.44±0.26	1.59±0.26
C2_5343	Fatty aldehyde dehydrogenase	ALDH3A2	1.99±0.45	1.19±0.41	1.81±0.49	2.04±0.67
C2_10150	F-box only protein 22	FBXO22	1.30±0.48	2.12±0.37	2.76±1.04	0.99±0.24
C2_5555	FCH domain only protein 2	FCHO2	1.04±0.11	1.35±0.12	1.31±0.15	1.15±0.15
C2_37659	Female protein	WTAP	1.10±0.13	0.74±0.06	0.80±0.17	1.36±0.49
FP331537	Fibrinogen alpha chain	FGA	1.40±0.23	1.27±0.20	0.90±0.16	0.95±0.28
FP339542	Fibrinogen beta chain	FGB	1.57±0.22	1.05±0.14	0.77±0.17	0.88±0.22
FP332283	Fibrinogen beta chain	FGB	1.75±0.31	1.51±0.28	1.08±0.13	1.10±0.24
C2_38689	Fibrinogen gamma chain	FGG	1.31±0.27	1.39±0.32	0.78±0.13	0.96±0.15
C2_58470	Fibronectin	FN1	1.21±0.10	0.95±0.07	0.89±0.16	0.98±0.21
C2_5254	Fibronectin	FN1	0.84±0.09	1.31±0.29	1.15±0.39	0.91±0.21
C2_63263	Fibronectin	FN1	1.16±0.22	1.29±0.29	1.22±0.55	0.96±0.22
C2_6274	Filamin-A	FLNA	1.80±0.26	1.02±0.10	1.00±0.25	1.00±0.19
C2_79807	Filamin-B	FLNB	1.84±0.27	1.52±0.27	1.19±0.23	1.48±0.19
C2_65152	Filamin-C	FLNC	1.35±0.70	1.65±0.85	2.86±0.96	1.66±0.59
C2_6583	FK506-binding protein 1	FK506-BP	0.82±0.04	1.40±0.28	1.26±0.24	1.34±0.28
C2_1227	Flavin reductase (NADPH)	BLVRB	1.05±0.18	1.52±0.58	1.32±0.31	1.14±0.19
C2_6545	Fructose-1,6-bisphosphatase 1	FBP1	0.61±0.08	1.79±0.26	1.33±0.14	1.11±0.28
C2_185	Fructose-bisphosphate aldolase B	ALDOB	0.89±0.09	1.98±0.50	2.89±0.83	2.53±0.85
C2_1285	Fructose-bisphosphate aldolase C-B	ALDOC	0.98±0.15	1.04±0.12	0.94±0.14	0.87±0.09
C2_23658	Fructose-bisphosphate aldolase, muscle type	FREM2	1.00±0.27	1.24±0.21	1.51±0.53	0.90±0.22
C2_32246	Fucolectin	FCL	2.14±0.47	1.60±0.47	0.80±0.11	0.92±0.28
C2_116478	Fucolectin-5	FCL5	2.34±0.56	0.92±0.09	1.71±1.05	0.80±0.18
C2_24896	Fumarylacetoacetate hydrolase domain-containing protein 1	FAHD1	1.80±0.36	0.96±0.17	0.94±0.12	1.38±0.35
C2_1568	Fumarylacetoacetate hydrolase domain-containing protein 2	FAHD2B	1.51±0.14	1.15±0.11	0.94±0.13	1.15±0.16
C2_2035	Galactocerebrosidase	GALC	1.45±0.19	0.92±0.13	0.86±0.12	1.36±0.32
C2_28569	Galectin-3	LGALS3	0.75±0.12	0.86±0.16	0.68±0.11	0.68±0.09
C2_985	Galectin-9	LGALS9B	1.75±0.31	1.10±0.12	1.05±0.12	1.15±0.19
C2_5451	Gamma-butyrobetaine dioxygenase	BBOX1	1.67±0.20	1.18±0.24	1.32±0.48	1.06±0.22
AM954422	Gamma-glutamylaminecyclotransferase A	GGACT	1.38±0.22	1.47±0.18	0.90±0.04	0.89±0.14

C2_15180	Gamma-glutamylcyclotransferase	GGCT	1.14±0.25	1.39±0.25	1.08±0.17	1.04±0.25
C2_1354	Gamma-glutamyltranspeptidase 1	GGT1	1.62±0.36	0.89±0.10	0.70±0.13	0.91±0.24
C2_63	Gamma-interferon-inducible lysosomal thiol reductase	IFI30	2.26±0.35	1.00±0.08	0.80±0.29	1.29±0.40
C2_34628	Gastric intrinsic factor	GIF	1.81±0.29	1.30±0.30	1.06±0.56	0.75±0.18
C2_750	Gastrotropin	FABP6	0.91±0.14	1.05±0.06	0.90±0.19	1.06±0.21
C2_14681	GDH/6PGL endoplasmic bifunctional protein	H6PD	1.41±0.25	1.01±0.16	1.67±0.37	0.98±0.16
C2_265	Gelsolin	GSN	1.04±0.16	1.74±0.15	1.28±0.17	1.04±0.20
C2_768	Glucosamine-6-phosphate isomerase 1	GNPDA1	0.87±0.07	1.21±0.31	1.10±0.13	1.10±0.20
C2_20259	Glucosamine--fructose-6-phosphate aminotransferase [isomerizing] 2	GNPDA2	0.96±0.24	0.78±0.09	1.59±0.57	0.98±0.13
C2_5943	Glucose-6-phosphate 1-dehydrogenase	G6PD	0.65±0.06	0.91±0.12	1.01±0.22	0.91±0.21
C2_394	Glucose-6-phosphate isomerase	GPI	2.13±0.32	1.25±0.28	1.19±0.33	1.45±0.35
C2_1745	Glucosylceramidase	GBA	2.08±0.31	1.06±0.23	1.03±0.29	1.36±0.38
C2_10880	Glucuronokinase 1	GLCAK1	0.78±0.12	0.91±0.14	0.79±0.08	1.21±0.15
C2_3924	Glutamate dehydrogenase, mitochondrial	GLUD1	0.74±0.07	1.45±0.25	1.53±0.50	0.95±0.18
C2_2794	Glutamate--cysteine ligase catalytic subunit	GCLC	1.13±0.07	0.94±0.11	1.17±0.16	1.20±0.11
C2_944	Glutamine synthetase	GLUL	1.02±0.14	0.84±0.12	0.87±0.22	1.01±0.16
C2_4118	Glutamyl aminopeptidase	ENPEP	1.90±0.36	1.43±0.36	0.97±0.19	1.07±0.35
C2_6320	Glutaredoxin 3	GLRX3	0.77±0.10	0.99±0.19	0.85±0.14	0.96±0.15
C2_345	Glutathione peroxidase 1	GPX1	1.93±0.46	1.02±0.24	0.70±0.22	1.47±0.51
C2_296	Glutathione peroxidase 2	GPX2	2.08±0.30	0.89±0.15	0.92±0.21	1.30±0.33
C2_19719	Glutathione reductase, mitochondrial	GSR	1.73±0.25	1.04±0.13	1.19±0.40	1.29±0.34
C2_5598	Glutathione S-transferase 3	GSTA3	1.12±0.37	1.48±0.15	0.92±0.21	0.90±0.23
C2_117130	Glutathione S-transferase A	GSTA1	1.13±0.28	2.81±0.83	1.73±0.37	1.48±0.41
C2_112663	Glutathione S-transferase A4	GSTA4	0.90±0.48	2.08±0.32	1.30±0.20	1.32±0.31
C2_430	Glutathione S-transferase kappa 1	GSTK1	2.28±0.49	1.00±0.12	1.02±0.18	1.20±0.26
C2_868	Glutathione S-transferase Mu 3	Gstm3	0.83±0.22	1.62±0.17	1.15±0.11	0.92±0.26
C2_1065	Glutathione S-transferase omega-1	GSTO1	1.03±0.11	1.40±0.15	0.94±0.13	1.12±0.12
C2_1660	Glutathione S-transferase theta-1	Gstt1	0.88±0.14	1.12±0.36	1.03±0.23	0.91±0.20
C2_5198	Glutathione S-transferase theta-4	Gstt4	0.60±0.09	1.39±0.56	1.62±0.71	1.90±0.74
C2_25988	Glutathione synthetase	GSS	1.44±0.24	1.22±0.21	1.04±0.26	1.12±0.29
C2_29	Glyceraldehyde 3-phosphate dehydrogenase, testis-specific	GAPDHS	2.31±0.52	1.89±0.09	1.29±0.15	1.66±0.34
C2_17	Glyceraldehyde-3-phosphate dehydrogenase	GAPDH	2.67±0.56	1.52±0.23	1.58±0.07	2.71±0.35
C2_688	Glycerol-3-phosphate dehydrogenase [NAD+], cytoplasmic	GPD1	1.86±0.79	2.21±1.31	3.01±2.19	3.60±2.54
C2_2813	Glycerol-3-phosphate dehydrogenase 1-like protein	GPD1L	0.80±0.15	0.94±0.17	1.37±0.44	1.34±0.48
C2_9479	Glycine cleavage system H protein, mitochondrial	GCSH	2.54±0.53	1.59±0.22	1.92±0.72	1.89±0.70
C2_27493	Glycine N-methyltransferase	GNMT	0.58±0.11	0.76±0.26	1.08±0.44	0.97±0.28
C2_15427	Glycogen debranching enzyme	AGL	0.72±0.36	1.10±0.15	0.92±0.26	0.67±0.20
C2_1350	Glycolipid transfer protein	GLTP	0.60±0.09	0.91±0.10	0.88±0.12	0.70±0.15
C2_637	Glycylpeptide N-tetradecanoyltransferase 1	NMT1	0.86±0.14	1.26±0.24	1.36±0.27	1.06±0.20
C2_1726	Glycyl-tRNA synthetase	GLYS	0.88±0.07	1.11±0.08	1.18±0.25	0.97±0.21
C2_2114	Glyoxalase domain-containing protein 4	GLOD4	0.82±0.10	1.36±0.17	1.02±0.17	1.25±0.22
C2_2680	Glyoxalase domain-containing protein 5	GLOD5	1.90±0.60	0.80±0.10	1.26±0.33	0.62±0.16
C2_1994	Glyoxylate reductase/hydroxypyruvate reductase	GRHPR	1.57±0.16	1.07±0.15	1.04±0.11	1.52±0.28
C2_3971	Grancalcin	GCA	0.91±0.03	1.47±0.35	1.78±0.35	1.49±0.59
C2_4717	Growth factor receptor-bound protein 2	GRB2	0.87±0.10	0.98±0.10	0.90±0.09	0.99±0.05
C2_26978	GTPase IMAP family member 2	GIMAP2	2.51±0.65	2.27±0.68	1.79±0.24	2.16±0.65
C2_1141	GTP-binding nuclear protein Ran	RAN	0.71±0.10	1.03±0.15	1.35±0.23	1.04±0.25
C2_1272	GTP-binding protein SAR1b	SAR1B	0.58±0.09	0.70±0.15	1.19±0.44	1.27±0.47
C2_762	Guanidinoacetate N-methyltransferase	GAMT	0.71±0.05	1.79±0.21	1.37±0.32	1.22±0.28
C2_829	Guanine nucleotide-binding protein G(i) subunit alpha-2	GNAI2	0.77±0.10	1.15±0.19	1.05±0.23	1.12±0.24
C2_3700	Guanine nucleotide-binding protein G(o) subunit alpha	GNAO1	1.25±0.36	1.96±0.98	4.38±1.74	1.76±0.57
C2_17403	Guanine nucleotide-binding protein G(q) subunit alpha	GNAQ	1.09±0.25	0.76±0.14	0.62±0.11	0.95±0.31
C2_45	Guanine nucleotide-binding protein subunit beta-2-like 1	GNB2L1	0.55±0.09	0.72±0.05	1.03±0.19	0.71±0.11
C2_5901	Harmonin	USH1C	0.90±0.08	1.02±0.18	0.96±0.19	1.10±0.11
C2_13612	Heat shock 70 kDa protein 4	HSPA4	0.70±0.28	1.16±0.33	1.57±0.34	1.07±0.38
C2_58	Heat shock cognate 70 kDa protein	Hspa1b	0.79±0.04	1.58±0.17	1.61±0.35	1.25±0.17
C2_2116	Heat shock protein 105 kDa	HSPH1	0.77±0.06	0.89±0.11	1.43±0.28	1.07±0.25
C2_7127	Hemoglobin subunit alpha-A	HBAA	1.68±0.53	1.57±0.40	0.68±0.17	0.89±0.28
C2_5435	Hemoglobin subunit alpha-B	HBAB	1.64±0.57	2.07±0.66	0.78±0.24	0.88±0.31
C2_105738	Hemoglobin subunit beta	HBB	1.32±0.40	1.65±0.32	0.82±0.15	0.78±0.17
C2_102093	Hemoglobin subunit beta	HBB	1.69±0.51	1.78±0.53	0.90±0.21	1.17±0.44
C2_78448	Hemoglobin subunit beta-A	Hbb-b2	1.54±0.51	1.32±0.31	0.74±0.29	1.08±0.52
C2_27397	Hemopexin	HPX	1.11±0.14	1.75±0.39	0.93±0.19	1.11±0.37
C2_1739	Hepatoma-derived growth factor	HDGF	0.77±0.12	1.09±0.26	0.80±0.20	0.78±0.12
C2_6512	Heterogeneous nuclear ribonucleoprotein A/B	HNRNPAB	0.85±0.08	1.15±0.41	1.31±0.46	1.41±0.38
C2_24145	Heterogeneous nuclear ribonucleoprotein A1	Hnrnpa1	1.05±0.22	1.77±0.31	1.19±0.30	1.36±0.18
C2_26519	Heterogeneous nuclear ribonucleoprotein Q	SYNCRIP	0.85±0.10	1.23±0.07	0.99±0.11	0.96±0.11
C2_1517	Heterogeneous nuclear ribonucleoprotein U-like protein 1	HNRNPUL1	0.97±0.10	1.31±0.15	1.02±0.12	1.44±0.19
C2_75390	Hexokinase-1	HK1	1.50±0.18	1.51±0.82	1.89±0.63	1.46±0.47
C2_8165	Hibernation-specific plasma protein HP-55	HP55	1.49±0.23	1.53±0.24	0.95±0.25	1.12±0.21
C2_11749	High choriolytic enzyme 1	HCEA	3.01±0.40	0.65±0.08	0.90±0.41	1.70±0.59
C2_1967	Histamine N-methyltransferase A	HNMT	0.48±0.06	1.36±0.56	0.69±0.13	0.74±0.17
C2_9935	Histidyl-tRNA synthetase, cytoplasmic	HARS2	0.86±0.11	1.27±0.12	1.31±0.09	0.84±0.16
C2_65292	Histidyl-tRNA synthetase, cytoplasmic	HARS2	0.93±0.18	1.09±0.10	0.70±0.10	0.65±0.06
C2_29246	Histone deacetylase 9-B	HDAC9B	1.26±0.46	16.35±3.14	7.86±2.40	6.91±5.53
C2_3677	Histone H1	H1F0	1.15±0.19	0.59±0.07	0.96±0.13	1.63±0.29
C2_12456	Histone H2A	H2AFB3	1.07±0.13	1.13±0.19	3.24±1.00	2.49±1.14
C2_101057	Histone H2B 1/2	HIST2H2BE	1.06±0.26	1.86±0.55	1.68±0.73	1.85±0.82

C2_117463	Histone H4	HIST1H4A	0.82±0.04	1.05±0.15	1.90±0.82	1.51±0.38
C2_3745	Histone-binding protein RBBP4	RBBP4	0.73±0.08	0.90±0.12	0.97±0.08	0.91±0.09
C2_25525	Host cell factor 1	HCFC1	2.28±0.28	1.59±0.42	0.76±0.13	1.14±0.27
C2_16741	Host cell factor 1	HCFC1	1.34±0.15	0.99±0.19	0.79±0.16	0.81±0.21
C2_17788	Huntingtin	HTT	6.60±5.68	2.19±1.25	2.09±0.98	1.86±0.74
C2_23228	Hyaluronidase-1	HYAL1	2.42±0.44	0.92±0.12	0.85±0.27	1.45±0.41
C2_465	Hydroxyacyl-coenzyme A dehydrogenase, mitochondrial	HADH	2.19±0.46	1.03±0.17	1.27±0.29	1.50±0.35
C2_610	Hydroxyacylglutathione hydrolase, mitochondrial	HAGH	0.89±0.16	1.11±0.14	0.88±0.09	0.67±0.11
C3_c25627	Hypothetical protein LOC100692391	GIMAP8	1.16±0.29	1.10±0.36	0.86±0.30	1.18±0.55
C2_679	Hypoxanthine-guanine phosphoribosyltransferase	HPRT1	0.46±0.06	1.66±0.21	1.01±0.22	1.09±0.28
C2_13391	IgGFc-binding protein	FCGBP	2.12±0.13	0.99±0.20	1.09±0.36	1.25±0.36
C2_46036	Immunoglobulin M heavy chain	IGHM	1.06±0.14	2.16±0.78	0.83±0.16	0.93±0.14
C2_2478	Immunoglobulin-binding protein 1	IGBP1	0.45±0.20	1.65±0.73	5.58±2.84	2.20±0.62
C3_lrc8695	Importin subunit beta-1	KPNB1	1.26±0.19	1.49±0.31	1.69±0.38	1.45±0.25
C2_14281	Importin-7	IPO7	0.49±0.13	0.88±0.19	1.79±0.31	0.76±0.34
C2_78142	Inositol monophosphatase 2	IMPA2	1.49±0.28	1.94±0.32	1.65±0.55	1.12±0.11
C2_13269	Inositol polyphosphate 5-phosphatase OCRL-1	OCRL	0.48±0.18	1.24±0.31	1.35±0.32	1.06±0.34
C2_8119	Insulin-like growth factor binding protein complex acid liable subunit	IGFALS	1.76±0.30	1.19±0.07	1.10±0.10	1.43±0.30
C2_36156	Intelectin-2	ITLN2	2.04±0.36	0.92±0.10	0.99±0.44	1.11±0.37
FP333792	Inter-alpha-trypsin inhibitor heavy chain H2	ITIH2	0.83±0.33	1.14±0.06	1.37±0.51	1.09±0.22
C2_12615	Inter-alpha-trypsin inhibitor heavy chain H3	ITIH3	0.94±0.09	2.05±0.42	1.37±0.21	1.19±0.29
C3_lrc30120	Inter-alpha-trypsin inhibitor heavy chain H3-like	ITIH3	1.06±0.07	1.98±0.39	1.00±0.10	1.00±0.19
C2_624	Interferon-induced 35 kDa protein homolog	IFI35	1.66±0.21	1.19±0.09	1.04±0.14	1.39±0.36
C2_9595	Interferon-induced protein with tetratricopeptide repeats 1	IFIT1B	0.90±0.16	1.34±0.25	0.71±0.14	0.92±0.18
C2_8010	Interleukin enhancer-binding factor 2 homolog	ILF2	0.84±0.23	1.07±0.23	1.56±0.43	1.05±0.27
C2_118270	Intermediate filament protein ON3	ION3	0.59±0.10	1.11±0.39	1.06±0.16	0.90±0.14
C2_2049	Intestinal-type alkaline phosphatase	ALPI	1.30±0.14	1.41±0.19	1.41±0.36	1.38±0.31
C2_17229	Isochorismatase domain-containing protein 2, mitochondrial	ISOC2	1.15±0.16	1.24±0.18	0.97±0.18	0.74±0.11
C2_2044	Isopentenyl-diphosphate Delta-isomerase 1	IDI1	1.00±0.14	0.88±0.14	0.81±0.17	1.43±0.44
C2_2160	Isovaleryl-CoA dehydrogenase, mitochondrial	IVD	2.14±0.32	1.25±0.19	1.20±0.34	0.94±0.13
C2_18025	IST1 homolog	IST1	0.96±0.09	1.22±0.29	1.05±0.17	0.98±0.17
C2_2147	Junction plakoglobin	JUP	0.81±0.11	1.07±0.28	1.79±0.72	1.43±0.41
C2_23019	Junction plakoglobin	JUP	0.66±0.14	1.04±0.24	1.16±0.29	1.16±0.30
C2_1814	Junctional adhesion molecule A	F11R	0.87±0.05	1.06±0.10	1.22±0.17	0.82±0.18
C2_2372	Keratin, type I cytoskeletal 18	KRT18	1.56±0.20	1.16±0.15	0.95±0.10	1.09±0.19
C2_116635	Keratin, type I cytoskeletal 18-A	KRT18A	2.06±0.44	1.04±0.37	0.66±0.27	1.24±0.49
C2_86457	Keratin, type I cytoskeletal 19	KRT19	0.74±0.12	1.25±0.10	1.03±0.11	0.88±0.14
C2_1442	Keratin, type II cytoskeletal 8	KRT8	1.45±0.21	1.24±0.32	0.95±0.20	1.17±0.30
C2_2275	Ketohexokinase	KHK	1.42±0.10	1.54±0.27	1.12±0.07	1.55±0.31
C2_1049	KH domain-containing, RNA-binding, signal transduction-associated protein 1	KHDRBS1	0.64±0.08	1.26±0.25	1.12±0.21	1.28±0.40
C2_3990	Kinectin	Ktn1	1.03±0.12	0.58±0.11	1.07±0.29	1.02±0.16
C2_4980	Kinectin	Ktn1	0.73±0.30	0.63±0.07	1.83±1.01	1.80±0.80
C2_7678	Kinesin light chain 1	KLC1	0.86±0.11	0.77±0.08	0.93±0.28	0.89±0.09
C2_29495	Kininogen	Kng1/Kng1l1	1.82±0.26	1.13±0.15	1.07±0.09	0.90±0.23
C2_29253	Kynureninase	KYNU	1.21±0.12	1.05±0.16	0.93±0.17	0.84±0.06
C2_1795	Kynurenine--oxoglutarate transaminase 1	CCBL1	1.15±0.20	1.26±0.15	1.33±0.23	1.14±0.13
C2_17959	Lactase-phlorizin hydrolase	LCT	2.35±0.31	1.08±0.18	1.05±0.26	1.16±0.40
C2_5123	Lactase-phlorizin hydrolase	LCT	2.11±0.26	0.97±0.18	0.89±0.23	1.12±0.39
C2_380	Lactoylglutathione lyase	GLO1	1.77±0.23	0.99±0.15	0.94±0.19	1.26±0.27
C2_4354	Lambda-crystallin homolog	CRYL1	1.81±0.28	1.11±0.13	1.01±0.16	1.34±0.22
C2_36474	Lanosterol synthase	LSS	0.59±0.14	0.80±0.30	1.79±0.59	2.20±0.64
C2_21093	L-asparaginase	ASPG	1.33±0.19	1.35±0.29	1.16±0.29	1.40±0.40
C2_141	Latexin	LXN	1.46±0.16	1.26±0.19	0.83±0.13	0.84±0.18
C2_18261	Leucine-rich alpha-2-glycoprotein	LRG1	1.66±0.27	1.73±0.30	1.27±0.17	1.36±0.22
C2_2628	Leukocyte elastase inhibitor	SERPINB1	2.50±0.48	1.14±0.26	1.17±0.26	1.58±0.36
C2_695	Leukotriene A-4 hydrolase	LTA4H	1.43±0.30	1.75±0.20	1.20±0.17	1.16±0.21
C2_95063	Lipid phosphate phosphohydrolase 1	Ppap2a	1.69±0.49	0.71±0.19	0.86±0.26	1.55±0.60
C2_22890	Lipocalin	LCN1	1.00±0.09	1.07±0.12	1.06±0.21	1.13±0.12
C2_25312	Lipopolysaccharide-responsive and beige-like anchor protein	LRBA	0.97±0.11	1.25±0.22	1.02±0.15	1.03±0.14
C2_61943	LisH domain and HEAT repeat-containing protein KIAA1468 homolog	KIAA1468	1.94±0.36	1.30±0.43	0.97±0.09	1.06±0.17
C2_17725	Lissencephaly-1 homolog A	PAFAH1B1	1.15±0.20	1.16±0.15	0.94±0.11	0.90±0.17
C2_6672	Liver carboxylesterase	CES1	2.06±0.43	1.35±0.33	1.50±0.56	1.60±0.35
C2_13242	Liver carboxylesterase 2	CES2	2.17±0.61	1.21±0.33	1.28±0.52	1.32±0.25
C2_1393	L-lactate dehydrogenase A chain	LDHA	0.83±0.06	1.76±0.09	1.28±0.19	1.03±0.12
C2_6197	L-lactate dehydrogenase B chain	LDHB	0.85±0.07	1.10±0.15	1.02±0.19	1.08±0.13
C2_22040	Long-chain fatty acid transport protein 4	SLC27A4	1.12±0.12	0.95±0.18	1.30±0.14	1.08±0.13
C2_12825	Long-chain specific acyl-CoA dehydrogenase, mitochondrial	ACADL	1.43±0.24	1.08±0.15	1.18±0.27	1.23±0.23
C2_10278	Long-chain-fatty-acid--CoA ligase 5	ACSL5	0.76±0.05	0.60±0.11	0.87±0.27	1.19±0.34
C2_12320	Long-chain-fatty-acid--CoA ligase ACSBG2	ACSBG2	0.83±0.19	1.29±0.21	0.92±0.31	0.79±0.10
C2_57935	Low molecular weight phosphotyrosine protein phosphatase	ACP1	1.18±0.15	1.11±0.14	1.13±0.19	1.37±0.29
C2_8471	Lumican	LUM	1.01±0.10	2.97±0.97	1.54±0.21	1.56±0.27
C2_3622	Lysozyme g	LYG1	0.94±0.07	1.56±0.31	1.44±0.21	1.60±0.41
C2_3105	Lysyl-tRNA synthetase	KARS	0.80±0.06	0.77±0.10	1.11±0.14	0.85±0.09
C2_439	Macrophage migration inhibitory factor	MIF	1.95±0.37	1.21±0.18	1.25±0.17	1.73±0.32
C2_5305	Macrophage-capping protein	CAPG	1.16±0.15	1.40±0.10	0.95±0.06	1.00±0.22

C2_121640	Major histocompatibility complex class I-related gene protein	MR1	2.00±0.32	1.46±0.25	1.17±0.29	1.17±0.18
C2_72990	Major vault protein	MVP	0.91±0.12	1.31±0.59	3.13±1.31	2.28±0.95
C2_11452	Malate dehydrogenase, cytoplasmic	MDH1	0.56±0.03	1.55±0.33	1.24±0.23	1.16±0.31
C2_612	Malate dehydrogenase, mitochondrial	MDH2	1.11±0.12	1.51±0.14	1.89±0.35	1.53±0.48
C2_1846	Maltase-glucoamylase, intestinal	MGAM	2.26±0.41	0.93±0.16	0.92±0.24	1.69±0.74
C2_23669	Mannose-1-phosphate guanyltransferase beta	GMPPB	1.06±0.19	0.93±0.13	1.06±0.26	1.31±0.22
C2_19667	Mannose-6-phosphate isomerase	MPI	0.98±0.21	1.12±0.15	0.77±0.12	0.85±0.13
C2_27538	Mannosyl-oligosaccharide 1,2-alpha-mannosidase IA	MAN1A1	1.89±0.35	1.15±0.17	1.22±0.38	1.03±0.19
C2_1554	Medium-chain specific acyl-CoA dehydrogenase, mitochondrial	ACADM	1.81±0.32	1.15±0.23	1.30±0.21	1.04±0.24
C2_4129	Melanotransferrin	MFI2	1.87±0.29	1.55±0.52	0.84±0.18	0.95±0.15
C2_1403	Meprin A subunit alpha	MEP1A	2.74±0.63	0.84±0.07	0.81±0.27	0.85±0.19
C2_5461	Meprin A subunit beta	MEP1B	2.49±0.49	0.88±0.13	0.92±0.19	1.07±0.24
C2_1481	Mesoderm-specific transcript homolog protein	MEST	1.32±0.25	1.09±0.25	0.79±0.23	0.69±0.14
C2_5987	Methylmalonate-semialdehyde dehydrogenase [acylating], mitochondrial	ALDH6A1	1.03±0.07	1.35±0.15	1.66±0.49	1.00±0.11
C2_327	Methyltransferase-like protein 7A	METTL7A	0.96±0.08	0.77±0.17	0.97±0.18	1.14±0.26
C2_830	Microsomal glutathione S-transferase 3	MGST3	0.69±0.19	0.73±0.22	1.42±0.68	0.60±0.17
C2_7905	Microsomal triglyceride transfer protein large subunit	MTTP	0.66±0.05	0.92±0.16	1.37±0.32	0.88±0.17
C2_8412	Mitochondrial enolase superfamily member 1	ENOSF1	1.12±0.18	0.90±0.07	1.11±0.16	0.85±0.12
C2_198	Mitochondrial fission 1 protein	FIS1	0.43±0.07	1.18±0.28	0.88±0.25	0.92±0.28
C2_10488	Mitogen-activated protein kinase 1	MAPK1	0.68±0.10	0.87±0.15	0.76±0.15	1.25±0.45
C2_3688	Mitogen-activated protein kinase 14A	MAPK14	1.02±0.28	0.87±0.17	1.01±0.19	0.98±0.27
C2_86	Moesin	MSN	0.99±0.10	1.18±0.16	0.78±0.04	0.88±0.13
C2_99049	Monofunctional C1-tetrahydrofolate synthase, mitochondrial	MTHFD1L	0.99±0.28	0.71±0.06	0.80±0.19	1.51±0.36
C2_2814	Motile sperm domain-containing protein 2	MOSPD2	1.04±0.15	1.20±0.21	0.97±0.16	1.11±0.28
C2_1615	Mucin-13	MUC13	1.93±0.27	1.17±0.18	0.86±0.16	1.15±0.20
C2_3396	Mucin-2	MUC2	1.76±0.27	1.37±0.19	0.85±0.22	1.53±0.36
C2_22932	Mucin-2-like	MUC2-L	0.92±0.08	1.40±0.36	0.69±0.10	1.08±0.16
FP338380	Murinoglobulin-1	Mug1/Mug2	1.66±0.25	1.81±0.37	1.02±0.21	1.11±0.29
C2_3500	Myosin light polypeptide 6	MYL6	1.08±0.18	1.34±0.14	1.42±0.38	1.23±0.18
C2_838	Myosin regulatory light chain 2, smooth muscle minor isoform	MYL12B	1.26±0.26	1.35±0.11	1.77±0.41	1.15±0.20
C2_25606	Myosin-10	MYH10	0.87±0.25	1.67±0.73	1.47±0.37	1.15±0.50
C2_19728	Myosin-9	MYH9	0.86±0.12	0.84±0.09	1.06±0.11	1.02±0.10
C2_65856	Myosin-Ic	MYO1C	0.93±0.06	1.03±0.16	0.96±0.20	1.24±0.21
C2_62971	Myosin-Ic	MYO1C	0.95±0.21	0.97±0.18	1.01±0.13	0.95±0.28
C2_21024	N(G),N(G)-dimethylarginine dimethylaminohydrolase 1	DDAH1	1.35±0.25	1.50±0.16	0.98±0.14	0.91±0.13
C2_24043	Na(+)/H(+) exchange regulatory cofactor NHE-RF1	SLC9A3R1	0.90±0.04	0.89±0.06	1.32±0.40	1.45±0.29
C2_3136	Na(+)/H(+) exchange regulatory cofactor NHE-RF3	PDZK1	0.77±0.09	1.09±0.10	0.99±0.05	1.00±0.22
C2_50960	Na(+)/H(+) exchange regulatory cofactor NHE-RF4	PDZD3	0.74±0.24	0.90±0.38	1.12±0.23	1.67±0.42
C2_6312	N-acetylated-alpha-linked acidic dipeptidase-like protein	NAALADL1	1.63±0.18	1.02±0.16	0.97±0.18	1.11±0.28
C2_2369	N-acetyl-D-glucosamine kinase	NAGK	1.28±0.14	0.91±0.14	0.87±0.21	1.27±0.31
C2_14558	N-acetylgalactosamine kinase	GALK2	0.82±0.14	0.91±0.07	1.17±0.24	1.13±0.14
C2_2102	N-acyl-phosphatidylethanolamine-hydrolyzing phospholipase D	NAPEPLD	1.48±0.35	1.35±0.48	0.97±0.21	1.21±0.21
C2_4705	NAD-dependent malic enzyme, mitochondrial	ME2	1.12±0.19	1.26±0.24	1.20±0.31	1.01±0.24
C2_357	NADH-cytochrome b5 reductase 2	CYB5R2	1.26±0.22	1.07±0.13	1.56±0.40	1.55±0.36
C2_568	NADH-cytochrome b5 reductase 3	Cyb5r3	0.65±0.07	0.69±0.13	1.20±0.35	0.80±0.18
C2_1776	NADP-dependent isocitrate dehydrogenase	IDH1	0.58±0.04	1.67±0.54	1.43±0.34	1.37±0.31
C2_3984	NADP-dependent malic enzyme	ME1	0.79±0.08	1.17±0.15	1.29±0.21	1.29±0.26
C2_4956	NADP-dependent malic enzyme, mitochondrial	ME3	1.16±0.12	1.46±0.19	1.63±0.48	1.15±0.21
C2_2964	NADPH--cytochrome P450 reductase	POR	0.99±0.10	0.98±0.14	1.08±0.17	1.01±0.13
C2_9509	N-alpha-acetyltransferase 38, NatC auxiliary subunit	NAA38	1.47±0.24	1.20±0.13	1.54±0.22	1.33±0.14
C2_43416	Neutral alpha-glucosidase AB	GANAB	1.13±0.10	1.02±0.12	0.92±0.14	1.02±0.13
C2_4237	Neutral alpha-glucosidase AB	GANAB	1.28±0.14	1.11±0.21	1.31±0.22	1.30±0.20
C2_4635	Neutral and basic amino acid transport protein rBAT	SLC3A1	1.72±0.18	1.15±0.18	1.04±0.19	1.48±0.47
C2_18906	Neutral ceramidase	ASAH2	2.17±0.35	1.51±0.42	1.00±0.32	1.07±0.35
C2_14365	Neutral ceramidase	ASAH2	1.96±0.27	1.79±0.50	1.16±0.44	0.96±0.22
C2_2429	NHP2-like protein 1	NHP2L1	1.38±0.40	1.93±0.29	1.37±0.26	1.41±0.32
C2_5958	Nicastrin	NCSTN	2.44±0.46	1.25±0.35	1.32±0.25	1.70±0.37
C2_1457	Nicotinamide phosphoribosyltransferase	NAMPT	1.37±0.30	0.82±0.17	1.70±0.50	1.33±0.36
C2_4414	Niemann-Pick C1-like protein 1	NPC1L1	2.47±0.54	1.14±0.12	0.95±0.38	1.21±0.31
C2_14786	Non-lysosomal glucosylceramidase	GBA2	1.82±0.11	1.71±0.22	1.77±0.35	1.44±0.27
C2_6980	Non-lysosomal glucosylceramidase	GBA2	0.92±0.20	1.05±0.26	1.02±0.33	0.72±0.17
C2_183	Non-specific cytotoxic cell receptor protein 1 homolog	NCCRP1	1.84±0.23	0.92±0.09	0.73±0.13	1.25±0.31
C2_20656	Nuclear factor 7, ovary	EEF1A1	0.78±0.07	1.21±0.10	1.20±0.08	1.06±0.21
C2_1121	Nuclear migration protein nudC	NUDC	1.80±0.53	1.13±0.20	0.68±0.21	1.83±0.58
C2_206	Nucleolar protein 56	NOP56	31.25±19.12	25.67±15.84	17.53±16.50	6.93±6.61
C2_27287	Nucleolysin TIA-1 isoform p40	TIA1	1.32±0.21	1.03±0.13	1.05±0.26	1.36±0.24
C2_2403	Nucleophosmin	NPM1	1.56±0.30	0.87±0.18	0.96±0.29	1.60±0.61
C2_71	Nucleoside diphosphate kinase A	NME1	1.17±0.20	1.55±0.24	1.26±0.32	0.85±0.25
C2_113264	Nucleoside diphosphate kinase A1	NME1-1	1.10±0.48	2.56±0.40	2.24±0.50	1.93±0.61
C2_3380	Nucleosome assembly protein 1-like 1	NAP1L1	1.35±0.18	1.19±0.13	0.90±0.12	1.28±0.30
C2_19448	Nucleosome assembly protein 1-like 4	NAP1L4	1.05±0.20	1.77±0.35	1.61±0.31	1.31±0.23
C2_5052	Obg-like ATPase 1	OLA1	0.88±0.07	0.60±0.09	1.16±0.47	0.94±0.24
C2_5698	OCIA domain-containing protein 2	OCIAD2	0.72±0.10	0.58±0.06	1.29±0.39	1.04±0.28

C2_5167	Omega-amidase NIT2	NIT2	1.12±0.26	1.14±0.16	0.97±0.11	0.76±0.19
C2_20490	Oxysterol-binding protein-related protein 9	OSBPL9	14.19±12.45	1.58±0.44	2.49±1.02	1.40±0.63
C2_557	Palmitoyl-protein thioesterase 1	PPT1	1.50±0.30	0.87±0.19	0.87±0.16	1.03±0.27
C2_3526	Pancreatic alpha-amylase	AMY2B	2.70±0.34	0.63±0.10	0.92±0.48	1.60±0.68
C2_50293	Parvalbumin beta 2	PRVB2	1.37±0.25	0.96±0.09	0.91±0.31	0.92±0.30
C2_8807	PDZ domain-containing protein GIPC1	GIPC1	0.86±0.10	1.10±0.20	1.29±0.21	1.15±0.17
C2_9030	Pentraxin fusion protein	PXN1	1.23±0.48	1.66±0.48	1.03±0.40	0.81±0.27
C2_428	Peptidyl-prolyl cis-trans isomerase	FKBP4	0.80±0.22	1.91±0.20	1.42±0.29	0.93±0.33
C2_1296	Peptidyl-prolyl cis-trans isomerase	FKBP4	0.72±0.10	1.72±0.37	1.18±0.15	1.20±0.33
C2_1675	Peptidyl-prolyl cis-trans isomerase B	PPIB	0.71±0.09	1.25±0.31	1.29±0.24	1.33±0.40
C2_13338	Peptidyl-prolyl cis-trans isomerase D	PPID	2.41±0.87	3.32±0.94	1.18±0.27	1.95±0.46
C2_8702	Peptidyl-prolyl cis-trans isomerase FKBP1A	FKBP1A	0.88±0.12	1.18±0.25	1.12±0.15	1.06±0.17
C2_8872	Peptidyl-prolyl cis-trans isomerase NIMA-interacting 1	PIN1	0.87±0.24	1.01±0.20	0.88±0.15	0.93±0.29
C2_466	Peroxiredoxin-1	PRDX1	1.39±0.23	1.72±0.23	1.69±0.22	1.41±0.13
C2_10204	Peroxiredoxin-2	PRDX2	3.41±1.21	3.36±0.63	2.57±0.61	2.41±0.51
C2_579	Peroxiredoxin-4	PRDX4	1.76±0.17	1.45±0.24	1.24±0.25	1.67±0.37
C2_199	Peroxiredoxin-6	PRDX6	1.03±0.13	0.95±0.10	1.82±0.58	2.18±0.78
C2_8400	Peroxisomal 2,4-dienoyl-CoA reductase	DECR2	0.98±0.04	0.96±0.04	1.32±0.23	1.12±0.21
C2_62152	Peroxisomal acyl-coenzyme A oxidase 3	ACOX3	0.48±0.14	1.02±0.20	1.89±0.39	1.04±0.32
C2_30152	Peroxisomal carnitine O-octanoyltransferase	CROT	1.27±0.18	0.88±0.24	0.82±0.12	1.07±0.24
C2_4577	Peroxisomal multifunctional enzyme type 2	HSD17B4	0.86±0.10	0.79±0.08	1.24±0.22	1.29±0.21
C2_4281	Peroxisomal N(1)-acetyl-spermine/spermidine oxidase	PAOX	0.61±0.05	1.41±0.49	0.73±0.12	0.68±0.07
C2_9766	Peroxisomal sarcosine oxidase	PIPOX	0.92±0.10	1.16±0.27	1.25±0.29	1.32±0.53
C2_4069	Phenazine biosynthesis-like domain-containing protein 1	PBLD	2.21±0.33	1.29±0.20	1.12±0.11	1.56±0.24
C2_2810	Phenylalanyl-tRNA synthetase beta chain	FARSB	0.99±0.14	1.12±0.19	1.13±0.22	1.42±0.38
C2_3888	Phosphatidylcholine transfer protein	PCTP	1.10±0.15	0.97±0.24	1.01±0.24	0.89±0.14
C2_99	Phosphatidylethanolamine-binding protein 1	PEBP1	0.78±0.06	1.44±0.19	1.09±0.07	0.95±0.15
C2_1495	Phosphatidylinositol transfer protein alpha isoform	PITPNA	0.73±0.07	1.07±0.13	1.27±0.15	0.95±0.15
C2_5143	Phosphatidylinositol-4-phosphate 3-kinase C2 domain-containing subunit alpha	PIK3C2A	1.00±0.32	1.08±0.20	2.39±0.70	0.82±0.36
C2_19167	Phosphoacetylglucosamine mutase	PGM3	0.76±0.15	1.23±0.29	0.74±0.12	1.04±0.18
C2_12073	Phosphoenolpyruvate carboxykinase [GTP], mitochondrial	PCK2	0.81±0.09	1.25±0.26	1.32±0.35	1.06±0.31
C2_6725	Phosphoglucomutase-2	PGM2	1.26±0.19	1.24±0.08	0.96±0.09	0.99±0.17
C2_1379	Phosphoglycerate kinase 1	PGK1	1.24±0.18	1.55±0.12	1.16±0.07	1.11±0.17
C2_2366	Phosphoglycerate mutase 1	PGAM1	0.89±0.12	1.26±0.21	0.99±0.15	1.04±0.17
C2_40269	Phospholipase A-2-activating protein	PLAA	1.73±0.21	1.00±0.12	0.95±0.23	1.29±0.41
C2_4374	Phospholipase B-like 1	PLBD1	1.95±0.22	0.99±0.08	0.92±0.22	1.10±0.26
C2_85	Phospholipid hydroperoxide glutathione peroxidase, mitochondrial	GPX4	2.01±0.44	1.13±0.16	0.84±0.17	1.26±0.28
C2_1982	Phosphomannomutase 2	PMM2	1.09±0.14	1.56±0.27	1.27±0.05	1.39±0.30
C2_19159	Phosphotriesterase-related protein	PTER	1.88±0.32	1.02±0.17	0.94±0.13	1.39±0.29
C2_31716	Phytanoyl-CoA dioxygenase domain-containing protein 1	PHYHD1	0.80±0.05	0.96±0.12	0.95±0.18	1.00±0.10
C2_23687	Plasma protease C1 inhibitor	SERPING1	1.42±0.13	1.50±0.26	0.93±0.19	0.84±0.18
C2_26329	Plasma protease C1 inhibitor	SERPING1	1.29±0.19	1.34±0.27	0.77±0.16	0.87±0.16
C2_4248	Plastin-1	PLS1	0.73±0.07	1.04±0.25	1.09±0.30	1.09±0.27
C2_952	Plastin-2	LCP1	1.07±0.11	1.84±0.12	1.24±0.12	0.96±0.11
C2_6788	Plastin-3	PLS3	1.34±0.14	0.85±0.08	1.04±0.26	1.12±0.27
C2_5230	Platelet glycoprotein 4	CD36	1.84±0.37	1.28±0.31	1.12±0.27	1.19±0.23
C2_10639	Platelet-activating factor acetylhydrolase	PLA2G7	0.81±0.11	1.04±0.29	0.86±0.16	0.89±0.15
C2_2316	Platelet-activating factor acetylhydrolase IB subunit gamma	PAFAH1B3	0.94±0.12	1.13±0.05	0.84±0.08	0.93±0.17
C2_21485	Plectin	PLEC	1.45±0.39	2.46±0.56	3.45±0.94	2.25±0.41
C2_16967	Plectin	PLEC	1.04±0.08	1.34±0.27	1.65±0.39	1.06±0.10
C3_c30637	Plectin	PLEC	1.75±0.39	1.99±0.33	1.58±0.66	1.64±0.30
C2_11515	Poly [ADP-ribose] polymerase 1	PARP1	0.70±0.06	0.98±0.15	1.00±0.12	0.79±0.15
C2_2960	Poly(U)-binding-splicing factor PUF60	PUF60	2.31±0.49	1.30±0.26	1.10±0.28	2.10±0.88
C2_6459	Poly(U)-specific endoribonuclease	ENDOU	2.01±0.31	0.81±0.07	1.05±0.36	1.09±0.38
C2_3168	Polyadenylate-binding protein 1	PABPC1	1.06±0.15	1.02±0.10	1.06±0.20	1.45±0.39
C2_8361	Polypyrimidine tract-binding protein 1	PTBP1	1.06±0.13	1.26±0.27	0.94±0.14	1.01±0.10
C2_6689	Polyserase-2	PRSS36	2.37±1.31	1.15±0.28	1.15±0.41	0.72±0.19
C2_4566	Porphobilinogen deaminase	HMBS	0.95±0.16	1.06±0.18	1.44±0.14	1.12±0.25
C2_1110	Prefoldin subunit 1	PFDN1	0.94±0.16	0.84±0.09	1.00±0.14	1.25±0.16
C2_120921	Prefoldin subunit 3	VBP1	0.96±0.15	1.08±0.28	0.82±0.23	1.20±0.46
C2_41817	Pregnancy zone protein	PZP	1.42±0.24	1.35±0.25	0.95±0.22	0.99±0.25
C2_3804	Pre-mRNA-processing factor 39	PRPF39	1.26±0.35	1.63±0.25	1.41±0.20	1.00±0.23
C2_9221	Proactivator polypeptide	PSAPL1	1.43±0.30	1.36±0.14	0.91±0.21	1.29±0.33
C2_18944	Probable 4-hydroxy-2-oxoglutarate aldolase, mitochondrial	HOGA1	1.42±0.37	0.70±0.10	0.96±0.26	1.29±0.25
C2_1682	Probable aminopeptidase NPEPL1	NPEPL1	2.12±0.34	1.15±0.24	1.14±0.18	1.79±0.49
C2_8194	Probable arylformamidase	AFMID	1.87±0.82	1.68±0.21	2.98±1.58	2.24±0.85
C2_2903	Probable ATP-dependent RNA helicase DDX23	DDX23	1.09±0.36	1.15±0.60	0.40±0.11	2.53±1.71
C2_26495	Probable D-tyrosyl-tRNA(Tyr) deacylase 2	DTD2	1.43±0.56	3.13±1.11	2.82±1.09	1.25±0.30
C2_20669	Probable peptidyl-tRNA hydrolase 2	PTH2	1.11±0.30	1.45±0.25	1.03±0.24	0.99±0.25
C2_1150	Probable serine carboxypeptidase CPVL	CPVL	2.08±0.31	1.03±0.16	0.87±0.21	1.97±0.72
C2_11771	Probable thiopurine S-methyltransferase	TPMT	2.25±0.93	4.40±2.30	3.88±2.37	4.21±2.01
C2_7946	Profilin-2	PFN2	1.90±0.23	1.32±0.15	1.16±0.15	1.41±0.25
C2_22586	Programmed cell death 6-interacting protein	PDCD6IP	1.32±0.10	1.18±0.06	1.14±0.17	1.08±0.17
C2_20093	Programmed cell death protein 4	PDCD4	0.72±0.10	1.14±0.25	0.81±0.17	0.92±0.15
C2_1796	Prolactin regulatory element-binding protein	PREB	1.56±0.18	1.17±0.28	0.85±0.10	1.64±0.46
C2_447	Proliferation-associated protein 2G4	PA2G4	0.70±0.09	0.90±0.09	0.96±0.10	0.88±0.15
C2_816	Proline synthase co-transcribed bacterial homolog protein	PROSC	0.87±0.06	1.32±0.21	1.37±0.07	1.08±0.16

C2_19946	Prolyl endopeptidase	PREP	1.30±0.17	1.09±0.13	0.83±0.17	1.06±0.27
C2_35352	Prolyl endopeptidase	PREP	1.60±0.25	1.31±0.17	1.25±0.31	1.04±0.21
C2_4229	Prostamide/prostaglandin F synthase	FAM213B	0.90±0.13	0.82±0.07	1.28±0.26	1.11±0.12
C2_62037	Proteasomal ubiquitin receptor ADRM1	ADRM1	0.64±0.11	0.75±0.20	0.69±0.11	0.74±0.13
C2_19056	Proteasome activator complex subunit 1	PSME1	1.08±0.14	0.79±0.04	1.10±0.29	1.31±0.27
C2_52053	Proteasome activator complex subunit 2	PSME2	0.56±0.07	0.83±0.11	1.08±0.26	1.04±0.17
C2_276	Proteasome subunit alpha type-1	PSMA1	1.80±0.30	1.44±0.13	1.71±0.17	1.57±0.12
C2_39253	Proteasome subunit alpha type-2	PSMA2	2.26±0.44	1.55±0.27	1.60±0.17	1.48±0.15
C2_667	Proteasome subunit alpha type-3	PSMA3	1.91±0.23	1.14±0.20	0.94±0.21	1.24±0.39
C2_1506	Proteasome subunit alpha type-4	PSMA4	1.82±0.33	1.05±0.21	0.77±0.15	1.29±0.41
C2_89	Proteasome subunit alpha type-5	PSMA5	1.70±0.33	1.20±0.17	1.40±0.10	1.34±0.25
C2_979	Proteasome subunit alpha type-6	PSMA6	1.93±0.38	1.33±0.32	0.86±0.13	1.44±0.44
C2_486	Proteasome subunit alpha type-7	PSMA7	2.25±0.37	1.44±0.19	1.47±0.30	1.53±0.40
C2_303	Proteasome subunit beta type-1-B	PSMA1B	1.84±0.29	1.30±0.27	1.02±0.15	1.34±0.36
C2_4220	Proteasome subunit beta type-2	PSMB2	2.23±0.45	1.10±0.21	0.88±0.13	1.38±0.43
C2_1113	Proteasome subunit beta type-3	PSMB3	2.03±0.31	1.34±0.23	1.00±0.18	1.38±0.42
C2_1989	Proteasome subunit beta type-4	PSMB4	2.23±0.40	1.39±0.29	1.00±0.21	1.75±0.59
C2_2719	Proteasome subunit beta type-5	PSMB5	1.48±0.21	0.93±0.18	0.76±0.16	1.20±0.28
C2_104936	Proteasome subunit beta type-6-B like protein	PSMB6L-B	1.69±0.23	0.98±0.15	0.85±0.27	1.25±0.38
C2_909	Proteasome subunit beta type-7	PSMB7	1.56±0.36	1.04±0.18	0.70±0.06	1.20±0.25
C2_21676	Proteasome subunit beta type-8	PSMB8	2.05±0.25	1.33±0.24	1.06±0.15	1.56±0.56
C2_121103	Proteasome subunit beta type-9	PSMB9	1.78±0.24	1.04±0.16	0.83±0.14	1.10±0.21
C2_2075	Protein archease	ZBTB8OS	0.86±0.23	1.24±0.34	0.97±0.36	0.41±0.11
C2_3894	Protein disulfide-isomerase	P4HB	0.79±0.07	1.46±0.30	1.75±0.49	0.92±0.20
C2_251	Protein disulfide-isomerase A3	PDIA3	0.84±0.10	1.32±0.29	1.57±0.36	0.97±0.16
C2_2492	Protein disulfide-isomerase A4	PDIA4	1.11±0.10	1.35±0.16	1.73±0.30	1.15±0.14
C2_10370	Protein disulfide-isomerase A6	PDIA6	0.78±0.05	0.97±0.06	1.13±0.16	0.98±0.10
C2_229	Protein DJ-1	PARK7	1.34±0.15	1.07±0.12	1.11±0.18	1.25±0.11
C2_1216	Protein ETHE1, mitochondrial	ETHE1	0.90±0.24	1.89±0.23	1.88±0.54	1.07±0.26
C2_11022	Protein farnesyltransferase subunit beta	FNTB	0.33±0.14	1.05±0.12	0.95±0.25	0.62±0.22
C2_1062	Protein farnesyltransferase/geranylgeranyltransferase type-1 subunit alpha	FNTA	0.90±0.06	0.94±0.10	0.78±0.11	0.85±0.12
C2_28019	Protein kinase C and casein kinase substrate in neurons protein 1	PACSIN1	0.97±0.03	0.98±0.16	0.99±0.14	1.30±0.15
C2_24702	Protein mago nashi homolog 2	MAGOHB	0.83±0.15	1.34±0.19	0.90±0.15	0.72±0.12
C2_1083	Protein NDRG1	NDRG1	0.84±0.13	0.74±0.19	1.07±0.32	1.19±0.32
C2_2272	Protein NipSnap homolog 3A	NIPSNAP3A	1.35±0.14	1.04±0.11	1.28±0.15	0.93±0.06
C2_1971	Protein phosphatase 1 regulatory subunit 7	PPP1R7	0.77±0.12	0.92±0.14	0.84±0.09	0.85±0.12
C2_8910	Protein phosphatase 1A	PPM1A	0.83±0.11	0.98±0.20	1.08±0.25	0.80±0.23
C2_52239	Protein PRRC1	PRRC1	1.03±0.22	2.11±0.48	1.95±0.33	1.30±0.44
C2_1280	Protein S100-A10	S100A10	0.58±0.15	2.67±1.30	1.46±0.64	0.72±0.26
C2_8005	Protein SET	SET	1.49±0.12	1.56±0.21	1.34±0.18	1.33±0.14
C2_7030	Protein transport protein Sec24A	SEC24A	0.79±0.10	1.11±0.24	1.36±0.27	1.17±0.29
C2_324	Protein transport protein Sec61 subunit alpha-like 1	SEC61A1	0.46±0.11	0.80±0.20	0.99±0.33	0.95±0.24
C2_6352	Protein Z-dependent protease inhibitor	SERPINA10	1.78±0.22	1.70±0.18	1.40±0.17	1.37±0.24
C2_13589	Protein-arginine deiminase type-2	PADI2	0.79±0.21	1.56±0.36	1.49±0.26	1.05±0.12
C2_31468	Prothrombin	F2	0.68±0.09	0.89±0.14	0.67±0.06	0.90±0.16
C2_16527	Prothrombin	F2	1.11±0.20	1.57±0.38	1.13±0.17	1.26±0.19
C2_13732	Protocadherin-like wing polarity protein stan	STAN	1.87±0.26	1.28±0.16	1.12±0.22	1.27±0.19
C2_7122	Pseudouridine-5'-monophosphatase	HDHD1	0.70±0.10	1.17±0.16	1.04±0.12	0.83±0.10
C2_21104	Puromycin-sensitive aminopeptidase	NPEPPS	1.74±0.28	1.11±0.18	0.93±0.12	1.50±0.34
C2_23472	Putative acyl-CoA dehydrogenase AidB	AIDB	0.89±0.19	0.91±0.17	1.37±0.33	1.18±0.32
C2_3029	Putative all-trans-retinol 13,14-reductase	RETSAT	0.93±0.06	0.88±0.10	1.28±0.18	1.31±0.22
C2_95323	Putative alpha-L-fucosidase	FUCA2	1.69±0.22	1.28±0.18	1.07±0.46	1.73±0.49
C2_962	Putative aminopeptidase W07G4.4	W07G4.4	3.01±0.65	1.88±0.32	1.67±0.30	2.18±0.36
C2_980	Putative deoxyribose-phosphate aldolase	DERA	1.01±0.23	0.69±0.05	0.71±0.06	0.81±0.07
C2_721	Putative hydroxypyruvate isomerase	HYI	32.78±19.89	11.06±9.63	7.97±6.79	28.15±17.63
C2_4760	Putative L-aspartate dehydrogenase	ASPDH	1.41±0.11	1.24±0.17	1.13±0.10	1.06±0.15
C2_37616	Putative leucine-rich repeat-containing protein DDB_G0290503	DDB_G0290503	1.24±0.22	0.88±0.11	0.78±0.19	1.17±0.42
C2_1758	Putative methyltransferase DDB_G0268948	DDB_G0268948	0.67±0.11	1.03±0.15	1.00±0.09	1.07±0.14
C2_586	Putative phospholipase B-like 2	PLBD2	1.97±0.39	0.85±0.08	0.68±0.10	1.43±0.41
C2_2726	Putative serine protease K12H4.7	K12H4.7	1.94±0.28	0.94±0.18	0.53±0.11	1.13±0.37
C2_8233	Pyridoxal kinase	PDXK	2.18±0.41	1.62±0.28	1.14±0.13	1.50±0.26
C2_4524	Pyridoxine-5'-phosphate oxidase	PNPO	0.91±0.13	1.72±0.41	1.58±0.32	1.22±0.31
C2_2476	Pyroglutamyl-peptidase 1	PGPEP1	2.85±1.57	1.37±0.26	0.94±0.23	1.35±0.40
C2_2277	Pyruvate dehydrogenase E1 component subunit beta, mitochondrial	PDHB	1.02±0.19	0.98±0.17	1.12±0.22	1.15±0.20
C2_13692	Pyruvate kinase isozyme M1	PKM	1.55±0.28	0.97±0.13	0.92±0.17	1.58±0.26
C2_110881	Pyruvate kinase isozymes M1/M2	PKM12	0.59±0.11	1.59±0.28	1.19±0.24	1.15±0.38
C2_96589	Pyruvate kinase isozymes M1/M2	PKM12	0.61±0.11	1.09±0.11	1.09±0.19	0.78±0.21
C2_7049	Pyruvate kinase isozymes R/L	PKRL	0.53±0.09	1.19±0.26	0.90±0.22	0.88±0.17
C2_2874	Pyruvate kinase muscle isozyme	PKM1	0.74±0.07	1.30±0.13	1.46±0.28	1.27±0.30
C2_1390	Quinone oxidoreductase	CRYZ	1.08±0.21	1.52±0.32	1.35±0.31	1.17±0.23
C2_32560	Rab GDP dissociation inhibitor alpha	GDI1	0.98±0.08	1.15±0.09	0.84±0.08	0.93±0.06
C2_455	Rab GDP dissociation inhibitor beta	GDI2	0.89±0.12	1.35±0.11	1.01±0.08	0.98±0.15
C2_32535	RalBP1-associated Eps domain-containing protein 1	REPS1	1.63±0.12	0.75±0.05	0.67±0.15	0.97±0.17
C2_35658	Ras GTPase-activating protein-binding protein 2	G3BP2	1.08±0.15	1.28±0.12	1.25±0.09	0.92±0.23
C2_7014	Ras GTPase-activating-like protein IQGAP2	IQGAP2	8.76±6.59	1.96±0.49	2.24±0.69	2.91±0.93

C2_1097	Ras-related C3 botulinum toxin substrate 1	RAC1	1.46±0.09	1.29±0.16	1.33±0.31	1.83±0.36
C2_2067	Ras-related protein Rab-11B	RAB11B	1.09±0.06	0.99±0.08	1.32±0.15	1.32±0.28
C2_10036	Ras-related protein Rab-14	RAB14	1.09±0.18	0.97±0.03	1.19±0.10	1.30±0.11
C2_7624	Ras-related protein Rab-1A	RAB1A	1.12±0.07	1.27±0.07	1.20±0.07	1.04±0.09
C2_67687	Ras-related protein Rab-1B	RAB1B	1.70±0.51	2.29±0.44	3.36±0.59	2.53±0.80
C2_1465	Ras-related protein Rab-2A	RAB2A	0.89±0.09	1.03±0.11	0.91±0.06	0.90±0.08
C2_7828	Ras-related protein Rab-5C	RAB5C	1.63±0.60	1.02±0.09	1.28±0.17	1.08±0.16
C2_7252	Ras-related protein Rab-7a	RAB7A	1.02±0.07	1.19±0.10	1.35±0.18	1.07±0.10
C2_935	Ras-related protein Ral-B	RALB	1.37±0.21	1.49±0.32	1.29±0.36	1.34±0.21
C2_879	Ras-related protein Rap-1b	RAP1B	1.61±0.26	1.81±0.33	1.56±0.27	1.16±0.18
C2_1650	Receptor expression-enhancing protein 5	REEP5	0.75±0.23	1.31±0.28	0.79±0.21	0.62±0.21
C2_1647	Regucalcin	RGN	2.02±0.37	1.11±0.16	0.86±0.15	1.36±0.31
C2_2457	Regulator of microtubule dynamics protein 1	RMDN1	0.99±0.35	1.54±0.42	1.63±0.33	1.04±0.26
C2_12838	Renalase	RNLS	0.96±0.08	1.36±0.13	1.11±0.20	0.99±0.11
C2_933	Retinal dehydrogenase 2	ALDH1A2	2.27±0.36	1.48±0.19	1.18±0.10	1.45±0.20
C2_9252	Retinol dehydrogenase 3	RDH3	0.99±0.28	1.31±0.25	1.87±0.54	2.04±0.88
C2_41464	Retinol dehydrogenase 7	RDH7	1.08±0.14	1.43±0.77	1.91±0.56	1.09±0.10
C2_11901	Retinol-binding protein 2	RBP2	0.57±0.06	1.75±0.34	1.20±0.26	1.27±0.36
C2_241	Rho GDP-dissociation inhibitor 1	ARHGDIA	0.75±0.15	1.88±0.12	1.42±0.27	0.96±0.22
C2_30696	Rho GTPase-activating protein 1	ARHGAP1	0.79±0.26	1.79±0.71	1.02±0.33	1.31±0.56
C2_2718	Ribokinase	RBKS	1.96±0.44	1.23±0.11	0.80±0.16	1.07±0.20
C2_8404	Ribonuclease UK114	HRSP12	2.01±0.46	1.61±0.21	2.16±0.37	1.75±0.18
C2_28116	Ribose-5-phosphate isomerase	RPIA	1.31±0.17	1.26±0.21	1.23±0.17	1.40±0.30
C2_1415	Ribose-phosphate pyrophosphokinase 1	PRPS1	1.21±0.27	1.07±0.27	1.17±0.30	1.46±0.26
C2_2840	Ribosomal protein S6 kinase 2 alpha	RPS6KA2	0.82±0.08	0.89±0.07	0.90±0.15	0.93±0.13
C3_c19359	Ribosyldihydronicotinamide dehydrogenase [quinone]	NQO2	1.41±0.20	1.73±0.27	1.12±0.21	1.18±0.25
C2_6486	RNA-binding protein 47	RBM47	0.97±0.11	1.25±0.25	1.01±0.15	1.14±0.15
C2_972	S-adenosylmethionine synthase isoform type-2	MAT2A	0.68±0.17	0.70±0.15	1.10±0.40	1.69±0.56
C2_1565	Scavenger mRNA-decapping enzyme DcpS	DCPS	0.88±0.26	1.90±0.57	1.24±0.17	1.03±0.50
C2_13518	Scavenger receptor class B member 1	SCARB1	1.26±0.17	1.09±0.22	1.06±0.16	1.25±0.36
C2_19513	Selenide, water dikinase 1	SEPHS1	1.66±0.16	2.00±0.31	2.88±0.56	1.52±0.29
C2_6263	Selenide, water dikinase 2	SEPHS2	1.43±0.13	1.36±0.09	1.35±0.10	1.06±0.08
C2_4668	Selenium-binding protein 1	SELENBP1	1.50±0.15	1.87±0.15	1.91±0.25	1.86±0.16
C2_5603	Septin-2	SEPT2	1.02±0.06	1.21±0.17	1.03±0.15	1.07±0.08
C2_22270	Septin-6	SEPT6	0.82±0.14	0.92±0.10	0.90±0.17	1.00±0.13
C2_8875	Septin-7	SEPT7	1.56±0.35	1.37±0.15	2.63±0.56	1.52±0.28
C2_1375	Serine hydroxymethyltransferase, cytosolic	SHMT1	1.68±0.23	1.56±0.16	1.60±0.22	1.30±0.05
C2_8391	Serine/threonine-protein kinase 24	STK24	1.33±0.47	2.75±1.33	1.96±0.98	2.84±1.12
C2_7698	Serine/threonine-protein phosphatase 2A 65 kDa regulatory subunit A beta isoform	PPP2R1B	0.75±0.11	0.76±0.14	1.06±0.30	1.09±0.20
C2_11251	Serine/threonine-protein phosphatase 2A catalytic subunit alpha isoform	PPP2CA	1.87±0.64	0.96±0.09	0.72±0.14	1.12±0.32
C2_22338	Serine/threonine-protein phosphatase 2B catalytic subunit alpha isoform	PPP3CA	1.98±0.24	1.46±0.12	1.28±0.26	1.47±0.31
C2_7354	Serine/threonine-protein phosphatase 5	PPP5C	1.36±0.10	1.09±0.19	0.98±0.13	1.15±0.22
C2_2436	Serine/threonine-protein phosphatase 6 catalytic subunit	PPP6C	0.92±0.11	1.07±0.14	0.87±0.24	0.50±0.15
C2_553	Serine/threonine-protein phosphatase PP1-gamma catalytic subunit	Ppp1cc	1.45±0.12	0.85±0.19	0.80±0.16	1.09±0.21
C2_7158	Serotransferrin	TF	1.27±0.22	1.74±0.34	1.42±0.39	1.36±0.39
C2_112963	Serpin B6	SERPINB6	0.91±0.16	1.17±0.39	1.74±0.79	0.82±0.27
C2_110965	Serpin B8	SERPINB8	2.81±0.37	2.10±0.30	1.62±0.15	2.07±0.39
C2_14608	Serum amyloid P-component	APCS	0.89±0.25	1.08±0.10	1.95±0.68	1.51±0.48
C2_7237	Seryl-tRNA synthetase, cytoplasmic	SARS	0.83±0.11	0.61±0.09	1.06±0.30	1.11±0.35
C2_5020	Sex hormone-binding globulin	SHBG	0.79±0.23	0.99±0.07	1.10±0.10	1.00±0.17
C2_778	S-formylglutathione hydrolase	ESD	1.49±0.12	1.32±0.20	0.89±0.07	1.49±0.37
C2_122	SH3 domain-binding glutamic acid-rich-like protein	SH3BGRL	1.07±0.07	1.46±0.20	1.09±0.09	1.16±0.13
C2_107681	SH3 domain-binding glutamic acid-rich-like protein 3	SH3BGRL3	1.60±0.96	3.03±1.09	10.53±4.24	4.20±1.75
C2_2197	Sialic acid synthase	NANS	0.82±0.13	1.21±0.30	1.58±0.38	1.23±0.21
C2_1438	Sialidase-1	NEU1	1.76±0.29	0.82±0.10	0.78±0.10	1.26±0.39
C2_2871	Signal transducer and activator of transcription 3	STAT3	0.91±0.10	0.97±0.14	0.88±0.16	0.90±0.09
C2_4819	Small nuclear ribonucleoprotein E	SNRPE	1.16±0.17	1.13±0.06	1.12±0.24	1.07±0.24
C2_68502	Small nuclear ribonucleoprotein Sm D1	SNRPD1	1.73±0.42	1.75±0.30	1.42±0.16	1.19±0.17
C2_7038	Small nuclear ribonucleoprotein Sm D3	SNRPD3	2.20±0.49	1.68±0.17	1.41±0.26	1.24±0.27
C2_1659	Sodium-dependent neutral amino acid transporter B(0)AT3	SLC6A18	1.85±0.42	2.40±1.06	2.02±0.63	2.71±1.01
C2_1290	Sorbitol dehydrogenase	SORD	1.67±0.21	1.14±0.09	0.87±0.10	1.17±0.17
C2_408	Sorcin	SRI	1.03±0.23	1.12±0.11	1.03±0.10	1.05±0.21
C2_2983	Sorting nexin-1	SNX1	1.05±0.07	0.94±0.05	0.99±0.16	1.11±0.10
C2_3908	Sorting nexin-4	SNX4	0.91±0.16	1.16±0.15	1.23±0.22	1.37±0.33
C2_1441	Sorting nexin-5	SNX5	1.00±0.22	0.93±0.09	1.36±0.26	1.09±0.14
C2_10159	Sorting nexin-8	SNX8	0.84±0.11	0.97±0.07	1.10±0.15	0.92±0.10
C2_2143	Sorting nexin-9	SNX9	1.12±0.17	0.89±0.10	0.97±0.19	1.01±0.09
C2_963	Spectrin alpha chain, brain	SPTAN1	0.72±0.09	1.15±0.14	1.02±0.07	1.02±0.23
C2_46348	Spectrin alpha chain, brain	SPTAN1	0.43±0.11	0.98±0.31	1.01±0.15	0.99±0.31
C2_84720	Spectrin alpha chain, brain	SPTAN1	0.58±0.15	0.88±0.13	0.98±0.11	0.78±0.24
C2_46691	Spectrin alpha chain, brain	SPTAN1	0.62±0.14	0.94±0.24	0.85±0.24	0.99±0.22
C2_4387	Sphingosine-1-phosphate lyase 1	SGPL1	1.11±0.22	0.77±0.06	1.40±0.19	1.10±0.15
C2_7011	Spliceosome RNA helicase DDX39B homolog	DDX39B	1.03±0.10	1.31±0.20	1.66±0.36	1.10±0.26
C3_c39473	Splicing factor 3B subunit 4	SF3B4	2.37±1.09	1.67±0.32	1.75±0.50	1.17±0.20
C2_7753	Splicing factor U2AF 65 kDa subunit	U2AF2	1.24±0.24	1.38±0.24	0.90±0.16	1.22±0.27

C2_1302	Staphylococcal nuclease domain-containing protein 1	SND1	1.11±0.14	1.04±0.17	1.04±0.14	1.06±0.12
C2_8732	Stress-induced-phosphoprotein 1	STIP1	1.17±0.18	1.08±0.21	0.87±0.21	1.01±0.13
C2_1571	Succinate dehydrogenase [ubiquinone] flavoprotein subunit, mitochondrial	SDHA	1.29±0.23	1.07±0.26	1.32±0.36	1.36±0.41
C2_11441	Succinyl-CoA ligase [GDP-forming] subunit beta, mitochondrial	SUCLG2	0.80±0.13	0.68±0.22	1.10±0.38	1.18±0.39
C2_10991	Sucrase-isomaltase, intestinal	SI	2.13±0.32	1.18±0.19	1.23±0.26	1.83±0.76
C2_1085	Sulfotransferase 1C1	SULT1C3	0.71±0.06	0.74±0.05	1.07±0.33	1.02±0.27
C2_3986	Sulfotransferase 1C2	SULT1C2	1.08±0.29	1.15±0.21	1.69±0.33	2.30±0.75
C2_7228	Sulfotransferase 6B1	SULT6B1	0.96±0.17	1.08±0.21	1.15±0.38	1.64±0.44
C2_8495	Sulfotransferase family cytosolic 2B member 1	SULT2B1	0.50±0.07	0.59±0.12	0.73±0.15	0.58±0.09
C2_368	Superoxide dismutase [Cu-Zn]	SOD1	2.83±0.54	1.47±0.29	0.96±0.24	2.24±0.61
C2_1642	Superoxide dismutase [Mn], mitochondrial	SOD2	1.54±0.22	0.91±0.10	1.17±0.20	1.21±0.24
C2_4089	Sushi domain-containing protein 2	SUSD2	1.79±0.27	0.93±0.10	0.92±0.21	1.19±0.29
C2_31339	Synaptic vesicle membrane protein VAT-1 homolog	VAT1	1.31±0.27	1.30±0.19	1.05±0.15	1.05±0.16
C2_3925	Synaptophysin-like protein 1	SYPL1	1.26±0.12	0.80±0.24	1.21±0.38	1.39±0.44
C2_83924	Talin-1	TLN1	0.68±0.17	2.71±1.04	1.35±0.34	1.53±0.58
C2_76944	Talin-1	TLN1	1.12±0.21	1.26±0.15	1.51±0.35	1.82±0.33
C2_15378	Talin-1	TLN1	1.19±0.14	0.98±0.07	1.01±0.15	1.04±0.07
C2_41984	Talin-2	TLN2	1.09±0.10	1.06±0.09	0.81±0.17	1.03±0.10
C2_4568	TAR DNA-binding protein 43	TARDBP	0.48±0.16	1.82±0.55	0.92±0.27	1.14±0.25
C2_8321	Target of Myb protein 1	TOM1	1.37±0.18	1.71±0.32	2.05±0.23	1.46±0.21
C2_298	T-complex protein 1 subunit epsilon	CCT5	0.85±0.14	0.99±0.13	1.42±0.31	1.20±0.19
C2_1691	T-complex protein 1 subunit eta	CCT7	0.95±0.08	0.75±0.13	1.24±0.27	1.10±0.12
C2_1250	T-complex protein 1 subunit gamma	CCT3	1.00±0.07	0.79±0.14	1.04±0.24	1.05±0.17
C2_3533	T-complex protein 1 subunit zeta	CCT6A	1.13±0.24	0.77±0.07	1.06±0.20	1.19±0.27
C2_2347	Tetratricopeptide repeat protein 38	TTC38	0.99±0.22	1.83±0.14	1.12±0.13	1.00±0.21
C2_7496	Thiamin pyrophosphokinase 1	TPK1	1.26±0.17	1.17±0.17	0.90±0.19	0.85±0.08
C2_15542	Thimet oligopeptidase	THOP1	1.67±0.13	1.06±0.15	1.04±0.24	1.29±0.28
C2_71169	Thimet oligopeptidase	THOP1	1.57±0.17	0.91±0.16	0.95±0.24	1.31±0.31
C2_1158	Thioredoxin	TXN	0.45±0.07	1.46±0.44	1.36±0.35	1.29±0.44
C2_5341	Thioredoxin domain-containing protein 5	TXNDC5	0.67±0.18	0.86±0.24	1.39±0.38	0.89±0.23
C2_1093	Thioredoxin reductase 3	TXNRD3	1.92±0.29	1.40±0.16	1.22±0.11	1.28±0.15
C2_1183	Thioredoxin-like protein 1	TXNL1	1.89±0.27	1.50±0.27	1.12±0.16	1.16±0.22
C2_13380	Thiosulfate sulfurtransferase/rhodanese-like domain-containing protein 1	TSTD1	0.75±0.10	2.07±0.80	1.89±0.91	1.25±0.35
C2_7672	Threonine synthase-like 2	THNSL2	0.84±0.07	0.76±0.09	0.97±0.16	0.95±0.08
C2_3045	Threonyl-tRNA synthetase, cytoplasmic	TARS	0.77±0.12	1.27±0.13	1.00±0.13	0.88±0.21
C2_1974	Toll-interacting protein	TOLLIP	0.76±0.11	1.15±0.07	0.94±0.06	1.01±0.09
C2_18467	Trans-1,2-dihydrobenzene-1,2-diol dehydrogenase	DHDH	0.89±0.08	0.98±0.21	1.07±0.13	1.10±0.14
C2_564	Transaldolase	TALDO1	0.68±0.08	1.76±0.29	1.31±0.14	1.09±0.31
C2_3567	Transferrin receptor protein 1	TFRC	1.13±0.17	0.86±0.13	1.27±0.30	1.29±0.11
C2_4791	Transgelin	TAGLN	1.04±0.12	1.77±0.55	0.93±0.24	0.92±0.06
C2_15562	Transitional endoplasmic reticulum ATPase	VCP	1.04±0.13	1.22±0.25	2.03±0.57	1.68±0.43
C3_c25177	Transitional endoplasmic reticulum ATPase	VCP	0.67±0.13	0.75±0.22	1.40±0.46	0.96±0.27
C3_c6442	Transketolase	TKT	2.05±0.50	1.41±0.17	1.10±0.17	1.57±0.28
C2_824	Transketolase-like protein 2	TKTL2	1.76±0.33	1.22±0.23	1.18±0.24	1.23±0.18
C2_15	Translationally-controlled tumor protein homolog	TPT1	1.41±0.22	1.17±0.16	0.91±0.19	1.54±0.35
C2_23686	Translin	TSN	1.95±0.33	1.50±0.24	1.34±0.16	1.52±0.35
C2_5754	Trehalase	TREH	2.04±0.42	1.04±0.27	0.89±0.36	0.74±0.13
C2_10341	Tricarboxylate transport protein, mitochondrial	Slc25a1	0.84±0.12	0.77±0.21	1.67±0.66	0.70±0.08
C2_2435	Trifunctional enzyme subunit beta, mitochondrial	HADHB	0.84±0.19	0.66±0.21	0.82±0.35	1.09±0.30
C2_52	Triosephosphate isomerase B	TPI1	1.53±0.33	1.80±0.18	1.32±0.10	1.44±0.24
C2_26986	tRNA (adenine-N(1)-)-methyltransferase non-catalytic subunit TRM6	TRMT6	0.74±0.04	0.99±0.30	0.88±0.16	0.81±0.13
C2_1213	Trypsin	2210010C04Rik	3.57±0.67	0.48±0.11	0.67±0.49	2.31±0.96
C2_84054	Trypsin-1	PRSS1	3.25±0.79	0.62±0.10	0.68±0.39	1.43±0.52
C2_121419	Trypsin-2	PRSS2	1.49±0.32	0.61±0.05	0.48±0.14	1.15±0.38
C2_4459	Trypsin-3	PRSS3	1.95±0.26	0.93±0.14	1.18±0.36	1.04±0.31
C2_38179	Tubulin alpha-1A chain	TUBA1A	1.11±0.14	0.94±0.05	1.38±0.42	1.33±0.24
C2_7711	Tubulin alpha-1B chain	TUBA1B	0.86±0.12	1.02±0.11	1.26±0.12	1.28±0.23
C2_905	Tubulin beta chain	TUBB	0.41±0.08	0.51±0.06	1.64±0.79	0.82±0.37
C2_14202	Tubulin beta-2C chain	TUBB4B	0.89±0.12	0.91±0.08	1.11±0.25	1.41±0.34
C2_2978	Tubulin-folding cofactor B	TBCB	1.01±0.21	1.62±0.26	1.57±0.36	1.69±0.25
C2_2326	Tumor necrosis factor receptor type 1-associated DEATH domain protein	TRADD	1.13±0.25	1.15±0.26	2.15±0.81	1.39±0.17
C2_6726	Tumor susceptibility gene 101 protein	TSG101	0.99±0.17	1.00±0.18	0.75±0.06	1.03±0.07
C2_877	Tumor-associated calcium signal transducer 2	TACSTD2	1.38±0.16	1.28±0.21	1.63±0.34	1.48±0.38
C2_6143	Twinfilin-1	TWF1	0.73±0.17	1.01±0.13	1.12±0.21	0.89±0.12
C2_12783	Type-4 ice-structuring protein LS-12	AFP4	0.46±0.04	0.29±0.05	2.59±1.47	1.83±1.13
C2_2163	Tyrosine-protein kinase CSK	CSK	1.30±0.14	1.22±0.22	0.80±0.17	0.97±0.12
C2_16846	Tyrosine-protein phosphatase non-receptor type 11	PTPN11	1.11±0.16	0.70±0.07	0.78±0.04	0.79±0.11
C2_26124	U6 snRNA-associated Sm-like protein LSm3	LSM3	1.17±0.19	1.02±0.14	0.99±0.15	1.01±0.15
C2_4328	U6 snRNA-associated Sm-like protein LSm4	LSM4	1.48±0.35	2.17±0.39	2.50±0.40	1.38±0.37
C2_2288	Ubiquilin-2	UBQLN2	1.00±0.06	1.10±0.05	1.03±0.09	1.11±0.16
C2_1964	Ubiquitin carboxyl-terminal hydrolase 14	USP14	1.00±0.12	0.94±0.06	1.33±0.20	1.30±0.13
C2_107070	Ubiquitin carboxyl-terminal hydrolase 21	USP21	1.48±0.74	2.50±0.63	2.27±0.46	1.85±0.68
C2_18831	Ubiquitin carboxyl-terminal hydrolase 7	USP7	1.43±0.13	1.34±0.14	1.26±0.22	1.13±0.16
C2_660	Ubiquitin carboxyl-terminal hydrolase isozyme L3	UCHL3	0.87±0.08	1.14±0.17	1.26±0.29	1.21±0.26

C2_470	Ubiquitin fusion degradation protein 1 homolog	UFD1L	1.64±0.17	1.06±0.10	0.95±0.08	1.20±0.22
C2_155	Ubiquitin thioesterase OTUB1	Otub1	0.70±0.12	0.91±0.16	0.96±0.21	0.85±0.10
C2_33738	Ubiquitin-40S ribosomal protein S27a	RPS27A	2.02±0.39	0.93±0.24	0.82±0.33	1.68±0.55
C2_5227	Ubiquitin-conjugating enzyme E2 D2	UBE2D2	0.71±0.11	1.17±0.09	0.75±0.14	0.81±0.12
C2_6947	Ubiquitin-conjugating enzyme E2 L3	UBE2L3	0.96±0.17	1.47±0.32	1.29±0.33	1.43±0.41
C2_17030	Ubiquitin-conjugating enzyme E2 N	UBE2N	0.85±0.07	0.90±0.06	1.27±0.34	1.08±0.16
C2_8231	Ubiquitin-like modifier-activating enzyme 1	UBA1	0.77±0.07	0.94±0.16	0.96±0.19	1.01±0.13
C2_6707	Ubiquitin-like modifier-activating enzyme ATG7	ATG7	1.31±0.38	0.98±0.12	1.02±0.08	0.82±0.10
C2_29802	Ubiquitin-like protein FUBI	FAU	0.88±0.12	0.88±0.16	2.22±0.73	2.27±1.11
C2_5019	Ubiquitin-like-conjugating enzyme ATG3	ATG3	1.38±0.26	0.98±0.10	1.52±0.27	1.64±0.16
C2_13734	UBX domain-containing protein 1	UBXN1	1.04±0.26	1.42±0.39	1.37±0.31	1.08±0.26
C2_7714	UDP-glucose 4-epimerase	GALE	1.11±0.11	1.18±0.24	1.22±0.22	1.23±0.12
C2_11633	UDP-glucose 6-dehydrogenase	UGDH	0.63±0.06	1.32±0.30	2.16±0.64	1.52±0.38
C2_3599	UDP-glucuronosyltransferase	UGT1A1	1.03±0.18	1.16±0.35	1.22±0.30	1.24±0.16
C2_12213	UDP-glucuronosyltransferase 1-9	UGT1A9	1.05±0.16	0.84±0.12	1.17±0.10	1.71±0.34
C2_7856	UDP-glucuronosyltransferase 2A1	UGT2A1	1.12±0.18	1.08±0.37	1.77±0.43	1.04±0.17
C2_5354	UDP-glucuronosyltransferase 2A2	UGT2A2	1.43±0.34	1.19±0.43	1.84±0.46	1.23±0.19
C2_24614	UDP-glucuronosyltransferase 2A3	UGT2A3	1.15±0.21	1.10±0.27	1.55±0.49	0.96±0.20
C2_54573	UDP-glucuronosyltransferase 2B15	UGT2B7	1.14±0.13	0.90±0.32	1.78±0.69	0.85±0.12
C2_111727	UDP-glucuronosyltransferase 2B17	UGT2B17	1.22±0.29	0.89±0.19	0.99±0.19	1.06±0.28
C2_12651	UDP-N-acetylhexosamine pyrophosphorylase	UAP1	0.53±0.05	0.88±0.28	0.92±0.25	0.83±0.17
C2_7433	UDP-N-acetylhexosamine pyrophosphorylase-like protein 1	UAP1L1	1.03±0.17	1.16±0.23	1.16±0.37	1.52±0.31
C2_6090	UMP-CMP kinase	CMPK1	0.76±0.09	1.04±0.27	1.75±0.67	1.77±0.63
C2_3672	Uncharacterized oxidoreductase C663.06c	SPCC663.06c	1.07±0.13	1.67±0.26	1.09±0.19	1.16±0.14
C2_9334	UPF0160 protein MYG1, mitochondrial	C12orf10	1.75±0.26	1.17±0.18	0.92±0.09	1.37±0.28
C2_6389	UPF0308 protein C9orf21	C9orf21	1.42±0.53	1.38±0.30	0.99±0.31	1.90±0.48
C2_5877	UPF0462 protein C4orf33 homolog	C4orf33	2.36±0.57	1.27±0.33	0.89±0.43	0.84±0.20
C2_41651	UPF0505 protein C16orf62 homolog	C16orf62	0.86±0.33	1.27±0.32	1.23±0.31	1.08±0.65
C2_19805	UPF0552 protein C15orf38 homolog	C15orf38	1.10±0.22	1.23±0.14	1.14±0.18	1.36±0.23
C2_31615	UPF0553 protein C9orf64 homolog	C9orf64	1.19±0.15	1.02±0.18	0.78±0.16	0.78±0.10
C2_2220	UPF0556 protein C19orf10 homolog	MYDGF	1.80±0.20	0.93±0.21	0.87±0.22	1.60±0.65
C2_1340	Uridine phosphorylase 1	UPP1	1.08±0.15	1.35±0.22	0.92±0.12	0.96±0.14
C2_4863	UTP--glucose-1-phosphate uridylyltransferase	UGP2	1.13±0.14	0.98±0.12	1.14±0.17	1.06±0.10
C2_20296	UV excision repair protein RAD23 homolog A	RAD23A	1.31±0.29	1.07±0.12	1.28±0.14	1.27±0.20
C2_15352	UV excision repair protein RAD23 homolog B	RAD23B	4.01±2.29	1.78±0.67	2.12±0.77	1.73±0.49
C2_8488	Vacuolar protein sorting-associated protein 26B-like	VPS26BL	1.09±0.21	1.03±0.15	1.09±0.20	1.20±0.22
C2_6316	Vacuolar protein sorting-associated protein 29	VPS29	0.96±0.07	1.13±0.16	1.27±0.17	1.20±0.11
C2_3610	Vacuolar protein sorting-associated protein 35	VPS35	1.03±0.22	1.33±0.28	1.22±0.12	1.00±0.14
C2_2867	Vacuolar protein sorting-associated protein 4B	VPS4B	0.97±0.14	1.29±0.10	0.98±0.17	1.34±0.32
C2_4706	Vacuolar protein-sorting-associated protein 36	VPS36	0.73±0.10	0.83±0.16	1.04±0.38	1.22±0.38
C2_5562	Very long-chain specific acyl-CoA dehydrogenase, mitochondrial	ACADVL	1.72±0.30	1.05±0.17	0.89±0.13	1.38±0.31
C2_47981	Very long-chain specific acyl-CoA dehydrogenase, mitochondrial	ACADVL	1.54±0.20	1.48±0.28	1.22±0.24	1.39±0.22
C2_8296	Vesicle-fusing ATPase	NSF	1.15±0.40	1.16±0.25	1.32±0.21	1.11±0.19
C2_1304	Vigilin	HDLBP	1.05±0.16	0.78±0.06	0.94±0.19	1.15±0.15
C2_10632	Villin-1	VIL1	0.74±0.04	1.28±0.14	1.57±0.18	1.34±0.24
C2_61501	Villin-1	VIL1	0.89±0.26	1.70±0.47	1.70±0.37	1.46±0.34
C2_9594	Vinculin	VCL	0.87±0.19	0.92±0.26	1.26±0.26	1.25±0.20
C2_44280	Vinculin	VCL	1.57±0.24	1.27±0.42	1.80±0.55	1.76±0.69
C2_661	Voltage-dependent anion-selective channel protein 2	VDAC2	1.36±0.21	1.00±0.27	1.57±0.51	1.25±0.14
C2_119048	Voltage-dependent anion-selective channel protein 3	VDAC3	1.46±0.41	1.15±0.27	1.82±0.63	1.14±0.13
C2_2397	von Willebrand factor A domain-containing protein 5A	VWA5A	1.48±0.19	1.39±0.22	1.09±0.17	1.24±0.24
C2_3806	V-type proton ATPase catalytic subunit A	ATP6V1A	1.06±0.06	1.00±0.06	1.29±0.23	1.52±0.20
C2_1084	V-type proton ATPase subunit B	ATP6V0A4	1.30±0.21	0.75±0.04	1.12±0.24	1.30±0.21
C2_5241	V-type proton ATPase subunit E 1	ATP6V0E1	1.29±0.10	0.88±0.11	1.26±0.29	1.24±0.19
C2_18009	V-type proton ATPase subunit G 1	ATP6V1G1	0.99±0.20	0.61±0.10	0.84±0.15	1.32±0.29
C2_275	V-type proton ATPase subunit H	ATP6V1H	0.74±0.12	0.72±0.11	0.78±0.11	0.78±0.07
C2_15764	WD repeat domain phosphoinositide-interacting protein 3	WDR45B	17.54±16.50	16.50±13.32	17.92±10.41	30.05±18.48
C2_1420	WD repeat-containing protein 1	WDR1	2.51±0.61	1.42±0.21	1.23±0.14	1.27±0.16
C2_1995	WD repeat-containing protein 61	WDR61	1.79±0.38	1.27±0.17	0.98±0.19	0.99±0.23
C2_4369	Xaa-Pro aminopeptidase 1	XPNPEP1	1.59±0.24	1.57±0.28	1.15±0.20	1.26±0.15
C2_2468	Xaa-Pro aminopeptidase 2	XPNPEP2	2.23±0.41	1.26±0.28	0.94±0.29	0.87±0.17
C2_1116	Xaa-Pro dipeptidase	PEPD	2.22±0.34	1.16±0.24	0.84±0.19	1.88±0.57
C2_17124	Xanthine dehydrogenase/oxidase	XDH	1.61±0.47	1.10±0.11	1.02±0.22	1.22±0.22
C2_10177	Xylose isomerase	XYLA	1.41±0.20	0.89±0.11	0.86±0.10	1.25±0.17
C2_35539	Zinc finger protein RFP	TRIM27	1.26±0.14	0.99±0.06	1.11±0.12	1.33±0.23
C2_16098	Zinc-binding alcohol dehydrogenase domain-containing protein 2	ZADH2	0.96±0.10	1.08±0.15	1.11±0.16	0.96±0.08
C2_39339	Zonadhesin	ZAN	2.39±0.23	0.88±0.25	0.76±0.26	1.09±0.34
C2_90139	Zonadhesin	ZAN	1.33±0.15	0.87±0.12	0.75±0.15	1.04±0.20
