# Supplementary material for: Under control: how a dietary additive can restore the gut microbiome and proteomic profile, and improve disease resilience in a marine teleostean fish fed vegetable diets
Source: Microbiome. 2017 Dec 28;5:164. doi: 10.1186/s40168-017-0390-3 (PMC5745981; doi:10.1186/s40168-017-0390-3)
Supplement: Supplementary file 9 — List of differentially expressed proteins (P < 0.05, one-way ANOVA). Data on protein expression are mean ± SEM of 6 fish fed the experimental diets. Different superscript letters in each row indicate significant differences among experimental groups. The number of the contig in the Sea Bream Database (http://nutrigroup-iats.org/seabreamdb) is indicated. (DOCX 63 kb) [file 40168_2017_390_MOESM9_ESM.docx]

**Table S4.** List of differentially expressed proteins (*p* < 0.05, one-way ANOVA). Data on protein expression are mean ± SEM of 6 fish fed the experimental diets. Different superscript letters in each row indicate significant differences among experimental groups. The number of the contig in the Sea Bream Database (http://nutrigroup-iats.org/seabreamdb) is indicated.

|  |  |  |  | Diet | | | | |
| --- | --- | --- | --- | --- | --- | --- | --- | --- |
| **Contig** | **Protein description** | **Symbol** | **D1** | **D2** | **D3** | **D4** | ***p* value** | **Cluster** |
| C2_15440 | 1-phosphatidylinositol phosphodiesterase | PLCA | 1.43±0.14 ^a^ | 0.77±0.05 ^b^ | 0.63±0.14 ^b^ | 0.97±0.26 ^ab^ | 0.014 | 3 |
| C2_1520 | 26S protease regulatory subunit 10B | PSMC6 | 0.77±0.04 ^a^ | 1.13±0.14 ^ab^ | 1.59±0.2 ^b^ | 1.11±0.21 ^ab^ | 0.018 | 4 |
| C2_850 | 3-hydroxybutyrate dehydrogenase type 2 | BDH2 | 1.75±0.18 ^a^ | 1.21±0.1 ^ab^ | 1.05±0.15 ^b^ | 1.23±0.16 ^ab^ | 0.021 | 3 |
| C2_971 | 40S ribosomal protein S17 | RPS17 | 1.04±0.26 ^a^ | 1.26±0.39 ^a^ | 6.43±2.19 ^b^ | 1.80±0.75 ^a^ | 0.013 | 4 |
| C2_593 | 40S ribosomal protein S4 | RPS4 | 0.61±0.05 ^a^ | 0.85±0.13 ^ab^ | 1.71±0.35 ^b^ | 1.08±0.33 ^ab^ | 0.034 | 2 |
| C2_5124 | 5'(3')-deoxyribonucleotidase, mitochondrial | NT5M | 0.93±0.07 ^a^ | 1.51±0.14 ^b^ | 1.28±0.13 ^ab^ | 1.14±0.14 ^ab^ | 0.022 | 4 |
| C2_46323 | 60S acidic ribosomal protein P1 | RPLP1 | 0.71±0.16 ^a^ | 1.49±0.06 ^b^ | 1.66±0.28 ^a^ | 1.00±0.26 ^ab^ | 0.018 | 4 |
| C2_6923 | 60S acidic ribosomal protein P2 | RPLP2 | 1.26±0.11 ^a^ | 3.01±0.42 ^b^ | 2.95±0.8 ^ab^ | 1.69±0.29 ^ab^ | 0.036 | 4 |
| C2_343 | 60S ribosomal protein L27a | RPL27A | 0.9±0.13 ^a^ | 1.04±0.22 ^a^ | 2.23±0.54 ^b^ | 1.87±0.30 ^ab^ | 0.03 | 4 |
| C2_14692 | 6-pyruvoyl tetrahydrobiopterin synthase | PTS | 1.63±0.21 ^a^ | 1.12±0.15 ^ab^ | 0.87±0.07 ^b^ | 1.29±0.22 ^ab^ | 0.036 | 1 |
| C2_7078 | Adenosine deaminase | ADA | 1.54±0.16 ^a^ | 0.97±0.09 ^ab^ | 0.85±0.09 ^b^ | 1.26±0.23 ^ab^ | 0.024 | 1 |
| C2_41283 | Adenylyl cyclase-associated protein 1 | CAP1 | 1.13±0.20 ^a^ | 2.46±0.17 ^b^ | 2.42±0.5 ^ab^ | 1.59±0.44 ^ab^ | 0.041 | 4 |
| C2_3814 | Aldehyde dehydrogenase family 9 member A1 | ALDH8A1 | 0.72±0.06 ^a^ | 1.15±0.16 ^ab^ | 1.49±0.19 ^b^ | 0.84±0.16 ^a^ | 0.009 | 2 |
| C2_3792 | Aldose 1-epimerase | GALM | 1.74±0.25 ^a^ | 1.06±0.14 ^b^ | 0.86±0.14 ^b^ | 1.21±0.15 ^b^ | 0.013 | 1 |
| C2_52709 | Alpha-methylacyl-CoA racemase | AMACR | 1.70±0.22 ^a^ | 0.96±0.16 ^b^ | 0.96±0.24 ^b^ | 0.90±0.21 ^b^ | 0.04 | 3 |
| C2_49201 | Angiotensin-converting enzyme | ACE | 2.32±0.37 ^a^ | 1.04±0.28 ^b^ | 0.71±0.23 ^b^ | 0.92±0.35 ^b^ | 0.007 | 1 |
| C2_94051 | Angiotensin-converting enzyme | ACE | 2.43±0.37 ^a^ | 1.26±0.22 ^ab^ | 0.91±0.26 ^b^ | 1.24±0.34 ^ab^ | 0.011 | 1 |
| C2_31056 | Angiotensin-converting enzyme | ACE | 2.66±0.55 ^a^ | 1.34±0.32 ^b^ | 1.08±0.37 ^b^ | 1.11±0.38 ^b^ | 0.042 | 1 |
| C2_1538 | Angiotensin-converting enzyme 2 | ACE2 | 2.22±0.41 ^a^ | 0.83±0.09 ^b^ | 0.81±0.26 ^b^ | 1.03±0.24 ^b^ | 0.004 | 1 |
| C2_89317 | Angiotensin-converting enzyme 2 | ACE2 | 2.08±0.41 ^a^ | 0.71±0.10 ^b^ | 0.73±0.31 ^b^ | 0.90±0.24 ^b^ | 0.008 | 1 |
| C2_1919 | Anionic trypsin-2 | PRSS2 | 2.74±0.46 ^a^ | 0.84±0.12 ^b^ | 0.86±0.30 ^b^ | 1.59±0.50 ^ab^ | 0.006 | 1 |
| C2_6208 | Apical endosomal glycoprotein | MAMDC4 | 1.28±0.12 ^a^ | 0.70±0.09 ^b^ | 0.65±0.11 ^b^ | 0.86±0.19 ^b^ | 0.014 | 3 |
| C3_c2363 | Apolipoprotein B-100-like | APOB | 1.29±0.15 ^a^ | 1.39±0.26 ^a^ | 0.56±0.10 ^b^ | 0.65±0.11 ^b^ | 0.003 | 3 |
| C2_6887 | Apoptosis-inducing factor 3 | AIFM3 | 0.76±0.34 ^a^ | 2.45±0.64 ^b^ | 1.78±0.22 ^ab^ | 1.12±0.26 ^ab^ | 0.035 | 4 |
| C2_645 | Aspartate aminotransferase, mitochondrial | GOT2 | 2.00±0.33 ^a^ | 1.02±0.14 ^b^ | 0.98±0.22 ^b^ | 1.38±0.34 ^ab^ | 0.05 | 1 |
| C2_4570 | Aspartoacylase | ASPA | 1.65±0.12 ^a^ | 1.01±0.17 ^ab^ | 0.88±0.21 ^b^ | 1.11±0.21 ^ab^ | 0.032 | 1 |
| C2_15853 | Aspartyl aminopeptidase | DNPEP | 2.21±0.39 ^a^ | 0.95±0.22 ^ab^ | 0.63±0.15 ^b^ | 1.71±0.55 ^ab^ | 0.022 | 1 |
| C2_5459 | Beta-ureidopropionase | UPB1 | 1.74±0.09 ^a^ | 1.01±0.18 ^b^ | 0.88±0.22 ^b^ | 1.32±0.31 ^ab^ | 0.047 | 1 |
| C2_73027 | Bile salt-activated lipase | CEL | 1.34±0.19 ^a^ | 0.65±0.10 ^b^ | 0.69±0.07 ^b^ | 0.68±0.12 ^b^ | 0.002 | 3 |
|  |  |  |  | Diet | | | | |
| **Contig** | **Protein Description** | **Symbol** | **D1** | **D2** | **D3** | **D4** | ***p* value** | **Cluster** |
| C2_885 | Bleomycin hydrolase | BLMH | 2.04±0.29 ^a^ | 1.06±0.18 ^b^ | 0.95±0.16 ^b^ | 1.44±0.33 ^ab^ | 0.025 | 1 |
| C2_20741 | Cadherin-23 | CDH23 | 1.99±0.70 ^a^ | 0.66±0.15 ^b^ | 0.34±0.07 ^b^ | 0.61±0.21 ^b^ | 0.024 | 3 |
| C2_5783 | Cadherin-related family member 2 | CDHR2 | 1.92±0.27 ^a^ | 0.94±0.25 ^ab^ | 0.70±0.14 ^b^ | 1.02±0.26 ^ab^ | 0.009 | 1 |
| C2_103184 | Calmodulin | CALM1 | 0.53±0.06 ^a^ | 1.31±0.16 ^b^ | 1.13±0.18 ^b^ | 0.93±0.22 ^ab^ | 0.021 | 2 |
| C2_1023 | Calreticulin | CALR | 0.86±0.08 ^a^ | 1.24±0.13 ^ab^ | 2.01±0.35 ^b^ | 1.31±0.31 ^ab^ | 0.027 | 4 |
| C2_1812 | Carboxymethylenebutenolidase homolog | CMBL | 2.32±0.49 ^a^ | 1.25±0.25 ^ab^ | 0.92±0.22 ^b^ | 1.37±0.33 ^ab^ | 0.05 | 1 |
| C2_997 | Catalase | CAT | 0.64±0.05 ^a^ | 1.01±0.09 ^ab^ | 1.16±0.15 ^b^ | 0.88±0.16 ^ab^ | 0.043 | 2 |
| C2_23957 | Catenin delta-1 | CTNND1 | 0.54±0.08 ^a^ | 0.62±0.09 ^a^ | 1.46±0.14 ^b^ | 0.87±0.18 ^a^ | 0.0002 | 2 |
| C2_12123 | Chymotrypsin A | CTRB1 | 3.18±0.58 ^a^ | 0.81±0.10 ^b^ | 1.02±0.40 ^ab^ | 1.77±0.63 ^ab^ | 0.009 | 3 |
| C2_4647 | Chymotrypsin B | CTRB2 | 4.32±1.10 ^a^ | 0.74±0.10 ^b^ | 0.71±0.21 ^b^ | 2.69±1.23 ^ab^ | 0.016 | 1 |
| C2_8139 | Chymotrypsin-C | CTRC | 2.19±0.19 ^a^ | 0.67±0.09 ^b^ | 0.62±0.20 ^b^ | 1.75±0.80 ^ab^ | 0.037 | 1 |
| C2_6507 | Chymotrypsin-like elastase family member 3B | CELA3B | 2.84±0.64 ^a^ | 0.70±0.11 ^b^ | 0.56±0.10 ^b^ | 1.29±0.40 ^b^ | 0.002 | 1 |
| C2_4909 | Chymotrypsin-like protease CTRL-1 | CTRL | 2.88±0.68 ^a^ | 0.66±0.15 ^b^ | 0.81±0.27 ^b^ | 1.28±0.42 ^b^ | 0.006 | 1 |
| C2_1398 | Complement C3 | C3 | 1.06±0.11 ^a^ | 2.29±0.55 ^b^ | 0.93±0.12 ^a^ | 0.85±0.12 ^a^ | 0.008 | 3 |
| C2_9760 | Complement factor B | CFB | 0.84±0.12 ^a^ | 1.97±0.50 ^b^ | 0.95±0.14 ^a^ | 0.91±0.10 ^a^ | 0.023 | 2 |
| C2_1763 | D-dopachrome decarboxylase | DDT | 1.82±0.28 ^a^ | 1.26±0.18 ^ab^ | 0.86±0.12 ^b^ | 1.17±0.24 ^ab^ | 0.035 | 1 |
| C2_94566 | Deoxyribonuclease-1 | DNASE1 | 2.29±0.39 ^a^ | 0.65±0.12 ^b^ | 0.67±0.26 ^b^ | 1.11±0.42 ^ab^ | 0.005 | 1 |
| C2_1242 | Dipeptidyl peptidase 4 | DPP4 | 2.36±0.34 ^a^ | 1.10±0.17 ^b^ | 0.90±0.22 ^b^ | 1.33±0.42 ^ab^ | 0.014 | 1 |
| C2_29850 | Ectonucleotide pyrophosphatase/ phosphodiesterase family member 3 | ENPP3 | 2.48±0.47 ^a^ | 1.02±0.27 ^ab^ | 0.62±0.16 ^b^ | 1.23±0.47 ^ab^ | 0.012 | 1 |
| C2_2589 | Ectonucleotide pyrophosphatase/ phosphodiesterase family member 6 | ENPP6 | 1.87±0.21 ^a^ | 1.08±0.17 ^b^ | 0.81±0.16 ^b^ | 0.88±0.14 ^b^ | 0.001 | 3 |
| C2_4289 | Elastase-1 | CELA1 | 3.18±0.62 ^a^ | 0.60±0.06 ^b^ | 1.06±0.62 ^ab^ | 1.84±0.7 ^ab^ | 0.021 | 3 |
| C2_68 | Eukaryotic translation initiation factor 3 subunit M | EIF3M | 0.44±0.13 ^a^ | 1.39±0.19 ^b^ | 1.33±0.30 ^b^ | 0.76±0.19 ^ab^ | 0.013 | 2 |
| C2_207 | F-actin-capping protein subunit alpha-2 | CAPZA2 | 0.91±0.17 ^a^ | 1.80±0.25 ^b^ | 1.34±0.09 ^ab^ | 1.26±0.12 ^ab^ | 0.012 | 4 |
| C2_227 | F-actin-capping protein subunit beta | CAPZB | 0.79±0.06 ^a^ | 1.48±0.17 ^b^ | 1.09±0.07 ^b^ | 1.03±0.13 ^ab^ | 0.004 | 4 |
| FP339542 | Fibrinogen beta chain | FGB | 1.57±0.22 ^a^ | 1.05±0.14 ^ab^ | 0.77±0.17 ^b^ | 0.88±0.22 ^b^ | 0.032 | 3 |
| C2_6274 | Filamin-A | FLNA | 1.80±0.26 ^a^ | 1.02±0.10 ^b^ | 1.00±0.25 ^b^ | 1.00±0.19 ^b^ | 0.034 | 3 |
| C2_6545 | Fructose-1,6-bisphosphatase 1 | FBP1 | 0.61±0.08 ^a^ | 1.79±0.26 ^b^ | 1.33±0.14 ^b^ | 1.11±0.28 ^ab^ | 0.006 | 4 |
| AM954422 | Gamma-glutamylaminecyclotransferase A | GGACT | 1.38±0.22 ^a^ | 1.47±0.18 ^a^ | 0.90±0.04 ^ab^ | 0.89±0.14 ^b^ | 0.033 | 3 |
| C2_63 | Gamma-interferon-inducible lysosomal thiol reductase | IFI30 | 2.26±0.35 ^a^ | 1.00±0.08 ^ab^ | 0.80±0.29 ^b^ | 1.29±0.40 ^ab^ | 0.015 | 1 |
| C2_265 | Gelsolin | GSN | 1.04±0.16 ^a^ | 1.74±0.15 ^b^ | 1.28±0.17 ^ab^ | 1.04±0.20 ^a^ | 0.028 |  |

|  |  |  |  |  |  |  |  |  |
| --- | --- | --- | --- | --- | --- | --- | --- | --- |
|  |  |  |  |  | Diet |  |  |  |
| **Contig** | **Protein Description** | **Symbol** | **D1** | **D2** | **D3** | **D4** | ***p* value** | **Cluster** |
| C2_296 | Glutathione peroxidase 2 | GPX2 | 2.08±0.30 ^a^ | 0.89±0.15 ^b^ | 0.92±0.21 ^b^ | 1.30±0.33 ^ab^ | 0.013 | 1 |
| C2_430 | Glutathione S-transferase kappa 1 | GSTK1 | 2.28±0.49 ^a^ | 1.00±0.12 ^b^ | 1.02±0.18 ^b^ | 1.20±0.26 ^b^ | 0.018 | 3 |
| C2_868 | Glutathione S-transferase Mu 3 | GSTM3 | 0.83±0.22 ^a^ | 1.62±0.17 ^b^ | 1.15±0.11 ^ab^ | 0.92±0.26 ^a^ | 0.044 | 2 |
| C2_17 | Glyceraldehyde-3-phosphate dehydrogenase | GAPDH | 2.67±0.56 ^a^ | 1.52±0.23 ^b^ | 1.58±0.07 ^b^ | 2.71±0.35 ^a^ | 0.035 | 1 |
| C2_762 | Guanidinoacetate N-methyltransferase | GAMT | 0.71±0.05 ^a^ | 1.79±0.21 ^b^ | 1.37±0.32 ^ab^ | 1.22±0.28 ^ab^ | 0.034 | 4 |
| C2_58 | Heat shock cognate 70 kDa protein | MED37C | 0.79±0.04 ^a^ | 1.58±0.17 ^b^ | 1.61±0.35 ^ab^ | 1.25±0.17 ^ab^ | 0.045 | 4 |
| C2_11749 | High choriolytic enzyme 1 | HCEA | 3.01±0.40 ^a^ | 0.65±0.08 ^b^ | 0.90±0.41 ^b^ | 1.70±0.59 ^ab^ | 0.003 | 1 |
| C2_9935 | Histidyl-tRNA synthetase, cytoplasmic | HARS2 | 0.86±0.11 ^a^ | 1.27±0.12 ^b^ | 1.31±0.09 ^b^ | 0.84±0.16 ^a^ | 0.019 | 2 |
| C2_29246 | Histone deacetylase 9-B | HDAC9B | 1.26±0.46 ^a^ | 16.35±3.14 ^b^ | 7.86±2.4 ^ab^ | 6.91±5.53 ^ab^ | 0.04 | 4 |
| C2_3677 | Histone H1 | H1F0 | 1.15±0.19 ^ab^ | 0.59±0.07 ^a^ | 0.96±0.13 ^a^ | 1.63±0.29 ^b^ | 0.007 | 1 |
| C2_25525 | Host cell factor 1 | HCFC1 | 2.28±0.28 ^a^ | 1.59±0.42 ^ab^ | 0.76±0.13 ^b^ | 1.14±0.27 ^ab^ | 0.011 | 1 |
| C2_23228 | Hyaluronidase-1 | HYAL1 | 2.42±0.44 ^a^ | 0.92±0.12 ^b^ | 0.85±0.27 ^b^ | 1.45±0.41 ^ab^ | 0.012 | 1 |
| C2_679 | Hypoxanthine-guanine phosphoribosyltransferase | HPRT1 | 0.46±0.06 ^a^ | 1.66±0.21 ^b^ | 1.01±0.22 ^ab^ | 1.09±0.28 ^ab^ | 0.006 | 4 |
| C2_13391 | IgGFc-binding protein | FCGBP | 2.12±0.13 ^a^ | 0.99±0.2 ^b^ | 1.09±0.36 ^ab^ | 1.25±0.36 ^ab^ | 0.038 | 3 |
| C2_14281 | Importin-7 | IPO7 | 0.49±0.13 ^a^ | 0.88±0.19 ^a^ | 1.79±0.31 ^b^ | 0.76±0.34 ^a^ | 0.011 | 2 |
| C3_lrc30120 | Inter-alpha-trypsin inhibitor heavy chain H3-like | ITIH3 | 1.06±0.07 ^a^ | 1.98±0.39 ^b^ | 1.00±0.1 ^a^ | 1.00±0.19 ^a^ | 0.014 |  |
| C2_2160 | Isovaleryl-CoA dehydrogenase, mitochondrial | IVD | 2.14±0.32 ^a^ | 1.25±0.19 ^b^ | 1.2±0.34 ^b^ | 0.94±0.13 ^b^ | 0.021 | 3 |
| C2_86457 | Keratin, type I cytoskeletal 19 | KRT19 | 0.74±0.12 ^a^ | 1.25±0.10 ^b^ | 1.03±0.11 ^ab^ | 0.88±0.14 ^ab^ | 0.04 | 2 |
| C2_29495 | Kininogen | KNG1L1 | 1.82±0.26 ^a^ | 1.13±0.15 ^b^ | 1.07±0.09 ^b^ | 0.90±0.23 ^b^ | 0.016 | 3 |
| C2_17959 | Lactase-phlorizin hydrolase | LCT | 2.35±0.31 ^a^ | 1.08±0.18 ^b^ | 1.05±0.26 ^b^ | 1.16±0.40 ^b^ | 0.016 | 1 |
| C2_5123 | Lactase-phlorizin hydrolase | LCT | 2.11±0.26 ^a^ | 0.97±0.18 ^b^ | 0.89±0.23 ^b^ | 1.12±0.39 ^ab^ | 0.018 | 1 |
| C2_380 | Lactoylglutathione lyase | GLO1 | 1.77±0.23 ^a^ | 0.99±0.15 ^b^ | 0.94±0.19 ^b^ | 1.26±0.27 ^b^ | 0.045 | 1 |
| C2_141 | Latexin | LXN | 1.46±0.16 ^a^ | 1.26±0.19 ^a^ | 0.83±0.13 ^b^ | 0.84±0.18 ^b^ | 0.034 | 3 |
| C2_2628 | Leukocyte elastase inhibitor | SERPINB1 | 2.50±0.48 ^a^ | 1.14±0.26 ^b^ | 1.17±0.26 ^b^ | 1.58±0.36 ^ab^ | 0.047 | 3 |
| C2_1393 | L-lactate dehydrogenase A chain | LDHA | 0.83±0.06 ^a^ | 1.76±0.09 ^c^ | 1.28±0.19 ^b^ | 1.03±0.12 ^ab^ | 0.0003 | 4 |
| C2_1403 | Meprin A subunit alpha | MEP1A | 2.74±0.63 ^a^ | 0.84±0.07 ^b^ | 0.81±0.27 ^b^ | 0.85±0.19 ^b^ | 0.002 | 3 |
| C2_5461 | Meprin A subunit beta | MEP1B | 2.49±0.49 ^a^ | 0.88±0.13 ^b^ | 0.92±0.19 ^b^ | 1.07±0.24 ^b^ | 0.003 | 1 |
| C2_1615 | Mucin-13 | MUC13 | 1.93±0.27 ^a^ | 1.17±0.18 ^ab^ | 0.86±0.16 ^b^ | 1.15±0.20 ^ab^ | 0.01 | 1 |
| C2_4414 | Niemann-Pick C1-like protein 1 | NPC1L1 | 2.47±0.54 ^a^ | 1.14±0.12 ^b^ | 0.95±0.38 ^b^ | 1.21±0.31 ^b^ | 0.033 | 1 |
|  |  |  |  |  | | | | |
|  |  |  |  |  | | | | |
|  |  |  |  | Diet | | | | |
| **Contig** | **Protein Description** | **Symbol** | **D1** | **D2** | **D3** | **D4** | ***p* value** | **Cluster** |
| C2_183 | Non-specific cytotoxic cell receptor protein 1 homolog | NCCRP1 | 1.84±0.23 ^a^ | 0.92±0.09 ^b^ | 0.73±0.13 ^b^ | 1.25±0.31 ^ab^ | 0.006 | 1 |
| C2_3526 | Pancreatic alpha-amylase | AMY2 | 2.7±0.34 ^a^ | 0.63±0.10 ^b^ | 0.92±0.48 ^b^ | 1.60±0.68 ^ab^ | 0.019 | 1 |
| C2_428 | Peptidyl-prolyl cis-trans isomerase | FKBP4 | 0.8±0.22 ^a^ | 1.91±0.20 ^b^ | 1.42±0.29 ^ab^ | 0.93±0.33 ^a^ | 0.031 | 2 |
| C2_62152 | Peroxisomal acyl-coenzyme A oxidase 3 | ACOX3 | 0.48±0.14 ^a^ | 1.02±0.20 ^ab^ | 1.89±0.39 ^b^ | 1.04±0.32 ^ab^ | 0.017 | 4 |
| C2_4069 | Phenazine biosynthesis-like domain-containing protein 1 | PBLD1 | 2.21±0.33 ^a^ | 1.29±0.20 ^b^ | 1.12±0.11 ^b^ | 1.56±0.24 ^ab^ | 0.019 | 3 |
| C2_99 | Phosphatidylethanolamine-binding protein 1 | PEBP1 | 0.78±0.06 ^a^ | 1.44±0.19 ^ab^ | 1.09±0.07 ^b^ | 0.95±0.15 ^ab^ | 0.012 | 2 |
| C2_4374 | Phospholipase B-like 1 | PLBD1 | 1.95±0.22 ^a^ | 0.99±0.08 ^b^ | 0.92±0.22 ^b^ | 1.1±0.26 ^ab^ | 0.007 | 1 |
| C2_19159 | Phosphotriesterase-related protein | PTER | 1.88±0.32 ^a^ | 1.02±0.17 ^b^ | 0.94±0.13 ^b^ | 1.39±0.29 ^ab^ | 0.046 | 1 |
| C2_952 | Plastin-2 | LCP1 | 1.07±0.11 ^a^ | 1.84±0.12 ^b^ | 1.24±0.12 ^a^ | 0.96±0.11 ^a^ | 0.0001 | 2 |
| C2_4220 | Proteasome subunit beta type-2 | PSMB2 | 2.23±0.45 ^a^ | 1.10±0.21 ^ab^ | 0.88±0.13 ^b^ | 1.38±0.43 ^ab^ | 0.05 | 1 |
| C2_121103 | Proteasome subunit beta type-9 | PSMB9 | 1.78±0.24 ^a^ | 1.04±0.16 ^b^ | 0.83±0.14 ^b^ | 1.10±0.21 ^b^ | 0.012 | 1 |
| C2_586 | Putative phospholipase B-like 2 | PLBD2 | 1.97±0.39 ^a^ | 0.85±0.08 ^b^ | 0.68±0.10 ^b^ | 1.43±0.41 ^ab^ | 0.021 | 1 |
| C2_2726 | Putative serine protease K12H4.7 | K12H4.7 | 1.94±0.28 ^a^ | 0.94±0.18 ^ab^ | 0.53±0.11 ^b^ | 1.13±0.37 ^ab^ | 0.007 | 1 |
| C2_32535 | RalBP1-associated Eps domain-containing protein 1 | REPS1 | 1.63±0.12 ^a^ | 0.75±0.05 ^b^ | 0.67±0.15 ^b^ | 0.97±0.17 ^b^ | 0.0002 | 3 |
| C2_1647 | Regucalcin | RGN | 2.02±0.37 ^a^ | 1.11±0.16 ^ab^ | 0.86±0.15 ^b^ | 1.36±0.31 ^ab^ | 0.032 | 1 |
| C2_933 | Retinal dehydrogenase 2 | ALDH1A2 | 2.27±0.36 ^a^ | 1.48±0.19 ^ab^ | 1.18±0.10 ^b^ | 1.45±0.20 ^ab^ | 0.02 | 3 |
| C2_241 | Rho GDP-dissociation inhibitor 1 | ARHGDIA | 0.75±0.15 ^a^ | 1.88±0.12 ^b^ | 1.42±0.27 ^ab^ | 0.96±0.22 ^a^ | 0.003 | 2 |
| C2_2718 | Ribokinase | RBKS | 1.96±0.44 ^a^ | 1.23±0.11 ^ab^ | 0.80±0.16 ^b^ | 1.07±0.20 ^ab^ | 0.029 | 1 |
| C2_1438 | Sialidase-1 | NEU1 | 1.76±0.29 ^a^ | 0.82±0.10 ^b^ | 0.78±0.10 ^b^ | 1.26±0.39 ^b^ | 0.042 | 1 |
| C2_1290 | Sorbitol dehydrogenase | SORD | 1.67±0.21 ^a^ | 1.14±0.09 ^ab^ | 0.87±0.10 ^b^ | 1.17±0.17 ^ab^ | 0.011 | 1 |
| C2_368 | Superoxide dismutase [Cu-Zn] | SOD1 | 2.83±0.54 ^a^ | 1.47±0.29 ^ab^ | 0.96±0.24 ^b^ | 2.24±0.61 ^ab^ | 0.037 | 1 |
| C2_4089 | Sushi domain-containing protein 2 | SUSD2 | 1.79±0.27 ^a^ | 0.93±0.10 ^b^ | 0.92±0.21 ^b^ | 1.19±0.29 ^b^ | 0.047 | 1 |
| C2_2347 | Tetratricopeptide repeat protein 38 | TTC38 | 0.99±0.22 ^a^ | 1.83±0.14 ^b^ | 1.12±0.13 ^a^ | 1.00±0.21 ^a^ | 0.009 | 4 |
| C2_1974 | Toll-interacting protein | TOLLIP | 0.76±0.11 ^a^ | 1.15±0.07 ^b^ | 0.94±0.06 ^ab^ | 1.01±0.09 ^ab^ | 0.03 | 2 |
| C2_564 | Transaldolase | TALDO1 | 0.68±0.08 ^a^ | 1.76±0.29 ^b^ | 1.31±0.14 ^ab^ | 1.09±0.31 ^ab^ | 0.025 | 4 |
| C2_5754 | Trehalase | TREH | 2.04±0.42 ^a^ | 1.04±0.27 ^b^ | 0.89±0.36 ^b^ | 0.74±0.13 ^b^ | 0.035 | 3 |
| C2_1213 | Trypsin | TRY | 3.57±0.67 ^a^ | 0.48±0.11 ^b^ | 0.67±0.49 ^b^ | 2.31±0.96 ^ab^ | 0.008 | 1 |
| C2_84054 | Trypsin-1 | PRSS1 | 3.25±0.79 ^a^ | 0.62±0.10 ^b^ | 0.68±0.39 ^b^ | 1.43±0.52 ^b^ | 0.005 | 1 |
| C2_121419 | Trypsin-2 | PRSS2 | 1.49±0.32 ^a^ | 0.61±0.05 ^b^ | 0.48±0.14 ^b^ | 1.15±0.38 ^ab^ | 0.041 | 1 |
| C2_470 | Ubiquitin fusion degradation protein 1 homolog | UFD1L | 1.64±0.17 ^a^ | 1.06±0.10 ^b^ | 0.95±0.08 ^b^ | 1.2±0.22 ^ab^ | 0.024 | 1 |
|  |  |  |  |  | Diet |  |  |  |
| **Contig** | **Protein Description** | **Symbol** | **D1** | **D2** | **D3** | **D4** | ***p* value** | **Cluster** |
| C2_5227 | Ubiquitin-conjugating enzyme E2 D2 | UBE2D2 | 0.71±0.11 ^a^ | 1.17±0.09 ^b^ | 0.75±0.14 ^ab^ | 0.81±0.12 ^ab^ | 0.046 | 2 |
| C2_12213 | UDP-glucuronosyltransferase 1-9 | UGT1A9 | 1.05±0.16 ^ab^ | 0.84±0.12 ^a^ | 1.17±0.10 ^ab^ | 1.71±0.34 ^b^ | 0.043 |  |
| C2_10632 | Villin-1 | VIL1 | 0.74±0.04 ^a^ | 1.28±0.14 ^b^ | 1.57±0.18 ^b^ | 1.34±0.24 ^ab^ | 0.017 | 4 |
| C2_1420 | WD repeat-containing protein 1 | WDR1 | 2.51±0.61 ^a^ | 1.42±0.21 ^b^ | 1.23±0.14 ^b^ | 1.27±0.16 ^b^ | 0.044 | 3 |
| C2_2468 | Xaa-Pro aminopeptidase 2 | XPNPEP2 | 2.23±0.41 ^a^ | 1.26±0.28 ^b^ | 0.94±0.29 ^b^ | 0.87±0.17 ^b^ | 0.016 | 3 |
| C2_10177 | Xylose isomerase | XYLA | 1.41±0.20 ^a^ | 0.89±0.11 ^b^ | 0.86±0.10 ^b^ | 1.25±0.17 ^ab^ | 0.04 | 1 |
| C2_39339 | Zonadhesin | ZAN | 2.39±0.23 ^a^ | 0.88±0.25 ^b^ | 0.76±0.26 ^b^ | 1.09±0.34 ^b^ | 0.001 | 1 |
